# The Olfactory Bulb Facilitates Use of Category Bounds for Classification of Odorants in Different Intensity Groups

**DOI:** 10.3389/fncel.2020.613635

**Published:** 2020-12-11

**Authors:** Justin Losacco, Nicholas M. George, Naoki Hiratani, Diego Restrepo

**Affiliations:** ^1^Neuroscience Graduate Program, University of Colorado Anschutz Medical Campus, Aurora, CO, United States; ^2^Department of Cell and Developmental Biology, University of Colorado Anschutz Medical Campus, Aurora, CO, United States; ^3^Gatsby Computational Neuroscience Unit, University College London, London, United Kingdom

**Keywords:** decoding, oscillations, learning, go-no go, odorant concentration

## Abstract

Signal processing of odor inputs to the olfactory bulb (OB) changes through top-down modulation whose shaping of neural rhythms in response to changes in stimulus intensity is not understood. Here we asked whether the representation of a high vs. low intensity odorant in the OB by oscillatory neural activity changed as the animal learned to discriminate odorant concentration ranges in a go-no go task. We trained mice to discriminate between high vs. low concentration odorants by learning to lick to the rewarded group (low or high). We recorded the local field potential (LFP) in the OB of these mice and calculated the theta-referenced beta or gamma oscillation power (theta phase-referenced power, or tPRP). We found that as the mouse learned to differentiate odorant concentrations, tPRP diverged between trials for the rewarded vs. the unrewarded concentration range. For the proficient animal, linear discriminant analysis was able to predict the rewarded odorant group and the performance of this classifier correlated with the percent correct behavior in the odor concentration discrimination task. Interestingly, the behavioral response and decoding accuracy were asymmetric as a function of concentration when the rewarded stimulus was shifted between the high and low odorant concentration ranges. A model for decision making motivated by the statistics of OB activity that uses a single threshold in a logarithmic concentration scale displays this asymmetry. Taken together with previous studies on the intensity criteria for decisions on odorant concentrations, our finding suggests that OB oscillatory events facilitate decision making to classify concentrations using a single intensity criterion.

## Introduction

Associative learning and changes in attention modulate circuit activity in early sensory processing areas such as the OB (Doucette et al., 2011; Gschwend et al., 2015; Chu et al., 2016; Losacco et al., 2020), the lateral geniculate nucleus (Ling et al., 2015) and the primary visual cortex (Pakan et al., 2018; Henschke et al., 2020). For example, in the OB, mitral cells change firing frequency and synchrony as mice learn to distinguish a rewarded odor from an unrewarded one (Doucette et al., 2011; Gire et al., 2013; Gschwend et al., 2015). This process aids in signal processing and improves stimulus decoding from neural activity. In the olfactory system, studies of the effect of learning on stimulus processing have predominantly been performed within the context of stimulus discrimination: can the animal differentiate between two odorants? However, how the olfactory system learns to distinguish across stimulus intensity ranges of the same odorant remains underinvestigated. In particular, while behavioral studies indicate that animals use a single intensity criterion to learn to differentiate between concentration ranges (Wojcik and Sirotin, 2014), the changes in OB processing that underlie discrimination of odorant concentration ranges remain unknown. Here, we ask whether neural processing is altered when animals learn to discriminate between different odorant concentrations ranges in a go-no go task. Mice were presented with six logarithmically spaced odorant concentrations (Wojcik and Sirotin, 2014) and they were rewarded when they licked a spout for 2 s in the presence of either the three highest or three lowest odorant concentrations (the rewarded stimulus).

The response of mitral/tufted (M/T) cells to odorant concentration is converted to discrete samples through sniffing and the pattern of activity within a sniff carries information on odorant intensity (Gross-Isseroff and Lancet, 1988; Chalansonnet and Chaput, 1998; Bathellier et al., 2008; Zhou and Belluscio, 2012; Patterson et al., 2013; Mainland et al., 2014; Sirotin et al., 2015; Jordan et al., 2018a; Parabucki et al., 2019). In the resting animal odorant-induced changes in the activity of the M/T cells is largest in the first sniff, and decreases subsequently, consistent with olfactory adaptation (Lecoq et al., 2009; Sirotin et al., 2015). We have recently shown that after mice learned to differentiate odorants in the go-no go task, neural oscillations in the high gamma (65–95 Hz, refered to as gamma) and beta (15–30 Hz) bands (referenced to an underlying theta rhythm) encode information on the contextual relevance of the odorant: Is this odorant rewarded? (Losacco et al., 2020). This chunking of neural activity within different phases of the theta oscillations [called phase-amplitude coupling, or PAC (Tort et al., 2010)], is thought to convey different information based on which phase in the theta wave the gamma or beta oscillation occurs (O'Keefe and Recce, 1993; Skaggs et al., 1996; Buzsaki and Draguhn, 2004; Lisman, 2005). For example, in dorsal CA1 the information on encoding a reward location in a spatial navigation task is thought to be transmitted at the peak of theta, while the information on retrieval of the memory is thought to be transmitted in the trough of the theta oscillation (Siegle and Wilson, 2014). Here, we asked whether theta referenced PAC (tPAC) and theta phase referenced power (tPRP) of beta and gamma oscillations carries information on odor concentration in the go-no go concentration discrimination task and whether they change over the course of learning. Additionally, we investigated whether tPRP specifically encodes high vs. low odorant concentration ranges.

## Results

### Odor Concentration Range Go-No Go Discrimination Task

**Figure 1:**
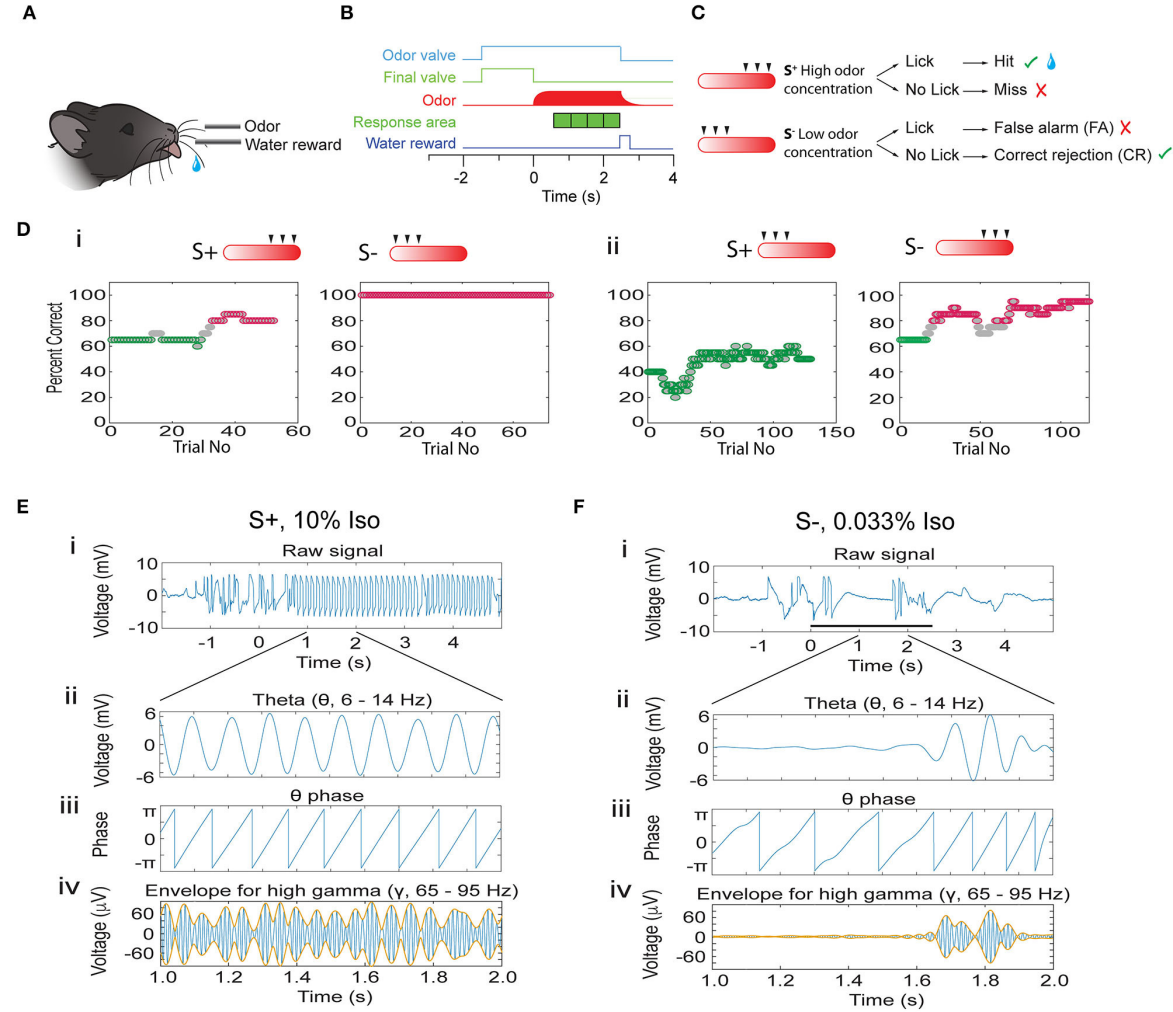
Introduction to the associative learning go-no go task for odorant concentration range discrimination and analysis of phase amplitude coupling for the LFP. **(A)** Water-deprived mice learn to lick on the water spout when presented with the rewarded stimuli (S+) in order to obtain a water reward. **(B)** Time-course for each trial. Immediately after the mouse starts the trial by licking on the water spout the final valve diverts the 2 L/min background air flow to the exhaust and the odor valve opens delivering 50 ml/min of odorant-saturated air. At the end of the final valve odorant equilibration period (random from 1 to 1.5 s) the final valve returns the air flow toward the mouse and the odorant concentration increases quickly. If the stimulus is the rewarded (S+) odorant the mouse must lick at least once in each 0.5 s response area (green cubes) to obtain a water reward. **(C)** Per trial behavioral outcome. The odorant was presented as air equilibrated with six different liquid dilutions (c_*liq*_) in mineral oil (10, 3.33, 1, 0.33, 0.1, and 0.033%). For the session shown here the three higher concentrations are the S+ and the three lower concentrations are the S- (the rewarded concentration range is switched for other sessions). When the animal licks at least once in each of the response areas when it is presented with the rewarded odorant the trial is a *Hit* and the mouse receives a water reward. If the mouse does not lick during one of the response areas it does not receive water (*Miss*). On the other hand, if the mouse refrains from licking in one of the response areas when presented with the S- stimulus the trial is a correct rejection (*CR*), and if the mouse licks in all response areas when presented the S- odorant the trial is a false alarm (*FA*). **(D)** Example for the performance of a mouse in two go-no go sessions where the rewarded stimuli belonged to the high concentration range **(i)** and in two sessions where the rewarded stimuli were the low concentration odorants **(ii)**. The percent correct is calculated in a sliding window of 20 trials. Green: percent correct ≤65%, light gray: percent correct >65%, and <80%, magenta: percent correct ≥80%. **(E,F)** Examples of the calculation of the theta phase and the theta amplitude envelope for the gamma LFP using the Hilbert transform. **(E)** S+, **(F)** S-. The top panels **(i)** show the LFP filtered from 4 to 100 Hz. Panel **(ii)** shows the LFP filtered with a theta bandpass (4–12 Hz). Panel **(iii)** shows the phase of the theta LFP calculated with the Hilbert transform. Finally, panel **(iv)** shows the LFP filtered with a gamma bandpass (65–95 Hz, blue) and the theta envelope calculated with the Hilbert transform (orange).

Do oscillations in the OB encode odorant concentration ranges and does the accuracy of encoding change as mice learn to discriminate in the go-no go task? We used tetrodes to record LFP oscillations in the OB of mice learning to associate a range of odorant concentrations with a water reward (go-no go odor concentration range task). In this task, mice which licked at least once in each of four 0.5 s time segments when the rewarded stimulus (S+) was presented received a water reward and the response was classified as a *Hit* (Figures 1A–C). A lack of licking in any of the time segments in the S+ segment was classified as a *Miss*. The mouse did not receive a reward if it licked for the unrewarded (S-) stimulus. Licking during S- was classified as a *false alarm* (FA), and correctly refraining from licking during S- trials was classified as a *correct rejection* (CR) (Figure 1C). We presented the animal with six different odorant concentrations generated by bubbling air into mineral oil containing different liquid dilutions (c_*liq*_) of either isoamyl acetate or acetophenone (0.033, 0.1, 0.33, 1, 3.3, and 10%). The rewarded stimulus was either the higher concentrations (1–10% c_*liq*_, Figure 1C) or the lower concentrations (0.033–0.33%). Figure 1D shows examples of behavioral performance in this go-no go task. Behavioral performance was quantified by the percent correct responses (both Hits and CRs) computed in a sliding window of 20 trials (see Methods). Mice learned to differentiate between concentration ranges regardless of whether the rewarded stimulus was the high (Figure 1Di) or the low concentration range (Figure 1Dii, also see Supplementary Figure 1). We focused on characterizing differences in OB oscillations between naïve animals (≤65% correct behavioral performance, green points in Figure 1D) and proficient mice (≥80% correct, magenta points in Figure 1D).

### The Strength of Phase Amplitude Coupling of Beta and Gamma Oscillations to Theta Increases When the Animal Becomes Proficient in the Concentration Go-No Go Task

Previous studies of LFP oscillations found strong coupling of gamma and beta oscillation bursts within specific phases of the lower frequency theta oscillations (Buonviso et al., 2003; Rojas-Libano et al., 2014; Losacco et al., 2020) and that these theta phase-amplitude coupled (PAC) gamma/beta oscillations encode for odorant contextual identity (Losacco et al., 2020). To quantify these phase-coupled oscillations we performed PAC analysis (Tort et al., 2010). We extracted the phase of the low frequency theta oscillation using a Hilbert transform of the theta-filtered LFP trace, thereby identifying the peak and trough of theta (Figures 1E,F,ii,iii). We estimated the amplitude of the theta-referenced fluctuations of beta and gamma oscillations by computing the theta envelope of the beta and gamma LFP using a Hilbert transform (Figures 1E,F,iv, yellow line).

**Figure 2:**
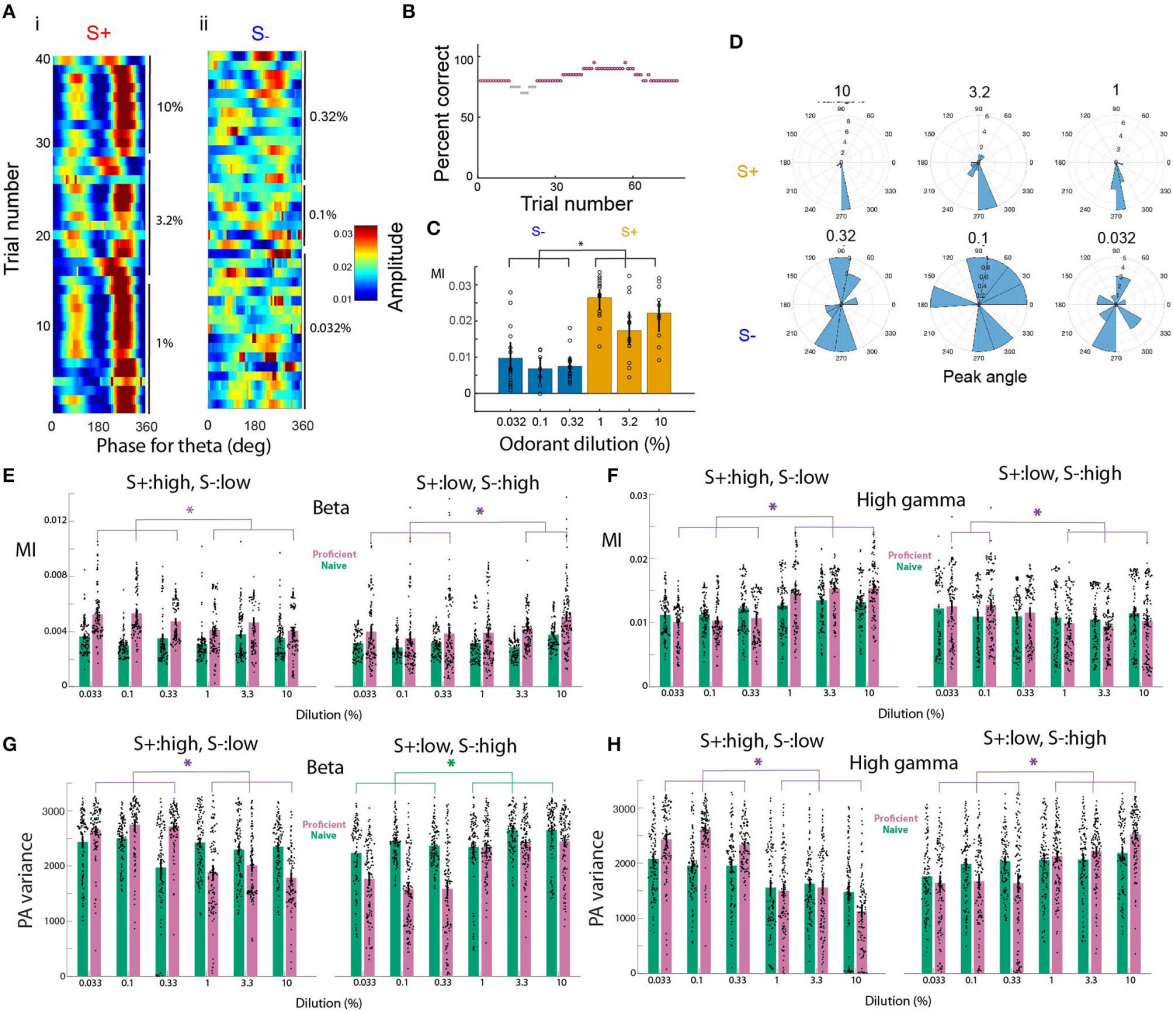
Phase amplitude coupling for the go-no go odorant concentration task. **(A–D)** tPAC analysis shown for a go-no go session where the mouse was proficient in differentiating between the high concentration odorants (c_*liq*_ 1–10%, rewarded stimulus, S+) and the low concentration odorants (c_*liq*_ 0.033–0.33%, unrewarded stimulus, S-). **(A)** Pseudocolor image showing the per-trial average amplitude for the theta envelope for the gamma LFP. The odorant used was isoamyl acetate. Odorant dilutions (c_*liq*_) for S+ were 10, 3.33, and 1% and for S- were 0.33, 0.1, and 0.033%. **(B)** Percent correct as a function of trial number. Light gray: percent correct >65% and <80%, magenta: percent correct ≥80%. **(C)**. Strength of tPAC quantified as the modulation index (MI) displayed for the six odorant dilutions. A GLM analysis indicated that there is a difference between S+ and S- (*p* < 0.001, 77 trials, 71 d.f., F-Statistic 18.3, *p*-value for the model < 0.001). The asterisk denotes *post-hoc* differences evaluated with either *t*-test or ranksum, *p* < pFDR = 0.033. **(D)** Rose plot histograms for the peak phase angle for gamma tPAC shown in **(A)**. **(E,F)** Modulation index quantifying the strength of tPAC. The bars show the average MI per electrode for all mice (*n* = 16 electrodes and nine mice). The violin plot shows the MI per electrode for each mouse. Green bars are the MI for naïve mice (percent correct ≤ 65%) and magenta bars are the MI for proficient mice (>80%). E. MI for beta, F. MI for gamma. For the beta tPAC a GLM finds statistically significant differences between odorant concentrations and between naïve and proficient, but not between naïve and proficient and for the interaction between naïve vs. proficient and concentration (*p* < 0.001, 2,784 observations, 2,776 d.f., F-statistic = 61.2, *p* < 0.001, nine mice). For the gamma tPAC a GLM finds statistically significant differences between odorant concentrations, between naïve and proficient and between naïve and proficient (*p* < 0.001, 2,784 observations, 2,776 d.f., F-statistic = 47.5, *p* < 0.001, nine mice). **(G,H)** Variance of the peak angle for tPAC. The bars show the average peak angle variance per electrode for all mice (*n* = 16 electrodes and nine mice). The violin plot shows the peak angle variance per electrode for each mouse. Green bars are for naïve mice (percent correct ≤ 65%) and magenta bars are for proficient mice (>80%). **(G)** beta, **(H)** gamma. For the beta tPAC a GLM finds statistically significant differences between odorant concentrations, between naïve and proficient and between naïve and proficient (*p* < 0.001, 2,784 observations, 2,776 d.f., F-statistic = 100, *p* < 0.001, nine mice). For the gamma tPAC a GLM finds statistically significant differences between odorant concentrations, between naïve and proficient and between naïve and proficient (*p* < 0.001, 2,784 observations, 2,776 d.f., F-statistic = 99.2, *p* < 0.001, nine mice).

We have previously shown that tPAC changes as an animal learns to discriminate two distinct odors (Losacco et al., 2020). Here, we surveyed tPAC in the OB in response to odor concentrations and we found a difference in the strength of tPAC between the rewarded and unrewarded concentrations once the animals became proficient. Figure 2A shows in pseudocolor the amplitude of the gamma oscillations as a function of theta phase for the different trials in a session where the animal was proficient (percent correct ≥ 80%, see Figure 2B for percent correct as function of trial for this session). For this session the amplitude of the gamma oscillations peaked at about 280° for S+, while for S- the peak of the gamma amplitude shifted from trial to trial (red peak in Figure 2A and peak angle in Figure 2D). The strength of tPAC appeared to be stronger for S+ than for S- (Figure 2A). We quantified tPAC using the modulation index, a measure of how localized high frequency firing is within the phase of theta oscillations (Tort et al., 2010). In previous studies using a go-no go odor discrimination task, tPAC modulation index fell between 0.005 and 0.04 for the OB (Losacco et al., 2020). For this example session, we found that the modulation index was larger for the rewarded stimulus (in this case the high concentration range) (Figure 2C). A gerneralized linear model (GLM) analysis for the modulation index indicated that there was a statistically significant difference between S+ and S- (*p* < 0.001, 77 trials, 71 d.f., F-Statistic 18.3, *p*-value for the model <0.001, Supplementary Table 1). Furthermore, as shown in Figures 2A,D, the theta phase for the largest amplitude for the gamma oscillation (the peak angle) appeared to vary to a larger extent for the unrewarded stimuli. This means that a downstream observer quantifying the amplitude of gamma oscillations at a specific fixed theta phase (e.g., 280°) would be detecting high amplitude gamma power for the S+ odorant compared to S- because of the higher modulation index and the lower peak angle variance for the rewarded stimulus.

We next analyzed whether the modulation index changed as the animal learned to discriminate odorant concentration ranges in the go-no go task. For both beta and gamma tPAC we found that the modulation index increased when the animals learned to differentiate the concentration ranges and differed between rewarded and unrewarded concentration (Figures 2E,F, also see Supplementary Figure 2 for an example of tPAC for a session where the mouse was naïve). For the beta tPAC, a GLM found significant differences between odorant concentrations and between rewarded vs. unrewarded stimuli and for the interaction between naïve vs. proficient and concentration (Figure 2E, *p* < 0.001, 2,784 observations, 2,776 d.f., F-statistic = 61.2, *p* < 0.001, nine mice, Supplementary Table 2). The statistical significance of the *interaction* between naïve vs. proficient and concentration indicates that the effect of one causal variable on an outcome depends on the state of a second causal variable (e.g., in this case in Figure 2E the difference between naïve and proficient for S+:high is larger for the lower concentrations). For beta tPAC there was no significant difference between naïve and proficient (*p* > 0.05). For gamma tPAC, a GLM found significant differences for the modulation index between odorant concentrations, between rewarded vs. unrewarded stimuli, and between naïve vs. proficient mice (Figure 2F, *p* < 0.001, 2,784 observations, 2,776 d.f., F-statistic = 47.5, *p* < 0.001, nine mice, Supplementary Table 3). Finally, we quantified the variation in the peak angle to determine whether the peak angle shifted on a trial by trial basis as found for S- in Figure 2A. Peak angle variance was higher for the unrewarded odorant concentration range for both beta and gamma tPAC (Figures 2G,H). For the beta tPAC, a GLM finds for the peak angle variances statistically significant differences between odorant concentrations, between naïve vs. proficient and between rewarded vs. unrewarded stimuli (Figure 2G, *p* < 0.001, 2,784 observations, 2,776 d.f., F-statistic = 100, *p* < 0.001, nine mice, Supplementary Table 4). For gamma tPAC, a GLM finds statistically significant differences between odorant concentrations, naïve vs. proficient and between rewarded vs. unrewarded stimuli (Figure 2H, *p* < 0.001, 2,784 observations, 2,776 d.f., F-statistic = 99.2, *p* < 0.001, nine mice, Supplementary Table 5).

Therefore, the strength of tPAC and peak angle variance changed as the animal learned to discriminate between odor concentration ranges. Gamma tPAC tends to become stronger for the rewarded stimuli as the animal learns and the peak angle variance became larger for the unrewarded stimuli compared to the rewarded odorant concentrations. For beta tPAC, we found similar changes in the peak angle variance, and tPAC tended to become stronger for the unrewarded odorant. These observations raised the question of whether the power of beta or gamma oscillations within a theta phase window could be used to discriminate between concentration ranges.

### Learning Elicits an Increase in the Difference Between the Rewarded and Unrewarded Stimuli in Theta Phase-Referenced Power

**Figure 3:**
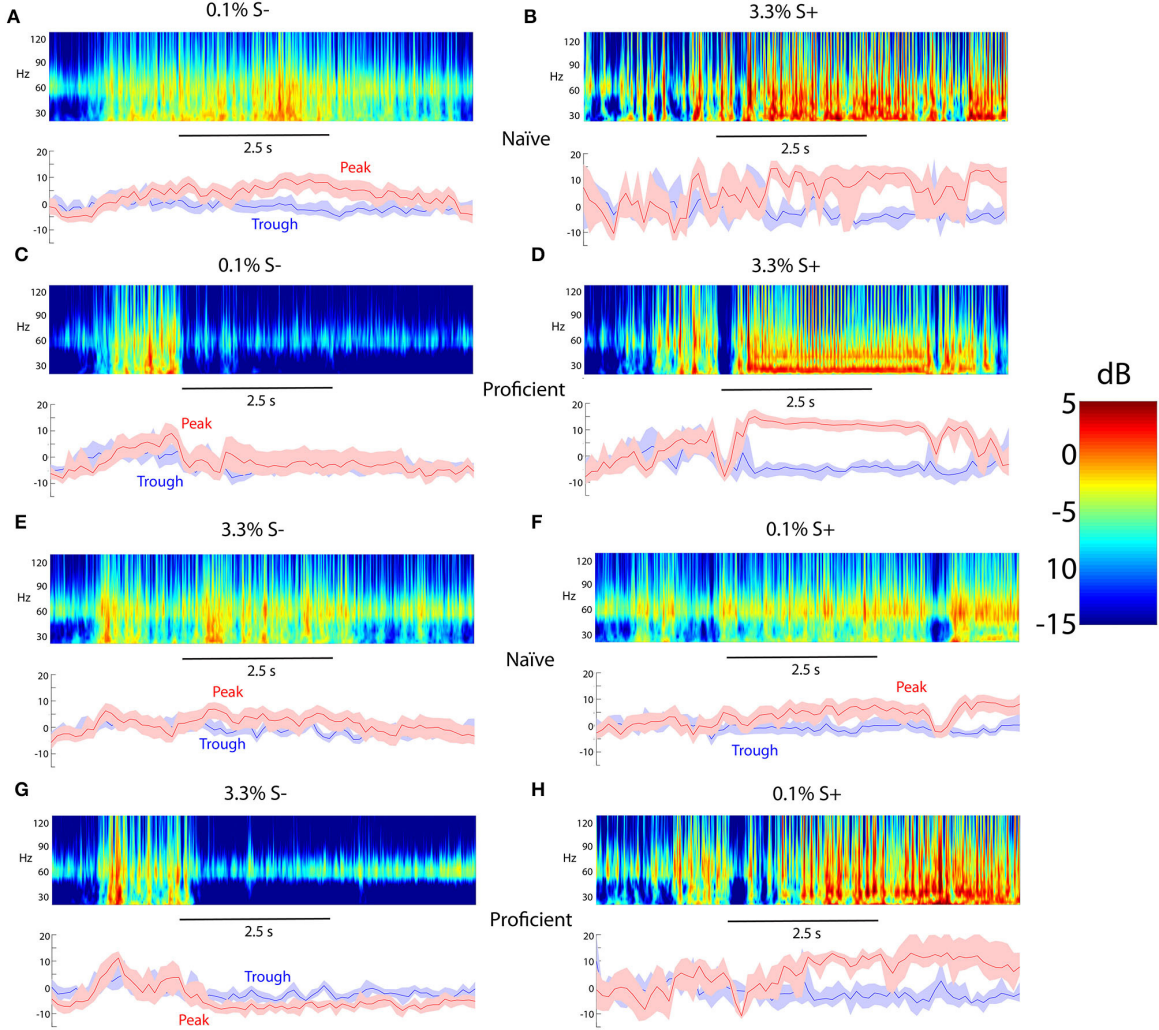
Examples of odorant-elicited changes in theta phase-referenced gamma power. **(A–D)** The rewarded stimulus (S+) is isoamyl acetate presented at a 3.3% c_*liq*_ in MO while the unrewarded odorant (S-) is isoamyl acetate at c_*liq*_ of 0.1% in MO. **(A,B)** Naïve mice. **(C,D)** Proficient mice. **(E–H)** The rewarded stimulus (S+) is acetophenone presented at a 0.1% c_*liq*_ while the unrewarded odorant (S-) is acetophenone at a dilution of 3.3% c_*liq*_. **(E,F)** Naïve mice. **(G,H)** Proficient mice. For each condition the top panel shows a pseudocolor figure representing the average power computed using wavelet analysis and the bottom panel shows the average gamma power referenced at either the peak or trough of the theta LFP oscillation. The shadow shows the 95% CI. The number of trials used for each condition are as follows: **(A)** 24, **(B)** 4, **(C)** 12, **(D)** 8, **(E)** 20, **(F)** 19, **(G)** 8, **(H)** 4. The 2.5 s time scale bar denotes the time for odorant application.

In earlier studies of the go-no go task where the animal learned to differentiate odorants we found that peak gamma tPRP conveyed more information on the rewarded stimulus than trough gamma tPRP (Losacco et al., 2020). We proceeded to quantify the power for beta and gamma oscillations referenced to the peak or trough of the theta LFP in the go-no go concentration task. Upper panels in Figure 3 show examples of the time course per trial for the power distribution estimated using a Morlet wavelet analysis. A Morlet wavelet is defined as a sine wave tapered by a Gaussian, and in the Morlet wavelet analysis LFP oscillations are fit to subsets of Morelet wavelets of different frequencies thereby quantifying the contribution of these wavelets to the oscillations. As performed with fast Fourier transform wavelet analysis allows evaluation of the power of LFP oscillations at different frequencies. However, unlike fast Fourier transform wavelet analysis is able to follow changes in power that occur in the theta frequency. The bottom panels show the gamma power referenced to the peak (red traces) or the trough (blue traces) of the theta LFP oscillation. Two stimuli belonging to either the unrewarded low concentration range (0.1% c_*liq*_ of acetopheonone) or the rewarded high concentration range (3.3% c_*liq*_) elicited relatively small changes in tPRP for the naïve animal (Figures 3A,B). In contrast, for the proficient animal the rewarded 3.3% c_*liq*_ stimulus elicits a substantial increase in peak-referenced tPRP (Figure 3D, red trace) and the unrewarded 0.1% c_*liq*_ stimulus elicits a decrease in tPRP (Figure 3C, red trace), while the trough-referenced changes in tPRP are small (blue traces in Figures 3C,D). Similar results are shown in Figures 3E–H for a session where the rewarded stimuli were the low concentration odorants.

**Figure 4:**
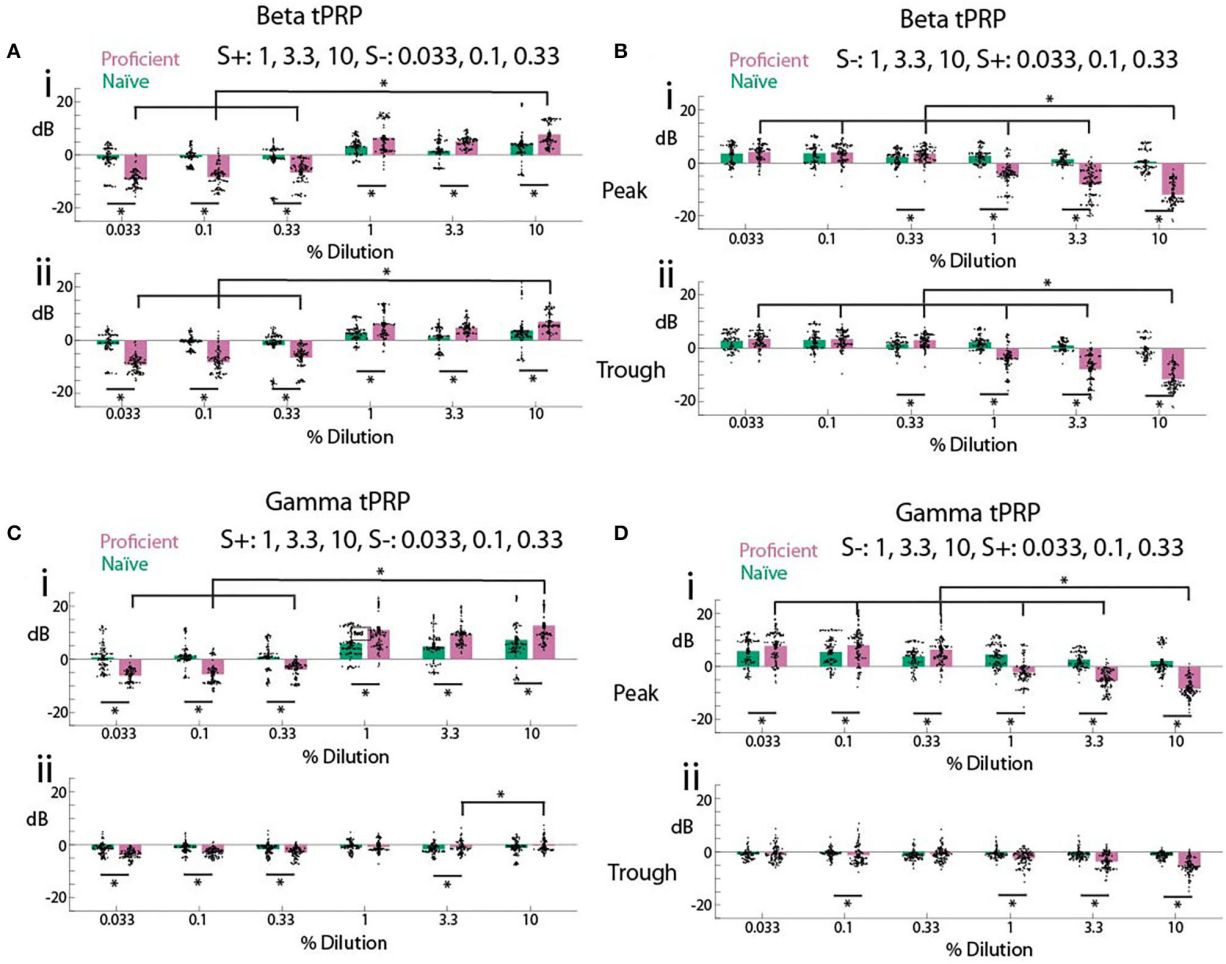
Summary of theta phase-referenced power. **(A,B)**. Beta tPRP. **(C,D)** Gamma tPRP. Top panels **(i)** are referenced to the peak of the theta oscillation while bottom panels **(ii)** are referenced to the trough. **(A,C)** are tPRP for sessions where the three highest dilutions were the rewarded odorant while B and D are tPRP for sessions where the three lowest dilutions were the rewarded odorant. tPRP is shown for naïve mice (percent correct behavior ≤ 65%, green bars) and proficient mice (>80%, magenta bars). For the beta tPRP **(A,B)** a GLM yielded significant differences between rewarded vs. unrewarded stimuli, proficient vs. naïve and high dilution rewarded vs. low dilution rewarded (*p* < 0.001, 5,568 observations, 5,552 d.f., F-statistic = 525, *p* < 0.001, nine mice), and there was no statistical significance between peak and trough tPRP (*p* > 0.05, 5,568 observations, 5,552 d.f., F-statistic = 525, *p* < 0.001, nine mice). For the gamma tPRP **(C,D)** a GLM yielded significant differences between rewarded vs. unrewarded stimuli, proficient vs. naïve, high dilution rewarded vs. low dilution rewarded and peak vs. trough (*p* < 0.001, 5,568 observations, 5,552 d.f., F-statistic = 525, *p* < 0.001, nine mice). Asterisks show significant differences in a *post-hoc t*-test or ranksum test with *p*-values < pFDR. pFDR was 0.044 for peak beta tPRP, 0.043 for trough beta tPRP, 0.043 for peak gamma tPRP and 0.029 for trough gamma tPRP. The only comparisons that are shown are between the highest c_*liq*_ and all other c_*liq*_ and between naïve and proficient for each c_*liq*_.

We proceeded to evaluate the difference in odorant-elicited changes in tPRP between naïve and proficient mice for beta and gamma tPRP. Furthermore, we asked whether these changes in tPRP were similar when the rewarded odorant was either the high or the low concentration range of odorant stimuli. When the high concentration range was rewarded there was a clear increase in beta tPRP for the rewarded stimulus, and a decrease for the unrewarded stimulus for proficient compared to naïve animals (Figure 4A). These changes in beta tPRP were similar for peak- and trough-referenced tPRP (Figure 4Ai vs. Figure 4Aii). When the rewarded stimulus was the low concentration range there was also a clear decrease in beta peak or trough-referenced tPRP for the unrewarded stimuli (in this case the high concentration odorants), but for the unrewarded high concentration range the proficient vs. naïve difference increased as the concentration increased, and there was not a substantial difference in tPRP between naïve and proficient for the rewarded low concentration range (S+, Figure 4B). For beta tPRP we found statistically significant differences between rewarded vs. unrewarded stimuli, proficient vs. naïve and for concentration (GLM, *p* < 0.001, 5,568 observations, 5,552 d.f., F-statistic = 525, *p* < 0.001, nine mice, Supplementary Table 6, Figures 4A,B), and there was no statistical significance between peak and trough tPRP (GLM, *p* > 0.05, 5,568 observations, 5,552 d.f., F-statistic = 525, *p* < 0.001, nine mice). *Post-hoc* ranksum or *t*-tests yielded differences between naïve and proficient for all concentrations for the experiments where the high concentrations were rewarded, but did not yield significant difference for the lowest two concentrations when the low concentrations were rewarded (asterisks in Figures 4A,B, *p*-values < pFDR. pFDR was 0.044 for peak beta tPRP, 0.043 for trough beta tPRP).

In contrast, there was a clear difference between peak-referenced and trough-referenced gamma tPRP in their dependence on learning (Figures 4C,D). When the high concentration range was rewarded there was a clear increase for peak gamma tPRP for the rewarded stimulus, and a decrease for the unrewarded stimulus as the animal learned to discriminate odorant concentrations (Figure 4Ci). Interestingly, the learning induced changes in gamma tPRP did not take place for trough-referenced tPRP (Figure 4Cii). Similar to theta-beta tPRP, when the rewarded stimulus was the low concentration range there was also a clear decrease in peak-referenced gamma tPRP for the unrewarded stimuli, and the proficient vs. naïve difference increased as the concentration increased (Figure 4Di). There were small changes in trough-referenced gamma tPRP (Figure 4Dii). For the gamma tPRP (Figures 4C,D) there was a significant differences between rewarded vs. unrewarded stimuli, proficient vs. naïve, concentration and peak vs. trough (Figures 4C,D, GLM, *p* < 0.001, 5,568 observations, 5,552 d.f., F-statistic = 525, *p* < 0.001, nine mice, Supplementary Table 7).

Thus, as the animal learns to discriminate rewarded and unrewarded odor concentration ranges (transitions from naïve to proficient), both beta and gamma tPRP increase. This raises the question whether the proficient animal's tPRP encodes the identity of the rewarded stimulus irrespective of odor concentration. Surprisingly, when the rewarded stimulus is the low concentration range the tPRP depends on odorant concentration, whereas we did not see this effect when the rewarded stimulus is the high odorant concentration range.

### Learning Elicits a Robust Increase in the Discriminability of Reinforced Stimuli for Theta Phase-Referenced Power in the Go-No Go Task

**Figure 5:**
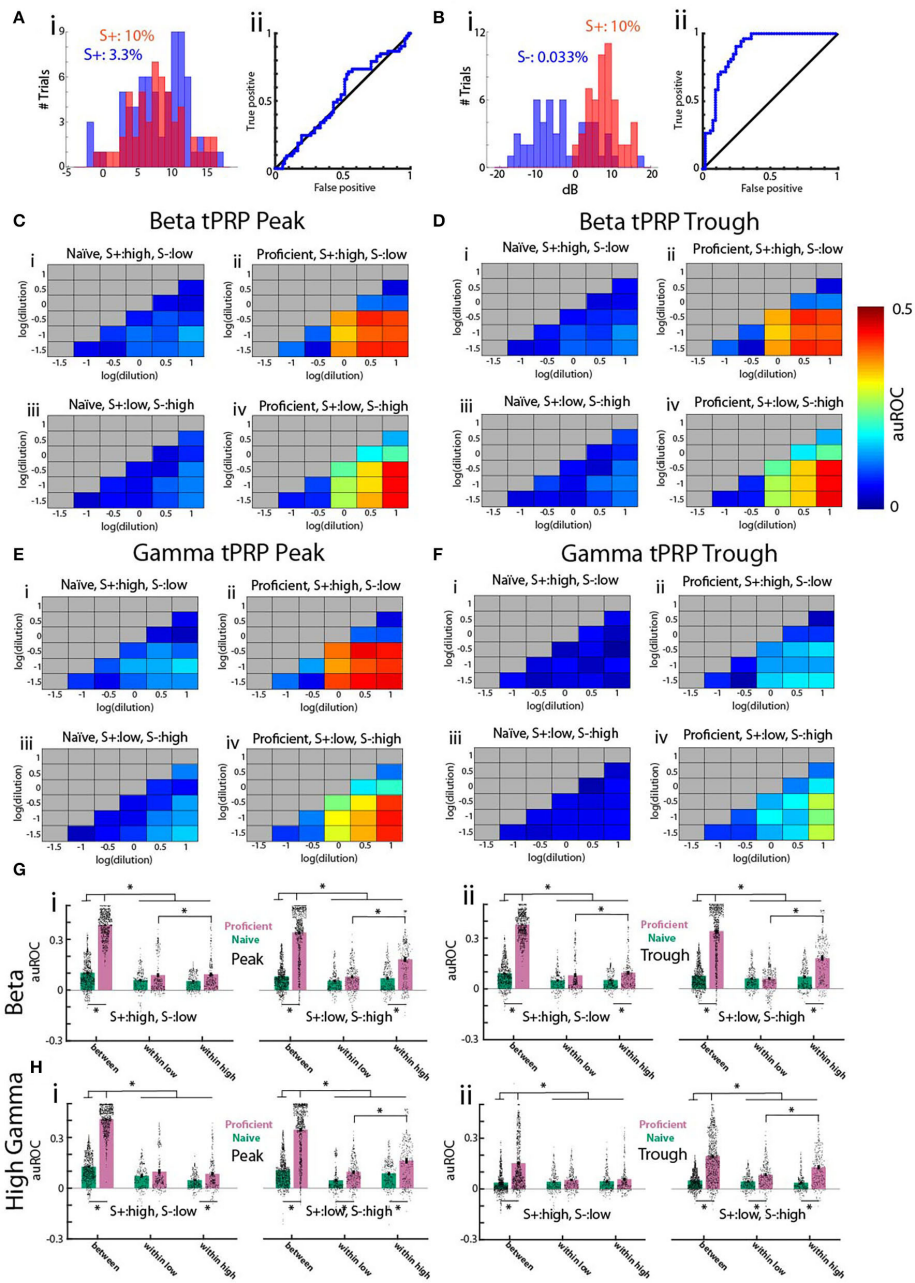
Receiver operating characteristic (ROC) analysis of the theta phase-referenced power for the go-no go task for odorant concentration. **(A,B)** Examples of histograms of gamma tPRP **(i)** and ROC curve **(ii)** for all proficient trials for acetophenone for one mouse. The rewarded stimuli (S+) were c_*liq*_ ≥ 1%. **(A)** Histogram and ROC for 53 trials with 10% c_*liq*_ and 72 trials with 3.3% c_*liq*_. **(B)** Histogram and ROC for 53 trials with 0.033% c_*liq*_ and 53 trials with 10% c_*liq*_. **(C–F)** Pseudocolor diagrams showing the average area under the ROC curve (auROC) for each pairwise comparison for all odorant concentrations for gamma tPRP power. An auROC of 0.5 indicates non-overlapping distributions, and an auROC of 0 indicates completely overlapping distributions. **(C,D)** are for beta tPRP and **(E,F)** are for gamma. **(C,E)** are peak-referenced tPRP and **(D,F)** are trough-referenced tPRP. **(i,ii)** are naïve **(i)** and proficient **(ii)** trials for experiments where the three highest concentrations (c_*liq*_ of 10, 3.3, and 1%) were the rewarded odorant (S+) and iii and iv were experiments where the three lowest concentrations (c_*liq*_ of 0.33, 0.1, and 0.033%) were the rewarded odorant. The average was calculated for each of the 16 electrodes for nine mice. **(G,H)** Bar graphs showing the average auROC for pairwise comparisons for all odorant concentrations for beta **(E)** or gamma **(F)** tPRP power for nine mice and 16 electrodes per mouse. Points in the violin plot shows the average auROC per electrode per mouse. **(i)** is peak-referenced tPRP and **(ii)** is trough-referenced tPRP. Each panel shows on the left the results per electrode for experiments where the three highest concentrations (c_*liq*_ of 10, 3.3, and 1%) were the rewarded odorant (S+, left) and on the right the results for experiments where the three lowest concentrations (c_*liq*_ of 0.33, 0.1, and 0.033%) were the rewarded odorant. The pairwise comparisons were grouped as comparisons between the low and high concentration ranges (between), between all concentrations within the low concentration range (within low) and between all concentrations within the high concentration range (within high). A GLM for the beta tPRP auROC yielded significant differences for proficient vs. naïve and high S+ vs. low S+ (*p* < 0.001, 17,888 observations, 17,964 d.f., F-statistic = 1,180, *p*-value for F-statistic < 0.001), but did not yield a statistical significance between peak and trough (*p* > 0.05). A GLM for the gamma tPRP auROC yielded significant differences for proficient vs. naïve, peak vs. trough and high S+ vs. low S+ (*p* < 0.001, 17,888 observations, 17,964 d.f., F-statistic = 880, *p*-value for F-statistic < 0.001). Asterisks denote significant *post-hoc* statistical difference assessed with either *t*-test or ranksum tests corrected for multiple comparisons, pFDR = 0.039 (Ei), 0.04 (Eii), 0.036 (Fi), and 0.045 (Fii).

We proceeded to evaluate the discriminability of per trial tPRP values comparing tPRP for trials with rewarded stimuli vs. trials with unrewarded stimuli using receiver operating characteristic (ROC) analysis (Fawcett, 2006). Figures 5A,B show ROC analysis for two concentration pairs for a proficient mouse engaged in a go-no go session where the high concentration range was the rewarded stimulus. First, we checked whether animals could differentiate between odor concentrations within the rewarded concentration range. The gamma peak tPRP histograms for 10 and 3.3% c_*liq*_ (both S+) overlap (Figure 5A) and, as expected, the ROC curve falls along the diagonal, indicating that tPRP can not be used to distinguish these two concentrations. In contrast, for c_*liq*_ 10% (S+) and 0.033% (S-) the gamma peak tPRP histograms are largely non-overlapping, reflecting better ability to discriminate as the ROC curve rises away from the diagonal. We defined the area under the ROC curve (auROC) as 0 when the curve falls along the diagonal and the stimuli cannot be differentiated and 0.5 when it lays along the top left axes, when the tPRP values are distinct for the two stimuli. For the ROC in Figure 5A the auROC is 0.02 and for the ROC in Figure 5B it is 0.38. Thus, ROC quantifies the extent of overlap between the two distributions.

To quantify the difference between tPRP distributions for all combinations of two odorant concentrations we calculated auROC for each electrode, for each of nine mice for trials when the mouse was naïve or proficient. The average auROC values for all combinations of odorant concentrations for theta-gamma tPRP are shown in pseudocolor in Figures 5C–F. To gauge the differences in auROC for different pairs of odorant concentrations, we display the average auROC in a bar graph and individual auROCs per electrode per mouse in a violin plot (Figures 5G,H). We group concentration pairs in the bar graph/violin plot as those within the high or low concentration ranges (labeled “within high” and “within low”) or the pairs with one concentration belonging to the high or low concentration ranges (labeled “between”). We found a large increase in auROC for both beta and gamma for the between group when the animals become proficient (compare Figures 5C–F panels i,iii with panels ii,iv and in Figures 5G,H compare green with magenta bars). Interestingly, the auROC does not differ between within low and within high when the rewarded stimulus is the high concentration range (left panels in Figures 5Gi,ii,Hi,ii). However, within high auROCs are higher than within low auROCs when the rewarded stimulus is the low concentration range (left panels in Figures 5Gi,ii,Hi,ii). This indicates that in the latter case there is information on concentration in the within high group tPRP. When we asesssed beta tPRP auROC scores with a GLM, we noted significant differences for proficient vs. naïve and high S+ vs. low S+ (Figure 5G, *p* < 0.001, 17,888 observations, 17,964 d.f., F-statistic = 1,180, *p*-value for F-statistic < 0.001, Supplementary Table 8). There was no significant difference between peak and trough (*p* > 0.05). A GLM for the gamma tPRP auROC yielded significant differences for proficient vs. naïve, peak vs. trough and high S+ vs. low S+ (Figure 5H, *p* < 0.001, 17,888 observations, 17,964 d.f., F-statistic = 880, *p*-value for F-statistic < 0.001, Supplementary Table 9). Asterisks in Figures 5E,F denote significant *post-hoc* statistical difference assessed with either *t*-test or ranksum tests corrected for multiple comparisons (pFDR = 0.039 for Ei, 0.04 for Eii, 0.036 for Fi and 0.045 for Fii).

In summary, auROC analysis indicates that as the animal learns the tPRP for rewarded vs. unrewarded stimuli become different. In addition, when the low concentration odorants are the reinforced stimuli the tPRP values within the high concentration (S-) odorants maintain discriminability, albeit with a lower auROC compared to auROCs quantified for concentrations belonging to the rewarded and unrewarded groups. The fact that there were differences in auROC when the rewarded stimulus was either the high or low concentration range raised the question whether there were differences in decoding accuracy between these conditions.

### Performance for Decoding the Rewarded Stimulus From the Phase Referenced Power Increases as the Animal Learns and Differs Depending on the Assignment of the Reward to High vs. Low Concentration

**Figure 6:**
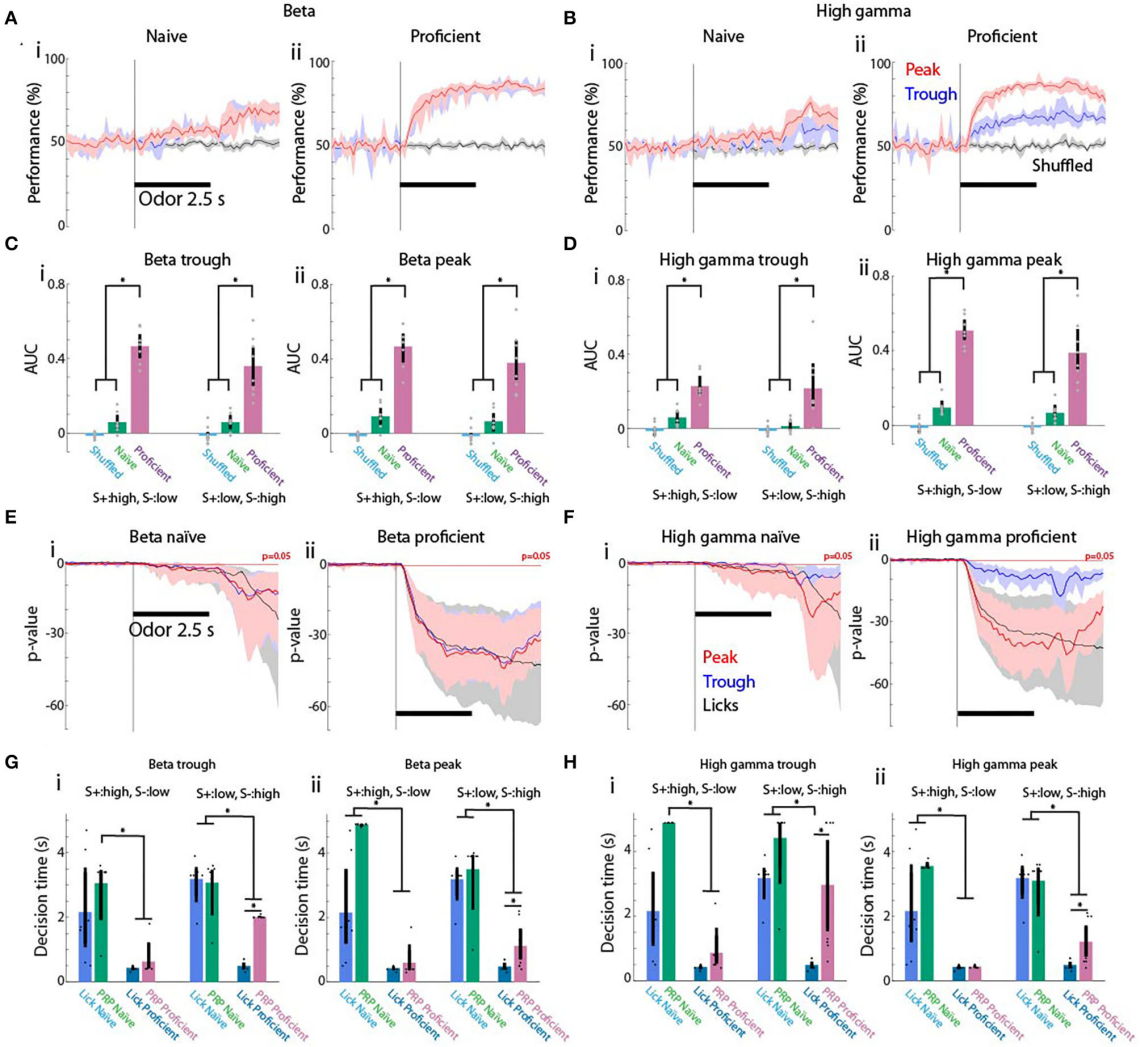
Discriminant analysis for decoding the rewarded stimulus in the odorant concentration go-no go task from the theta phase-referenced power. **(A,B)** Within trial time course for performance of the linear discriminant analysis (LDA) decoding of the rewarded stimulus from the tPRP evaluated for all trials for eight mice. **(A)** Beta tPRP. **(B)** Gamma tPRP. **(i)** Naïve. **(ii)** Proficient. Red denotes performance for the peak tPRP, blue the trough tPRP and black performance for shuffled stimulus reward. The shadow denotes the bootstrapped 95% CI, *n* = 8 mice. **(C,D)** Area under the curve (AUC) quantifying the performance of LDA decoding evaluated for all trials for eight mice. AUC is defined so that if the curve increases from 50 to 100% when the odorant is applied the AUC is 1, and when the curve is 50% throughout the AUC is zero. **(A)** Beta AUC. **(B)** Gamma AUC. **(i,ii)** AUC for LDA calculated with the trough **(i)** or peak **(ii)** tPRP. Magenta denotes proficient, green denotes naïve and light blue denotes shuffled. Vertical bars are the bootstrapped 95% CI. A GLM for the AUC for the LDA calculated for the beta tPRP yielded significant differences for proficient vs. naïve or shuffled (*P* < 0.001), and for high S+ vs. low S+ (*p* < 0.05, 88 observations, 76 d.f., F-statistic = 34.3, *p*-value for F-statistic < 0.001, *n* = 8 mice), but did not yield a statistical significance between peak and trough (*p* > 0.05). A GLM for AUC for the LDA calculated for the gamma tPRP yielded significant differences for proficient vs. naïve or shuffled and peak vs. trough (*p* < 0.001, 88 observations, 76 d.f., F-statistic = 29.2, *p*-value for F-statistic < 0.001), but not for and high S+ vs. low S+ (*p* > 0.05). Asterisks denote significant *post-hoc* statistical difference assessed with either *t*-test or ranksum tests corrected for multiple comparisons, pFDR = 0.04 (Ci), 0.036 (Cii), 0.03 (Di), and 0.04 (Dii). **(E,F)** Within trial time course for the *p*-value of a ranksum test evaluating the statistical difference between rewarded vs. unrewarded stimulus trials calculated using licks (black line) or with the prediction generated by LDA analysis with peak tPRP (red line) or trough tPRP (blue line). The *p*-values are shown for the sessions where the rewarded stimuli were the high concentration odorants. The horizontal red line denotes *p* = 0.05. **(A)** Beta tPRP. **(B)** Gamma tPRP. **(i)** Naïve. **(ii)** Proficient. The shadow denotes the bootstrapped 95% CI, *n* = 8 mice. **(G,H)** Decision time calculated when the *p*-value curves drop below *p* = 0.05. **(A)** Decision time for beta tPRP. **(B)** Decision time for gamma tPRP. **(i)** Naïve. **(ii)** Proficient. Light blue is the decision time for licks, green denotes decision time for naïve animals and magenta denotes decision time for proficient animals. The shadow denotes the bootstrapped 95% CI, *n* = 8 mice. A GLM for the decision times for the LDA calculated for the beta tPRP yielded significant differences for proficient vs. naïve and for the interaction between proficient vs. naïve and for high S+ vs. low S+ (*P* < 0.001, 87 observations, 75 d.f., F-statistic = 26.6, *p*-value for F-statistic < 0.001, *n* = 8 mice), but did not yield a statistical significance between peak and trough (*p* > 0.05). A GLM the decision times for the LDA calculated for the gamma tPRP yielded significant differences for proficient vs. naïve (*p* < 0.001, 87 observations, 75 d.f., F-statistic = 19.6, *p*-value for F-statistic < 0.001). Asterisks denote significant *post-hoc* statistical difference assessed with either *t* test or ranksum tests corrected for multiple comparisons, pFDR = 0.03 (Gi), 0.036 (Gii), 0.03 (Hi), and 0.034 (Hii).

If changes in tPRP are strongly related to learning, a simple classifier should be able to accurately discriminate between rewarded and unrewarded conditions. To test this, we used linear discriminant analysis (LDA). LDA places a hyperplane in *n* dimensional space to separate different categories. We trained the LDA algorithm to differentiate between rewarded and unrewarded stimuli using the tPRP recorded by the 16 electrodes in all trials minus one, and then asked whether the trial that was left out belonged to the reinforced stimulus group. As a control, the algorithm was trained after shuffling the identity of reinforced stimulus for each trial. Figures 6A,B show the time course for the performance of the LDA decoder for beta and gamma tPRP (*n* = 7 mice). The performance diverged strongly from the shuffled control shortly after odorant addition for proficient animals and reached levels above 70% by the end of odorant presentation (Figures 6Aii,Bii). In contrast, LDA performance did not increase appreciably during odorant exposure when the mice were naïve (Figures 6Ai,Bi). Decoding performance was higher for peak tPRP for the gamma tPRP, but not for the beta tPRP (compare Figures 6Aii,Bii). We quantified the performance of the decoder by calculating the area under the performance curve (AUC) normalized such that when the performance remains at 50% the AUC is zero and when the performance increases to 100% when the odorant is added the AUC is one. AUC values are shown for all conditions in Figure 6C (beta) and Figure 6D (gamma). AUC LDA performance for beta tPRP was significantly different (Figure 6C) for proficient vs. naïve or shuffled (*P* < 0.001) and for high S+ vs. low S+ (GLM, *p* < 0.05, 88 observations, 76 d.f., F-statistic = 34.3, *p*-value for F-statistic < 0.001, *n* = 8 mice, Supplementary Table 10). Peak and trough differences were not significantly different (GLM, *p* > 0.05). AUC for the LDA calculated for the gamma tPRP (Figure 6D) yielded significant differences for proficient vs. naïve or shuffled and peak vs. trough (GLM, *p* < 0.001, 88 observations, 76 d.f., F-statistic = 29.2, *p*-value for F-statistic < 0.001, Supplementary Table 11), but not for and high S+ vs. low S+ (GLM, *p* > 0.05).

We complemented the analysis of the performance of the LDA decoder by asking whether the decision time differs between the behavior (differential licking for the rewarded vs. unrewarded odorants) and the decoder (differential classification of the stimulus as rewarded vs. unrewarded by the LDA algorithm). As in previous studies we assessed the difference in licks or decoder classification by calculating the *p*-value for the difference between reinforced stimulus trials and unreinforced stimulus trials with a ranksum test (Losacco et al., 2020). Figures 6E,F show the time course for this *p*-value calculated for licks (black), peak tPRP (red), and trough tPRP (blue) when the reinforced stimulus was the high concentration range. As expected, the *p*-value for licks dropped quickly below 0.05 after addition of the odorant when the mice were proficient (Figure 6Eii or Figure 6Fii, black line). In contrast, when mice were naïve the *p*-value for licks dropped slowly after odorant addition and dropped more sharply after the mice were reinforced with water (Figure 6Ei or Figure 6Fi, black line). For beta tPRP decoding both the peak and trough tPRP *p*-values displayed a similar sharp drop below *p* = 0.05 after odorant addition when the animal was proficient (Figure 6Eii, red and blue lines), while the drop below 0.05 was more pronounced for the *p*-values calculated with the peak gamma tPRP (Figure 6Fii, red and blue lines).

The decision time was estimated as the time when the *p*-value for the ranksum test dropped below 0.05. The *p*-value was calculated with a ranksum test for licks (scored 1 for lick and 0 for no lick) for all trials for each time point. This allows for a reliable determination of decision time when the *p*-value falls monotonically below 0.05 (Doucette and Restrepo, 2008). We found that decision times for both licks and peak or trough tPRP decoding became shorter when the mice became proficient. A GLM for the decision times for the LDA calculated for the beta tPRP yielded significant differences for proficient vs. naïve and for the interaction between proficient and naïve and for high S+ vs. low S+ (Figure 6G, *P* < 0.001, 87 observations, 75 d.f., F-statistic = 26.6, *p*-value for F-statistic < 0.001, *n* = 8 mice, Supplementary Table 12), but did not yield a statistical significance between peak and trough (*p* > 0.05). When comparing decision times for the LDA calculated for the gamma tPRP were significantly different for proficient vs. naïve conditions (GLM, *p* < 0.001, 87 observations, 75 d.f., F-statistic = 19.6, *p*-value for F-statistic < 0.001, Supplementary Table 13). Interestingly, for proficient mice, *post-hoc t* or ranksum tests indicated that there was a significant difference for decision times calculated for tPRP decoding compared to lick decision times when the reinforced stimulus was the low concentration range, but not when the reinforced stimulus was the high concentration range Figures 6G,H. Thus, the speed of decision making differs when the rewarded stimulus is the low or high concentration range.

### Behavioral Performance Correlated With Performance for Decoding the Stimulus From Theta Phase-Referenced LFP Oscillation Power

**Figure 7:**
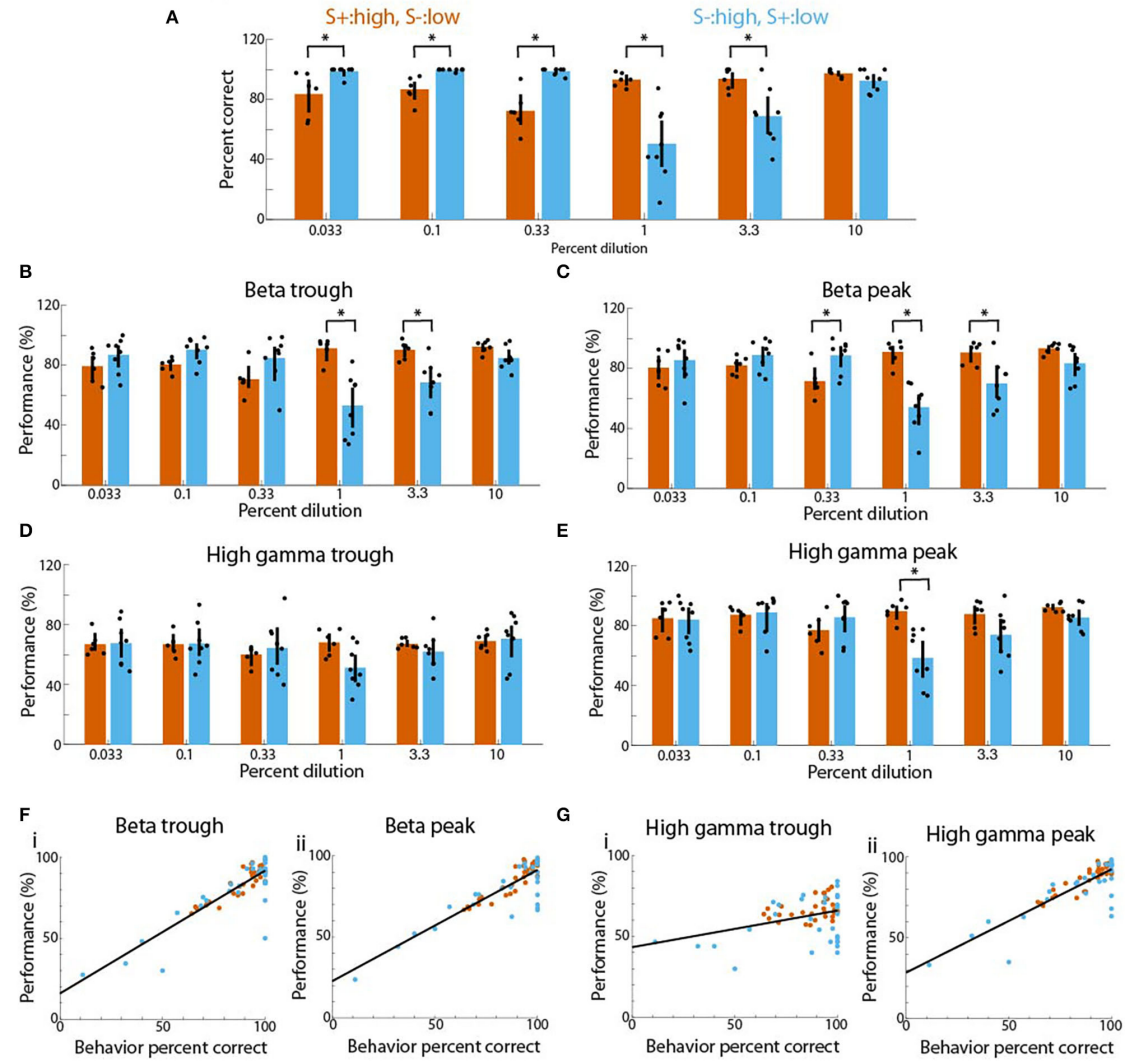
Linear relationship between tPRP decoding performance and behavioral percent correct for mice proficient in the go-no go concentration task. **(A)** Behavioral percent correct as a function of concentration shown for sessions where the high concentration range was rewarded (vermillon) and sessions where the low concentration range was rewarded (sky blue). A GLM analysis indicates that there was a significant difference for the interaction between reward for high vs. low concentration and odorant dilution (*p* < 0.001, 84 observations, 80 d.f., F-statistic 4.98, *p* < 0.01, *n* = 6 mice). Asterisks denote significant differences between the two sessions differing in rewarded stimulus tested *post-hoc* with either t or ranksum tests corrected for multiple comparisons (*p* < pFDA = 0.023). B and C. Performance for decoding the rewarded stimulus from the tPRP for beta oscillations. **(B)** shows results for trough-referenced tPRP and **(C)** shows decoding for peak-referenced tPRP. For the trough-referenced tPRP a GLM analysis indicated that there was a significant difference for reward for high vs. low concentration (*p* < 0.05) and odorant dilution (*p* < 0.05) for the interaction between reward for high vs. low concentration and odorant dilution (*p* < 0.01, 84 observations, 80 d.f., F-statistic 4.7, *p* < 0.01, *n* = 6 mice). Asterisks denote significant differences between the two sessions differing in rewarded stimulus tested *post-hoc* with either t or ranksum tests corrected for multiple comparisons (*p* < pFDA = 0.017). For the peak-referenced tPRP a GLM analysis indicated that there was a significant difference for reward for high vs. low concentration (*p* < 0.01) and odorant dilution (*p* < 0.05) for the interaction between reward for high vs. low concentration and odorant dilution (*p* < 0.01, 84 observations, 80 d.f., F-statistic 4.7, *p* < 0.01, *n* = 6 mice). Asterisks denote significant differences between the two sessions differing in rewarded stimulus tested *post-hoc* with either t or ranksum tests corrected for multiple comparisons (*p* < pFDA = 0.016). **(D,E)** Performance for decoding the rewarded stimulus from the tPRP for gamma oscillations. **(D)** shows results for trough-referenced tPRP and E shows decoding for peak-referenced tPRP. For the trough-referenced tPRP a GLM analysis indicated that there were no statistical differences (*p* > 0.05, 84 observations, 80 d.f., F-statistic 0.37, *p* > 0.05, *n* = 6 mice) and there were no significant differences in *post-hoc t* or ranksum tests corrected for multiple comparisons (*p* < pFDA = 0.0004). For the peak-referenced tPRP a glm analysis indicated that there was a significant difference for reward for high vs. low concentration (*p* < 0.01, 84 observations, 80 d.f., F-statistic 2.8, *p* < 0.05, *n* = 6 mice). The asterisk denotes a significant difference between the two sessions differing in rewarded stimulus tested *post-hoc* with a *t*-test corrected for multiple comparisons (*p* < pFDA = 0.002). **(F,G)** Relationship between tPRP stimulus decoding performance and percent correct for the percent correct responses in the go-no go concentration task plotted for all concentrations for sessions where the high concentration range was rewarded (vermillon) and sessions where the low concentration range was rewarded (sky blue) (only mice that were tested in both types of sessions were included, *n* = 5 mice). **(F)** Performance of beta tPRP. **(G)** Performance of gamma tPRP. **(i)** trough-referenced tPRP, **(ii)** peak referenced tPRP. Lines are linear best fits. A linear correlation analysis yielded the following correlation coefficients (rho) and *p*-values: Fi. rho 0.86, *p* < 0.001, Fii. rho 0.85, *p* < 0.001, Gi. rho 0.36, *p* < 0.01, Gii. rho 0.84, *p* < 0.001.

The differences in tPRP and decoding performance between sessions where the rewarded stimulus were the high vs. the low concentration odorants (**Figures 4–6**) raised the question whether the responses of the animals to the different odorant concentrations also differed. When we plotted the percent correct responses in the go-no go task as a function of odorant concentration we found that when the high concentration range was rewarded there were small differences in behavioral performance between the high and low odorants (vermillion bars in Figure 7A). In contrast, when the low concentration odorants were rewarded, the performance was high for the low concentration odorants, and changed as a function of concentration for the high concentration range with the lowest performance being the 1% c_*liq*_ (sky blue bars in Figure 7A). There was a significant difference for the interaction between reward for concentration and odorant dilution (GLM, *p* < 0.001, 84 observations, 80 d.f., F-statistic 4.98, *p* < 0.01, *n* = 6 mice, Supplementary Table 14). Interestingly, when we plotted the performance for decoding the stimulus from tPRP averaged between 2 and 2.5 s after odorant addition we found the same trend for theta-referenced beta and gamma LFP power (Figure 7B through Figure 7E). Thus, when the high concentration odorants were rewarded there were small differences in performance (vermillion bars in Figure 7B through Figure 7E) while when the low concentration odorants were rewarded there was a decreased performance for the 1 and 3.3% c_*liq*_ (sky blue bars in Figure 7B through Figure 7E). For the trough-referenced beta tPRP (Figure 7B) there was a significant difference for reward for high vs. low concentration (GLM, *p* < 0.05) and odorant dilution (GLM, *p* < 0.05) and for the interaction between reward for high vs. low concentration and odorant dilution (GLM, *p* < 0.01, 84 observations, 80 d.f., F-statistic 4.7, *p* < 0.01, *n* = 6 mice, Supplementary Table 15). For the peak-referenced beta tPRP (Figure 7C) there was a significant difference for reward for high vs. low concentration (GLM, *p* < 0.01) and odorant dilution (GLM, *p* < 0.05) and for the interaction between reward for high vs. low concentration and odorant dilution (GLM, *p* < 0.01, 84 observations, 80 d.f., F-statistic 4.7, *p* < 0.01, *n* = 6 mice, Supplementary Table 16).

Trough-referenced gamma tPRP had no statistical differences (GLM, *p* > 0.05, 84 observations, 80 d.f., F-statistic 0.37, *p* > 0.05, *n* = 6 mice, Supplementary Table 17) and there were no significant differences in *post-hoc t* or ranksum tests corrected for multiple comparisons (*p* < pFDA = 0.0004). For the peak-referenced tPRP there was a significant difference for reward for high vs. low concentration (GLM, *p* < 0.01, 84 observations, 80 d.f., F-statistic 2.8, *p* < 0.05, *n* = 6 mice, Supplementary Table 18). Finally, we found a linear relationship between decoding performance and behavioral performance (Figures 7F,G) indicating that tPRP signal processing in the OB may contribute to behavioral performance in the go-no go odorant concentration task.

### A Model of Odor Concentration Discrimination

**Figure 8:**
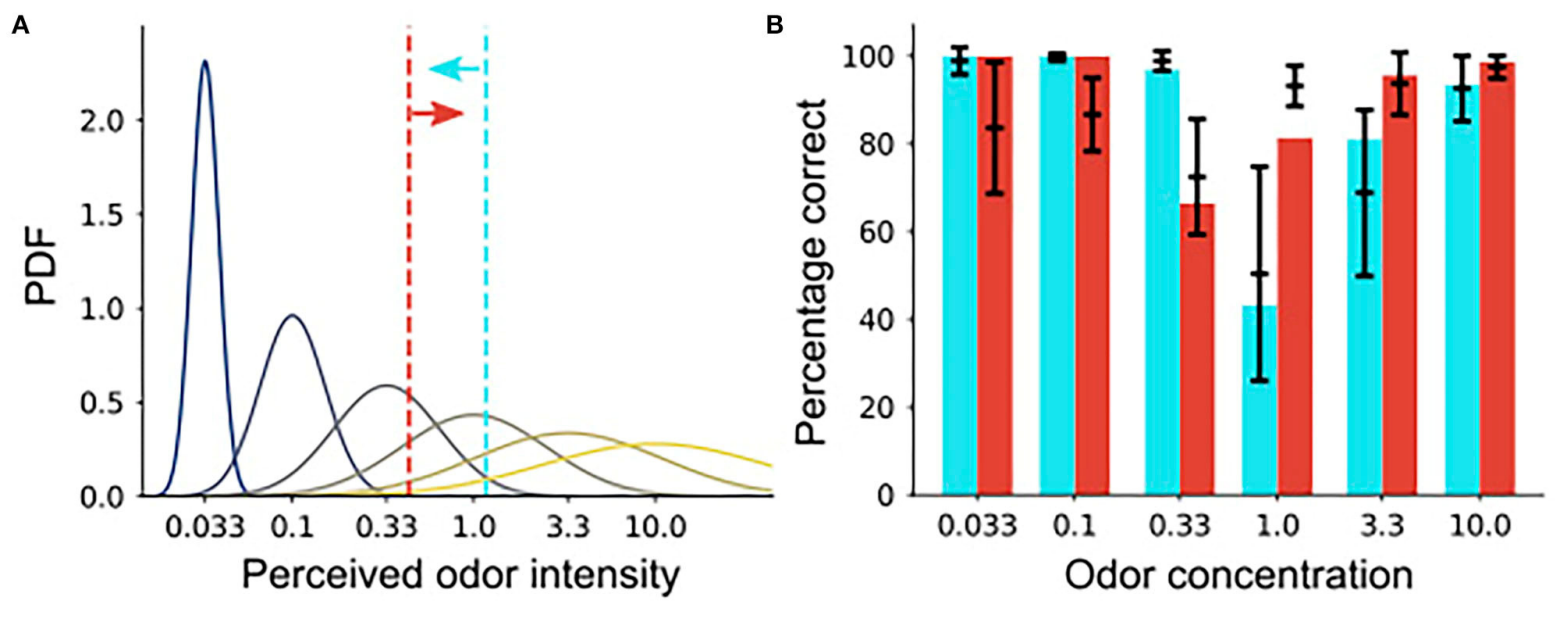
A signal classification model of the animal behavior. **(A)** A schematic of the perceived odor intensity distributions. The curves represent the probabilistic distribution of the perceived odor intensity in the model under each odor concentration. The x-axis is labeled with the corresponding odor concentrations, and the dotted vertical lines are boundaries for go decision in the two task conditions. **(B)** Fitting of the animal behavior. Black bars represent the range of animal behavior taken from Figure 7A (the means ± the standard deviations), while cyan and vermilion bars are predictions from the model.

We found an asymmetry in the false alarm rates between the high and low concentration rewarding conditions, which is reflected in the OB activity (Figure 7). To gain insights on the underlying mechanism, we constructed a simple model of the concentration discrimination task. In the model, the perceived intensity follows a Gaussian distribution centered around the true intensity (curves in Figure 8A). In the S+: high condition, for instance, the model makes a go decision if the perceived intensity is larger than the boundary shown in vermilion line, or vice versa (cyan line). Here, we used different boundaries for high/low rewarded conditions, and the variance in the perceived intensity was set to scale with the mean intesntiy (blue to yellow curves).

The model reproduced the behavioral result well-upon parameter fitting (Figure 8B). In particular, it qualitatively reproduced the asymmetry between the correct rejection rates in the two task conditions, though it overestimated the correct rejection rate under a very low odor concentration. The model supports the hypothesis that animals use a single boundary for concentration discrimination task, but it also suggests that, in go/no-go tasks, the boundary should be different depending on whether high or low concentrations are rewarded.

## Discussion

We studied changes in odorant signal processing in the OB of awake behaving mice learning to discriminate low and high concentration odorants in a go-no go task. We find that as the animal learns to differentiate between concentrations the tPRP of beta and gamma LFP oscillations tends to increase for reinforced odorant concentrations and decrease for unreinforced concentrations (Figure 4). These changes in tPRP are similar for peak- and trough-referenced power for the beta frequency, and are larger for peak-referenced gamma power compared to trough-referenced tPRP. The reinforced stimulus was successfully decoded from beta and gamma tPRP using a linear discriminant analysis (Figures 6, 7). Interestingly, we found that, while decoding precision is similar for all concentrations when the high odorants are rewarded, precision changes as a function of concentration for the high concentration range when the low concentration is rewarded (Figures 7B–E) and percent correct animal behavior follows a similar pattern (Figure 7A). Furthermore, we find a linear relationship between percent correct for animal behavior and decoding precision of stimulus reward from tPRP (Figures 7F,G). These findings suggest that as the animal learns to discriminate concentrations in the go-no go task oscillatory signal processing in the OB is altered to facilitate categorical classification of odorant concentration stimuli into low and high concentration ranges.

When awake or anesthetized animals are passively exposed to odorants, information on stimulus concentration is encoded by temporal patterns of mitral/tufted (M/T) cell activity (Chalansonnet and Chaput, 1998; Cang and Isaacson, 2003; Margrie and Schaefer, 2003; Stopfer et al., 2003; Sirotin et al., 2015). Here, we studied theta phase-referenced LFP power that reflects synchronized temporal patterns of activity of M/T cells. Our study found that when the animal becomes proficient classifying the stimuli within low/high concentration ranges the tPRP diverged between the two groups of stimuli (Figure 4). In the proficient animals there was a tendency for an increase in tPRP for the rewarded concentration odorants and a decrease for unrewarded odorant concentrations. Furthermore, we found that performance for decoding of the stimulus from tPRP increases as the animal becomes proficient (Figures 6A–D). Therefore, information carried by the OB tPRP on which stimulus is rewarded increased as the animal learned to differentiate concentration ranges.

Interestinlgy, when we compared behavioral performance for proficient mice between sessions rewarded for high vs. low concentrations we noticed that the number of FAs increased when the rewarded stimulus was the low odorant group (Figure 7A). The increased FAs could be due to the effect of stimulus strength on behavioral responsiveness (Macmillan et al., 1973; Sun and Landy, 2016). The increased FA in the low concentration condition increased the variance of percent correct performance revealing a substantial correlation between decoding the reinforced stimulus from tPRP and behavioral performance for the proficient animal (Figures 7F,G). Behavioral studies of mice making two alternative forced choice decisions to classify stimuli as low or high odorant concentration found that these decisions are made based on a scale of intensity with a single intensity criterion (Wojcik and Sirotin, 2014). We found that when we assumed that the animal decides whether the concentration is high or low using a fixed threshold θ (Wojcik and Sirotin, 2014), a model motivated by the statistics of OB activity (Pillow and Scott, 2012; Bolding and Franks, 2018) displays a concentration range discrimination behavior comparable to the behavior of the mice and decoding stimulus choice with tPRP (compare Figures 7A–D, 8). Our findings suggest that signal processing in the OB undergoes plasticity such that the information transferred to higher order centers such as piriform cortex is filtered to facilitate discrimination of odorant intensity according to the single intensity perceptual criterion. Finally, we did not find differences in the results for the two odorants used in this study, and we merge the results for the two odorants for the analysis presented here. Future studies are necessary to assess whether using different odorants or wider concentration ranges may alter the results.

The fact that the information on odorant concentration is encoded by the temporal pattern of activity of M/T cells and that there is large variance in sniff rates in the awake behaving animal raises the question of whether perception of odorant intensity is dependent on sniff rate. Two recent studies addressed this question. Shusterman and colleagues postulate that nose fluid dynamics contributes to sniff-invariant odor intensity coding (Shusterman et al., 2018). Furthermore, Jordan et al., propose that a possible reason for sniff modulation of the early olfactory system may be to directly inform downstream centers of nasal flow dynamics, so that an inference can be made about environmental concentration independent of sniff variance (Jordan et al., 2018b). These investigators raised the question of whether it is possible that in a mouse performing a concentration guided task, even the OB circuit could be altered by top-down circuits in such a way as to generate a sniff-invariant representation of concentration. The data presented in this study indicates that learning does alter encoding of odorant concentration ranges by theta-phase referenced beta and gamma oscillations in the OB (Figure 6). This likely is due to alteration of OB processing by top-down circuits (Gire et al., 2013). Furthermore, since transmission of information from the OB is filtered by piriform cortex feedback inhibition circuits (Bolding and Franks, 2018) and because beta OB oscillations depend on centrifugal OB input (Kay, 2015), it is likely that learning involves coordinated plasticity in the piriform cortex. This would involve piriform circuitry where cortical interneurons sharpen the latency shifts evoked by concentration change and encode concentration via the synchronicity of ensemble firing involved in perception of odorant intensity (Bolding and Franks, 2017).

Prediction of the speed of decision making through LDA analysis of tPRP yields decision making times that do not differ from the decision time calculated by a ranksum test of licks when the rewarded stimulus is the high concentration range (Figures 6G,H). However, prediction speed differs between tPRP LDA and licks when the rewarded stimulus is the low concentration range. This could be due to lack of information necessary to predict lick decision time in the tPRP. It is possible that in order to predict decision making time for the low concentration range reward condition it is necessary to consider other neural factors such as within sniff neural activity. However, the failure to predict decision making time could also be due to non-linear separation of the high dimensional data, and other decoding algorithms such as artificial neural networks, or nearest neghbor may provide better information on decision making.

Our study raises the question of which mechanisms mediate the changes elicited by learning in the tPRP beta and gamma power. Some of these changes could be due to alteration in olfactory input to the glomeruli. Indeed, odor fear conditioning elicits changes in the glomerular olfactory input for different odorant concentrations (Kass et al., 2013). However, the data in Figure 2 shows that there are significant differences for the strength of phase-amplitude coupling and for the variance of the peak angle of beta and gamma bursts between the rewarded and unrewarded stimuli in proficient mice. These changes in OB phase-amplitude coupling would be due to changes in subthreshold oscillations of the M/T cells that could be elicited, for example, by altered M/T-granule cell coupling that is known to increase pattern separation for odorant discrimination (Nunez-Parra et al., 2013; Gschwend et al., 2015).

In conclusion, we find that learning to differentiate subsets of odorants differing by odorant concentration in a go-no go task increases information conveyed by theta-phase referenced neural beta and gamma oscillations of the OB to categorize odorants as low or high intensity. Our finding suggests that OB oscillatory events facilitate decision making in downstream circuits to classify concentrations using a single intensity criterion.

## Materials and Methods

### Animals

The study was performed with male 6–11 months old OMP-hChR2V mice (Li et al., 2014) (eight mice) and one C57BL/6 mouse (Jackson stock number: 000664). All animal procedures were performed under approval from the Institutional Animal Care and Use Committee (IACUC) at the University of Colorado Anschutz Medical Campus under guidelines from the National Institutes of Health.

### Tetrode Implantation

Surgery was performed under approval from the Institutional Animal Care and Use Committee (IACUC) at the University of Colorado Anschutz Medical Campus, using aseptic technique. As per Li et al. (2015), tetrode boards (EIB-16, Neuralynx) were populated with four tetrodes consisting of four 12.5 μm nichrome wires coated with polyimide (Sandvik RO800). Electrode tips were electroplated to 0.2–0.4 MΩ impedance.

Two-months-old male mice were anesthetized with 5% isoflurane in oxygen. Intraperitoneal ketamine/xylazine (100 and 10 mg/kg, respectively) was then administered along with 100 μl of 2% lidocaine injected subcutaneously over the skull. After the mouse was found to be unresponsive to a toe pinch, the animal's head was then secured in the stereotaxic apparatus (Narishige SR-5M-HT) and the skull was leveled (≤50 μm difference DV between bregma and lambda). Gentamycin ophthalmic ointment was applied to the eyes to maintain hydration. After incising the skin overlaying the skull, the periosteum was cleared with 15% H_2_O_2_. A manipulator (Sutter MP-285) was zeroed at bregma and midline and the target location for OB implantation was marked with respect to bregma (AP +4.28 mm, ML +0.5 mm).

A craniotomy performed at this site (Marathon III drill) exposed dura mater which was removed prior to implantation. Another craniotomy was performed more caudally for implantation of one ground screw (Plastics1 00-96 × 1/16). The tetrode was positioned above the craniotomy over the OB while the ground wire was wrapped around the ground screw with the connection coated in silver paint (SPI Flash-Dry silver conductive paint). After securing the ground screw to the skull, the tetrodes were lowered into position at the rate of 1 mm/minute (AP +4.28 mm, ML +0.5 mm, and DV 1.0 mm). After reaching the target depth, the tetrode was adhered to the skull with C&B Metabond, followed by Teets “Cold Cure” dental cement. After curing (10 min), the tetrode was detached from the manipulator, the animal was removed from the stereotax and received subcutaneous injections of carprofen (10 mg/kg) and buprenorphine (0.05 mg/kg) and recovered on a heating pad kept at 37°C. The mice were monitored daily and received additional carprofen injections daily for the first 2 days postoperatively.

### Go-No Go Concentration Behavioral Task

Mice were water deprived until they reached 80% normal body mass. Then they were placed into the Slotnick olfactometer (Bodyak and Slotnick, 1999; Li et al., 2015) chamber where they could move freely. All mice were first trained to lick the water spout to obtain water in the presence of odor (1% isoamyl acetate in mineral oil, v/v) in the “begin” task (Slotnick and Restrepo, 2005). Training in the begin task required 2–5 sessions of 50–200 trials. Each session was run until the animal became satiated. Subsequently they learned to discriminate 1% isoamyl acetate (S+) vs. mineral oil (S-) in the “go no-go” task (Doucette et al., 2011; Li et al., 2015), followed by learning to discriminate high from low odorant concentrations. For the go-no go odorant concentration discrimination task one of six odorant concentrations was presented randomly in each trial. Odorized air was generated by bubbling air at 50 ml/min through mineral oil with either isoamyl acetate or acetophenone diluted in six logarithmically-spaced v/v liquid dilution (c_*liq*_): 10, 3.3, 1, 0.33, 0.1, and 0.033% (Wojcik and Sirotin, 2014). The high odorant concentration range was 10, 3.3, and 1% c_*liq*_ and the low odorant concentration range was 0.33, 0.1, and 0.033% c_*liq*_.

The go-no go task was performed as described by Losacco et al. (2020) with the exception that the rewarded stimulus was either the high or low concentration odorants. Mice self-initiated trials by poking their head into the odor delivery port, breaking a photodiode beam (Figure 1A). During reinforced odorant delivery (lasting 2.5 s) they must lick a water delivery spout at least once during each of four 0.5 s-long response areas in order to register the decision as a Hit (Figure 1B). Licks were detected as electrical connectivity between the water spout and the ground plate on which they stand (Slotnick and Restrepo, 2005). If the mice licked during a rewarded odorant trial, they received ~10 μl water reinforcement. The mice learn to refrain from licking for the unrewarded odorant due to the unrewarded effort of sustained licking. This task was not designed to require the mouse to respond as soon as possible. The proficient mouse starts licking at the beginning of the trial and for correct rejections the last lick that takes place 0.3–0.7 s after odorant onset (Figures 6E,F). Rather, this task was designed to make the mouse aware that the two stimuli have different valences, and allow the mouse to take it's time to make the decision.

Mice were presented blocks of 20 trials, with 10 S+ and 10 S- trials presented at random. Animals performed as many as 10 blocks per session. Sessions were terminated when animals demonstrated satiety/disengagement from the task or when they performed at or above 80% correct discrimination in three or more blocks in a session. Supplementary Figure 1 shows which of the two odorants (isoamyl acetate or acetophenone) and which odorant group (high vs. low concentrations) was the reinforced stimulus for each go-no go session for each of the nine mice. Data were analyzed for all odorant sessions. Data were analyzed within two performance windows: when the animal was performing below 65% (naïve) or above 80% (proficient). The results obtained with the two odorants were similar and the results from both odorants were merged for data analysis.

Odor stimulus delivery time was measured with a photoionization detector (miniPID, Aurora Scientific). Figure 1, Supplementary Figure 1 of Losacco et al. (2020) shows the time course for odorant concentration measured at the odor spout. The time difference between valve opening and detection of odor at the odor port was between 66 and 133 ms, depending on which olfactometer was used. The minimum intertrial interval in all the sessions for this study was 13.5 s. Using an multi exponential fit of the time course for odorant concentration measured with the PID we estimate that the largest residual odorant is equivalent to a 0.00008% c_*liq*_, two orders of magnitude smaller than the smallest c_*liq*_ used in the concentration series indicating that the olfactometer does not compromise concentration delivery. Finally, after valve opening the odorant raised sharply (0.12 s half time) and then increased more slowly for the rest of the odorant application interval (2.16 s half time) (Figure 1, Supplementary Figure 1 in Losacco et al. (2020). As a result, the mouse is making a decision on the dynamic change in concentration as opposed to a step change in concentration.

### Neural Recording

Extracellular potentials from the four tetrodes were captured and digitized at 20 kHz on the RHD2216 amplifier of the Intan RHD2000 Evaluation System with a 1–750 Hz bandpass filter. Information on behavioral events (valve times, mouse presence at the odor port) was sent through a digital line from the Slotnick olfactometer to the Intan board. Licks detected by the Slotnick olfactometer were recorded as an analog signal by the Intan board and were digitized at 20 kHz.

### Phase Amplitude Coupling

PAC data were processed using the Hilbert transform method described by Tort et al. (2010). Briefly, data were bandpass filtered with a 20th order Butterworth filter using Matlab's filtfilt function with zero phase shift to extract LFP in the low frequency oscillation used for phase estimation (theta, 2–14 Hz, Figures 1Eiii,Fiii) and the high frequency oscillation used for estimation of the amplitude of the envelope (either beta, 15–30 Hz, or gamma, 65–95 Hz, Figures 1E,F). Hilbert transform established the theta phase (Figures 1Eiii,Fiii) and, separately, the envelope for beta or gamma (red line in Figures 1Eiv,Fiv). To quantify the strength of tPAC we calculated the modulation index (MI) estimating the KL distance to quantify the difference between the observed beta/gamma amplitude distribution along the phase of theta from a uniform distribution. If tPAC is non-existent, MI = 0, meaning the mean amplitude is distributed uniformly over theta phases, and if tPAC is a delta function MI = 1. MI for signals measured in brain areas such as the hippocampus typically fall between 0 and 0.03 (Tort et al., 2010).

### Theta Phase-Referenced LFP Power

tPRP was calculated as detailed in Losacco et al. (2020) using custom Matlab code. Briefly, tPAC was calculated using the approach documented by Tort et al. (2010), as described above and summarized in Figure 1. Peak and trough theta phases are defined as the phase for maxima and minima of the tPAC distribution measured for the S+ trials. A continuous Morlet wavelet transform was used to estimate the power for the high frequency oscillations (Buonviso et al., 2003). tPRP was estimated as the power of the high frequency oscillations (beta or gamma) measured at the peak or trough of tPAC. The Matlab code used for data analysis has been deposited to https://github.com/restrepd/drgMaster.

### Statistical Analysis

Statistical analysis was performed in Matlab. tPAC parameters and tPRP were calculated separately per electrode (16 electrodes per mouse) for all electrodes per mouse. Statistical significance for changes in measured parameters for factors such as learning and odorant identity was estimated using generalized linear model (GLM) analysis, with *post-hoc* tests for all data pairs corrected for multiple comparisons using false discovery rate (Curran-Everett, 2000). The *post hoc* comparisons between pairs of data were performed either with a *t*-test, or a ranksum test, depending on the result of an Anderson-Darling test of normality. GLM is a general statistical method that includes regression and analysis of variance. Degrees of freedom and statistical significance have the same meaning in GLM as in analysis of variance and regression (Agresti, 2015). In addition, as a complementary assessment of significant differences (Halsey et al., 2015) we display 95% confidence intervals (CIs) shown in the figures as vertical black lines were estimated by bootstrap analysis of the mean by sampling with replacement 1,000 times.

### Linear Discriminant Analysis

Classification of trials using tPRP was accomplished via LDA in Matlab whereby tPRP for every trial except one were used to train the LDA, and the missing trial was classified by the LDA prediction. LDA, lick ranksum differences and lick rate were computed in 0.1 s bins. This was repeated for all trials and was performed separately for peak and trough tPRP, and for analysis where the identity of the odorants was shuffled. LDA and dimensionality analysis were performed either on a per-mouse basis where the input was the tPRP recorded from 16 electrodes, or on pooled mouse data where the input was the tPRP recorded from 16 × N electrodes where *N* is the number of mice. For pooled mouse analysis a pooled response vector was therefore created by concatenating across animals and the number of trials *n*, was determined by the session with the lowest number of trials for a single odorant (Chu et al., 2016). LDA decoding was not calculated for mice that did not have more than 20 trials for both naïve and proficient mice.

### Behavioral Modeling

For a given concentration *c*_*k*_∈{0.033, 0.1, 0.33, 1.0, 3.3, 10}, we modeled the perceived intensity by a random Gaussian variable *x* ~ *N*(μ_*k*_, σ_*k*_^2^), where the mean and the variance are given as μ_*k*_ = log(*c*_*k*_/*c*_min_) and σ_*k*_^2^ = *a*_1_μ_*k*_ + *a*_2_μ_*k*_^2^, This is because the OB activity roughly scales logarithmically with the concentration (Bolding and Franks, 2018), and its variance scales linearly with the firing rate if the activity is Poisson, and potentially more steeply if bursty (Pillow and Scott, 2012). Suppose an animal decides whether the concentration is high or low using a fixed threshold θ (Wojcik and Sirotin, 2014), the probability that the animal judges the concentration as low is given by a Gaussian cumulative distribution function Φ[(θ - μ_*k*_)/σ_*k*_].

We fitted this model to the behavioral data, by minimizing the mean squared error between the estimated performance from the model and the mean performance over all the mice. Minimization was performed with gradient descent from various different initial states, which robustly converged onto *c*_min_ = 0.015, *a*_1_ = 0, *a*_2_ = 0.0478, and the thresholds for the low and the high tasks were converged to θ_low_ = 4.357 and θ_high_ = 3.378, respectively. The simulation code is deposited at https://github.com/nhiratani/odor_concentration_classification.

## Data Availability Statement

The raw data supporting the conclusions of this article will be made available by the authors, without undue reservation.

## Ethics Statement

The animal study was reviewed and approved by Institutional Animal Care and Use Committee University of Colorado Anschutz Medical Campus.

## Author Contributions

JL and DR designed the experiments and performed the data analysis. JL performed the *in vivo* experiments. NH performed the behavioral modeling. All authors wrote and edited the manuscript.

## Conflict of Interest

The authors declare that the research was conducted in the absence of any commercial or financial relationships that could be construed as a potential conflict of interest.

## Supplementary Material

Supplementary Figure 1 Percent correct for the go-no go concentration task shown as a function of trial number for all sessions performed by the nine animals included in this study. Sessions are shown from first to last. The number of days between sessions is 1–5 days. The odorant group that was rewarded is shown above the plot for each session (high: 10, 3.3, and 1% c_*liq*_, low: 0.33, 0.1, and 0.033% c_*liq*_).

Supplementary Figure 2 Phase amplitude coupling for the go-no go odorant concentration task for a session where the mouse was naïve and the rewarded stimuli were high concentration odorants. **(A–D)** tPAC analysis shown for a go-no go session where the mouse was naïve in differentiating between the high concentration odorants (c_*liq*_ 1–10%, rewarded stimulus, S+) and the low concentration odorants (c_*liq*_ 0.033–0.33%, unrewarded stimulus, S-). **(A)** Pseudocolor image showing the per-trial average amplitude for the theta envelope for the gamma LFP. The odorant used was isoamyl acetate. Odorant dilutions (c_*liq*_) for S+ were 10, 3.33, and 1% and for S- were 0.33, 0.1, and 0.033%. **(B)** Percent correct as a function of trial number. Light gray: percent correct >65% and <80%, blue: percent correct ≤ 65%. **(C)** Strength of tPAC quantified as the modulation index (MI) displayed for the six odorant dilutions. A GLM analysis indicated that there is no difference between S+ and S- (*p* > 0.05, 134 trials, 131 d.f., F-Statistic 6.33, *p*-value for the model < 0.05, Supplementary Table 19). The asterisk denotes *post-hoc* differences evaluated with either *t*-test or ranksum, *p* < pFDR = 0.01. **(D)** Rose plot histograms for the peak phase angle for gamma tPAC shown in **(A)**.

Supplementary Figure 3 Phase amplitude coupling for the go-no go odorant concentration task for a session where the mouse was proficient and the rewarded stimuli were low concentration odorants. **(A–D)** tPAC analysis shown for a go-no go session where the mouse was proficient in differentiating between the high concentration odorants (c_*liq*_ 1–10%, rewarded stimulus, S-) and the low concentration odorants (c_*liq*_ 0.033–0.33%, unrewarded stimulus, S+). **(A)** Pseudocolor image showing the per-trial average amplitude for the theta envelope for the gamma LFP. The odorant used was isoamyl acetate. Odorant dilutions (c_*liq*_) for S- were 10, 3.33, and 1% and for S+ were 0.33, 0.1, and 0.033%. **(B)** Percent correct as a function of trial number. Light gray: percent correct >65% and <80%, magenta: percent correct ≥ 80%. **(C)** Strength of tPAC quantified as the modulation index (MI) displayed for the six odorant dilutions. A GLM analysis indicated that there is no difference between S+ and S- (*p* > 0.05, 70 trials, 67 d.f., F-Statistic 1.99, *p*-value for the model > 0.05, Supplementary Table 20). **(D)** Rose plot histograms for the peak phase angle for gamma tPAC shown in **(A)**.
