# Supplementary material for: The Olfactory Bulb Facilitates Use of Category Bounds for Classification of Odorants in Different Intensity Groups
Source: Front Cell Neurosci. 2020 Dec 11;14:613635. doi: 10.3389/fncel.2020.613635 (PMC7759615; doi:10.3389/fncel.2020.613635)
Supplement: Supplementary file 1 [file Table_1.pdf]

Table S1. Generalized linear model analysis for Figure 2C

MI: Modulation index
spm: S+ or S-
conc: log10(c_{liq})

Generalized linear regression model:
MI ~ 1 + spm + conc
Distribution = Normal

Estimated Coefficients:

	Estimate	SE	tStat	pValue
(Intercept)	0.023804	0.0014369	16.567	1.3137e-26
spm_1	-0.019114	0.0032797	-5.8281	1.3652e-07
conc	-0.0035439	0.0019129	-1.8526	0.067923

77 observations, 74 error degrees of freedom
Estimated Dispersion: 5.16e-05
F-statistic vs. constant model: 37.5, p-value = 5.65e-12

Ranksum or t-test p values

pFDR = 3.333333e-02

p value t-test for 0.32 vs 1 = 2.234311e-10
p value t-test for 0.1 vs 1 = 2.169374e-07
p value t-test for 0.32 vs 10 = 2.375760e-06
p value ranksum for 0.032 vs 1 = 2.769150e-05
p value t-test for 0.1 vs 10 = 1.684955e-04
p value t-test for 0.32 vs 3.2 = 8.868303e-04
p value ranksum for 0.032 vs 10 = 9.918563e-04
p value t-test for 1 vs 3.2 = 3.053955e-03
p value t-test for 0.1 vs 3.2 = 8.535633e-03
p value ranksum for 0.032 vs 3.2 = 9.464361e-03

p values below are > pFDR

p value t-test for 1 vs 10 = 1.144331e-01
p value t-test for 3.2 vs 10 = 1.622700e-01
p value ranksum for 0.032 vs 0.1 = 5.675953e-01
p value t-test for 0.1 vs 0.32 = 7.519570e-01
p value ranksum for 0.032 vs 0.32 = 7.659271e-01
