# Supplementary material for: The Olfactory Bulb Facilitates Use of Category Bounds for Classification of Odorants in Different Intensity Groups
Source: Front Cell Neurosci. 2020 Dec 11;14:613635. doi: 10.3389/fncel.2020.613635 (PMC7759615; doi:10.3389/fncel.2020.613635)
Supplement: Supplementary file 2 [file Table_2.pdf]

Table S2. Generalized linear regression model for Figure 2E, modulation index for beta tPAC.

mi: modulation index
group: S+: high vs. S+ low
perCorr: naïve vs. proficient
concentration: log10(c_{liq})

Generalized linear regression model:
mi ~ 1 + group*perCorr + group*concentration + perCorr*concentration + group:perCorr:concentration

Distribution = Normal

Estimated Coefficients:

	Estimate	SE	tStat	pValue
(Intercept)	0.0038242	0.00014642	26.118	1.2051e-134
group_2	0.0010082	0.00020049	5.0284	5.26e-07
perCorr_2	-0.0002531	0.00020707	1.2223	0.22169
concentration	0.00024539	3.7597e-05	6.5268	7.9601e-11
group_2:perCorr_2	-0.0011044	0.00028823	-3.8318	0.00013005
group_2:concentration	-0.00045933	5.1482e-05	-8.9221	8.1242e-19
perCorr_2:concentration	-0.00028827	5.3171e-05	-5.4217	6.4126e-08
group_2:perCorr_2:concentration	0.00041357	7.401e-05	5.588	2.5194e-08

2784 observations, 2776 error degrees of freedom
Estimated Dispersion: 2.77e-06
F-statistic vs. constant model: 61.2, p-value = 4.27e-82

Ranksum or t-test for mi for theta Beta

pFDR = 3.278986e-02

p value ranksum for S+ high -1.4949 Proficient vs S+ low 0.50515 Naive = 3.175948e-31
p value ranksum for S+ high -1 Proficient vs S+ low 0.50515 Naive = 3.583183e-31
p value ranksum for S+ high -1 Proficient vs S+ low -1 Naive = 2.034462e-29
p value ranksum for S+ high -1 Proficient vs S+ high -1 Naive = 1.605802e-28
p value ranksum for S+ high -1 Naive vs S+ high -1.4949 Proficient = 3.668589e-28
p value ranksum for S+ high -1.4949 Proficient vs S+ low -1 Naive = 5.791757e-28
p value ranksum for S+ high 0 Naive vs S+ high -1.4949 Proficient = 1.372724e-27
p value ranksum for S+ high 0 Naive vs S+ high -1 Proficient = 1.883608e-27
p value ranksum for S+ high -0.49485 Proficient vs S+ low -1 Naive = 3.837917e-25
p value ranksum for S+ high -0.49485 Proficient vs S+ high -1 Naive = 8.679265e-25
p value ranksum for S+ high 0 Naive vs S+ high -0.49485 Proficient = 1.323810e-23
p value ranksum for S+ high -0.49485 Proficient vs S+ low 0.50515 Naive = 3.037880e-23
p value ranksum for S+ high -1 Proficient vs S+ low -1.4949 Naive = 4.882856e-23
p value ranksum for S+ high -1 Proficient vs S+ low -0.49485 Naive = 6.924517e-23
p value ranksum for S+ high -1.4949 Proficient vs S+ low -0.49485 Naive = 1.105991e-21
p value ranksum for S+ high -1.4949 Proficient vs S+ low -1.4949 Naive = 1.150973e-21

p value ranksum for S+ high -1 Proficient vs S+ low 0 Naive = 4.780511e-21
p value ranksum for S+ high -1.4949 Proficient vs S+ low 0 Naive = 6.030396e-20
p value ranksum for S+ low 1 Proficient vs S+ low 0.50515 Naive = 8.484417e-20
p value ranksum for S+ low 1 Proficient vs S+ low -1 Naive = 7.812467e-19
p value ranksum for S+ low 0.50515 Proficient vs S+ low -1 Naive = 8.110066e-18
p value ranksum for S+ high -0.49485 Naive vs S+ high -1 Proficient = 1.346534e-17
p value ranksum for S+ high -1 Naive vs S+ low 1 Proficient = 1.383217e-17
p value ranksum for S+ high -0.49485 Naive vs S+ high -1.4949 Proficient = 5.254337e-17
p value ranksum for S+ high -0.49485 Proficient vs S+ low 0 Naive = 1.598699e-16
p value ranksum for S+ high 0 Naive vs S+ low 1 Proficient = 1.640717e-16
p value ranksum for S+ high -1 Proficient vs S+ low -1 Proficient = 3.673101e-16
p value ranksum for S+ high 1 Naive vs S+ high -1 Proficient = 1.093424e-15
p value ranksum for S+ high -1.4949 Proficient vs S+ low -1 Proficient = 1.284644e-15
p value ranksum for S+ high -0.49485 Proficient vs S+ low -0.49485 Naive = 1.775571e-15
p value ranksum for S+ low 0.50515 Proficient vs S+ low 0.50515 Naive = 2.936915e-15
p value ranksum for S+ low 1 Proficient vs S+ low -1.4949 Naive = 9.185241e-15
p value ranksum for S+ high 0.50515 Proficient vs S+ low -1 Naive = 9.974062e-15
p value ranksum for S+ high 1 Naive vs S+ high -1.4949 Proficient = 1.423562e-14
p value ranksum for S+ high -0.49485 Proficient vs S+ high -0.49485 Naive = 2.577748e-14
p value ranksum for S+ high -0.49485 Proficient vs S+ low -1.4949 Naive = 3.488913e-14
p value ranksum for S+ low 1 Proficient vs S+ low -0.49485 Naive = 4.090063e-14
p value ranksum for S+ high -1 Naive vs S+ low 0.50515 Proficient = 7.656271e-14
p value ranksum for S+ low 1 Proficient vs S+ low 0 Naive = 8.946233e-14
p value ranksum for S+ high -1 Proficient vs S+ low 1 Naive = 1.894165e-13
p value ranksum for S+ high 0 Naive vs S+ low 0.50515 Proficient = 1.963898e-13
p value ranksum for S+ high -1 Proficient vs S+ high -1.4949 Naive = 2.209920e-13
p value ranksum for S+ high -1.4949 Proficient vs S+ high -1.4949 Naive = 4.077522e-13
p value ranksum for S+ high -1.4949 Proficient vs S+ low 1 Naive = 8.686360e-13
p value ranksum for S+ low 1 Naive vs S+ low -1 Naive = 9.794169e-13
p value ranksum for S+ high 0.50515 Proficient vs S+ low 0.50515 Naive = 1.381001e-12
p value ranksum for S+ high -0.49485 Proficient vs S+ low -1 Proficient = 3.543922e-12
p value ranksum for S+ high 0.50515 Proficient vs S+ high -1 Naive = 6.828957e-12
p value ranksum for S+ high 0.50515 Proficient vs S+ high 0 Naive = 4.073431e-11
p value ranksum for S+ high 1 Proficient vs S+ low -1 Naive = 6.611619e-11
p value ranksum for S+ low 1 Proficient vs S+ low -1 Proficient = 7.328639e-11
p value ranksum for S+ high 0 Proficient vs S+ low -1 Naive = 1.884847e-10
p value ranksum for S+ high 0.50515 Proficient vs S+ low 0 Naive = 1.962389e-10
p value ranksum for S+ high -0.49485 Naive vs S+ low 1 Proficient = 2.280950e-10
p value ranksum for S+ low 0.50515 Proficient vs S+ low 0 Naive = 4.259385e-10
p value ranksum for S+ high 1 Naive vs S+ low 1 Proficient = 8.861895e-10
p value ranksum for S+ low 0.50515 Proficient vs S+ low -1.4949 Naive = 1.411879e-09
p value ranksum for S+ high -1 Proficient vs S+ low -0.49485 Proficient = 1.603278e-09
p value ranksum for S+ high -1 Proficient vs S+ low 0 Proficient = 1.621882e-09
p value ranksum for S+ high 1 Proficient vs S+ high -1 Naive = 3.161301e-09
p value ranksum for S+ high -1.4949 Proficient vs S+ low 0 Proficient = 3.220682e-09
p value ranksum for S+ high 0.50515 Proficient vs S+ low -1.4949 Naive = 3.494535e-09
p value ranksum for S+ high 1 Proficient vs S+ low 0.50515 Naive = 4.213909e-09
p value ranksum for S+ high 1 Naive vs S+ high -0.49485 Proficient = 4.540358e-09
p value ranksum for S+ low 0.50515 Proficient vs S+ low -0.49485 Naive = 6.178887e-09
p value ranksum for S+ high -1 Naive vs S+ low 1 Naive = 7.169490e-09
p value ranksum for S+ high 0.50515 Proficient vs S+ low -0.49485 Naive = 9.849846e-09

p value ranksum for S+ high -1 Proficient vs S+ low -1.4949 Proficient = 1.109570e-08
p value ranksum for S+ high 1 Proficient vs S+ high 0 Naive = 1.112141e-08
p value ranksum for S+ high 1 Proficient vs S+ high -1 Proficient = 1.167352e-08
p value ranksum for S+ high -0.49485 Proficient vs S+ high -1.4949 Naive = 1.715936e-08
p value ranksum for S+ high 0 Naive vs S+ low 1 Naive = 2.571818e-08
p value ranksum for S+ high 0 Proficient vs S+ high -1 Proficient = 2.633378e-08
p value ranksum for S+ low 1 Naive vs S+ low 0.50515 Naive = 2.664692e-08
p value ranksum for S+ low 0.50515 Proficient vs S+ low -1 Proficient = 2.831957e-08
p value ranksum for S+ high -1.4949 Naive vs S+ low 1 Proficient = 3.061482e-08
p value ranksum for S+ high 0.50515 Naive vs S+ high -1.4949 Proficient = 3.069814e-08
p value ranksum for S+ high 0 Proficient vs S+ low 0.50515 Naive = 3.217681e-08
p value ranksum for S+ high -0.49485 Proficient vs S+ low 1 Naive = 3.703935e-08
p value ranksum for S+ high -1.4949 Proficient vs S+ low -1.4949 Proficient = 4.480293e-08
p value ranksum for S+ high -1.4949 Naive vs S+ low -1 Naive = 5.629551e-08
p value ranksum for S+ high -0.49485 Naive vs S+ low 0.50515 Proficient = 7.786054e-08
p value ranksum for S+ high -1.4949 Proficient vs S+ low -0.49485 Proficient = 8.031189e-08
p value ranksum for S+ high 0.50515 Naive vs S+ high -1 Proficient = 8.224147e-08
p value ranksum for S+ high 0.50515 Proficient vs S+ low -1 Proficient = 1.439007e-07
p value ranksum for S+ high 0 Proficient vs S+ high -1 Naive = 1.693498e-07
p value ranksum for S+ low 1 Proficient vs S+ low 1 Naive = 1.889643e-07
p value ranksum for S+ high -1 Proficient vs S+ low 0.50515 Proficient = 2.475314e-07
p value ranksum for S+ high 0 Proficient vs S+ high 0 Naive = 3.074390e-07
p value ranksum for S+ high 0 Proficient vs S+ high -1.4949 Proficient = 3.660078e-07
p value ranksum for S+ high 0.50515 Naive vs S+ high -0.49485 Proficient = 3.948924e-07
p value ranksum for S+ high -1.4949 Naive vs S+ low 0.50515 Naive = 4.744923e-07
p value ranksum for S+ high 1 Proficient vs S+ high -1.4949 Proficient = 6.404086e-07
p value ranksum for S+ high 0.50515 Proficient vs S+ high -0.49485 Naive = 1.204492e-06
p value ranksum for S+ high -0.49485 Proficient vs S+ low 0 Proficient = 1.758438e-06
p value ranksum for S+ high 1 Proficient vs S+ low 0 Naive = 2.254981e-06
p value ranksum for S+ high 1 Naive vs S+ high 0.50515 Proficient = 2.624800e-06
p value ranksum for S+ high -1.4949 Proficient vs S+ low 0.50515 Proficient = 2.808844e-06
p value ranksum for S+ low 1 Naive vs S+ low 0 Naive = 4.157738e-06
p value ranksum for S+ high -0.49485 Proficient vs S+ low -0.49485 Proficient = 4.369084e-06
p value ranksum for S+ high 0.50515 Naive vs S+ low 1 Proficient = 4.447822e-06
p value ranksum for S+ high -0.49485 Proficient vs S+ low -1.4949 Proficient = 5.408688e-06
p value ranksum for S+ low 1 Proficient vs S+ low 0 Proficient = 5.799812e-06
p value ranksum for S+ low 1 Proficient vs S+ low -0.49485 Proficient = 6.485071e-06
p value ranksum for S+ high 0 Proficient vs S+ low 0 Naive = 9.063841e-06
p value ranksum for S+ high -1 Naive vs S+ high -1.4949 Naive = 1.264201e-05
p value ranksum for S+ low 1 Naive vs S+ low -1 Proficient = 1.634556e-05
p value ranksum for S+ high 1 Naive vs S+ low 0.50515 Proficient = 1.869696e-05
p value ranksum for S+ low 1 Proficient vs S+ low -1.4949 Proficient = 3.573357e-05
p value ranksum for S+ high -0.49485 Naive vs S+ low 1 Naive = 3.912454e-05
p value ranksum for S+ high 0.50515 Proficient vs S+ high 0.50515 Naive = 4.239358e-05
p value ranksum for S+ high 1 Proficient vs S+ low -0.49485 Naive = 5.150979e-05
p value ranksum for S+ high 0 Naive vs S+ high -1.4949 Naive = 5.242488e-05
p value ranksum for S+ high 0.50515 Proficient vs S+ high -1.4949 Naive = 5.288818e-05
p value ranksum for S+ high 1 Proficient vs S+ low -1.4949 Naive = 6.246355e-05
p value ranksum for S+ high 1 Proficient vs S+ high -0.49485 Naive = 6.638829e-05
p value ranksum for S+ high 0 Proficient vs S+ low -1 Proficient = 7.456707e-05
p value ranksum for S+ high 1 Naive vs S+ low 0.50515 Naive = 8.454517e-05

p value ranksum for S+ high 1 Proficient vs S+ low -1 Proficient = 1.055972e-04
p value ranksum for S+ high 0.50515 Proficient vs S+ low -0.49485 Proficient = 1.166176e-04
p value ranksum for S+ low 1 Naive vs S+ low -1.4949 Naive = 1.404248e-04
p value ranksum for S+ high -1.4949 Naive vs S+ low 0 Naive = 1.415999e-04
p value ranksum for S+ high 0.50515 Naive vs S+ low 0.50515 Naive = 1.657646e-04
p value ranksum for S+ high 0 Proficient vs S+ low -1.4949 Naive = 1.799919e-04
p value ranksum for S+ high 0.50515 Proficient vs S+ low 1 Naive = 2.432468e-04
p value ranksum for S+ high -1.4949 Naive vs S+ low -1 Proficient = 2.510641e-04
p value ranksum for S+ low 1 Naive vs S+ low -0.49485 Naive = 2.744035e-04
p value ranksum for S+ high 0 Proficient vs S+ low -0.49485 Naive = 3.019817e-04
p value ranksum for S+ high 0.50515 Naive vs S+ low -1 Naive = 3.428026e-04
p value ranksum for S+ high 1 Proficient vs S+ low 1 Proficient = 3.471523e-04
p value ranksum for S+ high 0 Proficient vs S+ low 1 Proficient = 5.256155e-04
p value ranksum for S+ high 0 Proficient vs S+ high -0.49485 Naive = 5.677411e-04
p value ranksum for S+ high -0.49485 Proficient vs S+ high -1 Proficient = 6.759571e-04
p value ranksum for S+ low 1 Proficient vs S+ low 0.50515 Proficient = 7.786176e-04
p value ranksum for S+ low 0 Proficient vs S+ low -1 Naive = 9.302873e-04
p value ranksum for S+ high 0.50515 Proficient vs S+ low 0 Proficient = 9.746038e-04
p value ranksum for S+ high 0 Proficient vs S+ high -0.49485 Proficient = 1.134674e-03
p value ranksum for S+ high 1 Naive vs S+ low -1 Naive = 1.555710e-03
p value ranksum for S+ high 0.50515 Proficient vs S+ low -1.4949 Proficient = 1.671842e-03
p value ranksum for S+ high 0.50515 Proficient vs S+ high -1 Proficient = 1.816370e-03
p value ranksum for S+ low -1 Naive vs S+ low -1.4949 Proficient = 1.862085e-03
p value ranksum for S+ high -1.4949 Naive vs S+ low 0.50515 Proficient = 2.317827e-03
p value ranksum for S+ low -0.49485 Naive vs S+ low -1 Naive = 2.379124e-03
p value ranksum for S+ high 1 Proficient vs S+ high -0.49485 Proficient = 2.461814e-03
p value ranksum for S+ high -1.4949 Naive vs S+ low -1.4949 Naive = 2.635133e-03
p value ranksum for S+ high -1.4949 Naive vs S+ low -0.49485 Naive = 2.838615e-03
p value ranksum for S+ low 0.50515 Proficient vs S+ low -0.49485 Proficient = 3.881921e-03
p value ranksum for S+ low 0.50515 Proficient vs S+ low 0 Proficient = 4.095390e-03
p value ranksum for S+ high -0.49485 Proficient vs S+ low 0.50515 Proficient = 4.628091e-03
p value ranksum for S+ high 1 Naive vs S+ low 0 Naive = 4.775503e-03
p value ranksum for S+ low 0.50515 Naive vs S+ low 0 Proficient = 5.257146e-03
p value ranksum for S+ high 0.50515 Naive vs S+ low 0 Naive = 5.392157e-03
p value ranksum for S+ low 0.50515 Naive vs S+ low -1.4949 Proficient = 5.472925e-03
p value ranksum for S+ high 0.50515 Naive vs S+ low -1 Proficient = 6.310365e-03
p value ranksum for S+ high -0.49485 Naive vs S+ high -1.4949 Naive = 6.803153e-03
p value ranksum for S+ high 1 Naive vs S+ high -1 Naive = 7.327412e-03
p value ranksum for S+ high 0.50515 Naive vs S+ low -1.4949 Naive = 7.791679e-03
p value ranksum for S+ high 1 Naive vs S+ high 0 Proficient = 7.887635e-03
p value ranksum for S+ high -0.49485 Proficient vs S+ high -1.4949 Proficient = 7.887635e-03
p value ranksum for S+ high 1 Proficient vs S+ high 1 Naive = 9.631175e-03
p value ranksum for S+ high 1 Naive vs S+ low -1 Proficient = 9.926418e-03
p value ranksum for S+ low -1 Naive vs S+ low -1.4949 Naive = 1.385675e-02
p value ranksum for S+ low 0.50515 Proficient vs S+ low -1.4949 Proficient = 1.387451e-02
p value ranksum for S+ low 0 Naive vs S+ low -1.4949 Proficient = 1.429399e-02
p value ranksum for S+ high 0.50515 Naive vs S+ low 0.50515 Proficient = 1.466813e-02
p value ranksum for S+ high 1 Naive vs S+ high 0 Naive = 1.535736e-02
p value ranksum for S+ high -1 Naive vs S+ low 0 Proficient = 1.576204e-02
p value ranksum for S+ high -0.49485 Naive vs S+ low -1 Naive = 1.588843e-02
p value ranksum for S+ high 0.50515 Naive vs S+ high -1 Naive = 1.671531e-02

p value ranksum for S+ low 1 Naive vs S+ low 0.50515 Proficient = 1.789480e-02
p value ranksum for S+ low 0 Proficient vs S+ low 0 Naive = 1.891066e-02
p value ranksum for S+ low 0 Proficient vs S+ low -1 Proficient = 1.916404e-02
p value ranksum for S+ high -1 Naive vs S+ low -1.4949 Proficient = 1.958306e-02
p value ranksum for S+ low 0.50515 Naive vs S+ low -1.4949 Naive = 2.109342e-02
p value ranksum for S+ high 0 Proficient vs S+ low -0.49485 Proficient = 2.204952e-02
p value ranksum for S+ high 1 Proficient vs S+ high 0.50515 Proficient = 2.275993e-02
p value ranksum for S+ low 0.50515 Naive vs S+ low -0.49485 Naive = 2.275993e-02
p value ranksum for S+ low -1 Proficient vs S+ low -1.4949 Proficient = 2.788995e-02
p value ranksum for S+ high 0.50515 Naive vs S+ high 0 Naive = 2.983452e-02
p value ranksum for S+ high 0.50515 Proficient vs S+ low 0.50515 Proficient = 3.157588e-02
p value ranksum for S+ high 0.50515 Naive vs S+ low -0.49485 Naive = 3.274753e-02

p values below are > pFDR

p value ranksum for S+ high 1 Naive vs S+ low 1 Naive = 3.394442e-02
p value ranksum for S+ high 0 Naive vs S+ low -1 Naive = 3.535791e-02
p value ranksum for S+ high 0.50515 Naive vs S+ high 0 Proficient = 3.719512e-02
p value ranksum for S+ high 0.50515 Proficient vs S+ high 0 Proficient = 3.872283e-02
p value ranksum for S+ high 0 Naive vs S+ low 0 Proficient = 4.307216e-02
p value ranksum for S+ high 1 Proficient vs S+ low -0.49485 Proficient = 4.345789e-02
p value ranksum for S+ high -1 Naive vs S+ low -1 Naive = 4.787733e-02
p value ranksum for S+ high 0 Naive vs S+ low -1.4949 Proficient = 5.069484e-02
p value ranksum for S+ high 1 Proficient vs S+ high 0.50515 Naive = 5.097976e-02
p value ranksum for S+ low 0 Naive vs S+ low -0.49485 Naive = 5.297135e-02
p value ranksum for S+ high -1 Naive vs S+ low -0.49485 Naive = 5.322481e-02
p value ranksum for S+ high 1 Proficient vs S+ high -1.4949 Naive = 5.661300e-02
p value ranksum for S+ high 0.50515 Proficient vs S+ high -1.4949 Proficient = 6.542164e-02
p value ranksum for S+ low 1 Naive vs S+ low -0.49485 Proficient = 7.225533e-02
p value ranksum for S+ low -0.49485 Naive vs S+ low -1 Proficient = 8.092861e-02
p value ranksum for S+ low -1.4949 Proficient vs S+ low -1.4949 Naive = 8.591190e-02
p value ranksum for S+ high 1 Proficient vs S+ low 0 Proficient = 8.728184e-02
p value ranksum for S+ high 0 Proficient vs S+ high -1.4949 Naive = 8.911393e-02
p value ranksum for S+ high 1 Naive vs S+ low -1.4949 Naive = 8.989224e-02
p value ranksum for S+ high 0 Naive vs S+ low -0.49485 Naive = 1.075368e-01
p value ranksum for S+ high 0 Proficient vs S+ low -1.4949 Proficient = 1.108540e-01
p value ranksum for S+ low 0 Proficient vs S+ low -1.4949 Naive = 1.112718e-01
p value ranksum for S+ high 1 Naive vs S+ high -1.4949 Naive = 1.121182e-01
p value ranksum for S+ high 0 Proficient vs S+ low 0 Proficient = 1.125326e-01
p value ranksum for S+ high 1 Proficient vs S+ low -1.4949 Proficient = 1.207814e-01
p value ranksum for S+ low 0 Naive vs S+ low -1.4949 Naive = 1.217415e-01
p value ranksum for S+ low -1 Proficient vs S+ low -1.4949 Naive = 1.290255e-01
p value ranksum for S+ low -0.49485 Naive vs S+ low -1.4949 Proficient = 1.357346e-01
p value ranksum for S+ high 1 Proficient vs S+ low 1 Naive = 1.384675e-01
p value ranksum for S+ high 0 Naive vs S+ low -1.4949 Naive = 1.384675e-01
p value ranksum for S+ low -0.49485 Proficient vs S+ low -1 Naive = 1.574821e-01
p value ranksum for S+ high -0.49485 Naive vs S+ low -1 Proficient = 1.602377e-01
p value ranksum for S+ high 1 Naive vs S+ low -0.49485 Naive = 1.661719e-01
p value ranksum for S+ high 0.50515 Naive vs S+ low 1 Naive = 1.661719e-01
p value ranksum for S+ high 1 Naive vs S+ high -0.49485 Naive = 1.706320e-01
p value ranksum for S+ low 0 Proficient vs S+ low -0.49485 Naive = 1.716256e-01

p value ranksum for S+ high -1.4949 Naive vs S+ low -0.49485 Proficient = 1.722105e-01
p value ranksum for S+ high -0.49485 Naive vs S+ low 0 Naive = 1.784857e-01
p value ranksum for S+ low 1 Naive vs S+ low 0 Proficient = 1.805563e-01
p value ranksum for S+ high -0.49485 Naive vs S+ low 0 Proficient = 1.981426e-01
p value ranksum for S+ high -1 Naive vs S+ low -1.4949 Naive = 1.985704e-01
p value ranksum for S+ low -0.49485 Proficient vs S+ low -1.4949 Proficient = 2.094249e-01
p value ranksum for S+ high -1 Proficient vs S+ low 1 Proficient = 2.176447e-01
p value ranksum for S+ high 1 Naive vs S+ high 0.50515 Naive = 2.179400e-01
p value ranksum for S+ low 1 Naive vs S+ low -1.4949 Proficient = 2.289777e-01
p value ranksum for S+ high -0.49485 Naive vs S+ low -1.4949 Proficient = 2.392334e-01
p value ranksum for S+ high 0 Naive vs S+ low -1 Proficient = 2.422222e-01
p value ranksum for S+ low 0.50515 Naive vs S+ low -0.49485 Proficient = 2.614975e-01
p value ranksum for S+ high 0 Proficient vs S+ low 0.50515 Proficient = 2.703058e-01
p value ranksum for S+ high 0.50515 Naive vs S+ low -0.49485 Proficient = 2.859949e-01
p value ranksum for S+ low 0 Naive vs S+ low -0.49485 Proficient = 2.876815e-01
p value ranksum for S+ high -0.49485 Proficient vs S+ low 1 Proficient = 3.066764e-01
p value ranksum for S+ low 0 Proficient vs S+ low -0.49485 Proficient = 3.296032e-01
p value ranksum for S+ high 0.50515 Naive vs S+ high -0.49485 Naive = 3.680948e-01
p value ranksum for S+ high -1.4949 Naive vs S+ low 1 Naive = 3.702934e-01
p value ranksum for S+ high -0.49485 Naive vs S+ low 0.50515 Naive = 3.836557e-01
p value ranksum for S+ high -0.49485 Naive vs S+ low -0.49485 Naive = 4.089079e-01
p value ranksum for S+ high 1 Naive vs S+ low -0.49485 Proficient = 4.106169e-01
p value ranksum for S+ high -1 Naive vs S+ low -1 Proficient = 4.138041e-01
p value ranksum for S+ high 0.50515 Proficient vs S+ low 1 Proficient = 4.387219e-01
p value ranksum for S+ high 0 Proficient vs S+ low 1 Naive = 4.597767e-01
p value ranksum for S+ high 1 Proficient vs S+ low 0.50515 Proficient = 4.817231e-01
p value ranksum for S+ low -0.49485 Proficient vs S+ low -1 Proficient = 5.032683e-01
p value ranksum for S+ high -1 Naive vs S+ low -0.49485 Proficient = 5.124171e-01
p value ranksum for S+ high -1.4949 Proficient vs S+ low 1 Proficient = 5.136177e-01
p value ranksum for S+ low 0 Naive vs S+ low -1 Naive = 5.287401e-01
p value ranksum for S+ high 1 Naive vs S+ low -1.4949 Proficient = 5.367037e-01
p value ranksum for S+ high 0 Naive vs S+ high -0.49485 Naive = 5.588388e-01
p value ranksum for S+ high -1.4949 Naive vs S+ low 0 Proficient = 5.615559e-01
p value ranksum for S+ high 0 Naive vs S+ low 0 Naive = 5.713873e-01
p value ranksum for S+ low 0.50515 Naive vs S+ low -1 Naive = 5.741940e-01
p value ranksum for S+ high -1 Naive vs S+ low 0 Naive = 6.040507e-01
p value ranksum for S+ low -0.49485 Naive vs S+ low -1.4949 Naive = 6.112609e-01
p value ranksum for S+ high 0 Naive vs S+ low -0.49485 Proficient = 6.194239e-01
p value ranksum for S+ high -1 Proficient vs S+ high -1.4949 Proficient = 6.243344e-01
p value ranksum for S+ high -0.49485 Naive vs S+ high -1 Naive = 6.434280e-01
p value ranksum for S+ high -0.49485 Naive vs S+ low -0.49485 Proficient = 6.553459e-01
p value ranksum for S+ high 1 Naive vs S+ low 0 Proficient = 6.580401e-01
p value ranksum for S+ high 0.50515 Naive vs S+ low -1.4949 Proficient = 7.183840e-01
p value ranksum for S+ high -1.4949 Naive vs S+ low -1.4949 Proficient = 7.183840e-01
p value ranksum for S+ low -0.49485 Proficient vs S+ low -1.4949 Naive = 7.323639e-01
p value ranksum for S+ high 0 Naive vs S+ low 0.50515 Naive = 7.453631e-01
p value ranksum for S+ high 0.50515 Naive vs S+ low 0 Proficient = 7.862653e-01
p value ranksum for S+ high -0.49485 Naive vs S+ low -1.4949 Naive = 7.910383e-01
p value ranksum for S+ low 0.50515 Naive vs S+ low -1 Proficient = 7.934409e-01
p value ranksum for S+ high 0.50515 Proficient vs S+ high -0.49485 Proficient = 8.117593e-01
p value ranksum for S+ high 0 Naive vs S+ high -1 Naive = 8.229727e-01

p value ranksum for S+ low 0 Naive vs S+ low -1 Proficient = 8.237627e-01
p value ranksum for S+ high 1 Proficient vs S+ high 0 Proficient = 8.503516e-01
p value ranksum for S+ low -1 Proficient vs S+ low -1 Naive = 8.792747e-01
p value ranksum for S+ high 0.50515 Naive vs S+ high -1.4949 Naive = 9.350860e-01
p value ranksum for S+ high -1 Naive vs S+ low 0.50515 Naive = 9.383660e-01
p value ranksum for S+ low 0.50515 Naive vs S+ low 0 Naive = 9.761480e-01
p value ranksum for S+ low 0 Proficient vs S+ low -1.4949 Proficient = 9.818176e-01
p value ranksum for S+ low -0.49485 Proficient vs S+ low -0.49485 Naive = 9.888479e-01
