# Supplementary material for: The Olfactory Bulb Facilitates Use of Category Bounds for Classification of Odorants in Different Intensity Groups
Source: Front Cell Neurosci. 2020 Dec 11;14:613635. doi: 10.3389/fncel.2020.613635 (PMC7759615; doi:10.3389/fncel.2020.613635)
Supplement: Supplementary file 3 [file Table_3.pdf]

Table S3. Generalized linear regression model for Figure 2F, modulation index for gamma tPAC.

mi: modulation index
group: S+: high vs. S+ low
perCorr: naïve vs. proficient
concentration: log10(c_{liq})

Generalized linear regression model:

mi ~ 1 + group*perCorr + group*concentration + perCorr*concentration + group:perCorr:concentration

Distribution = Normal

Estimated Coefficients:

	Estimate	SE	tStat	pValue
(Intercept)	0.01721	0.00038508	44.692	0
group_2	-0.0083852	0.0005273	-15.902	1.46e-54
perCorr_2	-0.00314	0.00054459	-5.7658	9.0247e-09
concentration	-0.0012836	9.888e-05	-12.981	1.8796e-37
group_2:perCorr_2	0.0049288	0.00075804	6.5021	9.3583e-11
group_2:concentration	0.0019295	0.0001354	14.251	1.5866e-44
perCorr_2:concentration	0.00079382	0.00013984	5.6767	1.5152e-08
group_2:perCorr_2:concentration	-0.0012871	0.00019465	-6.6126	4.5114e-11

2784 observations, 2776 error degrees of freedom
Estimated Dispersion: 1.92e-05
F-statistic vs. constant model: 45.7, p-value = 1.35e-61

Ranksum or t-test for mi for theta High gamma

pFDR = 2.898551e-02

p value t-test for S+ high 1 Proficient vs S+ high -1.4949 Proficient = 2.857591e-32
p value t-test for S+ high 1 Proficient vs S+ high -1 Proficient = 4.641331e-25
p value t-test for S+ high 1 Proficient vs S+ low 0.50515 Proficient = 7.293447e-24
p value ranksum for S+ high 0.50515 Proficient vs S+ low 0.50515 Proficient = 9.558297e-24
p value t-test for S+ high 1 Proficient vs S+ high -0.49485 Proficient = 9.199549e-22
p value ranksum for S+ high 0.50515 Proficient vs S+ high -1 Proficient = 1.046679e-19
p value ranksum for S+ high 0.50515 Proficient vs S+ high -1.4949 Proficient = 1.046679e-19
p value ranksum for S+ high 0.50515 Proficient vs S+ high -0.49485 Proficient = 4.206418e-19
p value ranksum for S+ high 0.50515 Proficient vs S+ low 0 Proficient = 6.498478e-19
p value t-test for S+ high 1 Proficient vs S+ low 0 Proficient = 4.774217e-18
p value ranksum for S+ high 0.50515 Proficient vs S+ high -1 Naive = 9.345982e-17
p value ranksum for S+ high 0.50515 Proficient vs S+ low 0.50515 Naive = 2.916670e-16

p value t-test for S+ high 1 Proficient vs S+ high -1 Naive = 3.598792e-16
p value ranksum for S+ high 0.50515 Proficient vs S+ high -1.4949 Naive = 8.497345e-16
p value t-test for S+ high 1 Proficient vs S+ low 0.50515 Naive = 2.382844e-14
p value t-test for S+ high 1 Proficient vs S+ low -1 Naive = 5.003237e-14
p value t-test for S+ high 1 Proficient vs S+ low -0.49485 Naive = 5.027453e-14
p value t-test for S+ high 1 Proficient vs S+ low 1 Proficient = 6.340705e-14
p value ranksum for S+ high 0.50515 Proficient vs S+ low -1 Naive = 1.050312e-13
p value ranksum for S+ high 0.50515 Proficient vs S+ low 1 Proficient = 2.321067e-13
p value ranksum for S+ high 0.50515 Proficient vs S+ low -0.49485 Naive = 3.778354e-13
p value ranksum for S+ high 0.50515 Naive vs S+ low 0.50515 Proficient = 6.888145e-13
p value ranksum for S+ high 1 Naive vs S+ low 0.50515 Proficient = 7.274044e-13
p value ranksum for S+ high 0 Proficient vs S+ low 0.50515 Proficient = 1.393190e-12
p value t-test for S+ high 1 Proficient vs S+ high -0.49485 Naive = 3.154525e-12
p value t-test for S+ high 1 Proficient vs S+ low 0 Naive = 6.339925e-12
p value ranksum for S+ high 0.50515 Proficient vs S+ low -0.49485 Proficient = 8.981308e-12
p value ranksum for S+ high 0.50515 Proficient vs S+ low 0 Naive = 1.730045e-11
p value t-test for S+ high 1 Proficient vs S+ high 0 Proficient = 5.421090e-11
p value t-test for S+ high 1 Proficient vs S+ high -1.4949 Naive = 7.669794e-11
p value ranksum for S+ high 0 Proficient vs S+ high -1.4949 Proficient = 8.124161e-11
p value ranksum for S+ high 0 Proficient vs S+ low 0 Proficient = 1.124298e-10
p value ranksum for S+ high 0.50515 Naive vs S+ high -1.4949 Proficient = 2.274293e-10
p value ranksum for S+ high 1 Naive vs S+ high -1.4949 Proficient = 3.771093e-10
p value t-test for S+ high 1 Proficient vs S+ low -0.49485 Proficient = 6.349471e-10
p value ranksum for S+ high 0 Proficient vs S+ high -1 Proficient = 8.065891e-10
p value ranksum for S+ high 0.50515 Naive vs S+ low 0 Proficient = 1.106367e-09
p value t-test for S+ high 1 Proficient vs S+ low 1 Naive = 1.312285e-09
p value ranksum for S+ high 0.50515 Naive vs S+ high -1 Proficient = 1.459513e-09
p value ranksum for S+ high 1 Naive vs S+ high -1 Proficient = 2.520674e-09
p value ranksum for S+ high 0.50515 Proficient vs S+ high -0.49485 Naive = 2.823347e-09
p value t-test for S+ high 1 Proficient vs S+ high 0 Naive = 1.324974e-08
p value t-test for S+ high 1 Proficient vs S+ low -1.4949 Naive = 1.531243e-08
p value ranksum for S+ high 0 Proficient vs S+ high -0.49485 Proficient = 1.635646e-08
p value ranksum for S+ high 1 Naive vs S+ low 0 Proficient = 2.553744e-08
p value ranksum for S+ high 0.50515 Naive vs S+ high -0.49485 Proficient = 3.493236e-08
p value ranksum for S+ high 0 Naive vs S+ low 0.50515 Proficient = 4.527652e-08
p value ranksum for S+ high 0.50515 Proficient vs S+ low 1 Naive = 7.087203e-08
p value ranksum for S+ high 0 Proficient vs S+ low 1 Proficient = 8.031189e-08
p value ranksum for S+ high 0 Proficient vs S+ high -1.4949 Naive = 1.182647e-07
p value ranksum for S+ high 0 Proficient vs S+ low -1 Naive = 2.186384e-07
p value ranksum for S+ high 0.50515 Proficient vs S+ high 0 Naive = 3.318545e-07
p value ranksum for S+ high -0.49485 Naive vs S+ low 0.50515 Proficient = 3.975992e-07
p value ranksum for S+ high 0 Proficient vs S+ low -0.49485 Naive = 7.918081e-07
p value ranksum for S+ high 0.50515 Naive vs S+ high -1.4949 Naive = 9.372082e-07
p value ranksum for S+ high 0 Proficient vs S+ low 0.50515 Naive = 1.308876e-06
p value ranksum for S+ high 0 Proficient vs S+ high -1 Naive = 1.625936e-06
p value ranksum for S+ high 0.50515 Naive vs S+ high -1 Naive = 1.765161e-06
p value ranksum for S+ high 0 Proficient vs S+ low 0 Naive = 2.348387e-06
p value ranksum for S+ high 0 Naive vs S+ high -1.4949 Proficient = 2.348387e-06
p value ranksum for S+ low 0.50515 Proficient vs S+ low -1 Proficient = 2.815977e-06
p value t-test for S+ high 1 Proficient vs S+ high 0.50515 Naive = 2.843989e-06
p value ranksum for S+ high 1 Naive vs S+ high -1 Naive = 3.083225e-06

p value ranksum for S+ high 0 Naive vs S+ low 0 Proficient = 3.718052e-06
p value ranksum for S+ high 1 Naive vs S+ high 0.50515 Proficient = 3.727611e-06
p value ranksum for S+ low 0.50515 Proficient vs S+ low -1.4949 Proficient = 4.035880e-06
p value ranksum for S+ high 0.50515 Naive vs S+ low 0.50515 Naive = 4.869197e-06
p value ranksum for S+ high 0.50515 Naive vs S+ low 1 Proficient = 4.905774e-06
p value t-test for S+ high 1 Proficient vs S+ high 1 Naive = 5.059950e-06
p value ranksum for S+ high 1 Naive vs S+ low 1 Proficient = 5.361003e-06
p value ranksum for S+ high 0.50515 Proficient vs S+ low -1.4949 Proficient = 5.603521e-06
p value t-test for S+ high 1 Proficient vs S+ low -1.4949 Proficient = 1.079429e-05
p value ranksum for S+ high 0 Naive vs S+ high -1 Proficient = 1.469442e-05
p value ranksum for S+ high 0.50515 Proficient vs S+ high 0.50515 Naive = 1.997455e-05
p value ranksum for S+ high 0.50515 Naive vs S+ low -1 Naive = 2.314333e-05
p value ranksum for S+ high 0.50515 Naive vs S+ low -0.49485 Naive = 2.678689e-05
p value ranksum for S+ high 1 Naive vs S+ low 0.50515 Naive = 2.703185e-05
p value ranksum for S+ high 1 Naive vs S+ high -0.49485 Proficient = 2.987086e-05
p value ranksum for S+ low 0.50515 Proficient vs S+ low -1.4949 Naive = 3.199646e-05
p value ranksum for S+ low 0 Proficient vs S+ low -1 Proficient = 3.295330e-05
p value ranksum for S+ high 0 Proficient vs S+ low -0.49485 Proficient = 3.387398e-05
p value ranksum for S+ high -0.49485 Naive vs S+ low 0 Proficient = 4.113734e-05
p value t-test for S+ high -1 Naive vs S+ low 0.50515 Proficient = 4.568197e-05
p value ranksum for S+ high 1 Naive vs S+ high -1.4949 Naive = 4.592065e-05
p value ranksum for S+ low 0 Proficient vs S+ low -1.4949 Proficient = 6.109519e-05
p value t-test for S+ high 1 Proficient vs S+ low -1 Proficient = 6.164861e-05
p value ranksum for S+ high -1.4949 Proficient vs S+ low -1.4949 Proficient = 8.778590e-05
p value ranksum for S+ high -0.49485 Naive vs S+ high -1 Proficient = 1.055242e-04
p value ranksum for S+ high 0.50515 Naive vs S+ low 0 Naive = 1.119609e-04
p value ranksum for S+ high 0 Proficient vs S+ low 1 Naive = 1.280910e-04
p value ranksum for S+ high -0.49485 Naive vs S+ high -1.4949 Proficient = 1.513395e-04
p value ranksum for S+ high 0.50515 Proficient vs S+ low -1.4949 Naive = 1.741685e-04
p value ranksum for S+ high 0 Naive vs S+ high -0.49485 Proficient = 1.937708e-04
p value ranksum for S+ low 0.50515 Proficient vs S+ low -0.49485 Proficient = 3.182108e-04
p value ranksum for S+ high 0.50515 Proficient vs S+ low -1 Proficient = 3.422366e-04
p value ranksum for S+ low 0 Proficient vs S+ low -1.4949 Naive = 3.674831e-04
p value ranksum for S+ high 1 Naive vs S+ low 0 Naive = 4.138110e-04
p value ranksum for S+ low 1 Proficient vs S+ low -1 Proficient = 5.270063e-04
p value ranksum for S+ high -1.4949 Naive vs S+ low 0.50515 Proficient = 6.117513e-04
p value ranksum for S+ high -1 Proficient vs S+ low -1 Proficient = 6.462053e-04
p value ranksum for S+ high -1 Proficient vs S+ low -1.4949 Proficient = 7.454217e-04
p value ranksum for S+ high -1.4949 Proficient vs S+ low -1 Proficient = 8.303180e-04
p value ranksum for S+ high 0 Proficient vs S+ high -0.49485 Naive = 8.844424e-04
p value ranksum for S+ high 0 Naive vs S+ low 1 Proficient = 8.997839e-04
p value ranksum for S+ low 1 Proficient vs S+ low -1.4949 Proficient = 1.010111e-03
p value ranksum for S+ high 1 Naive vs S+ low -0.49485 Naive = 1.070513e-03
p value ranksum for S+ high -1.4949 Proficient vs S+ low -0.49485 Proficient = 1.083339e-03
p value t-test for S+ high -1 Naive vs S+ high -1.4949 Proficient = 1.126027e-03
p value ranksum for S+ high 1 Naive vs S+ low 1 Naive = 1.358798e-03
p value ranksum for S+ high 0.50515 Naive vs S+ low -0.49485 Proficient = 1.415223e-03
p value ranksum for S+ low 0.50515 Naive vs S+ low -1 Proficient = 1.480481e-03
p value ranksum for S+ low 1 Naive vs S+ low 0.50515 Proficient = 1.578526e-03
p value ranksum for S+ high -1.4949 Proficient vs S+ low -1.4949 Naive = 1.867870e-03
p value ranksum for S+ high -0.49485 Proficient vs S+ high -0.49485 Naive = 2.102055e-03

p value ranksum for S+ high 0 Naive vs S+ low 0.50515 Naive = 2.298984e-03
p value ranksum for S+ high 0 Naive vs S+ high -1.4949 Naive = 2.330739e-03
p value ranksum for S+ low 0.50515 Naive vs S+ low -1.4949 Naive = 2.529850e-03
p value ranksum for S+ low 0.50515 Proficient vs S+ low -0.49485 Naive = 2.687111e-03
p value ranksum for S+ low 0 Proficient vs S+ low -0.49485 Proficient = 2.926207e-03
p value ranksum for S+ low 1 Proficient vs S+ low -1.4949 Naive = 3.485778e-03
p value ranksum for S+ high 0 Naive vs S+ low -1 Naive = 4.837267e-03
p value ranksum for S+ high -0.49485 Naive vs S+ low 0.50515 Naive = 4.868419e-03
p value ranksum for S+ high 0 Naive vs S+ low 0 Naive = 5.636887e-03
p value ranksum for S+ high 1 Naive vs S+ low -1 Naive = 6.041753e-03
p value ranksum for S+ high 0 Naive vs S+ high -1 Naive = 6.079828e-03
p value ranksum for S+ high -0.49485 Proficient vs S+ low -1.4949 Proficient = 7.061513e-03
p value ranksum for S+ high -0.49485 Naive vs S+ low 1 Proficient = 7.221042e-03
p value ranksum for S+ high -1 Proficient vs S+ high -1 Naive = 7.464016e-03
p value t-test for S+ high -1 Naive vs S+ low 0 Proficient = 7.817535e-03
p value ranksum for S+ high 0 Naive vs S+ low -0.49485 Naive = 8.332663e-03
p value ranksum for S+ low 0.50515 Proficient vs S+ low -1 Naive = 8.477690e-03
p value ranksum for S+ high -0.49485 Proficient vs S+ low -1 Proficient = 8.905639e-03
p value ranksum for S+ low 1 Naive vs S+ low 0 Proficient = 9.101957e-03
p value ranksum for S+ low 0 Naive vs S+ low -1 Proficient = 9.201557e-03
p value ranksum for S+ low 0.50515 Naive vs S+ low -1.4949 Proficient = 9.251721e-03
p value ranksum for S+ high 0 Proficient vs S+ low -1.4949 Naive = 9.402954e-03
p value t-test for S+ high -1 Naive vs S+ low -1 Proficient = 9.441003e-03
p value ranksum for S+ high -1.4949 Naive vs S+ low 0 Proficient = 9.506219e-03
p value ranksum for S+ high 0 Proficient vs S+ low -1.4949 Proficient = 9.873004e-03
p value ranksum for S+ high 0.50515 Naive vs S+ low 1 Naive = 1.010248e-02
p value ranksum for S+ high 0 Proficient vs S+ high 0 Naive = 1.022347e-02
p value ranksum for S+ high -1.4949 Proficient vs S+ high -1.4949 Naive = 1.022347e-02
p value ranksum for S+ low -0.49485 Naive vs S+ low -1.4949 Proficient = 1.064460e-02
p value ranksum for S+ low -1 Proficient vs S+ low -1 Naive = 1.099139e-02
p value ranksum for S+ low -0.49485 Naive vs S+ low -1 Proficient = 1.110920e-02
p value ranksum for S+ high -0.49485 Proficient vs S+ low -1.4949 Naive = 1.123875e-02
p value ranksum for S+ low -1 Naive vs S+ low -1.4949 Proficient = 1.241554e-02
p value ranksum for S+ high 1 Naive vs S+ high 0 Proficient = 1.330910e-02
p value t-test for S+ high -1 Naive vs S+ low 0.50515 Naive = 1.340152e-02
p value ranksum for S+ high 0.50515 Naive vs S+ high -0.49485 Naive = 1.346359e-02
p value ranksum for S+ high 0 Proficient vs S+ low -1 Proficient = 1.378434e-02
p value ranksum for S+ high -1 Proficient vs S+ low -1.4949 Naive = 1.570964e-02
p value ranksum for S+ high -1.4949 Naive vs S+ low -1.4949 Proficient = 1.789480e-02
p value ranksum for S+ low -1 Naive vs S+ low -1.4949 Naive = 1.868928e-02
p value ranksum for S+ low 0 Naive vs S+ low -1.4949 Proficient = 1.987757e-02
p value t-test for S+ high -1 Naive vs S+ low -1.4949 Proficient = 2.049603e-02
p value t-test for S+ high -1 Naive vs S+ low -1.4949 Naive = 2.127911e-02
p value ranksum for S+ high -1.4949 Proficient vs S+ low -1 Naive = 2.239349e-02
p value ranksum for S+ high -0.49485 Naive vs S+ low 0 Naive = 2.560985e-02
p value ranksum for S+ low -0.49485 Naive vs S+ low -1.4949 Naive = 2.686184e-02

p values below are > pFDR

p value ranksum for S+ high -0.49485 Proficient vs S+ low 0.50515 Proficient = 3.084573e-02
p value ranksum for S+ high -1.4949 Proficient vs S+ low -0.49485 Naive = 3.110048e-02

p value ranksum for S+ low 0 Naive vs S+ low -1.4949 Naive = 3.158697e-02
p value ranksum for S+ high 0.50515 Proficient vs S+ high 0 Proficient = 3.814381e-02
p value ranksum for S+ high 1 Naive vs S+ high -0.49485 Naive = 3.852900e-02
p value ranksum for S+ high -0.49485 Naive vs S+ high -1.4949 Naive = 3.872283e-02
p value ranksum for S+ low 1 Naive vs S+ low 0.50515 Naive = 4.493513e-02
p value ranksum for S+ low 1 Naive vs S+ low -1 Proficient = 4.583407e-02
p value ranksum for S+ low 0 Proficient vs S+ low -0.49485 Naive = 4.644492e-02
p value ranksum for S+ high -1.4949 Naive vs S+ low -1 Proficient = 4.665006e-02
p value ranksum for S+ low 0.50515 Proficient vs S+ low 0.50515 Naive = 4.810747e-02
p value ranksum for S+ high 0 Naive vs S+ low 1 Naive = 4.834404e-02
p value ranksum for S+ low 1 Proficient vs S+ low -0.49485 Proficient = 4.892544e-02
p value ranksum for S+ high -1 Proficient vs S+ low 1 Naive = 5.000772e-02
p value ranksum for S+ high -0.49485 Naive vs S+ high -1 Naive = 5.122523e-02
p value ranksum for S+ high -1.4949 Proficient vs S+ low 0.50515 Proficient = 5.622006e-02
p value ranksum for S+ low 0.50515 Proficient vs S+ low 0 Naive = 5.941657e-02
p value ranksum for S+ high -1 Proficient vs S+ low -0.49485 Proficient = 6.017526e-02
p value ranksum for S+ low 0 Proficient vs S+ low -1 Naive = 6.223778e-02
p value ranksum for S+ high -0.49485 Naive vs S+ low -1 Naive = 6.392884e-02
p value ranksum for S+ low 1 Proficient vs S+ low 1 Naive = 6.849542e-02
p value ranksum for S+ high -1 Proficient vs S+ high -1.4949 Naive = 7.465650e-02
p value ranksum for S+ high -0.49485 Naive vs S+ low -0.49485 Naive = 7.669430e-02
p value ranksum for S+ low -0.49485 Proficient vs S+ low -1 Proficient = 7.728495e-02
p value ranksum for S+ high 0 Naive vs S+ low -0.49485 Proficient = 7.900217e-02
p value t-test for S+ high -1 Naive vs S+ low 1 Proficient = 8.979394e-02
p value ranksum for S+ high 0.50515 Naive vs S+ high 0 Naive = 9.966513e-02
p value ranksum for S+ low -0.49485 Proficient vs S+ low -0.49485 Naive = 1.067439e-01
p value ranksum for S+ high -0.49485 Proficient vs S+ low 0 Proficient = 1.083732e-01
p value ranksum for S+ high 1 Naive vs S+ low -0.49485 Proficient = 1.100221e-01
p value ranksum for S+ high -1.4949 Naive vs S+ low -1.4949 Naive = 1.173340e-01
p value ranksum for S+ low -0.49485 Proficient vs S+ low -1.4949 Naive = 1.176890e-01
p value ranksum for S+ high -0.49485 Proficient vs S+ high -1.4949 Proficient = 1.242455e-01
p value ranksum for S+ high -1.4949 Proficient vs S+ low 1 Naive = 1.262775e-01
p value ranksum for S+ low -0.49485 Proficient vs S+ low -1.4949 Proficient = 1.301687e-01
p value ranksum for S+ high 0.50515 Naive vs S+ low -1.4949 Proficient = 1.304410e-01
p value ranksum for S+ low 0.50515 Naive vs S+ low -0.49485 Proficient = 1.372067e-01
p value ranksum for S+ high -0.49485 Naive vs S+ low 1 Naive = 1.521338e-01
p value ranksum for S+ high -0.49485 Proficient vs S+ low 1 Naive = 1.706320e-01
p value ranksum for S+ high -0.49485 Proficient vs S+ low -0.49485 Proficient = 1.739741e-01
p value ranksum for S+ low -0.49485 Proficient vs S+ low -1 Naive = 1.836086e-01
p value ranksum for S+ low 1 Naive vs S+ low -1.4949 Proficient = 1.848402e-01
p value ranksum for S+ high -1.4949 Naive vs S+ low 1 Proficient = 1.854583e-01
p value ranksum for S+ low 0 Proficient vs S+ low 0 Naive = 1.917233e-01
p value ranksum for S+ high -1 Proficient vs S+ low -0.49485 Naive = 2.058657e-01
p value ranksum for S+ low 1 Proficient vs S+ low 0.50515 Proficient = 2.082021e-01
p value ranksum for S+ high -1.4949 Naive vs S+ low -0.49485 Proficient = 2.128155e-01
p value t-test for S+ high -1 Naive vs S+ low 0 Naive = 2.138406e-01
p value ranksum for S+ high -0.49485 Proficient vs S+ high -1 Naive = 2.179400e-01
p value ranksum for S+ high -1 Proficient vs S+ low 0.50515 Proficient = 2.218464e-01
p value ranksum for S+ high -1.4949 Naive vs S+ low 0.50515 Naive = 2.281189e-01
p value ranksum for S+ low 1 Proficient vs S+ low -0.49485 Naive = 2.347991e-01
p value ranksum for S+ low 1 Proficient vs S+ low -1 Naive = 2.422222e-01

p value ranksum for S+ high 1 Naive vs S+ low -1 Proficient = 2.513459e-01
p value ranksum for S+ high 0.50515 Naive vs S+ high 0 Proficient = 2.520345e-01
p value ranksum for S+ high 0.50515 Naive vs S+ low -1 Proficient = 2.662771e-01
p value ranksum for S+ low 1 Naive vs S+ low -1.4949 Naive = 2.686208e-01
p value ranksum for S+ high -1 Proficient vs S+ low 0 Proficient = 2.711166e-01
p value ranksum for S+ high -1 Proficient vs S+ low -1 Naive = 2.822338e-01
p value ranksum for S+ low 1 Naive vs S+ low 0 Naive = 2.859416e-01
p value ranksum for S+ low 1 Naive vs S+ low -1 Naive = 3.108424e-01
p value ranksum for S+ low 0.50515 Naive vs S+ low 0 Proficient = 3.137851e-01
p value ranksum for S+ high 1 Naive vs S+ high 0.50515 Naive = 3.157904e-01
p value ranksum for S+ low -1 Proficient vs S+ low -1.4949 Naive = 3.255662e-01
p value ranksum for S+ high 0.50515 Naive vs S+ low -1.4949 Naive = 3.465577e-01
p value ranksum for S+ low 0.50515 Naive vs S+ low -0.49485 Naive = 3.476151e-01
p value ranksum for S+ high 0 Naive vs S+ high -0.49485 Naive = 3.550744e-01
p value ranksum for S+ high -0.49485 Naive vs S+ low -0.49485 Proficient = 3.577195e-01
p value ranksum for S+ low 0 Naive vs S+ low -0.49485 Proficient = 3.635945e-01
p value ranksum for S+ high -0.49485 Proficient vs S+ high -1.4949 Naive = 3.847824e-01
p value ranksum for S+ low 1 Proficient vs S+ low 0 Naive = 3.897439e-01
p value t-test for S+ high -1 Naive vs S+ low -1 Naive = 3.902086e-01
p value ranksum for S+ high 1 Naive vs S+ low -1.4949 Naive = 3.938686e-01
p value t-test for S+ high -1 Naive vs S+ low -0.49485 Naive = 4.027919e-01
p value ranksum for S+ low 1 Naive vs S+ low -0.49485 Naive = 4.124264e-01
p value ranksum for S+ high -1.4949 Naive vs S+ low 0 Naive = 4.302864e-01
p value ranksum for S+ high -1.4949 Proficient vs S+ low 0 Naive = 4.485874e-01
p value ranksum for S+ high -0.49485 Naive vs S+ low -1.4949 Proficient = 5.136177e-01
p value t-test for S+ high -1 Naive vs S+ low 1 Naive = 5.296845e-01
p value ranksum for S+ low 0.50515 Proficient vs S+ low 0 Proficient = 5.428013e-01
p value t-test for S+ high -1 Naive vs S+ low -0.49485 Proficient = 5.546475e-01
p value ranksum for S+ high -1.4949 Proficient vs S+ low 0.50515 Naive = 5.713873e-01
p value ranksum for S+ high -0.49485 Naive vs S+ low -1 Proficient = 5.716503e-01
p value ranksum for S+ high -1 Proficient vs S+ low 0 Naive = 5.755998e-01
p value ranksum for S+ low 0.50515 Naive vs S+ low -1 Naive = 5.826529e-01
p value ranksum for S+ high 1 Naive vs S+ high 0 Naive = 6.040507e-01
p value ranksum for S+ high -0.49485 Proficient vs S+ low 1 Proficient = 6.181100e-01
p value ranksum for S+ low 1 Proficient vs S+ low 0 Proficient = 6.202645e-01
p value ranksum for S+ high -1.4949 Proficient vs S+ low 0 Proficient = 6.246914e-01
p value ranksum for S+ high 0 Naive vs S+ low -1.4949 Naive = 6.463869e-01
p value ranksum for S+ high -0.49485 Proficient vs S+ high -1 Proficient = 6.523215e-01
p value ranksum for S+ high -1 Proficient vs S+ low 1 Proficient = 6.811177e-01
p value ranksum for S+ high 1 Naive vs S+ low -1.4949 Proficient = 6.879637e-01
p value ranksum for S+ high -0.49485 Proficient vs S+ low -0.49485 Naive = 7.360179e-01
p value ranksum for S+ high -1.4949 Naive vs S+ low 1 Naive = 7.484866e-01
p value ranksum for S+ low 0.50515 Naive vs S+ low 0 Naive = 7.500499e-01
p value ranksum for S+ low -0.49485 Naive vs S+ low -1 Naive = 7.578816e-01
p value ranksum for S+ low 0 Naive vs S+ low -0.49485 Naive = 7.751981e-01
p value ranksum for S+ low 1 Proficient vs S+ low 0.50515 Naive = 7.819687e-01
p value ranksum for S+ high -0.49485 Proficient vs S+ low -1 Naive = 8.005845e-01
p value ranksum for S+ low -1.4949 Proficient vs S+ low -1.4949 Naive = 8.035159e-01
p value ranksum for S+ high -1.4949 Naive vs S+ low -1 Naive = 8.053689e-01
p value ranksum for S+ low 1 Naive vs S+ low -0.49485 Proficient = 8.107326e-01
p value ranksum for S+ low -1 Proficient vs S+ low -1.4949 Proficient = 8.216917e-01

p value ranksum for S+ high -0.49485 Proficient vs S+ low 0.50515 Naive = 8.390541e-01
p value ranksum for S+ high -1 Proficient vs S+ low 0.50515 Naive = 8.422787e-01
p value t-test for S+ high 1 Proficient vs S+ high 0.50515 Proficient = 8.509872e-01
p value ranksum for S+ high -0.49485 Proficient vs S+ low 0 Naive = 8.519681e-01
p value ranksum for S+ high 0 Naive vs S+ low -1 Proficient = 8.881016e-01
p value ranksum for S+ high -0.49485 Naive vs S+ low -1.4949 Naive = 8.893058e-01
p value t-test for S+ high -1 Naive vs S+ high -1.4949 Naive = 9.297231e-01
p value ranksum for S+ high 0 Naive vs S+ low -1.4949 Proficient = 9.383514e-01
p value ranksum for S+ high -1.4949 Naive vs S+ low -0.49485 Naive = 9.695710e-01
p value ranksum for S+ low 0 Naive vs S+ low -1 Naive = 9.761480e-01
p value ranksum for S+ high -1.4949 Proficient vs S+ low 1 Proficient = 9.843876e-01
p value ranksum for S+ high -1 Proficient vs S+ high -1.4949 Proficient = 9.975322e-01
