# Supplementary material for: The Olfactory Bulb Facilitates Use of Category Bounds for Classification of Odorants in Different Intensity Groups
Source: Front Cell Neurosci. 2020 Dec 11;14:613635. doi: 10.3389/fncel.2020.613635 (PMC7759615; doi:10.3389/fncel.2020.613635)
Supplement: Supplementary file 4 [file Table_4.pdf]

Table S4. Generalized linear regression model for Figure 2G, peak angle variance for beta tPAC.

PA: peak angle variance
group: S+: high vs. S+ low
perCorr: naïve vs. proficient
concentration: log10(c_{liq})

Generalized linear regression model:

PA ~ 1 + group*perCorr + group*concentration + perCorr*concentration + group:perCorr:concentration

Distribution = Normal

Estimated Coefficients:

	Estimate	SE	tStat	pValue
(Intercept)	1561.7	52.59	29.695	1.6156e-168
group_2	1122.2	72.012	15.583	1.497e-52
perCorr_2	708.25	74.374	9.5228	3.5364e-21
concentration	210.61	13.504	15.596	1.2457e-52
group_2:perCorr_2	-675.11	103.52	-6.5213	8.2525e-11
group_2:concentration	-399.89	18.491	-21.626	5.5597e-96
perCorr_2:concentration	-193.41	19.098	-10.128	1.0692e-23
group_2:perCorr_2:concentration	306.46	26.583	11.529	4.5238e-30

2784 observations, 2776 error degrees of freedom
Estimated Dispersion: 3.57e+05
F-statistic vs. constant model: 100, p-value = 2.93e-131

Ranksum or t-test for PA variance for theta Beta

pFDR = 3.750000e-02

p value t-test for S+ low 0.50515 Naive vs S+ low -1 Proficient = 2.587220e-37
p value ranksum for S+ high -0.49485 Proficient vs S+ low -1 Proficient = 2.810760e-30
p value t-test for S+ low 0.50515 Naive vs S+ low -0.49485 Proficient = 2.139345e-28
p value ranksum for S+ high -1 Proficient vs S+ low -1 Proficient = 4.682784e-28
p value t-test for S+ low 0.50515 Naive vs S+ low -1.4949 Proficient = 5.955649e-28
p value ranksum for S+ high -1.4949 Proficient vs S+ low -1 Proficient = 8.506280e-28
p value ranksum for S+ high -0.49485 Proficient vs S+ low -1.4949 Proficient = 1.741543e-27
p value ranksum for S+ high 1 Proficient vs S+ high -0.49485 Proficient = 9.675126e-27
p value ranksum for S+ low 1 Naive vs S+ low -1 Proficient = 4.635218e-26
p value ranksum for S+ high 1 Proficient vs S+ low 0.50515 Naive = 5.791392e-26
p value ranksum for S+ high -0.49485 Proficient vs S+ low -0.49485 Proficient = 2.333381e-25
p value ranksum for S+ high -1 Proficient vs S+ low -0.49485 Proficient = 4.030522e-25

p value ranksum for S+ high -1 Proficient vs S+ low -1.4949 Proficient = 5.190067e-25
p value ranksum for S+ high -1.4949 Proficient vs S+ low -1.4949 Proficient = 1.022266e-24
p value ranksum for S+ high -1 Naive vs S+ low -1 Proficient = 2.724306e-24
p value ranksum for S+ high 1 Proficient vs S+ high -1 Proficient = 2.797255e-24
p value t-test for S+ low -0.49485 Naive vs S+ low -1 Proficient = 5.047883e-24
p value ranksum for S+ high 1 Proficient vs S+ high -1.4949 Proficient = 5.267760e-24
p value ranksum for S+ high -1.4949 Proficient vs S+ low -0.49485 Proficient = 5.264021e-23
p value ranksum for S+ low -1 Proficient vs S+ low -1 Naive = 8.525927e-23
p value ranksum for S+ low 1 Naive vs S+ low -1.4949 Proficient = 8.847271e-23
p value ranksum for S+ high 1 Proficient vs S+ low 1 Naive = 2.305856e-22
p value ranksum for S+ low 1 Naive vs S+ low -0.49485 Proficient = 1.160185e-21
p value ranksum for S+ low 1 Proficient vs S+ low -1 Proficient = 5.696473e-21
p value ranksum for S+ high 1 Proficient vs S+ high -1 Naive = 1.293552e-20
p value ranksum for S+ low 0.50515 Proficient vs S+ low -1 Proficient = 1.911568e-20
p value ranksum for S+ high 1 Proficient vs S+ low -1 Naive = 2.949576e-19
p value ranksum for S+ high -1.4949 Naive vs S+ low -1 Proficient = 2.995090e-19
p value t-test for S+ low -1 Naive vs S+ low -1.4949 Proficient = 4.631200e-19
p value ranksum for S+ high -1 Naive vs S+ low -1.4949 Proficient = 1.037609e-18
p value ranksum for S+ high 0 Naive vs S+ low -1 Proficient = 1.072765e-18
p value ranksum for S+ high -1 Naive vs S+ low -0.49485 Proficient = 1.146645e-18
p value ranksum for S+ high 0 Proficient vs S+ high -1 Proficient = 1.777654e-18
p value ranksum for S+ low 0 Proficient vs S+ low -1 Proficient = 2.090935e-18
p value ranksum for S+ high 0 Proficient vs S+ high -0.49485 Proficient = 5.690434e-18
p value ranksum for S+ high 0.50515 Proficient vs S+ high -0.49485 Proficient = 7.322909e-18
p value ranksum for S+ high 1 Naive vs S+ low -1 Proficient = 7.724408e-18
p value ranksum for S+ low -0.49485 Proficient vs S+ low -1 Naive = 1.158195e-17
p value ranksum for S+ high 0.50515 Proficient vs S+ high -1 Proficient = 1.638138e-17
p value ranksum for S+ low 1 Proficient vs S+ low -0.49485 Proficient = 1.119151e-16
p value ranksum for S+ low 0 Naive vs S+ low -1 Proficient = 1.298149e-16
p value ranksum for S+ high 1 Proficient vs S+ low 0.50515 Proficient = 3.052211e-16
p value ranksum for S+ high 0.50515 Proficient vs S+ low 0.50515 Naive = 3.768020e-16
p value ranksum for S+ low 1 Proficient vs S+ low -1.4949 Proficient = 4.891905e-16
p value ranksum for S+ high 1 Proficient vs S+ low 1 Proficient = 6.480602e-16
p value ranksum for S+ low 0.50515 Proficient vs S+ low -0.49485 Proficient = 6.823527e-16
p value ranksum for S+ high 0 Proficient vs S+ low 0.50515 Naive = 8.937521e-16
p value ranksum for S+ high 0 Proficient vs S+ high -1.4949 Proficient = 1.229564e-15
p value ranksum for S+ high 1 Proficient vs S+ low -0.49485 Naive = 1.314717e-15
p value t-test for S+ low -0.49485 Naive vs S+ low -1.4949 Proficient = 1.602002e-15
p value ranksum for S+ high 0.50515 Proficient vs S+ high -1.4949 Proficient = 2.028389e-15
p value ranksum for S+ high 0 Proficient vs S+ low 1 Naive = 2.028389e-15
p value ranksum for S+ low -0.49485 Proficient vs S+ low -0.49485 Naive = 3.784242e-15
p value ranksum for S+ high -0.49485 Naive vs S+ high -1 Proficient = 4.408301e-15
p value ranksum for S+ high -1.4949 Naive vs S+ low -0.49485 Proficient = 6.645138e-15
p value ranksum for S+ high 0 Naive vs S+ low -0.49485 Proficient = 6.997008e-15
p value ranksum for S+ high 0.50515 Proficient vs S+ low 1 Naive = 1.231052e-14
p value ranksum for S+ high -1.4949 Naive vs S+ low -1.4949 Proficient = 1.286171e-14
p value ranksum for S+ high 1 Proficient vs S+ high -1.4949 Naive = 1.423562e-14
p value ranksum for S+ high 1 Proficient vs S+ high 0 Naive = 2.093742e-14
p value ranksum for S+ high 0.50515 Naive vs S+ low -1 Proficient = 2.234284e-14
p value ranksum for S+ high 1 Proficient vs S+ low 0 Proficient = 2.581652e-14
p value ranksum for S+ low 0.50515 Proficient vs S+ low -1.4949 Proficient = 2.978240e-14

p value ranksum for S+ low 0 Proficient vs S+ low -0.49485 Proficient = 3.392751e-14
p value ranksum for S+ high -1 Proficient vs S+ low -1.4949 Naive = 1.573358e-13
p value t-test for S+ low 0.50515 Naive vs S+ low -1.4949 Naive = 1.588879e-13
p value ranksum for S+ low 0 Naive vs S+ low -0.49485 Proficient = 2.321067e-13
p value ranksum for S+ high 1 Naive vs S+ low -0.49485 Proficient = 2.353561e-13
p value ranksum for S+ low -1 Proficient vs S+ low -1.4949 Naive = 2.780116e-13
p value ranksum for S+ high -0.49485 Proficient vs S+ low -0.49485 Naive = 3.243241e-13
p value ranksum for S+ high 1 Proficient vs S+ high 1 Naive = 3.664819e-13
p value ranksum for S+ high 1 Proficient vs S+ low 0 Naive = 5.121593e-13
p value ranksum for S+ high -0.49485 Proficient vs S+ high -0.49485 Naive = 6.522466e-13
p value ranksum for S+ high -1 Proficient vs S+ low -0.49485 Naive = 1.172046e-12
p value ranksum for S+ low 0 Naive vs S+ low -1.4949 Proficient = 1.250859e-12
p value ranksum for S+ high 0 Naive vs S+ low -1.4949 Proficient = 1.948298e-12
p value ranksum for S+ high -0.49485 Proficient vs S+ low -1.4949 Naive = 7.887885e-12
p value ranksum for S+ high 1 Naive vs S+ low -1.4949 Proficient = 7.990235e-12
p value ranksum for S+ low 0 Proficient vs S+ low -1.4949 Proficient = 8.131483e-12
p value ranksum for S+ high 1 Naive vs S+ high -1 Proficient = 1.302135e-11
p value ranksum for S+ high 0.50515 Proficient vs S+ high -1 Naive = 2.327286e-11
p value ranksum for S+ high -0.49485 Naive vs S+ low 1 Naive = 4.877756e-11
p value ranksum for S+ high 0 Proficient vs S+ high -1 Naive = 4.947266e-11
p value ranksum for S+ high 0.50515 Naive vs S+ low -0.49485 Proficient = 5.547600e-11
p value ranksum for S+ high -0.49485 Naive vs S+ high -1.4949 Proficient = 6.342176e-11
p value ranksum for S+ high -1 Proficient vs S+ low 0 Proficient = 6.942252e-11
p value ranksum for S+ high -1.4949 Proficient vs S+ low -0.49485 Naive = 1.082316e-10
p value ranksum for S+ high -0.49485 Proficient vs S+ low 0 Proficient = 2.834359e-10
p value ranksum for S+ low -0.49485 Proficient vs S+ low -1.4949 Naive = 5.152290e-10
p value ranksum for S+ high 1 Proficient vs S+ high 0.50515 Naive = 7.268930e-10
p value ranksum for S+ high -1 Proficient vs S+ low -1 Naive = 7.961794e-10
p value ranksum for S+ low 1 Naive vs S+ low -1.4949 Naive = 8.065891e-10
p value ranksum for S+ high 0 Proficient vs S+ low -1 Naive = 8.278083e-10
p value t-test for S+ low 0.50515 Naive vs S+ low -0.49485 Naive = 9.672521e-10
p value ranksum for S+ high -0.49485 Proficient vs S+ low -1 Naive = 1.044965e-09
p value ranksum for S+ high -0.49485 Naive vs S+ low 0.50515 Naive = 1.100006e-09
p value ranksum for S+ high -1 Proficient vs S+ low 0.50515 Proficient = 1.145681e-09
p value ranksum for S+ high 0.50515 Naive vs S+ high -1 Proficient = 1.173462e-09
p value ranksum for S+ high 1 Naive vs S+ high -0.49485 Proficient = 1.459513e-09
p value ranksum for S+ high 0 Proficient vs S+ low 1 Proficient = 1.640683e-09
p value ranksum for S+ high -1 Proficient vs S+ low 0 Naive = 2.007167e-09
p value ranksum for S+ high 0.50515 Proficient vs S+ low -1 Naive = 2.336705e-09
p value ranksum for S+ low 1 Naive vs S+ low -0.49485 Naive = 2.336705e-09
p value ranksum for S+ high -1.4949 Proficient vs S+ low -1.4949 Naive = 3.282469e-09
p value ranksum for S+ high 1 Proficient vs S+ low -1.4949 Naive = 8.935432e-09
p value ranksum for S+ high -1 Proficient vs S+ high -1 Naive = 9.155979e-09
p value ranksum for S+ high 0 Proficient vs S+ low 0.50515 Proficient = 9.205580e-09
p value ranksum for S+ high 0.50515 Naive vs S+ low -1.4949 Proficient = 1.134144e-08
p value t-test for S+ low 0.50515 Naive vs S+ low 0 Naive = 1.207156e-08
p value ranksum for S+ high 0 Proficient vs S+ high -1.4949 Naive = 1.210499e-08
p value ranksum for S+ high 0 Proficient vs S+ high 0 Naive = 1.736599e-08
p value ranksum for S+ low -1.4949 Proficient vs S+ low -1.4949 Naive = 1.771726e-08
p value ranksum for S+ high 0.50515 Naive vs S+ high -0.49485 Proficient = 2.028224e-08
p value t-test for S+ low 0.50515 Naive vs S+ low -1 Naive = 2.551893e-08

p value ranksum for S+ high 0.50515 Proficient vs S+ low 1 Proficient = 2.553727e-08
p value ranksum for S+ high -1.4949 Proficient vs S+ low 0 Proficient = 2.997012e-08
p value ranksum for S+ high -0.49485 Proficient vs S+ low 0.50515 Proficient = 3.948115e-08
p value ranksum for S+ high -1 Proficient vs S+ low 1 Proficient = 5.135174e-08
p value ranksum for S+ high 0.50515 Proficient vs S+ low 0.50515 Proficient = 5.882462e-08
p value ranksum for S+ high -1 Proficient vs S+ high -1.4949 Naive = 6.464881e-08
p value ranksum for S+ low 1 Naive vs S+ low 0 Proficient = 8.721984e-08
p value ranksum for S+ high 0.50515 Proficient vs S+ high 0 Naive = 1.117700e-07
p value ranksum for S+ high 0.50515 Proficient vs S+ high -1.4949 Naive = 1.143254e-07
p value ranksum for S+ high 0 Proficient vs S+ low -0.49485 Naive = 1.448075e-07
p value ranksum for S+ high -0.49485 Proficient vs S+ low 0 Naive = 1.601554e-07
p value ranksum for S+ high 1 Naive vs S+ low 1 Naive = 1.810578e-07
p value t-test for S+ low 0.50515 Naive vs S+ low 0 Proficient = 2.278987e-07
p value ranksum for S+ high -1.4949 Proficient vs S+ low -1 Naive = 2.785857e-07
p value ranksum for S+ high 1 Naive vs S+ high -1.4949 Proficient = 3.282554e-07
p value ranksum for S+ high 0 Proficient vs S+ low 0 Proficient = 4.927797e-07
p value ranksum for S+ low 1 Naive vs S+ low -1 Naive = 6.611912e-07
p value ranksum for S+ high 0.50515 Proficient vs S+ low -0.49485 Naive = 7.198385e-07
p value ranksum for S+ high 0 Proficient vs S+ low 0 Naive = 8.002199e-07
p value ranksum for S+ high 1 Naive vs S+ high 0 Proficient = 1.029958e-06
p value ranksum for S+ high 0.50515 Naive vs S+ high -1.4949 Proficient = 1.108142e-06
p value ranksum for S+ high 0.50515 Naive vs S+ low 1 Naive = 1.378477e-06
p value ranksum for S+ high 0.50515 Proficient vs S+ low 0 Proficient = 1.543938e-06
p value ranksum for S+ high 0.50515 Proficient vs S+ low 0 Naive = 1.642743e-06
p value ranksum for S+ high 0 Naive vs S+ high -1 Proficient = 1.642743e-06
p value ranksum for S+ high -0.49485 Proficient vs S+ high -1 Naive = 2.816679e-06
p value ranksum for S+ high 0.50515 Proficient vs S+ low -1 Proficient = 2.860402e-06
p value ranksum for S+ low 1 Naive vs S+ low 0.50515 Proficient = 3.132094e-06
p value ranksum for S+ high -0.49485 Naive vs S+ low -1 Proficient = 3.247184e-06
p value ranksum for S+ low 1 Naive vs S+ low 0 Naive = 3.307098e-06
p value ranksum for S+ high 1 Naive vs S+ high 0.50515 Proficient = 4.116750e-06
p value ranksum for S+ high -1.4949 Proficient vs S+ low 0.50515 Proficient = 4.291661e-06
p value ranksum for S+ high 0.50515 Naive vs S+ low 0.50515 Naive = 4.774272e-06
p value ranksum for S+ high -1.4949 Proficient vs S+ low 0 Naive = 1.428685e-05
p value ranksum for S+ high -0.49485 Naive vs S+ high -1 Naive = 1.613246e-05
p value ranksum for S+ high 1 Naive vs S+ low 0.50515 Naive = 1.628576e-05
p value ranksum for S+ high 0.50515 Naive vs S+ high 0 Proficient = 2.678689e-05
p value ranksum for S+ high -0.49485 Naive vs S+ low 1 Proficient = 3.225518e-05
p value ranksum for S+ high -1 Naive vs S+ low 1 Naive = 5.288818e-05
p value ranksum for S+ high -0.49485 Proficient vs S+ low 1 Proficient = 7.515104e-05
p value ranksum for S+ high 0 Naive vs S+ high -0.49485 Proficient = 7.961020e-05
p value ranksum for S+ low 0.50515 Proficient vs S+ low 0.50515 Naive = 9.483135e-05
p value t-test for S+ low -1 Naive vs S+ low -1.4949 Naive = 9.536420e-05
p value ranksum for S+ high -0.49485 Naive vs S+ high -1.4949 Naive = 1.100734e-04
p value ranksum for S+ high 0.50515 Proficient vs S+ low -0.49485 Proficient = 1.601003e-04
p value ranksum for S+ high -0.49485 Naive vs S+ low -0.49485 Proficient = 2.106247e-04
p value ranksum for S+ high -0.49485 Proficient vs S+ high -1.4949 Naive = 2.298719e-04
p value ranksum for S+ high -1 Naive vs S+ high -1.4949 Proficient = 2.855945e-04
p value ranksum for S+ high 0 Proficient vs S+ low -1.4949 Naive = 3.117370e-04
p value ranksum for S+ high 0 Naive vs S+ high -0.49485 Naive = 3.321087e-04
p value ranksum for S+ low 1 Proficient vs S+ low 1 Naive = 3.398022e-04

p value ranksum for S+ high 0.50515 Proficient vs S+ high 0.50515 Naive = 3.510095e-04
p value ranksum for S+ high -0.49485 Naive vs S+ low -1 Naive = 4.437763e-04
p value ranksum for S+ high -1.4949 Naive vs S+ low 1 Naive = 5.506547e-04
p value ranksum for S+ high 1 Proficient vs S+ high -0.49485 Naive = 6.861646e-04
p value ranksum for S+ high -0.49485 Naive vs S+ low 0.50515 Proficient = 7.012347e-04
p value ranksum for S+ high -1 Proficient vs S+ low 0.50515 Naive = 8.212292e-04
p value ranksum for S+ high -1 Naive vs S+ low -0.49485 Naive = 1.039707e-03
p value ranksum for S+ high 0 Proficient vs S+ low -1 Proficient = 1.041312e-03
p value ranksum for S+ high -0.49485 Naive vs S+ low 0 Naive = 1.167982e-03
p value ranksum for S+ high 0 Naive vs S+ high -1.4949 Proficient = 1.228709e-03
p value ranksum for S+ high -1 Naive vs S+ low -1.4949 Naive = 1.480293e-03
p value ranksum for S+ high 0 Naive vs S+ low 1 Naive = 1.533809e-03
p value ranksum for S+ high 0.50515 Proficient vs S+ low -1.4949 Naive = 1.693276e-03
p value ranksum for S+ high -1.4949 Naive vs S+ low -1.4949 Naive = 1.920708e-03
p value ranksum for S+ high -0.49485 Naive vs S+ low -1.4949 Proficient = 2.232953e-03
p value ranksum for S+ low 1 Proficient vs S+ low -1.4949 Naive = 2.275085e-03
p value ranksum for S+ high -1 Proficient vs S+ high -1.4949 Proficient = 2.725913e-03
p value ranksum for S+ high -1.4949 Proficient vs S+ low 1 Proficient = 3.147228e-03
p value ranksum for S+ high 0 Naive vs S+ low 0.50515 Naive = 3.779160e-03
p value ranksum for S+ high 1 Proficient vs S+ high 0.50515 Proficient = 4.035025e-03
p value ranksum for S+ low 1 Proficient vs S+ low -0.49485 Naive = 5.226946e-03
p value ranksum for S+ high -1 Naive vs S+ low 0 Proficient = 5.226955e-03
p value ranksum for S+ high -0.49485 Naive vs S+ low 0 Proficient = 5.827926e-03
p value ranksum for S+ high 0.50515 Naive vs S+ high -1 Naive = 6.156626e-03
p value ranksum for S+ high -1.4949 Proficient vs S+ high -1.4949 Naive = 7.556349e-03
p value ranksum for S+ high 0 Proficient vs S+ low -0.49485 Proficient = 7.762089e-03
p value ranksum for S+ high -1.4949 Naive vs S+ low -0.49485 Naive = 9.805568e-03
p value ranksum for S+ high 0.50515 Proficient vs S+ low -1.4949 Proficient = 1.087470e-02
p value ranksum for S+ high 1 Naive vs S+ high -0.49485 Naive = 1.137122e-02
p value ranksum for S+ high -0.49485 Naive vs S+ low -0.49485 Naive = 1.307970e-02
p value ranksum for S+ high -1 Naive vs S+ low 0.50515 Naive = 1.579881e-02
p value t-test for S+ low -0.49485 Naive vs S+ low -1.4949 Naive = 1.669805e-02
p value t-test for S+ low -0.49485 Naive vs S+ low -1 Naive = 1.782808e-02
p value ranksum for S+ high 1 Proficient vs S+ low -1 Proficient = 1.807586e-02
p value ranksum for S+ low -1 Proficient vs S+ low -1.4949 Proficient = 1.831407e-02
p value ranksum for S+ high -0.49485 Proficient vs S+ high -1 Proficient = 1.879312e-02
p value ranksum for S+ low 0 Naive vs S+ low -0.49485 Naive = 2.363527e-02
p value ranksum for S+ low 1 Proficient vs S+ low 0 Proficient = 2.405631e-02
p value ranksum for S+ high 0.50515 Naive vs S+ low 1 Proficient = 2.767082e-02
p value ranksum for S+ low 1 Proficient vs S+ low 0.50515 Naive = 3.247154e-02
p value ranksum for S+ high -1 Proficient vs S+ low 1 Naive = 3.342697e-02
p value ranksum for S+ high 0.50515 Naive vs S+ high -1.4949 Naive = 3.571902e-02

p values below are > pFDR

p value ranksum for S+ high 1 Naive vs S+ low 1 Proficient = 3.866467e-02
p value ranksum for S+ high 0 Naive vs S+ low -1.4949 Naive = 3.930931e-02
p value ranksum for S+ high -0.49485 Proficient vs S+ low 0.50515 Naive = 3.970447e-02
p value ranksum for S+ high 1 Naive vs S+ high -1.4949 Naive = 4.091024e-02
p value ranksum for S+ high 1 Naive vs S+ high -1 Naive = 4.111419e-02
p value ranksum for S+ high 0.50515 Naive vs S+ high -0.49485 Naive = 4.320008e-02

p value ranksum for S+ high -1.4949 Naive vs S+ low 0 Proficient = 4.522999e-02
p value ranksum for S+ low -0.49485 Proficient vs S+ low -1.4949 Proficient = 5.698804e-02
p value ranksum for S+ high -1 Naive vs S+ low -1 Naive = 6.046064e-02
p value ranksum for S+ high -1.4949 Naive vs S+ low 0.50515 Naive = 6.482111e-02
p value ranksum for S+ low 0 Naive vs S+ low -1.4949 Naive = 6.880709e-02
p value ranksum for S+ low 0.50515 Proficient vs S+ low -1.4949 Naive = 7.805296e-02
p value ranksum for S+ high 0.50515 Naive vs S+ low -1 Naive = 7.912881e-02
p value ranksum for S+ low 1 Naive vs S+ low 0.50515 Naive = 8.162581e-02
p value ranksum for S+ high 0.50515 Naive vs S+ high 0 Naive = 9.106995e-02
p value ranksum for S+ high 0 Proficient vs S+ high -0.49485 Naive = 9.714269e-02
p value ranksum for S+ high 1 Proficient vs S+ low -0.49485 Proficient = 1.039393e-01
p value ranksum for S+ low 1 Proficient vs S+ low -1 Naive = 1.067438e-01
p value ranksum for S+ high -1 Naive vs S+ low 0.50515 Proficient = 1.185661e-01
p value ranksum for S+ high -0.49485 Naive vs S+ low -1.4949 Naive = 1.278159e-01
p value ranksum for S+ high -1.4949 Naive vs S+ low -1 Naive = 1.278184e-01
p value ranksum for S+ low 0 Proficient vs S+ low 0 Naive = 1.328270e-01
p value ranksum for S+ high 0.50515 Naive vs S+ low 0 Naive = 1.509572e-01
p value ranksum for S+ low 0.50515 Proficient vs S+ low -0.49485 Naive = 1.520787e-01
p value ranksum for S+ high 0.50515 Proficient vs S+ high 0 Proficient = 1.563073e-01
p value ranksum for S+ high 0.50515 Naive vs S+ low 0.50515 Proficient = 1.602377e-01
p value ranksum for S+ low 0 Proficient vs S+ low -1 Naive = 1.745650e-01
p value ranksum for S+ low 1 Proficient vs S+ low 0.50515 Proficient = 1.814902e-01
p value ranksum for S+ high -1.4949 Naive vs S+ low 0 Naive = 1.879845e-01
p value ranksum for S+ low 1 Proficient vs S+ low 0 Naive = 1.917232e-01
p value ranksum for S+ high 0 Proficient vs S+ low -1.4949 Proficient = 2.190389e-01
p value ranksum for S+ high -1.4949 Naive vs S+ low 0.50515 Proficient = 2.246795e-01
p value ranksum for S+ high -1.4949 Proficient vs S+ low 0.50515 Naive = 2.571933e-01
p value ranksum for S+ high 0.50515 Proficient vs S+ high -0.49485 Naive = 2.624217e-01
p value ranksum for S+ high 1 Naive vs S+ low -1.4949 Naive = 2.641850e-01
p value ranksum for S+ high 0 Naive vs S+ low -0.49485 Naive = 2.641850e-01
p value ranksum for S+ high 0 Naive vs S+ low 0 Proficient = 2.694968e-01
p value ranksum for S+ high 1 Naive vs S+ high 0 Naive = 2.704093e-01
p value ranksum for S+ high -1.4949 Proficient vs S+ low 1 Naive = 2.944040e-01
p value ranksum for S+ high -1 Naive vs S+ low 0 Naive = 3.059459e-01
p value ranksum for S+ high 1 Naive vs S+ low 0 Naive = 3.177840e-01
p value ranksum for S+ low 0.50515 Proficient vs S+ low 0 Proficient = 3.204744e-01
p value ranksum for S+ high -0.49485 Proficient vs S+ high -1.4949 Proficient = 3.238141e-01
p value ranksum for S+ high 1 Proficient vs S+ high 0 Proficient = 3.780526e-01
p value ranksum for S+ high 1 Naive vs S+ high 0.50515 Naive = 3.996129e-01
p value ranksum for S+ low 0 Naive vs S+ low -1 Naive = 4.136032e-01
p value ranksum for S+ low 0 Proficient vs S+ low -1.4949 Naive = 4.387219e-01
p value ranksum for S+ high 0 Naive vs S+ high -1 Naive = 4.723906e-01
p value ranksum for S+ high 1 Naive vs S+ low 0.50515 Proficient = 4.934086e-01
p value ranksum for S+ high 1 Naive vs S+ low -1 Naive = 5.087022e-01
p value ranksum for S+ high 0.50515 Naive vs S+ low -0.49485 Naive = 5.166693e-01
p value ranksum for S+ high 0.50515 Naive vs S+ low 0 Proficient = 5.318004e-01
p value ranksum for S+ high 1 Naive vs S+ low -0.49485 Naive = 5.968789e-01
p value ranksum for S+ low 0.50515 Proficient vs S+ low 0 Naive = 6.115592e-01
p value ranksum for S+ high -1 Naive vs S+ high -1.4949 Naive = 6.493515e-01
p value ranksum for S+ high 0 Naive vs S+ low 1 Proficient = 6.934589e-01
p value ranksum for S+ high 0.50515 Naive vs S+ low -1.4949 Naive = 7.189870e-01

p value ranksum for S+ high 0 Naive vs S+ low 0.50515 Proficient = 7.337668e-01
p value ranksum for S+ high -1 Naive vs S+ low 1 Proficient = 7.549158e-01
p value ranksum for S+ low 0 Proficient vs S+ low -0.49485 Naive = 7.591684e-01
p value ranksum for S+ low -0.49485 Proficient vs S+ low -1 Proficient = 7.708923e-01
p value ranksum for S+ high 1 Naive vs S+ low 0 Proficient = 7.920044e-01
p value ranksum for S+ high 0 Naive vs S+ high -1.4949 Naive = 8.261832e-01
p value ranksum for S+ low 0.50515 Proficient vs S+ low -1 Naive = 8.616668e-01
p value ranksum for S+ high -0.49485 Proficient vs S+ low 1 Naive = 9.088897e-01
p value ranksum for S+ high 0 Naive vs S+ low 0 Naive = 9.334464e-01
p value ranksum for S+ high 0 Naive vs S+ low -1 Naive = 9.449293e-01
p value ranksum for S+ high -1.4949 Naive vs S+ low 1 Proficient = 9.650669e-01
p value ranksum for S+ high 1 Proficient vs S+ low -1.4949 Proficient = 9.695241e-01
