# Supplementary material for: The Olfactory Bulb Facilitates Use of Category Bounds for Classification of Odorants in Different Intensity Groups
Source: Front Cell Neurosci. 2020 Dec 11;14:613635. doi: 10.3389/fncel.2020.613635 (PMC7759615; doi:10.3389/fncel.2020.613635)
Supplement: Supplementary file 5 [file Table_5.pdf]

Table S5. Generalized linear regression model for Figure 2H, peak angle variance for gamma tPAC.

PA: peak angle variance
group: S+: high vs. S+ low
perCorr: naïve vs. proficient
concentration: log10(c_{liq})

Generalized linear regression model:

PA ~ 1 + group*perCorr + group*concentration + perCorr*concentration + group:perCorr:concentration

Distribution = Normal

Estimated Coefficients:

	Estimate	SE	tStat	pValue
(Intercept)	871.5	59.567	14.63	9.4406e-47
group_2	1750.9	81.566	21.466	1.0623e-94
perCorr_2	464.45	84.241	5.5134	3.8444e-08
concentration	301.78	15.295	19.73	3.3669e-81
group_2:perCorr_2	-836.76	117.26	-7.136	1.2213e-12
group_2:concentration	-488.67	20.944	-23.332	4.2781e-110
perCorr_2:concentration	-176.36	21.631	-8.1533	5.3033e-16
group_2:perCorr_2:concentration	295.47	30.109	9.8133	2.271e-22

2784 observations, 2776 error degrees of freedom
Estimated Dispersion: 4.59e+05
F-statistic vs. constant model: 99.2, p-value = 1.1e-129

Ranksum or t-test for PA variance for theta High gamma

pFDR = 3.659420e-02

p value t-test for S+ high 1 Proficient vs S+ low 1 Proficient = 2.247304e-46
p value t-test for S+ high 1 Proficient vs S+ high -0.49485 Proficient = 1.724670e-43
p value t-test for S+ high 1 Proficient vs S+ high -1 Proficient = 2.358247e-43
p value t-test for S+ high 1 Proficient vs S+ low 0.50515 Proficient = 4.452094e-31
p value t-test for S+ high 1 Proficient vs S+ high -1.4949 Proficient = 6.652952e-30
p value t-test for S+ high 0 Proficient vs S+ low 1 Proficient = 2.358845e-29
p value t-test for S+ high 0 Proficient vs S+ high -1 Proficient = 8.720720e-27
p value t-test for S+ high 1 Proficient vs S+ low 0 Proficient = 9.363040e-27
p value t-test for S+ high 0.50515 Naive vs S+ high -1 Proficient = 9.951631e-27
p value t-test for S+ high 0.50515 Naive vs S+ low 1 Proficient = 1.282165e-26
p value t-test for S+ high 0 Proficient vs S+ high -0.49485 Proficient = 4.627742e-26
p value ranksum for S+ high 0.50515 Proficient vs S+ high -1 Proficient = 1.387053e-25

p value ranksum for S+ high -1 Proficient vs S+ low -1.4949 Proficient = 1.929504e-24
p value ranksum for S+ high 0 Naive vs S+ high -1 Proficient = 5.729679e-24
p value t-test for S+ high 1 Proficient vs S+ low 0.50515 Naive = 6.286014e-24
p value ranksum for S+ high -1 Proficient vs S+ low -1 Proficient = 1.244992e-23
p value ranksum for S+ high 1 Naive vs S+ high -1 Proficient = 1.808845e-23
p value t-test for S+ high 1 Proficient vs S+ low -0.49485 Naive = 9.194940e-23
p value t-test for S+ high 1 Proficient vs S+ high -1.4949 Naive = 1.540268e-22
p value t-test for S+ high 1 Proficient vs S+ low 1 Naive = 8.581787e-22
p value t-test for S+ high 1 Proficient vs S+ low 0 Naive = 9.350219e-22
p value ranksum for S+ high 0.50515 Proficient vs S+ low 1 Proficient = 1.339790e-21
p value ranksum for S+ high 1 Naive vs S+ low 1 Proficient = 1.465716e-21
p value t-test for S+ high 1 Proficient vs S+ low -1 Naive = 1.860316e-21
p value ranksum for S+ high -1 Proficient vs S+ low -1.4949 Naive = 5.815879e-21
p value ranksum for S+ low 1 Proficient vs S+ low -1.4949 Proficient = 1.531321e-20
p value ranksum for S+ high 0 Naive vs S+ low 1 Proficient = 1.783898e-20
p value ranksum for S+ low 1 Proficient vs S+ low -1 Proficient = 7.728966e-20
p value ranksum for S+ low 1 Proficient vs S+ low -1.4949 Naive = 2.048804e-18
p value ranksum for S+ high -1 Proficient vs S+ high -1 Naive = 2.561860e-18
p value t-test for S+ high 0.50515 Naive vs S+ high -0.49485 Proficient = 7.502933e-18
p value ranksum for S+ high -1 Proficient vs S+ low -0.49485 Proficient = 7.599923e-18
p value t-test for S+ high 1 Proficient vs S+ high -1 Naive = 9.224918e-18
p value ranksum for S+ high 1 Naive vs S+ high -1.4949 Proficient = 5.441260e-17
p value ranksum for S+ high -1 Proficient vs S+ low -0.49485 Naive = 5.836326e-17
p value ranksum for S+ high -0.49485 Naive vs S+ high -1 Proficient = 1.171027e-16
p value t-test for S+ high 0 Proficient vs S+ high -1.4949 Proficient = 1.897444e-16
p value t-test for S+ high 0 Proficient vs S+ low 0.50515 Proficient = 5.789568e-16
p value ranksum for S+ high 1 Naive vs S+ high -0.49485 Proficient = 1.271350e-15
p value ranksum for S+ high 0.50515 Proficient vs S+ high -1.4949 Proficient = 1.453399e-15
p value ranksum for S+ high -1 Proficient vs S+ low -1 Naive = 1.688994e-15
p value t-test for S+ high 1 Proficient vs S+ high -0.49485 Naive = 2.077342e-15
p value t-test for S+ high -0.49485 Proficient vs S+ low -1.4949 Proficient = 3.044253e-15
p value ranksum for S+ low 1 Proficient vs S+ low -0.49485 Proficient = 3.287052e-15
p value ranksum for S+ high -1 Naive vs S+ low 1 Proficient = 6.264199e-15
p value ranksum for S+ high 0 Naive vs S+ high -1.4949 Proficient = 6.640736e-15
p value t-test for S+ high -0.49485 Proficient vs S+ low -1.4949 Naive = 6.828670e-15
p value ranksum for S+ high -1.4949 Proficient vs S+ low -1.4949 Proficient = 1.647612e-14
p value ranksum for S+ high -1.4949 Proficient vs S+ low -1 Proficient = 2.299835e-14
p value t-test for S+ high -0.49485 Proficient vs S+ low -1 Proficient = 2.514791e-14
p value ranksum for S+ high 0.50515 Proficient vs S+ high -0.49485 Proficient = 3.837521e-14
p value t-test for S+ high 0.50515 Naive vs S+ high -1.4949 Naive = 5.620933e-14
p value t-test for S+ high 0.50515 Naive vs S+ high -1.4949 Proficient = 6.583478e-14
p value ranksum for S+ high 0 Naive vs S+ high -0.49485 Proficient = 1.347275e-13
p value ranksum for S+ high -1 Proficient vs S+ high -1.4949 Naive = 1.389810e-13
p value ranksum for S+ high -1.4949 Proficient vs S+ low -1.4949 Naive = 1.478788e-13
p value t-test for S+ high 0.50515 Naive vs S+ low 0.50515 Proficient = 7.782794e-13
p value t-test for S+ high 0 Proficient vs S+ low 0 Proficient = 1.290014e-12
p value ranksum for S+ high -1 Proficient vs S+ low 0.50515 Naive = 1.422738e-12
p value ranksum for S+ high -1 Proficient vs S+ low 0 Proficient = 1.551378e-12
p value ranksum for S+ low 1 Proficient vs S+ low -0.49485 Naive = 1.681457e-12
p value t-test for S+ high 1 Proficient vs S+ low -1.4949 Naive = 1.711953e-12
p value ranksum for S+ high 1 Naive vs S+ low 0.50515 Proficient = 2.256457e-12

p value ranksum for S+ high -0.49485 Naive vs S+ low 1 Proficient = 2.577855e-12
p value t-test for S+ high -0.49485 Proficient vs S+ low -0.49485 Proficient = 8.809233e-12
p value ranksum for S+ high -1 Proficient vs S+ low 0 Naive = 1.021386e-11
p value ranksum for S+ high 0.50515 Proficient vs S+ low 0.50515 Proficient = 1.178559e-11
p value ranksum for S+ low 1 Proficient vs S+ low -1 Naive = 1.485941e-11
p value ranksum for S+ high -1.4949 Proficient vs S+ low -0.49485 Proficient = 2.508717e-11
p value t-test for S+ high 0.50515 Naive vs S+ high -1 Naive = 3.491979e-11
p value ranksum for S+ high 0 Naive vs S+ low 0.50515 Proficient = 3.856340e-11
p value t-test for S+ high 0 Proficient vs S+ low 1 Naive = 4.076993e-11
p value t-test for S+ high 0 Proficient vs S+ low -0.49485 Naive = 5.102852e-11
p value t-test for S+ high 1 Proficient vs S+ high 0 Proficient = 7.106239e-11
p value t-test for S+ high 1 Proficient vs S+ high 0.50515 Proficient = 7.487430e-11
p value ranksum for S+ high -1 Proficient vs S+ low 0.50515 Proficient = 1.110557e-10
p value t-test for S+ high 0 Proficient vs S+ high -1.4949 Naive = 1.243583e-10
p value t-test for S+ high 0 Proficient vs S+ low 0.50515 Naive = 1.714278e-10
p value ranksum for S+ high -1 Naive vs S+ high -1.4949 Proficient = 2.043046e-10
p value t-test for S+ high 0 Proficient vs S+ low 0 Naive = 6.709277e-10
p value t-test for S+ high -1.4949 Naive vs S+ low 1 Proficient = 7.133733e-10
p value ranksum for S+ low 0.50515 Proficient vs S+ low -1.4949 Proficient = 1.082143e-09
p value t-test for S+ high 0.50515 Naive vs S+ low 1 Naive = 1.179081e-09
p value t-test for S+ high 0.50515 Naive vs S+ low 0 Proficient = 1.323530e-09
p value t-test for S+ low -0.49485 Naive vs S+ low -1.4949 Naive = 1.425246e-09
p value ranksum for S+ high 1 Naive vs S+ low 1 Naive = 1.859769e-09
p value ranksum for S+ low 0.50515 Proficient vs S+ low -1 Proficient = 2.738507e-09
p value ranksum for S+ high 1 Naive vs S+ low 0 Proficient = 3.294382e-09
p value t-test for S+ low 0 Proficient vs S+ low -1.4949 Proficient = 4.130864e-09
p value ranksum for S+ high 0 Naive vs S+ low 1 Naive = 4.320020e-09
p value t-test for S+ low 0 Proficient vs S+ low -1 Proficient = 4.663384e-09
p value ranksum for S+ high 0.50515 Proficient vs S+ low 1 Naive = 5.076539e-09
p value t-test for S+ high 0 Proficient vs S+ low -1 Naive = 6.464488e-09
p value ranksum for S+ high 0.50515 Proficient vs S+ low 0 Proficient = 7.380604e-09
p value ranksum for S+ low 1 Proficient vs S+ low 0.50515 Naive = 8.153636e-09
p value ranksum for S+ low 0.50515 Proficient vs S+ low -1.4949 Naive = 8.808667e-09
p value ranksum for S+ low 1 Proficient vs S+ low 0 Naive = 9.619929e-09
p value ranksum for S+ high 0 Naive vs S+ low 0 Proficient = 1.211062e-08
p value ranksum for S+ low 1 Proficient vs S+ low 0 Proficient = 1.321998e-08
p value t-test for S+ high 1 Proficient vs S+ high 0.50515 Naive = 1.400903e-08
p value t-test for S+ high -0.49485 Proficient vs S+ high -1 Naive = 2.057327e-08
p value t-test for S+ high 0.50515 Naive vs S+ low 0.50515 Naive = 3.123796e-08
p value t-test for S+ high 1 Proficient vs S+ low -1 Proficient = 3.275526e-08
p value ranksum for S+ high -0.49485 Naive vs S+ high -1.4949 Proficient = 4.622648e-08
p value ranksum for S+ high -1.4949 Proficient vs S+ low -0.49485 Naive = 5.895646e-08
p value t-test for S+ high 0.50515 Naive vs S+ low -0.49485 Naive = 6.310852e-08
p value t-test for S+ high -0.49485 Proficient vs S+ low -1 Naive = 8.897472e-08
p value t-test for S+ high -0.49485 Proficient vs S+ high -1 Proficient = 9.893194e-08
p value t-test for S+ low 0 Proficient vs S+ low -0.49485 Proficient = 1.301644e-07
p value ranksum for S+ high -1.4949 Proficient vs S+ low -1 Naive = 1.448075e-07
p value ranksum for S+ high -1 Proficient vs S+ low 1 Naive = 1.464399e-07
p value ranksum for S+ high 1 Naive vs S+ low 0.50515 Naive = 1.619499e-07
p value t-test for S+ high 1 Proficient vs S+ low -1.4949 Proficient = 1.884467e-07
p value ranksum for S+ low 1 Naive vs S+ low -1.4949 Proficient = 1.947410e-07

p value t-test for S+ high 0.50515 Naive vs S+ low 0 Naive = 2.105703e-07
p value ranksum for S+ high 1 Naive vs S+ high -1.4949 Naive = 2.816495e-07
p value ranksum for S+ low 1 Naive vs S+ low -1 Proficient = 3.604478e-07
p value ranksum for S+ low 0.50515 Proficient vs S+ low -0.49485 Proficient = 4.493653e-07
p value ranksum for S+ high 0 Naive vs S+ high -1.4949 Naive = 4.495942e-07
p value t-test for S+ high -0.49485 Proficient vs S+ low -0.49485 Naive = 4.737209e-07
p value t-test for S+ low 0 Naive vs S+ low -1.4949 Naive = 5.864882e-07
p value ranksum for S+ high 0.50515 Proficient vs S+ high -1.4949 Naive = 6.136672e-07
p value ranksum for S+ high 1 Naive vs S+ low 0 Naive = 7.122181e-07
p value t-test for S+ high 0.50515 Naive vs S+ low -1 Naive = 7.907678e-07
p value t-test for S+ high 0 Proficient vs S+ high -1 Naive = 7.914944e-07
p value ranksum for S+ high 1 Naive vs S+ low -0.49485 Naive = 1.029931e-06
p value ranksum for S+ high 0.50515 Proficient vs S+ low 0.50515 Naive = 1.204477e-06
p value ranksum for S+ high -1.4949 Proficient vs S+ high -1.4949 Naive = 1.576499e-06
p value ranksum for S+ low 1 Proficient vs S+ low 0.50515 Proficient = 1.584374e-06
p value t-test for S+ high -1.4949 Naive vs S+ low -1.4949 Proficient = 1.591783e-06
p value t-test for S+ high -0.49485 Proficient vs S+ high -0.49485 Naive = 2.508821e-06
p value ranksum for S+ high 0 Naive vs S+ low -0.49485 Naive = 2.705413e-06
p value ranksum for S+ high 0 Naive vs S+ low 0.50515 Naive = 2.961866e-06
p value ranksum for S+ high 0.50515 Proficient vs S+ low 0 Naive = 2.991741e-06
p value t-test for S+ high 0 Proficient vs S+ high -0.49485 Naive = 3.276380e-06
p value ranksum for S+ high 0.50515 Proficient vs S+ low -0.49485 Naive = 3.373833e-06
p value ranksum for S+ low 1 Naive vs S+ low -1.4949 Naive = 4.035933e-06
p value t-test for S+ high 0.50515 Naive vs S+ high -0.49485 Naive = 4.078445e-06
p value t-test for S+ high 1 Proficient vs S+ low -0.49485 Proficient = 4.384885e-06
p value t-test for S+ low 0 Proficient vs S+ low -1.4949 Naive = 4.519096e-06
p value ranksum for S+ high 0 Naive vs S+ low 0 Naive = 5.215781e-06
p value ranksum for S+ high -1.4949 Proficient vs S+ low 0 Naive = 5.640979e-06
p value t-test for S+ high -1.4949 Naive vs S+ low -1 Proficient = 6.795532e-06
p value t-test for S+ low -0.49485 Naive vs S+ low -1.4949 Proficient = 8.103907e-06
p value ranksum for S+ low 1 Naive vs S+ low -0.49485 Proficient = 1.193984e-05
p value ranksum for S+ high 1 Naive vs S+ low -1 Naive = 1.252300e-05
p value ranksum for S+ high -1 Naive vs S+ low 0.50515 Proficient = 1.368440e-05
p value ranksum for S+ high -1.4949 Proficient vs S+ low 0.50515 Naive = 1.455734e-05
p value t-test for S+ low 0 Naive vs S+ low -1.4949 Proficient = 1.544730e-05
p value t-test for S+ high -1.4949 Naive vs S+ low -1.4949 Naive = 1.727826e-05
p value ranksum for S+ high 1 Naive vs S+ high -1 Naive = 2.150301e-05
p value t-test for S+ high 1 Proficient vs S+ high 0 Naive = 2.221962e-05
p value ranksum for S+ low 1 Proficient vs S+ low 1 Naive = 2.302690e-05
p value ranksum for S+ high 0.50515 Proficient vs S+ low -1 Naive = 2.335626e-05
p value t-test for S+ high -1.4949 Naive vs S+ low -0.49485 Proficient = 2.629108e-05
p value ranksum for S+ high 1 Naive vs S+ high -0.49485 Naive = 2.752695e-05
p value ranksum for S+ high -1.4949 Proficient vs S+ low 0 Proficient = 2.761199e-05
p value t-test for S+ high -0.49485 Proficient vs S+ low 0 Naive = 3.002374e-05
p value t-test for S+ low -0.49485 Naive vs S+ low -1 Proficient = 3.304692e-05
p value ranksum for S+ low 0.50515 Naive vs S+ low -1.4949 Proficient = 3.527850e-05
p value t-test for S+ low 0 Naive vs S+ low -1 Proficient = 5.543098e-05
p value ranksum for S+ high 0 Naive vs S+ low -1 Naive = 5.824673e-05
p value t-test for S+ high -0.49485 Proficient vs S+ high -1.4949 Naive = 5.950143e-05
p value t-test for S+ high -0.49485 Proficient vs S+ low 0.50515 Naive = 6.655377e-05
p value ranksum for S+ low 0.50515 Naive vs S+ low -1 Proficient = 8.915392e-05

p value ranksum for S+ high 0.50515 Proficient vs S+ high -0.49485 Naive = 1.238611e-04
p value t-test for S+ low 0 Naive vs S+ low -0.49485 Proficient = 1.277298e-04
p value ranksum for S+ low 0.50515 Naive vs S+ low -1.4949 Naive = 1.756075e-04
p value ranksum for S+ high 0 Naive vs S+ high -0.49485 Naive = 2.280124e-04
p value ranksum for S+ high 0 Naive vs S+ high -1 Naive = 2.336194e-04
p value ranksum for S+ high 0.50515 Proficient vs S+ high -1 Naive = 5.591333e-04
p value ranksum for S+ low 0.50515 Naive vs S+ low -0.49485 Proficient = 6.965323e-04
p value ranksum for S+ high -1.4949 Proficient vs S+ low 1 Naive = 6.966304e-04
p value ranksum for S+ high -0.49485 Naive vs S+ low 0.50515 Proficient = 7.454118e-04
p value ranksum for S+ high -1.4949 Proficient vs S+ low 0.50515 Proficient = 9.179728e-04
p value t-test for S+ high 1 Proficient vs S+ high 1 Naive = 1.243867e-03
p value ranksum for S+ low -1 Naive vs S+ low -1.4949 Proficient = 1.352623e-03
p value t-test for S+ high 0 Proficient vs S+ low -1.4949 Naive = 1.530856e-03
p value ranksum for S+ low 0.50515 Proficient vs S+ low -0.49485 Naive = 1.558452e-03
p value t-test for S+ high -0.49485 Proficient vs S+ low 1 Proficient = 1.995686e-03
p value t-test for S+ high -0.49485 Proficient vs S+ low 0 Proficient = 2.117275e-03
p value ranksum for S+ high -1 Naive vs S+ low 1 Naive = 2.283256e-03
p value ranksum for S+ low -0.49485 Proficient vs S+ low -0.49485 Naive = 2.574203e-03
p value ranksum for S+ low -1 Proficient vs S+ low -1 Naive = 2.736865e-03
p value ranksum for S+ low 0.50515 Proficient vs S+ low -1 Naive = 3.090701e-03
p value ranksum for S+ high -0.49485 Naive vs S+ low -1.4949 Proficient = 4.904997e-03
p value ranksum for S+ low -1 Naive vs S+ low -1.4949 Naive = 5.059173e-03
p value ranksum for S+ high -0.49485 Naive vs S+ low -1 Proficient = 6.346074e-03
p value ranksum for S+ high -1 Naive vs S+ low 0 Proficient = 6.638999e-03
p value ranksum for S+ high -1 Naive vs S+ low -1.4949 Proficient = 9.052504e-03
p value ranksum for S+ low -0.49485 Proficient vs S+ low -1 Naive = 9.980090e-03
p value ranksum for S+ high -1 Naive vs S+ low -1.4949 Naive = 1.059422e-02
p value ranksum for S+ high -0.49485 Naive vs S+ low -1.4949 Naive = 1.104172e-02
p value ranksum for S+ high -0.49485 Naive vs S+ low 1 Naive = 1.709463e-02
p value ranksum for S+ high -1 Naive vs S+ low -1 Proficient = 1.727350e-02
p value ranksum for S+ high -0.49485 Naive vs S+ low -0.49485 Proficient = 2.068177e-02
p value ranksum for S+ low 1 Naive vs S+ low -1 Naive = 2.167598e-02
p value t-test for S+ high -0.49485 Proficient vs S+ low 1 Naive = 2.393630e-02
p value ranksum for S+ high -1 Naive vs S+ low -0.49485 Proficient = 2.575564e-02
p value ranksum for S+ low 1 Naive vs S+ low -0.49485 Naive = 2.891436e-02
p value ranksum for S+ high -1 Naive vs S+ low 0.50515 Naive = 3.158697e-02
p value ranksum for S+ low 0.50515 Proficient vs S+ low 0 Naive = 3.464650e-02

p values below are > pFDR

p value ranksum for S+ high -0.49485 Naive vs S+ low 0 Proficient = 3.990856e-02
p value ranksum for S+ high 1 Naive vs S+ low -1.4949 Naive = 4.384463e-02
p value t-test for S+ high -1.4949 Naive vs S+ low 0.50515 Proficient = 5.938227e-02
p value t-test for S+ high 0.50515 Naive vs S+ low -1.4949 Naive = 6.684321e-02
p value t-test for S+ low 0 Naive vs S+ low -1 Naive = 6.761284e-02
p value t-test for S+ high -0.49485 Proficient vs S+ low 0.50515 Proficient = 6.928928e-02
p value t-test for S+ low 0 Proficient vs S+ low -1 Naive = 6.937921e-02
p value ranksum for S+ high -1 Proficient vs S+ high -1.4949 Proficient = 6.943783e-02
p value ranksum for S+ high 0 Naive vs S+ low -1.4949 Naive = 7.039269e-02
p value ranksum for S+ low 0.50515 Proficient vs S+ low 0.50515 Naive = 7.166612e-02
p value ranksum for S+ high 1 Naive vs S+ low -1 Proficient = 7.587413e-02

p value t-test for S+ high 0 Proficient vs S+ low -1 Proficient = 7.651453e-02
p value ranksum for S+ high 0.50515 Proficient vs S+ low -1.4949 Naive = 7.877707e-02
p value ranksum for S+ high 1 Naive vs S+ low -0.49485 Proficient = 1.043360e-01
p value ranksum for S+ high -1 Naive vs S+ high -1.4949 Naive = 1.154157e-01
p value t-test for S+ high -1.4949 Naive vs S+ low 1 Naive = 1.248783e-01
p value ranksum for S+ high -1 Naive vs S+ low 0 Naive = 1.267894e-01
p value ranksum for S+ high 1 Naive vs S+ low -1.4949 Proficient = 1.271563e-01
p value t-test for S+ high 0.50515 Naive vs S+ high 0 Proficient = 1.372422e-01
p value ranksum for S+ high -1 Proficient vs S+ low 1 Proficient = 1.416970e-01
p value t-test for S+ high -1.4949 Naive vs S+ low -1 Naive = 1.418141e-01
p value t-test for S+ high 0 Proficient vs S+ low -1.4949 Proficient = 1.469604e-01
p value t-test for S+ high -0.49485 Proficient vs S+ high -1.4949 Proficient = 1.481728e-01
p value ranksum for S+ low 0.50515 Proficient vs S+ low 0 Proficient = 1.501058e-01
p value ranksum for S+ high -0.49485 Naive vs S+ low 0.50515 Naive = 1.575149e-01
p value ranksum for S+ low 1 Naive vs S+ low 0 Naive = 1.791520e-01
p value t-test for S+ high 0.50515 Naive vs S+ high 0 Naive = 1.895597e-01
p value ranksum for S+ low 1 Naive vs S+ low 0.50515 Naive = 1.907658e-01
p value t-test for S+ low 0 Proficient vs S+ low -0.49485 Naive = 2.031904e-01
p value ranksum for S+ high 0 Naive vs S+ low -1 Proficient = 2.067205e-01
p value t-test for S+ high 0 Proficient vs S+ low -0.49485 Proficient = 2.091538e-01
p value ranksum for S+ high -1 Naive vs S+ low -0.49485 Naive = 2.281189e-01
p value ranksum for S+ high 0.50515 Proficient vs S+ low -1 Proficient = 2.304230e-01
p value t-test for S+ low -0.49485 Naive vs S+ low -1 Naive = 2.446502e-01
p value ranksum for S+ high -0.49485 Naive vs S+ high -1.4949 Naive = 2.452684e-01
p value ranksum for S+ low 0.50515 Naive vs S+ low -1 Naive = 2.776451e-01
p value ranksum for S+ high 0 Naive vs S+ low -1.4949 Proficient = 2.897987e-01
p value ranksum for S+ high 0 Naive vs S+ low -0.49485 Proficient = 2.919270e-01
p value ranksum for S+ high 0.50515 Proficient vs S+ low -1.4949 Proficient = 3.210011e-01
p value ranksum for S+ high -0.49485 Naive vs S+ low 0 Naive = 3.329973e-01
p value ranksum for S+ low 0.50515 Naive vs S+ low -0.49485 Naive = 3.413014e-01
p value ranksum for S+ high 0.50515 Proficient vs S+ low -0.49485 Proficient = 3.499796e-01
p value t-test for S+ low 0 Proficient vs S+ low 0 Naive = 3.603780e-01
p value ranksum for S+ low 1 Naive vs S+ low 0 Proficient = 4.063900e-01
p value ranksum for S+ high -1 Naive vs S+ low -1 Naive = 4.242837e-01
p value ranksum for S+ low -1.4949 Proficient vs S+ low -1.4949 Naive = 4.656192e-01
p value ranksum for S+ high -0.49485 Naive vs S+ high -1 Naive = 4.903651e-01
p value t-test for S+ high -1.4949 Naive vs S+ low -0.49485 Naive = 5.011256e-01
p value t-test for S+ high -1.4949 Naive vs S+ low 0 Proficient = 5.035434e-01
p value ranksum for S+ high -0.49485 Naive vs S+ low -0.49485 Naive = 5.300896e-01
p value ranksum for S+ high 0.50515 Proficient vs S+ high 0 Proficient = 5.355073e-01
p value t-test for S+ high 0 Proficient vs S+ high 0 Naive = 5.497822e-01
p value ranksum for S+ high 1 Naive vs S+ high 0.50515 Proficient = 5.630061e-01
p value ranksum for S+ high 1 Naive vs S+ high 0.50515 Naive = 5.657933e-01
p value ranksum for S+ high -1.4949 Proficient vs S+ low 1 Proficient = 5.837482e-01
p value ranksum for S+ high 0.50515 Proficient vs S+ high 0.50515 Naive = 6.083720e-01
p value ranksum for S+ high 1 Naive vs S+ high 0 Naive = 6.331160e-01
p value ranksum for S+ low -0.49485 Proficient vs S+ low -1.4949 Naive = 6.379438e-01
p value ranksum for S+ low 0.50515 Naive vs S+ low 0 Proficient = 6.607388e-01
p value t-test for S+ high 0.50515 Naive vs S+ low -1 Proficient = 6.620371e-01
p value t-test for S+ low 0 Naive vs S+ low -0.49485 Naive = 6.929969e-01
p value t-test for S+ high -1.4949 Naive vs S+ low 0 Naive = 7.128194e-01

p value ranksum for S+ high -0.49485 Naive vs S+ low -1 Naive = 7.189867e-01
p value ranksum for S+ low -1 Proficient vs S+ low -1.4949 Naive = 7.422032e-01
p value ranksum for S+ low 0.50515 Naive vs S+ low 0 Naive = 7.563132e-01
p value ranksum for S+ low 1 Naive vs S+ low 0.50515 Proficient = 7.733954e-01
p value ranksum for S+ high 1 Naive vs S+ high 0 Proficient = 8.037730e-01
p value t-test for S+ high -1.4949 Naive vs S+ low 0.50515 Naive = 8.170723e-01
p value ranksum for S+ low -1 Proficient vs S+ low -1.4949 Proficient = 8.467283e-01
p value ranksum for S+ low -0.49485 Proficient vs S+ low -1.4949 Proficient = 8.626226e-01
p value ranksum for S+ high 0.50515 Proficient vs S+ high 0 Naive = 8.681661e-01
p value t-test for S+ high 0.50515 Naive vs S+ low -1.4949 Proficient = 9.168931e-01
p value t-test for S+ high 0.50515 Naive vs S+ low -0.49485 Proficient = 9.484806e-01
p value ranksum for S+ low -0.49485 Proficient vs S+ low -1 Proficient = 9.966326e-01
