# Supplementary material for: The Olfactory Bulb Facilitates Use of Category Bounds for Classification of Odorants in Different Intensity Groups
Source: Front Cell Neurosci. 2020 Dec 11;14:613635. doi: 10.3389/fncel.2020.613635 (PMC7759615; doi:10.3389/fncel.2020.613635)
Supplement: Supplementary file 6 [file Table_6.pdf]

Table S6. Generalized linear regression model for Figures 4A and B, beta tPRP.

Peak_wave: tPRP
group: S+: high vs. S+ low
perCorr: naïve vs. proficient
peak_trough: peak vs. trough
dilution: log10(c_{liq})

Generalized linear regression model:

Peak_wave ~ Peak_wave~group+perCorr+spm+peak_trough+dilution
+group*perCorr*spm*peak_trough*dilution

Distribution = Normal

Estimated Coefficients:

	Estimate	SE	tStat	pValue
(Intercept)	7.6677	0.58348	13.141	7.2449e-39
group_2	-5.5611	1.4067	-3.9533	7.8041e-05
perCorr_2	-3.542	0.82516	-4.2925	1.7968e-05
spm_2	-9.0365	1.4876	-6.0746	1.3256e-09
peak_trough_2	-0.65754	0.82516	-0.7968	0.42557
dilution	-0.61547	0.2701	-2.2787	0.022724
group_2:perCorr_2	1.1228	2.0474	0.54843	0.58342
group_2:spm_2	-8.839	2.0369	-4.3394	1.4545e-05
perCorr_2:spm_2	2.8899	2.1038	1.3737	0.1696
group_2:peak_trough_2	0.55415	1.9894	0.27856	0.7806
perCorr_2:peak_trough_2	-0.0575	1.167	-0.04927	0.9607
spm_2:peak_trough_2	0.82465	2.1038	0.39199	0.69508
group_2:dilution	0.95668	0.36985	2.5867	0.0097148
perCorr_2:dilution	0.042903	0.38197	0.11232	0.91057
spm_2:dilution	-0.73682	0.38197	-1.929	0.053784
peak_trough_2:dilution	0.061343	0.38197	0.16059	0.87242
group_2:perCorr_2:spm_2	14.587	2.9283	4.9815	6.5021e-07
group_2:perCorr_2:peak_trough_2	-0.21965	2.8954	-0.0758	0.93953
group_2:spm_2:peak_trough_2	-0.32365	2.8807	-0.1123	0.91055
perCorr_2:spm_2:peak_trough_2	0.18782	2.9752	0.0631	0.94967
group_2:perCorr_2:dilution	0.29023	0.53169	0.54587	0.58518
group_2:spm_2:dilution	4.2097	0.52304	8.0486	1.0166e-15
perCorr_2:spm_2:dilution	1.4682	0.54019	2.7179	0.0065904
group_2:peak_trough_2:dilution	-0.13914	0.52304	-0.26602	0.79024
perCorr_2:peak_trough_2:dilution	0.10784	0.54019	0.19963	0.84177
spm_2:peak_trough_2:dilution	-0.06322	0.54019	-0.11705	0.90683
group_2:perCorr_2:spm_2:peak_trough_2	-0.78542	4.1412	-0.18966	0.84958
group_2:perCorr_2:spm_2:dilution	-4.5151	0.75192	-6.0048	2.0374e-09
group_2:perCorr_2:peak_trough_2:dilution	-0.0718	0.75192	-0.0956	0.92384

group_2:spm_2:peak_trough_2:dilution	0.03304	0.73969	0.04467	0.96437
perCorr_2:spm_2:peak_trough_2:dilution	-0.181	0.76395	-0.23693	0.81272
group_2:perCorr_2:spm_2: peak_trough_2:dilution	0.28709	1.0634	0.26998	0.78718

5568 observations, 5536 error degrees of freedom
Estimated Dispersion: 16.3
F-statistic vs. constant model: 297, p-value = 0

Ranksum or t-test

pFDR = 4.365942e-02

p value ranksum for S+ low Hi1 Naive vs S+ low Low6 Naive = 1.972742e-43
p value ranksum for S+ low Hi1 Naive vs S+ low Low4 Naive = 3.151352e-43
p value ranksum for S+ low Hi1 Naive vs S+ low Low5 Naive = 4.371217e-43
p value ranksum for S+ low Hi2 Naive vs S+ low Low4 Naive = 1.312622e-41
p value ranksum for S+ low Hi2 Naive vs S+ low Low6 Naive = 8.335362e-41
p value ranksum for S+ high Hi1 Naive vs S+ low Hi1 Naive = 1.066320e-40
p value ranksum for S+ high Hi2 Naive vs S+ low Hi1 Naive = 1.066320e-40
p value ranksum for S+ low Hi1 Naive vs S+ low Low5 Proficient = 1.239052e-40
p value ranksum for S+ high Hi1 Naive vs S+ low Hi2 Naive = 1.270432e-40
p value ranksum for S+ high Hi3 Proficient vs S+ low Hi1 Naive = 1.270432e-40
p value ranksum for S+ low Hi1 Naive vs S+ low Low4 Proficient = 1.302602e-40
p value ranksum for S+ low Hi1 Naive vs S+ low Hi3 Proficient = 1.369391e-40
p value ranksum for S+ low Hi1 Naive vs S+ low Low6 Proficient = 1.369391e-40
p value ranksum for S+ high Hi3 Naive vs S+ low Hi1 Naive = 1.476013e-40
p value ranksum for S+ high Hi2 Naive vs S+ low Hi2 Naive = 1.551641e-40
p value ranksum for S+ low Hi1 Naive vs S+ low Hi2 Proficient = 1.590888e-40
p value ranksum for S+ low Hi2 Naive vs S+ low Low5 Naive = 2.109568e-40
p value ranksum for S+ high Low6 Naive vs S+ low Low6 Naive = 2.685999e-40
p value ranksum for S+ high Low6 Naive vs S+ low Low4 Naive = 3.277825e-40
p value ranksum for S+ low Hi1 Naive vs S+ low Hi1 Proficient = 4.878175e-40
p value ranksum for S+ high Low6 Naive vs S+ low Low5 Naive = 9.761324e-40
p value ranksum for S+ high Low5 Proficient vs S+ low Hi1 Naive = 2.097323e-39
p value ranksum for S+ high Low4 Naive vs S+ low Low4 Naive = 2.491546e-39
p value ranksum for S+ high Hi2 Proficient vs S+ low Hi1 Naive = 3.514435e-39
p value ranksum for S+ high Hi3 Naive vs S+ low Hi2 Naive = 4.953911e-39
p value ranksum for S+ high Low5 Naive vs S+ low Low6 Naive = 5.599179e-39
p value ranksum for S+ high Low5 Naive vs S+ low Low4 Naive = 6.025763e-39
p value ranksum for S+ high Low4 Naive vs S+ low Low6 Naive = 1.450813e-38
p value ranksum for S+ high Low5 Naive vs S+ low Low5 Naive = 2.795989e-38
p value ranksum for S+ high Hi1 Naive vs S+ high Low4 Naive = 2.984822e-38
p value ranksum for S+ high Hi1 Naive vs S+ high Low6 Naive = 2.984822e-38
p value ranksum for S+ high Hi2 Naive vs S+ high Low4 Naive = 2.984822e-38
p value ranksum for S+ high Hi2 Naive vs S+ high Low6 Naive = 2.984822e-38
p value ranksum for S+ high Low4 Naive vs S+ low Low5 Naive = 3.831167e-38
p value ranksum for S+ high Hi1 Naive vs S+ high Low5 Naive = 4.461106e-38
p value ranksum for S+ high Hi3 Naive vs S+ high Low6 Naive = 5.099475e-38
p value ranksum for S+ high Hi2 Naive vs S+ high Low5 Naive = 5.379548e-38
p value ranksum for S+ high Hi3 Proficient vs S+ high Low6 Naive = 1.048045e-37

p value ranksum for S+ high Low6 Naive vs S+ low Low5 Proficient = 1.165776e-37
p value ranksum for S+ high Low6 Naive vs S+ low Hi3 Proficient = 1.404287e-37
p value ranksum for S+ low Hi2 Naive vs S+ low Hi3 Proficient = 1.443038e-37
p value ranksum for S+ high Low6 Naive vs S+ low Low4 Proficient = 1.646934e-37
p value ranksum for S+ high Low6 Naive vs S+ low Low6 Proficient = 1.983123e-37
p value ranksum for S+ high Hi1 Proficient vs S+ low Hi1 Naive = 2.222205e-37
p value ranksum for S+ low Hi2 Naive vs S+ low Low5 Proficient = 2.565538e-37
p value ranksum for S+ high Hi3 Proficient vs S+ low Hi2 Naive = 2.961547e-37
p value ranksum for S+ high Low6 Naive vs S+ low Hi2 Proficient = 3.029763e-37
p value ranksum for S+ high Hi3 Naive vs S+ high Low5 Naive = 5.275614e-37
p value ranksum for S+ high Hi3 Naive vs S+ high Low4 Naive = 6.018695e-37
p value ranksum for S+ low Hi2 Naive vs S+ low Low4 Proficient = 8.656641e-37
p value ranksum for S+ high Hi2 Proficient vs S+ high Low6 Naive = 8.699623e-37
p value ranksum for S+ low Hi2 Naive vs S+ low Hi2 Proficient = 9.296016e-37
p value ranksum for S+ low Hi2 Naive vs S+ low Low6 Proficient = 1.206856e-36
p value ranksum for S+ high Low4 Naive vs S+ low Hi3 Proficient = 5.421192e-36
p value ranksum for S+ high Hi1 Naive vs S+ low Hi3 Naive = 5.594969e-36
p value ranksum for S+ high Low6 Naive vs S+ low Hi1 Proficient = 6.014903e-36
p value ranksum for S+ high Low4 Naive vs S+ low Hi2 Proficient = 6.673183e-36
p value ranksum for S+ high Low5 Proficient vs S+ high Low6 Naive = 7.597527e-36
p value ranksum for S+ high Low5 Naive vs S+ low Hi3 Proficient = 1.120507e-35
p value ranksum for S+ high Hi3 Proficient vs S+ high Low5 Naive = 1.414041e-35
p value ranksum for S+ high Low5 Naive vs S+ low Low5 Proficient = 1.783859e-35
p value ranksum for S+ high Low4 Naive vs S+ low Low5 Proficient = 2.430306e-35
p value ranksum for S+ high Hi3 Proficient vs S+ high Low4 Naive = 2.558696e-35
p value ranksum for S+ high Low5 Naive vs S+ low Low4 Proficient = 2.764021e-35
p value ranksum for S+ high Low5 Naive vs S+ low Low6 Proficient = 3.063464e-35
p value ranksum for S+ high Low4 Naive vs S+ low Low4 Proficient = 9.454967e-35
p value ranksum for S+ high Hi2 Naive vs S+ low Hi3 Naive = 1.099180e-34
p value ranksum for S+ high Hi1 Proficient vs S+ high Low6 Naive = 1.159495e-34
p value ranksum for S+ high Low5 Naive vs S+ low Hi2 Proficient = 1.220120e-34
p value ranksum for S+ high Low4 Naive vs S+ low Low6 Proficient = 1.495769e-34
p value ranksum for S+ high Hi1 Naive vs S+ high Low5 Proficient = 1.730310e-33
p value ranksum for S+ high Low6 Proficient vs S+ low Hi1 Naive = 4.391972e-33
p value ranksum for S+ low Hi3 Naive vs S+ low Low6 Naive = 9.981663e-33
p value ranksum for S+ low Hi3 Naive vs S+ low Low5 Naive = 1.407837e-32
p value ranksum for S+ high Hi2 Naive vs S+ high Low5 Proficient = 3.385434e-32
p value ranksum for S+ high Hi2 Proficient vs S+ high Low5 Naive = 4.542667e-32
p value ranksum for S+ high Hi3 Naive vs S+ low Hi3 Naive = 4.990485e-32
p value ranksum for S+ high Hi1 Proficient vs S+ low Hi2 Naive = 1.374787e-31
p value ranksum for S+ high Hi1 Proficient vs S+ high Low5 Naive = 1.734635e-31
p value ranksum for S+ low Hi3 Naive vs S+ low Low4 Naive = 1.900601e-31
p value ranksum for S+ high Hi1 Naive vs S+ high Low6 Proficient = 1.829356e-30
p value ranksum for S+ high Hi1 Naive vs S+ high Low4 Proficient = 3.087106e-30
p value ranksum for S+ high Low5 Naive vs S+ low Hi1 Proficient = 3.161260e-30
p value ranksum for S+ low Hi1 Naive vs S+ low Hi3 Naive = 8.619015e-30
p value ranksum for S+ low Hi3 Naive vs S+ low Low6 Proficient = 9.107785e-30
p value ranksum for S+ high Low5 Naive vs S+ high Low5 Proficient = 1.158809e-29
p value ranksum for S+ low Hi3 Naive vs S+ low Low5 Proficient = 3.530625e-29
p value ranksum for S+ high Hi1 Proficient vs S+ high Low4 Naive = 1.464462e-28
p value ranksum for S+ high Hi3 Proficient vs S+ low Hi3 Naive = 2.045844e-28

p value ranksum for S+ high Hi2 Naive vs S+ high Low6 Proficient = 3.753462e-28
p value ranksum for S+ high Hi2 Proficient vs S+ low Hi2 Naive = 4.401643e-28
p value ranksum for S+ high Hi2 Naive vs S+ high Low4 Proficient = 2.108520e-27
p value ranksum for S+ low Hi3 Naive vs S+ low Hi3 Proficient = 2.179515e-27
p value ranksum for S+ high Hi1 Naive vs S+ low Hi2 Proficient = 3.784186e-27
p value ranksum for S+ low Hi3 Naive vs S+ low Low4 Proficient = 2.451456e-26
p value ranksum for S+ low Hi1 Proficient vs S+ low Hi2 Naive = 2.875605e-26
p value ranksum for S+ high Hi3 Naive vs S+ high Low5 Proficient = 4.259156e-26
p value ranksum for S+ high Low5 Proficient vs S+ low Hi2 Naive = 2.034504e-25
p value ranksum for S+ high Low4 Naive vs S+ low Hi1 Proficient = 1.274760e-24
p value ranksum for S+ low Hi2 Proficient vs S+ low Hi3 Naive = 1.339084e-24
p value ranksum for S+ high Hi2 Naive vs S+ low Hi2 Proficient = 2.311760e-24
p value ranksum for S+ high Low5 Proficient vs S+ low Low6 Naive = 3.842128e-24
p value ranksum for S+ high Hi2 Proficient vs S+ high Low4 Naive = 5.379734e-24
p value ranksum for S+ high Low6 Naive vs S+ high Low6 Proficient = 8.716773e-24
p value ranksum for S+ high Low4 Naive vs S+ high Low5 Proficient = 1.351699e-23
p value ranksum for S+ high Low4 Proficient vs S+ low Hi1 Naive = 6.826685e-23
p value ranksum for S+ high Low5 Proficient vs S+ low Low4 Naive = 6.954443e-23
p value ranksum for S+ high Hi1 Naive vs S+ low Hi1 Proficient = 6.318275e-22
p value ranksum for S+ high Low5 Proficient vs S+ low Low5 Naive = 1.181268e-21
p value ranksum for S+ high Low5 Naive vs S+ high Low6 Proficient = 1.293552e-20
p value ranksum for S+ high Hi3 Naive vs S+ high Low6 Proficient = 1.397943e-20
p value ranksum for S+ high Hi1 Proficient vs S+ low Hi3 Naive = 1.847428e-20
p value ranksum for S+ high Low6 Proficient vs S+ low Low6 Naive = 2.485219e-20
p value ranksum for S+ high Low6 Proficient vs S+ low Hi2 Naive = 3.225902e-20
p value ranksum for S+ high Low4 Proficient vs S+ high Low6 Naive = 1.364172e-19
p value ranksum for S+ high Hi3 Naive vs S+ high Low4 Proficient = 5.665188e-19
p value ranksum for S+ high Low4 Proficient vs S+ low Hi2 Naive = 7.555451e-19
p value ranksum for S+ high Hi1 Naive vs S+ low Low4 Proficient = 1.208978e-18
p value ranksum for S+ high Low6 Proficient vs S+ low Low5 Naive = 1.471239e-18
p value ranksum for S+ high Low6 Naive vs S+ low Hi3 Naive = 2.756933e-18
p value ranksum for S+ high Low6 Proficient vs S+ low Low4 Naive = 3.413808e-18
p value ranksum for S+ high Low4 Proficient vs S+ low Low6 Naive = 5.577784e-18
p value ranksum for S+ high Low4 Proficient vs S+ high Low5 Naive = 1.106276e-17
p value ranksum for S+ high Low4 Proficient vs S+ low Low4 Naive = 4.107340e-17
p value ranksum for S+ high Low4 Naive vs S+ high Low6 Proficient = 5.441673e-17
p value ranksum for S+ high Low4 Proficient vs S+ low Low5 Naive = 5.913933e-17
p value ranksum for S+ high Low4 Naive vs S+ high Low4 Proficient = 7.453063e-17
p value ranksum for S+ low Hi1 Proficient vs S+ low Hi3 Naive = 7.493791e-17
p value ranksum for S+ high Hi1 Naive vs S+ low Hi3 Proficient = 2.374715e-16
p value ranksum for S+ high Hi3 Naive vs S+ low Hi1 Proficient = 2.374715e-16
p value ranksum for S+ high Hi2 Naive vs S+ low Hi1 Proficient = 2.677447e-16
p value ranksum for S+ high Hi1 Naive vs S+ high Hi2 Proficient = 4.621671e-16
p value ranksum for S+ high Hi3 Naive vs S+ low Hi2 Proficient = 1.866516e-15
p value ranksum for S+ high Low4 Naive vs S+ low Hi1 Naive = 2.684939e-15
p value ranksum for S+ high Hi3 Proficient vs S+ high Low5 Proficient = 2.969721e-15
p value ranksum for S+ high Hi2 Naive vs S+ low Low4 Proficient = 4.557392e-15
p value ranksum for S+ high Low5 Proficient vs S+ low Low5 Proficient = 1.378374e-14
p value ranksum for S+ high Hi2 Proficient vs S+ low Hi3 Naive = 2.234284e-14
p value ranksum for S+ high Hi1 Proficient vs S+ high Low5 Proficient = 2.577748e-14
p value ranksum for S+ low Hi2 Proficient vs S+ low Low6 Naive = 6.549601e-14

p value ranksum for S+ high Low5 Proficient vs S+ low Low6 Proficient = 9.415774e-14
p value ranksum for S+ high Hi3 Proficient vs S+ high Low6 Proficient = 9.714618e-14
p value ranksum for S+ high Low5 Naive vs S+ low Hi3 Naive = 1.637803e-13
p value ranksum for S+ high Low6 Proficient vs S+ low Low6 Proficient = 2.386645e-13
p value ranksum for S+ high Hi1 Naive vs S+ low Low4 Naive = 5.927581e-13
p value ranksum for S+ high Low5 Proficient vs S+ low Hi3 Proficient = 7.586704e-13
p value ranksum for S+ high Hi1 Naive vs S+ high Hi3 Proficient = 7.818602e-13
p value ranksum for S+ high Low6 Proficient vs S+ low Low5 Proficient = 7.818602e-13
p value ranksum for S+ high Hi3 Proficient vs S+ high Low4 Proficient = 1.071491e-12
p value ranksum for S+ high Hi1 Proficient vs S+ high Low6 Proficient = 2.808878e-12
p value ranksum for S+ high Hi1 Proficient vs S+ high Low4 Proficient = 4.485097e-12
p value ranksum for S+ low Hi1 Proficient vs S+ low Low6 Naive = 5.124389e-12
p value ranksum for S+ high Low5 Proficient vs S+ low Hi3 Naive = 8.981308e-12
p value ranksum for S+ high Low5 Proficient vs S+ low Low4 Proficient = 2.833139e-11
p value ranksum for S+ low Hi2 Proficient vs S+ low Low5 Naive = 3.072830e-11
p value ranksum for S+ high Hi2 Naive vs S+ low Hi3 Proficient = 3.213602e-11
p value ranksum for S+ low Hi1 Proficient vs S+ low Low5 Naive = 3.314923e-11
p value ranksum for S+ high Low4 Proficient vs S+ low Hi3 Naive = 3.905261e-11
p value ranksum for S+ high Low4 Proficient vs S+ low Low6 Proficient = 4.615953e-11
p value ranksum for S+ high Low6 Proficient vs S+ low Hi3 Proficient = 8.124161e-11
p value ranksum for S+ high Low4 Proficient vs S+ low Low5 Proficient = 8.819682e-11
p value ranksum for S+ high Low6 Proficient vs S+ low Hi3 Naive = 1.318792e-10
p value ranksum for S+ high Low4 Proficient vs S+ low Hi3 Proficient = 2.705661e-10
p value ranksum for S+ high Hi2 Naive vs S+ high Hi2 Proficient = 3.483082e-10
p value ranksum for S+ low Hi1 Proficient vs S+ low Low4 Naive = 4.060921e-10
p value ranksum for S+ low Hi2 Proficient vs S+ low Low4 Naive = 4.158998e-10
p value ranksum for S+ high Hi1 Naive vs S+ low Low5 Proficient = 4.909192e-10
p value ranksum for S+ high Low6 Proficient vs S+ low Low4 Proficient = 6.635011e-10
p value ranksum for S+ high Hi2 Proficient vs S+ high Hi3 Naive = 7.364182e-10
p value ranksum for S+ high Low5 Naive vs S+ low Hi1 Naive = 1.548706e-09
p value ranksum for S+ high Hi3 Naive vs S+ low Low4 Proficient = 1.658416e-09
p value ranksum for S+ low Hi1 Naive vs S+ low Hi2 Naive = 5.540510e-09
p value ranksum for S+ high Hi1 Naive vs S+ high Hi1 Proficient = 6.110286e-09
p value ranksum for S+ high Low6 Naive vs S+ low Hi1 Naive = 6.110455e-09
p value ranksum for S+ high Low4 Naive vs S+ high Low6 Naive = 7.438179e-09
p value ranksum for S+ high Low4 Proficient vs S+ low Low4 Proficient = 7.622718e-09
p value ranksum for S+ low Hi1 Proficient vs S+ low Low5 Proficient = 1.085496e-08
p value ranksum for S+ low Hi1 Proficient vs S+ low Low6 Proficient = 1.956975e-08
p value ranksum for S+ high Hi1 Naive vs S+ low Low5 Naive = 2.394862e-08
p value ranksum for S+ high Hi1 Naive vs S+ low Low6 Naive = 2.722836e-08
p value ranksum for S+ high Low5 Proficient vs S+ low Hi2 Proficient = 4.676881e-08
p value ranksum for S+ high Hi1 Naive vs S+ low Low6 Proficient = 6.464881e-08
p value ranksum for S+ low Hi1 Proficient vs S+ low Hi3 Proficient = 2.114996e-07
p value ranksum for S+ high Hi2 Naive vs S+ high Hi3 Proficient = 2.579703e-07
p value ranksum for S+ high Hi3 Naive vs S+ low Hi3 Proficient = 2.879000e-07
p value ranksum for S+ low Low4 Proficient vs S+ low Low6 Naive = 3.266617e-07
p value ranksum for S+ low Low4 Proficient vs S+ low Low5 Naive = 7.532664e-07
p value ranksum for S+ high Hi3 Proficient vs S+ low Hi1 Proficient = 7.590048e-07
p value ranksum for S+ high Hi2 Proficient vs S+ high Low5 Proficient = 1.019232e-06
p value ranksum for S+ high Hi1 Proficient vs S+ high Hi2 Naive = 1.143371e-06
p value ranksum for S+ high Low6 Proficient vs S+ low Hi2 Proficient = 1.255641e-06

p value ranksum for S+ high Hi2 Naive vs S+ low Low4 Naive = 1.446352e-06
p value ranksum for S+ low Hi2 Naive vs S+ low Hi3 Naive = 1.694759e-06
p value ranksum for S+ high Hi1 Proficient vs S+ low Hi2 Proficient = 1.747151e-06
p value ranksum for S+ high Hi3 Proficient vs S+ low Hi2 Proficient = 3.996097e-06
p value ranksum for S+ high Hi1 Proficient vs S+ low Hi1 Proficient = 4.199117e-06
p value ranksum for S+ high Hi2 Proficient vs S+ low Low6 Naive = 1.086588e-05
p value ranksum for S+ low Hi2 Proficient vs S+ low Low6 Proficient = 1.525512e-05
p value ranksum for S+ low Hi2 Proficient vs S+ low Low5 Proficient = 1.613456e-05
p value ranksum for S+ high Low4 Proficient vs S+ low Hi2 Proficient = 1.754488e-05
p value t-test for S+ low Low4 Naive vs S+ low Low6 Naive = 2.311795e-05
p value ranksum for S+ high Hi3 Naive vs S+ high Hi3 Proficient = 3.577326e-05
p value ranksum for S+ low Hi1 Proficient vs S+ low Low4 Proficient = 4.972482e-05
p value ranksum for S+ high Hi2 Proficient vs S+ low Low5 Naive = 5.066414e-05
p value ranksum for S+ high Hi2 Proficient vs S+ high Low6 Proficient = 5.927531e-05
p value ranksum for S+ low Hi3 Proficient vs S+ low Low6 Naive = 6.841760e-05
p value ranksum for S+ high Hi3 Naive vs S+ low Low5 Proficient = 1.020046e-04
p value t-test for S+ low Low4 Naive vs S+ low Low4 Proficient = 1.031456e-04
p value ranksum for S+ high Hi3 Naive vs S+ low Low4 Naive = 1.496626e-04
p value ranksum for S+ high Low4 Naive vs S+ high Low5 Naive = 2.068450e-04
p value ranksum for S+ high Low4 Naive vs S+ low Hi3 Naive = 2.233823e-04
p value ranksum for S+ high Hi1 Naive vs S+ high Hi2 Naive = 2.744035e-04
p value ranksum for S+ high Hi2 Proficient vs S+ high Low4 Proficient = 3.068231e-04
p value ranksum for S+ low Hi1 Proficient vs S+ low Hi2 Proficient = 3.767460e-04
p value ranksum for S+ low Low4 Proficient vs S+ low Low6 Proficient = 5.764683e-04
p value ranksum for S+ low Hi3 Proficient vs S+ low Low5 Naive = 1.000797e-03
p value ranksum for S+ high Hi2 Naive vs S+ low Low5 Proficient = 1.017143e-03
p value ranksum for S+ high Hi2 Proficient vs S+ low Low6 Proficient = 1.168071e-03
p value ranksum for S+ high Low6 Naive vs S+ low Hi2 Naive = 2.233077e-03
p value ranksum for S+ high Hi2 Proficient vs S+ low Low4 Naive = 2.317827e-03
p value ranksum for S+ high Hi3 Naive vs S+ low Low6 Proficient = 2.781742e-03
p value ranksum for S+ high Hi1 Proficient vs S+ high Hi3 Naive = 3.076879e-03
p value ranksum for S+ low Hi2 Proficient vs S+ low Hi3 Proficient = 4.334558e-03
p value ranksum for S+ high Hi3 Naive vs S+ low Low5 Naive = 5.441618e-03
p value ranksum for S+ high Hi1 Proficient vs S+ low Low4 Proficient = 5.495842e-03
p value ranksum for S+ high Hi3 Proficient vs S+ low Low4 Proficient = 6.677470e-03
p value ranksum for S+ high Hi3 Proficient vs S+ low Low6 Naive = 6.751916e-03
p value ranksum for S+ low Hi3 Proficient vs S+ low Low4 Naive = 7.719216e-03
p value ranksum for S+ high Hi1 Proficient vs S+ low Low6 Naive = 7.935724e-03
p value ranksum for S+ low Low4 Proficient vs S+ low Low5 Proficient = 9.290663e-03
p value ranksum for S+ high Hi2 Proficient vs S+ low Low5 Proficient = 1.171042e-02
p value t-test for S+ low Low4 Naive vs S+ low Low5 Naive = 1.470807e-02
p value ranksum for S+ high Hi2 Naive vs S+ low Low5 Naive = 1.780486e-02
p value ranksum for S+ high Hi3 Naive vs S+ low Low6 Naive = 1.919632e-02
p value ranksum for S+ high Hi1 Proficient vs S+ low Hi3 Proficient = 2.338224e-02
p value ranksum for S+ high Hi2 Naive vs S+ low Low6 Naive = 2.349513e-02
p value ranksum for S+ high Hi2 Proficient vs S+ high Hi3 Proficient = 2.937134e-02
p value ranksum for S+ high Hi2 Naive vs S+ low Low6 Proficient = 3.062045e-02
p value ranksum for S+ high Hi3 Proficient vs S+ low Low5 Naive = 3.728342e-02
p value ranksum for S+ high Hi2 Proficient vs S+ low Hi1 Proficient = 3.833599e-02
p value ranksum for S+ low Hi2 Proficient vs S+ low Low4 Proficient = 3.970447e-02

p values below are > pFDR

p value ranksum for S+ low Hi3 Proficient vs S+ low Low6 Proficient = 4.471526e-02
p value ranksum for S+ high Low4 Naive vs S+ low Hi2 Naive = 7.255143e-02
p value ranksum for S+ high Hi1 Proficient vs S+ high Hi2 Proficient = 7.948168e-02
p value ranksum for S+ high Hi2 Proficient vs S+ low Hi2 Proficient = 8.198769e-02
p value ranksum for S+ high Hi3 Proficient vs S+ low Hi3 Proficient = 1.005164e-01
p value ranksum for S+ low Hi3 Proficient vs S+ low Low5 Proficient = 1.070869e-01
p value ranksum for S+ high Hi1 Proficient vs S+ low Low5 Naive = 1.342747e-01
p value ranksum for S+ high Hi1 Naive vs S+ high Hi3 Naive = 1.515446e-01
p value ranksum for S+ low Low5 Proficient vs S+ low Low6 Naive = 1.580303e-01
p value ranksum for S+ high Low5 Naive vs S+ low Hi2 Naive = 1.698798e-01
p value ranksum for S+ low Low5 Naive vs S+ low Low5 Proficient = 2.000987e-01
p value ranksum for S+ high Hi2 Proficient vs S+ low Hi3 Proficient = 2.133538e-01
p value ranksum for S+ high Low5 Naive vs S+ high Low6 Naive = 2.345487e-01
p value ranksum for S+ high Low4 Proficient vs S+ high Low5 Proficient = 3.128154e-01
p value ranksum for S+ low Hi3 Proficient vs S+ low Low4 Proficient = 3.147967e-01
p value ranksum for S+ high Hi3 Proficient vs S+ low Low6 Proficient = 3.228039e-01
p value ranksum for S+ low Low6 Naive vs S+ low Low6 Proficient = 3.357566e-01
p value ranksum for S+ high Low6 Proficient vs S+ low Hi1 Proficient = 3.680948e-01
p value ranksum for S+ high Low5 Proficient vs S+ low Hi1 Proficient = 3.996129e-01
p value ranksum for S+ low Low5 Naive vs S+ low Low6 Naive = 4.715244e-01
p value ranksum for S+ high Hi3 Proficient vs S+ low Low4 Naive = 4.863792e-01
p value ranksum for S+ high Hi3 Proficient vs S+ low Low5 Proficient = 4.890696e-01
p value ranksum for S+ high Hi1 Proficient vs S+ low Low4 Naive = 5.136177e-01
p value ranksum for S+ high Hi2 Naive vs S+ high Hi3 Naive = 5.368663e-01
p value ranksum for S+ high Low4 Proficient vs S+ low Hi1 Proficient = 5.854854e-01
p value ranksum for S+ high Hi1 Proficient vs S+ low Low6 Proficient = 6.156052e-01
p value ranksum for S+ low Low5 Naive vs S+ low Low6 Proficient = 6.326286e-01
p value ranksum for S+ high Low4 Proficient vs S+ high Low6 Proficient = 6.627601e-01
p value ranksum for S+ high Hi1 Proficient vs S+ high Hi3 Proficient = 7.159052e-01
p value ranksum for S+ high Hi2 Proficient vs S+ low Low4 Proficient = 7.189870e-01
p value t-test for S+ low Low4 Naive vs S+ low Low5 Proficient = 7.937240e-01
p value ranksum for S+ low Low5 Proficient vs S+ low Low6 Proficient = 9.056216e-01
p value t-test for S+ low Low4 Naive vs S+ low Low6 Proficient = 9.356782e-01
p value ranksum for S+ high Hi1 Proficient vs S+ low Low5 Proficient = 9.646396e-01
p value ranksum for S+ high Low5 Proficient vs S+ high Low6 Proficient = 9.942418e-01

Ranksum or t-test for prp trough for theta Beta

pFDR = 4.275362e-02

p value ranksum for S+ low Hi1 Naive vs S+ low Low6 Naive = 2.379579e-43
p value ranksum for S+ low Hi1 Naive vs S+ low Low4 Naive = 3.225932e-43
p value ranksum for S+ low Hi1 Naive vs S+ low Low5 Naive = 4.171732e-43
p value ranksum for S+ low Hi2 Naive vs S+ low Low4 Naive = 2.435747e-41
p value ranksum for S+ low Hi2 Naive vs S+ low Low6 Naive = 5.927479e-41

p value ranksum for S+ low Hi2 Naive vs S+ low Low5 Naive = 6.203354e-41
p value ranksum for S+ high Hi1 Naive vs S+ low Hi1 Naive = 1.066320e-40
p value ranksum for S+ high Hi2 Naive vs S+ low Hi1 Naive = 1.066320e-40
p value ranksum for S+ high Hi1 Naive vs S+ low Hi2 Naive = 1.149464e-40
p value ranksum for S+ high Hi3 Naive vs S+ low Hi1 Naive = 1.239052e-40
p value ranksum for S+ high Hi3 Proficient vs S+ low Hi1 Naive = 1.239052e-40
p value ranksum for S+ low Hi1 Naive vs S+ low Low5 Proficient = 1.404052e-40
p value ranksum for S+ low Hi1 Naive vs S+ low Hi3 Proficient = 1.476013e-40
p value ranksum for S+ low Hi1 Naive vs S+ low Low6 Proficient = 1.513357e-40
p value ranksum for S+ high Hi2 Naive vs S+ low Hi2 Naive = 1.551641e-40
p value ranksum for S+ low Hi1 Naive vs S+ low Hi2 Proficient = 1.672367e-40
p value ranksum for S+ low Hi1 Naive vs S+ low Low4 Proficient = 1.847981e-40
p value ranksum for S+ high Low6 Naive vs S+ low Low6 Naive = 2.146354e-40
p value ranksum for S+ high Low6 Naive vs S+ low Low4 Naive = 2.823156e-40
p value ranksum for S+ high Low5 Proficient vs S+ low Hi1 Naive = 7.621737e-40
p value ranksum for S+ high Low6 Naive vs S+ low Low5 Naive = 9.063335e-40
p value ranksum for S+ low Hi1 Naive vs S+ low Hi1 Proficient = 1.345785e-39
p value ranksum for S+ high Hi3 Naive vs S+ low Hi2 Naive = 4.276489e-39
p value ranksum for S+ high Low4 Naive vs S+ low Low4 Naive = 6.175019e-39
p value ranksum for S+ high Low5 Naive vs S+ low Low6 Naive = 9.129647e-39
p value ranksum for S+ high Low4 Naive vs S+ low Low6 Naive = 1.381855e-38
p value ranksum for S+ high Low4 Naive vs S+ low Low5 Naive = 1.762574e-38
p value ranksum for S+ high Low5 Naive vs S+ low Low4 Naive = 2.089477e-38
p value ranksum for S+ high Hi1 Naive vs S+ high Low4 Naive = 2.984822e-38
p value ranksum for S+ high Hi1 Naive vs S+ high Low6 Naive = 2.984822e-38
p value ranksum for S+ high Hi2 Naive vs S+ high Low6 Naive = 3.065958e-38
p value ranksum for S+ high Hi2 Naive vs S+ high Low4 Naive = 3.322757e-38
p value ranksum for S+ high Low5 Naive vs S+ low Low5 Naive = 3.650078e-38
p value ranksum for S+ high Hi3 Naive vs S+ high Low6 Naive = 4.582081e-38
p value ranksum for S+ high Hi2 Proficient vs S+ low Hi1 Naive = 6.064673e-38
p value ranksum for S+ high Hi1 Naive vs S+ high Low5 Naive = 6.314844e-38
p value ranksum for S+ high Low6 Naive vs S+ low Hi3 Proficient = 9.173710e-38
p value ranksum for S+ high Hi3 Proficient vs S+ high Low6 Naive = 9.421406e-38
p value ranksum for S+ high Low6 Naive vs S+ low Low5 Proficient = 9.421406e-38
p value ranksum for S+ high Hi2 Naive vs S+ high Low5 Naive = 1.197207e-37
p value ranksum for S+ high Low6 Naive vs S+ low Low4 Proficient = 1.197207e-37
p value ranksum for S+ high Low6 Naive vs S+ low Low6 Proficient = 1.331581e-37
p value ranksum for S+ high Hi1 Naive vs S+ low Hi3 Naive = 1.748533e-37
p value ranksum for S+ high Low6 Naive vs S+ low Hi2 Proficient = 2.036419e-37
p value ranksum for S+ high Hi1 Proficient vs S+ low Hi1 Naive = 2.961547e-37
p value ranksum for S+ low Hi2 Naive vs S+ low Hi3 Proficient = 4.237726e-37
p value ranksum for S+ high Hi3 Naive vs S+ high Low5 Naive = 5.561222e-37
p value ranksum for S+ low Hi2 Naive vs S+ low Low5 Proficient = 5.916550e-37
p value ranksum for S+ high Hi3 Naive vs S+ high Low4 Naive = 6.687331e-37
p value ranksum for S+ high Hi3 Proficient vs S+ low Hi2 Naive = 7.157428e-37
p value ranksum for S+ high Low5 Proficient vs S+ high Low6 Naive = 8.039803e-37
p value ranksum for S+ high Hi2 Proficient vs S+ high Low6 Naive = 2.978556e-36
p value ranksum for S+ high Low6 Naive vs S+ low Hi1 Proficient = 4.518976e-36
p value ranksum for S+ low Hi2 Naive vs S+ low Low6 Proficient = 6.750667e-36
p value ranksum for S+ high Low4 Naive vs S+ low Hi3 Proficient = 8.212091e-36
p value ranksum for S+ high Hi3 Proficient vs S+ high Low5 Naive = 9.108987e-36

p value ranksum for S+ high Low5 Naive vs S+ low Low5 Proficient = 2.082302e-35
p value ranksum for S+ high Low5 Naive vs S+ low Low6 Proficient = 2.430306e-35
p value ranksum for S+ high Low5 Naive vs S+ low Hi3 Proficient = 2.625395e-35
p value ranksum for S+ low Hi2 Naive vs S+ low Hi2 Proficient = 3.539075e-35
p value ranksum for S+ low Hi2 Naive vs S+ low Low4 Proficient = 5.497459e-35
p value ranksum for S+ high Hi3 Proficient vs S+ high Low4 Naive = 5.968441e-35
p value ranksum for S+ high Hi2 Naive vs S+ low Hi3 Naive = 6.171729e-35
p value ranksum for S+ high Low4 Naive vs S+ low Hi2 Proficient = 7.513370e-35
p value ranksum for S+ high Low4 Naive vs S+ low Low5 Proficient = 7.707860e-35
p value ranksum for S+ high Low5 Naive vs S+ low Low4 Proficient = 1.047078e-34
p value ranksum for S+ high Hi1 Proficient vs S+ high Low6 Naive = 1.074124e-34
p value ranksum for S+ high Hi1 Naive vs S+ high Low5 Proficient = 1.573844e-34
p value ranksum for S+ high Hi1 Naive vs S+ high Low4 Proficient = 2.549874e-34
p value ranksum for S+ high Low5 Naive vs S+ low Hi2 Proficient = 3.202959e-34
p value ranksum for S+ high Low4 Naive vs S+ low Low6 Proficient = 3.921532e-34
p value ranksum for S+ high Low4 Naive vs S+ low Low4 Proficient = 9.011972e-34
p value ranksum for S+ high Hi1 Naive vs S+ high Low6 Proficient = 5.449001e-33
p value ranksum for S+ high Hi3 Naive vs S+ low Hi3 Naive = 1.100943e-32
p value ranksum for S+ high Low6 Proficient vs S+ low Hi1 Naive = 1.922584e-32
p value ranksum for S+ low Hi3 Naive vs S+ low Low5 Naive = 2.850478e-32
p value ranksum for S+ low Hi3 Naive vs S+ low Low6 Naive = 4.900359e-32
p value ranksum for S+ low Hi3 Naive vs S+ low Low4 Naive = 8.576437e-32
p value ranksum for S+ high Hi1 Proficient vs S+ low Hi2 Naive = 1.231836e-31
p value ranksum for S+ high Hi2 Proficient vs S+ high Low5 Naive = 1.296349e-31
p value ranksum for S+ high Hi1 Proficient vs S+ high Low5 Naive = 1.820806e-31
p value ranksum for S+ high Hi2 Naive vs S+ high Low5 Proficient = 2.105648e-31
p value ranksum for S+ high Hi2 Naive vs S+ high Low4 Proficient = 3.851983e-31
p value ranksum for S+ high Low5 Naive vs S+ low Hi1 Proficient = 9.598063e-31
p value ranksum for S+ high Low5 Naive vs S+ high Low5 Proficient = 2.377036e-30
p value ranksum for S+ high Hi1 Naive vs S+ low Hi2 Proficient = 3.314912e-30
p value ranksum for S+ high Hi2 Naive vs S+ high Low6 Proficient = 2.693226e-29
p value ranksum for S+ high Hi1 Proficient vs S+ high Low4 Naive = 4.825849e-28
p value ranksum for S+ low Hi3 Naive vs S+ low Low6 Proficient = 5.996897e-28
p value ranksum for S+ low Hi1 Naive vs S+ low Hi3 Naive = 7.981841e-28
p value ranksum for S+ low Hi3 Naive vs S+ low Low5 Proficient = 9.046599e-28
p value ranksum for S+ high Hi2 Proficient vs S+ low Hi2 Naive = 1.088032e-27
p value ranksum for S+ low Hi3 Naive vs S+ low Hi3 Proficient = 1.928595e-27
p value ranksum for S+ high Hi3 Proficient vs S+ low Hi3 Naive = 5.543881e-27
p value ranksum for S+ high Hi3 Naive vs S+ high Low5 Proficient = 1.130550e-26
p value ranksum for S+ high Low4 Naive vs S+ high Low5 Proficient = 1.130550e-26
p value ranksum for S+ high Low5 Proficient vs S+ low Hi2 Naive = 1.318144e-26
p value ranksum for S+ high Hi1 Naive vs S+ low Low4 Proficient = 2.455334e-26
p value ranksum for S+ low Hi1 Proficient vs S+ low Hi2 Naive = 1.773610e-25
p value ranksum for S+ high Hi2 Naive vs S+ low Hi2 Proficient = 3.445632e-25
p value ranksum for S+ low Hi2 Proficient vs S+ low Hi3 Naive = 2.572307e-24
p value ranksum for S+ high Low4 Naive vs S+ low Hi1 Proficient = 5.610801e-24
p value ranksum for S+ high Low5 Proficient vs S+ low Low6 Naive = 7.910507e-24
p value ranksum for S+ high Hi3 Naive vs S+ high Low4 Proficient = 8.716773e-24
p value ranksum for S+ low Hi3 Naive vs S+ low Low4 Proficient = 1.070569e-23
p value ranksum for S+ high Hi2 Proficient vs S+ high Low4 Naive = 1.771819e-23
p value ranksum for S+ high Low4 Proficient vs S+ low Hi1 Naive = 1.955633e-23

p value ranksum for S+ high Hi1 Naive vs S+ low Hi1 Proficient = 2.092173e-23
p value ranksum for S+ high Low5 Proficient vs S+ low Low4 Naive = 4.056323e-23
p value ranksum for S+ high Low5 Proficient vs S+ low Low5 Naive = 4.370197e-23
p value ranksum for S+ high Hi3 Naive vs S+ high Low6 Proficient = 4.496757e-23
p value ranksum for S+ high Low6 Naive vs S+ high Low6 Proficient = 5.715149e-22
p value ranksum for S+ high Hi1 Naive vs S+ high Hi2 Proficient = 1.646476e-21
p value ranksum for S+ high Hi1 Proficient vs S+ low Hi3 Naive = 3.642532e-20
p value ranksum for S+ high Low5 Naive vs S+ high Low6 Proficient = 4.795199e-20
p value ranksum for S+ high Low4 Proficient vs S+ high Low6 Naive = 8.024171e-20
p value ranksum for S+ high Hi2 Naive vs S+ low Low4 Proficient = 1.776507e-19
p value ranksum for S+ high Low4 Proficient vs S+ low Hi2 Naive = 2.170405e-19
p value ranksum for S+ high Low6 Proficient vs S+ low Low6 Naive = 4.342015e-19
p value ranksum for S+ high Hi1 Naive vs S+ low Hi3 Proficient = 6.101924e-19
p value ranksum for S+ high Low4 Proficient vs S+ low Low6 Naive = 1.572151e-18
p value ranksum for S+ high Low4 Naive vs S+ high Low4 Proficient = 2.561860e-18
p value ranksum for S+ high Hi3 Naive vs S+ low Hi1 Proficient = 4.418245e-18
p value ranksum for S+ high Low6 Proficient vs S+ low Low5 Naive = 4.975419e-18
p value ranksum for S+ high Low4 Proficient vs S+ low Low5 Naive = 5.057366e-18
p value ranksum for S+ high Low6 Proficient vs S+ low Low4 Naive = 5.669516e-18
p value ranksum for S+ high Low6 Naive vs S+ low Hi3 Naive = 6.565048e-18
p value ranksum for S+ high Hi3 Naive vs S+ low Hi2 Proficient = 6.938111e-18
p value ranksum for S+ high Low6 Proficient vs S+ low Hi2 Naive = 7.121479e-18
p value ranksum for S+ high Hi3 Proficient vs S+ high Low5 Proficient = 9.084251e-18
p value ranksum for S+ high Hi2 Naive vs S+ low Hi1 Proficient = 1.029843e-17
p value ranksum for S+ high Low4 Proficient vs S+ high Low5 Naive = 1.231537e-17
p value ranksum for S+ high Low4 Naive vs S+ high Low6 Proficient = 2.691893e-17
p value ranksum for S+ high Low4 Proficient vs S+ low Low4 Naive = 4.968879e-17
p value ranksum for S+ high Hi1 Naive vs S+ low Low4 Naive = 1.947186e-16
p value ranksum for S+ high Low5 Proficient vs S+ low Low5 Proficient = 9.400145e-16
p value ranksum for S+ high Hi1 Naive vs S+ high Hi3 Proficient = 2.132069e-15
p value ranksum for S+ high Low4 Naive vs S+ low Hi1 Naive = 9.598552e-15
p value ranksum for S+ high Hi1 Proficient vs S+ high Low5 Proficient = 1.030278e-14
p value ranksum for S+ low Hi1 Proficient vs S+ low Low5 Naive = 1.343728e-14
p value ranksum for S+ low Hi1 Proficient vs S+ low Low6 Naive = 1.671761e-14
p value ranksum for S+ high Hi3 Naive vs S+ low Low4 Proficient = 3.171397e-14
p value ranksum for S+ high Hi2 Proficient vs S+ low Hi3 Naive = 5.215218e-14
p value ranksum for S+ low Hi1 Proficient vs S+ low Hi3 Naive = 5.680943e-14
p value ranksum for S+ high Hi3 Proficient vs S+ high Low6 Proficient = 1.208399e-13
p value ranksum for S+ low Hi2 Proficient vs S+ low Low6 Naive = 3.149023e-13
p value ranksum for S+ high Low5 Proficient vs S+ low Hi3 Naive = 7.892997e-13
p value ranksum for S+ high Low5 Naive vs S+ low Hi3 Naive = 9.041353e-13
p value ranksum for S+ high Low6 Proficient vs S+ low Low6 Proficient = 1.071491e-12
p value ranksum for S+ high Low6 Proficient vs S+ low Low5 Proficient = 1.444068e-12
p value ranksum for S+ high Hi1 Proficient vs S+ high Low4 Proficient = 1.602418e-12
p value ranksum for S+ high Hi3 Proficient vs S+ high Low4 Proficient = 1.626387e-12
p value ranksum for S+ high Hi2 Naive vs S+ high Hi2 Proficient = 1.777768e-12
p value ranksum for S+ high Low5 Proficient vs S+ low Low6 Proficient = 2.424600e-12
p value ranksum for S+ high Hi2 Proficient vs S+ high Hi3 Naive = 2.648485e-12
p value ranksum for S+ low Hi2 Proficient vs S+ low Low5 Naive = 5.690997e-12
p value ranksum for S+ low Hi1 Proficient vs S+ low Low4 Naive = 6.155819e-12
p value ranksum for S+ high Hi1 Naive vs S+ low Low5 Proficient = 8.476094e-12

p value ranksum for S+ high Hi1 Naive vs S+ low Low6 Naive = 1.035718e-11
p value ranksum for S+ high Low5 Proficient vs S+ low Hi3 Proficient = 1.883375e-11
p value ranksum for S+ low Hi2 Proficient vs S+ low Low4 Naive = 2.237364e-11
p value ranksum for S+ high Hi1 Naive vs S+ high Hi1 Proficient = 2.427636e-11
p value ranksum for S+ high Hi1 Naive vs S+ low Low5 Naive = 2.812206e-11
p value ranksum for S+ high Hi1 Naive vs S+ low Low6 Proficient = 2.954783e-11
p value ranksum for S+ high Hi2 Naive vs S+ low Hi3 Proficient = 4.306379e-11
p value ranksum for S+ high Hi1 Proficient vs S+ high Low6 Proficient = 5.016267e-11
p value ranksum for S+ high Low4 Proficient vs S+ low Hi3 Naive = 5.211539e-11
p value ranksum for S+ high Low4 Proficient vs S+ low Low5 Proficient = 7.180339e-11
p value ranksum for S+ high Low6 Proficient vs S+ low Hi3 Proficient = 2.565060e-10
p value ranksum for S+ high Low4 Proficient vs S+ low Low6 Proficient = 3.347245e-10
p value ranksum for S+ high Low5 Proficient vs S+ low Low4 Proficient = 3.771093e-10
p value ranksum for S+ high Low4 Naive vs S+ high Low6 Naive = 4.974142e-10
p value ranksum for S+ low Hi1 Proficient vs S+ low Low5 Proficient = 4.974142e-10
p value ranksum for S+ high Low6 Proficient vs S+ low Hi3 Naive = 7.789128e-10
p value ranksum for S+ high Low5 Naive vs S+ low Hi1 Naive = 1.186376e-09
p value ranksum for S+ high Hi3 Naive vs S+ low Hi3 Proficient = 1.813117e-09
p value ranksum for S+ high Low4 Proficient vs S+ low Hi3 Proficient = 3.494535e-09
p value ranksum for S+ high Hi3 Proficient vs S+ low Hi1 Proficient = 6.660134e-09
p value ranksum for S+ low Hi1 Naive vs S+ low Hi2 Naive = 1.309006e-08
p value ranksum for S+ low Hi1 Proficient vs S+ low Low6 Proficient = 2.633378e-08
p value ranksum for S+ low Low4 Proficient vs S+ low Low6 Naive = 4.387012e-08
p value ranksum for S+ low Hi1 Proficient vs S+ low Hi3 Proficient = 5.313264e-08
p value ranksum for S+ high Low6 Naive vs S+ low Hi1 Naive = 6.528014e-08
p value ranksum for S+ high Low6 Proficient vs S+ low Low4 Proficient = 6.691783e-08
p value ranksum for S+ high Hi3 Naive vs S+ low Low4 Naive = 1.395889e-07
p value ranksum for S+ low Low4 Proficient vs S+ low Low5 Naive = 1.439007e-07
p value ranksum for S+ high Hi2 Naive vs S+ high Hi3 Proficient = 2.260098e-07
p value ranksum for S+ high Hi1 Proficient vs S+ high Hi2 Naive = 3.906353e-07
p value ranksum for S+ high Hi3 Proficient vs S+ low Hi2 Proficient = 4.168583e-07
p value ranksum for S+ high Low4 Proficient vs S+ low Low4 Proficient = 1.051737e-06
p value ranksum for S+ high Hi2 Proficient vs S+ high Low5 Proficient = 1.528508e-06
p value ranksum for S+ low Hi2 Naive vs S+ low Hi3 Naive = 1.723489e-06
p value ranksum for S+ high Hi1 Proficient vs S+ low Hi1 Proficient = 2.121531e-06
p value ranksum for S+ high Hi2 Naive vs S+ low Low4 Naive = 2.174328e-06
p value ranksum for S+ low Hi2 Proficient vs S+ low Low5 Proficient = 2.301229e-06
p value ranksum for S+ high Hi3 Naive vs S+ high Hi3 Proficient = 2.470375e-06
p value ranksum for S+ high Hi3 Naive vs S+ low Low6 Proficient = 3.021979e-06
p value ranksum for S+ low Low4 Naive vs S+ low Low4 Proficient = 3.718052e-06
p value ranksum for S+ high Hi1 Naive vs S+ high Hi2 Naive = 3.956637e-06
p value ranksum for S+ high Hi1 Proficient vs S+ low Hi2 Proficient = 5.696430e-06
p value ranksum for S+ low Hi2 Proficient vs S+ low Low6 Proficient = 5.865829e-06
p value ranksum for S+ high Hi3 Naive vs S+ low Low5 Proficient = 6.466007e-06
p value ranksum for S+ high Hi2 Proficient vs S+ high Low4 Proficient = 9.239068e-06
p value ranksum for S+ high Low6 Proficient vs S+ low Hi2 Proficient = 1.161089e-05
p value ranksum for S+ high Low4 Naive vs S+ high Low5 Naive = 2.467523e-05
p value ranksum for S+ high Hi2 Proficient vs S+ low Low6 Naive = 3.673849e-05
p value ranksum for S+ low Hi1 Proficient vs S+ low Low4 Proficient = 4.239358e-05
p value ranksum for S+ high Hi1 Proficient vs S+ high Hi3 Naive = 5.288818e-05
p value ranksum for S+ high Hi2 Proficient vs S+ high Low6 Proficient = 5.288818e-05

p value ranksum for S+ high Low5 Proficient vs S+ low Hi2 Proficient = 7.625333e-05
p value ranksum for S+ high Hi2 Proficient vs S+ low Low5 Naive = 1.166176e-04
p value ranksum for S+ high Hi2 Naive vs S+ low Low5 Proficient = 1.167833e-04
p value ranksum for S+ high Hi3 Naive vs S+ low Low6 Naive = 1.542199e-04
p value ranksum for S+ high Hi3 Naive vs S+ low Low5 Naive = 1.750856e-04
p value ranksum for S+ high Low4 Naive vs S+ low Hi3 Naive = 1.790329e-04
p value ranksum for S+ high Hi2 Naive vs S+ low Low6 Proficient = 2.810677e-04
p value ranksum for S+ low Hi1 Proficient vs S+ low Hi2 Proficient = 2.925128e-04
p value ranksum for S+ low Hi3 Proficient vs S+ low Low6 Naive = 3.622985e-04
p value ranksum for S+ high Hi3 Proficient vs S+ low Low4 Proficient = 9.245053e-04
p value ranksum for S+ low Hi3 Proficient vs S+ low Low5 Naive = 1.069159e-03
p value ranksum for S+ low Hi2 Proficient vs S+ low Hi3 Proficient = 1.086231e-03
p value ranksum for S+ high Low4 Proficient vs S+ low Hi2 Proficient = 1.118309e-03
p value ranksum for S+ low Low4 Proficient vs S+ low Low6 Proficient = 1.211094e-03
p value ranksum for S+ high Hi2 Naive vs S+ low Low5 Naive = 1.433592e-03
p value ranksum for S+ high Hi2 Proficient vs S+ low Low4 Naive = 1.792779e-03
p value ranksum for S+ low Low4 Proficient vs S+ low Low5 Proficient = 2.346766e-03
p value ranksum for S+ high Hi2 Naive vs S+ low Low6 Naive = 2.496228e-03
p value ranksum for S+ high Low6 Naive vs S+ low Hi2 Naive = 3.072063e-03
p value ranksum for S+ high Hi1 Proficient vs S+ low Low4 Proficient = 5.601323e-03
p value ranksum for S+ high Hi2 Proficient vs S+ low Low6 Proficient = 6.513141e-03
p value ranksum for S+ low Hi3 Proficient vs S+ low Low4 Naive = 7.719216e-03
p value ranksum for S+ high Hi2 Proficient vs S+ low Low5 Proficient = 7.984648e-03
p value ranksum for S+ high Hi2 Proficient vs S+ low Hi1 Proficient = 1.953425e-02
p value ranksum for S+ high Hi2 Proficient vs S+ high Hi3 Proficient = 2.132479e-02
p value ranksum for S+ low Hi2 Proficient vs S+ low Low4 Proficient = 2.414846e-02
p value ranksum for S+ high Hi2 Proficient vs S+ low Hi2 Proficient = 3.700781e-02

p values below are > pFDR

p value ranksum for S+ high Low4 Naive vs S+ low Hi2 Naive = 4.483102e-02
p value ranksum for S+ high Hi3 Proficient vs S+ low Low6 Naive = 4.727005e-02
p value ranksum for S+ high Hi1 Proficient vs S+ low Low6 Naive = 5.047486e-02
p value ranksum for S+ high Hi3 Proficient vs S+ low Hi3 Proficient = 5.502755e-02
p value ranksum for S+ high Hi1 Proficient vs S+ low Hi3 Proficient = 6.275496e-02
p value ranksum for S+ low Hi3 Proficient vs S+ low Low5 Proficient = 7.332270e-02
p value ranksum for S+ high Hi3 Proficient vs S+ low Low5 Naive = 8.388961e-02
p value ranksum for S+ high Hi1 Proficient vs S+ high Hi2 Proficient = 8.795666e-02
p value ranksum for S+ low Low4 Naive vs S+ low Low6 Naive = 9.483309e-02
p value ranksum for S+ low Low5 Naive vs S+ low Low6 Proficient = 1.067439e-01
p value ranksum for S+ low Low6 Naive vs S+ low Low6 Proficient = 1.087836e-01
p value ranksum for S+ high Hi1 Proficient vs S+ low Low5 Naive = 1.129554e-01
p value ranksum for S+ high Hi1 Naive vs S+ high Hi3 Naive = 1.173340e-01
p value ranksum for S+ low Low5 Proficient vs S+ low Low6 Naive = 1.198915e-01
p value ranksum for S+ low Low4 Naive vs S+ low Low5 Naive = 1.214085e-01
p value ranksum for S+ low Hi3 Proficient vs S+ low Low6 Proficient = 1.237416e-01
p value ranksum for S+ high Low5 Naive vs S+ high Low6 Naive = 1.304190e-01
p value ranksum for S+ high Low5 Naive vs S+ low Hi2 Naive = 1.328270e-01
p value ranksum for S+ low Hi3 Proficient vs S+ low Low4 Proficient = 1.599531e-01
p value ranksum for S+ low Low5 Naive vs S+ low Low5 Proficient = 1.624684e-01
p value ranksum for S+ high Hi2 Naive vs S+ high Hi3 Naive = 1.778211e-01

p value ranksum for S+ high Low4 Proficient vs S+ high Low5 Proficient = 2.677295e-01
p value ranksum for S+ high Hi1 Proficient vs S+ low Low4 Naive = 3.102173e-01
p value ranksum for S+ high Low4 Proficient vs S+ low Hi1 Proficient = 3.593819e-01
p value ranksum for S+ high Hi3 Proficient vs S+ low Low4 Naive = 3.715210e-01
p value ranksum for S+ high Hi2 Proficient vs S+ low Hi3 Proficient = 4.375542e-01
p value ranksum for S+ high Low6 Proficient vs S+ low Hi1 Proficient = 5.233577e-01
p value ranksum for S+ low Low5 Proficient vs S+ low Low6 Proficient = 5.314419e-01
p value ranksum for S+ high Low4 Proficient vs S+ high Low6 Proficient = 5.491723e-01
p value ranksum for S+ high Hi1 Proficient vs S+ high Hi3 Proficient = 6.449068e-01
p value ranksum for S+ high Hi2 Proficient vs S+ low Low4 Proficient = 6.508358e-01
p value ranksum for S+ high Hi1 Proficient vs S+ low Low6 Proficient = 7.406857e-01
p value ranksum for S+ low Low4 Naive vs S+ low Low5 Proficient = 7.805379e-01
p value ranksum for S+ high Hi1 Proficient vs S+ low Low5 Proficient = 8.149593e-01
p value ranksum for S+ high Hi3 Proficient vs S+ low Low5 Proficient = 8.293967e-01
p value ranksum for S+ low Low4 Naive vs S+ low Low6 Proficient = 8.689956e-01
p value ranksum for S+ low Low5 Naive vs S+ low Low6 Naive = 9.039258e-01
p value ranksum for S+ high Low5 Proficient vs S+ high Low6 Proficient = 9.105244e-01
p value ranksum for S+ high Hi3 Proficient vs S+ low Low6 Proficient = 9.613527e-01
p value ranksum for S+ high Low5 Proficient vs S+ low Hi1 Proficient = 9.646396e-01
