# Supplementary material for: The Olfactory Bulb Facilitates Use of Category Bounds for Classification of Odorants in Different Intensity Groups
Source: Front Cell Neurosci. 2020 Dec 11;14:613635. doi: 10.3389/fncel.2020.613635 (PMC7759615; doi:10.3389/fncel.2020.613635)
Supplement: Supplementary file 7 [file Table_7.pdf]

Table S7. Generalized linear regression model for Figures 4C and D, gamma tPRP.

Peak_wave: tPRP
group: S+: high vs. S+ low
perCorr: naïve vs. proficient
peak_trough: peak vs. trough
dilution: log10(c_{liq})

Generalized linear regression model:

Peak_wave ~ Peak_wave~group+perCorr+spm+peak_trough+dilution
+group*perCorr*spm*peak_trough*dilution

Distribution = Normal

Estimated Coefficients:

	Estimate	SE	tStat	pValue
(Intercept)	12.719	0.5616	22.645	1.1777e-108
group_2	-9.1331	1.3541	-6.7448	1.6888e-11
perCorr_2	-5.4576	0.7943	-6.8709	7.0798e-12
spm_2	-12.01	1.4319	-8.3872	6.2548e-17
peak_trough_2	-13.005	0.7943	-16.373	7.0368e-59
dilution	-0.8275	0.26	-3.1831	0.0014653
group_2:perCorr_2	2.0124	1.9708	1.0211	0.30724
group_2:spm_2	-3.0023	1.9608	-1.5312	0.12578
perCorr_2:spm_2	5.8814	2.0251	2.9043	0.0036958
group_2:peak_trough_2	8.2054	1.915	4.2848	1.8598e-05
perCorr_2:peak_trough_2	4.0311	1.1233	3.5886	0.00033533
spm_2:peak_trough_2	10.908	2.0251	5.3866	7.4767e-08
group_2:dilution	1.5729	0.35601	4.4182	1.0143e-05
perCorr_2:dilution	0.19082	0.36769	0.51896	0.60381
spm_2:dilution	-0.3607	0.36769	-0.9812	0.32653
peak_trough_2:dilution	0.62758	0.36769	1.7068	0.087914
group_2:perCorr_2:spm_2	9.5506	2.8188	3.3882	0.00070844
group_2:perCorr_2:peak_trough_2	-1.3384	2.7871	-0.4802	0.63109
group_2:spm_2:peak_trough_2	-1.4374	2.773	-0.5183	0.60423
perCorr_2:spm_2:peak_trough_2	-4.4494	2.8639	-1.5536	0.12033
group_2:perCorr_2:dilution	0.03009	0.5118	0.05880	0.95311
group_2:spm_2:dilution	2.6593	0.50348	5.2819	1.3275e-07
perCorr_2:spm_2:dilution	0.96669	0.51999	1.859	0.063074
group_2:peak_trough_2:dilution	-1.3305	0.50348	-2.6427	0.0082479
perCorr_2:peak_trough_2:dilution	0.2644	0.51999	0.50847	0.61114
spm_2:peak_trough_2:dilution	0.20027	0.51999	0.38514	0.70015
group_2:perCorr_2:spm_2:peak_trough_2	-5.1676	3.9864	-1.2963	0.19492
group_2:perCorr_2:spm_2:dilution	-3.0039	0.7238	-4.1501	3.3729e-05
group_2:perCorr_2:peak_trough_2:dilution	-0.3490	0.7238	-0.4822	0.62963
group_2:spm_2:peak_trough_2:dilution	-1.0593	0.7120	-1.4878	0.13687

perCorr_2:spm_2:peak_trough_2:dilution	-1.0909	0.7353	-1.4834	0.13802
group_2:perCorr_2:spm_2:peak_trough_2:dilution	1.812	1.0236	1.7702	0.076742

5568 observations, 5536 error degrees of freedom
Estimated Dispersion: 15.1
F-statistic vs. constant model: 255, p-value = 0

Ranksum or t-test for prp peak for theta High gamma

pFDR = 4.347826e-02

p value ranksum for S+ low Hi1 Naive vs S+ low Low4 Naive = 2.225921e-42
p value ranksum for S+ low Hi1 Naive vs S+ low Low6 Naive = 3.534884e-42
p value ranksum for S+ low Hi1 Naive vs S+ low Low5 Naive = 2.991783e-41
p value ranksum for S+ high Hi1 Naive vs S+ low Hi1 Naive = 1.066320e-40
p value ranksum for S+ high Hi1 Naive vs S+ low Hi2 Naive = 1.066320e-40
p value ranksum for S+ high Hi2 Naive vs S+ low Hi1 Naive = 1.066320e-40
p value ranksum for S+ high Hi2 Naive vs S+ low Hi2 Naive = 1.093348e-40
p value ranksum for S+ high Hi3 Naive vs S+ low Hi1 Naive = 1.590888e-40
p value ranksum for S+ high Hi3 Naive vs S+ low Hi2 Naive = 1.280960e-39
p value ranksum for S+ low Hi1 Naive vs S+ low Hi2 Proficient = 1.413870e-39
p value ranksum for S+ low Hi1 Naive vs S+ low Hi3 Proficient = 1.413870e-39
p value ranksum for S+ low Hi2 Naive vs S+ low Low4 Naive = 1.741612e-39
p value ranksum for S+ high Low6 Naive vs S+ low Low4 Naive = 4.953911e-39
p value ranksum for S+ high Hi3 Proficient vs S+ low Hi1 Naive = 5.331610e-39
p value ranksum for S+ low Hi2 Naive vs S+ low Low6 Naive = 6.468570e-39
p value ranksum for S+ low Hi1 Naive vs S+ low Low5 Proficient = 8.909467e-39
p value ranksum for S+ high Low6 Naive vs S+ low Low6 Naive = 1.284481e-38
p value ranksum for S+ low Hi1 Naive vs S+ low Low6 Proficient = 2.599748e-38
p value ranksum for S+ high Hi1 Naive vs S+ high Low4 Naive = 2.984822e-38
p value ranksum for S+ high Hi1 Naive vs S+ high Low6 Naive = 2.984822e-38
p value ranksum for S+ high Hi2 Naive vs S+ high Low4 Naive = 2.984822e-38
p value ranksum for S+ high Hi2 Naive vs S+ high Low6 Naive = 2.984822e-38
p value ranksum for S+ high Hi1 Naive vs S+ high Low5 Naive = 3.413022e-38
p value ranksum for S+ high Hi2 Naive vs S+ high Low5 Naive = 4.008143e-38
p value ranksum for S+ high Hi3 Naive vs S+ high Low6 Naive = 4.228599e-38
p value ranksum for S+ low Hi1 Naive vs S+ low Low4 Proficient = 4.649295e-38
p value ranksum for S+ high Low6 Naive vs S+ low Low5 Naive = 8.100530e-38
p value ranksum for S+ high Hi1 Naive vs S+ low Hi3 Naive = 8.707755e-38
p value ranksum for S+ high Hi3 Naive vs S+ high Low4 Naive = 4.386063e-37
p value ranksum for S+ high Hi3 Naive vs S+ high Low5 Naive = 4.747407e-37
p value ranksum for S+ high Hi3 Proficient vs S+ high Low6 Naive = 5.862194e-37
p value ranksum for S+ low Hi2 Naive vs S+ low Low5 Naive = 6.104135e-37
p value ranksum for S+ high Hi2 Proficient vs S+ low Hi1 Naive = 9.748123e-37
p value ranksum for S+ high Low6 Naive vs S+ low Hi3 Proficient = 1.549527e-36
p value ranksum for S+ low Hi1 Naive vs S+ low Hi1 Proficient = 1.642066e-36
p value ranksum for S+ high Low6 Naive vs S+ low Low5 Proficient = 1.720621e-36
p value ranksum for S+ high Low6 Naive vs S+ low Hi2 Proficient = 2.417199e-36

p value ranksum for S+ high Hi2 Naive vs S+ low Hi3 Naive = 2.454103e-36
p value ranksum for S+ high Low5 Proficient vs S+ low Hi1 Naive = 5.214253e-36
p value ranksum for S+ high Low4 Naive vs S+ low Low4 Naive = 8.143287e-36
p value ranksum for S+ high Hi1 Proficient vs S+ low Hi1 Naive = 1.156819e-35
p value ranksum for S+ high Low6 Naive vs S+ low Low6 Proficient = 2.192419e-35
p value ranksum for S+ high Low5 Naive vs S+ low Low6 Naive = 2.677981e-35
p value ranksum for S+ low Hi2 Naive vs S+ low Hi3 Proficient = 2.740981e-35
p value ranksum for S+ high Low4 Naive vs S+ low Low6 Naive = 3.300954e-35
p value ranksum for S+ high Low5 Naive vs S+ low Low4 Naive = 4.896431e-35
p value ranksum for S+ high Low5 Naive vs S+ low Low5 Naive = 5.128579e-35
p value ranksum for S+ low Hi2 Naive vs S+ low Hi2 Proficient = 6.615090e-35
p value ranksum for S+ high Hi3 Proficient vs S+ low Hi2 Naive = 8.931791e-35
p value ranksum for S+ high Hi3 Naive vs S+ low Hi3 Naive = 1.823379e-34
p value ranksum for S+ high Low6 Naive vs S+ low Low4 Proficient = 3.369291e-34
p value ranksum for S+ low Hi2 Naive vs S+ low Low5 Proficient = 1.107153e-33
p value ranksum for S+ low Hi2 Naive vs S+ low Low6 Proficient = 3.063572e-33
p value ranksum for S+ high Hi3 Proficient vs S+ high Low5 Naive = 4.466357e-33
p value ranksum for S+ high Low6 Naive vs S+ low Hi1 Proficient = 6.324300e-33
p value ranksum for S+ high Low6 Proficient vs S+ low Hi1 Naive = 7.526407e-33
p value ranksum for S+ high Low4 Naive vs S+ low Hi3 Proficient = 1.090598e-32
p value ranksum for S+ low Hi2 Naive vs S+ low Low4 Proficient = 1.258807e-32
p value ranksum for S+ high Low4 Naive vs S+ low Hi2 Proficient = 2.020738e-32
p value ranksum for S+ high Hi2 Proficient vs S+ high Low6 Naive = 9.000003e-32
p value ranksum for S+ high Low4 Naive vs S+ low Low5 Naive = 1.128184e-31
p value ranksum for S+ high Hi1 Naive vs S+ high Low4 Proficient = 2.376496e-31
p value ranksum for S+ high Hi1 Naive vs S+ low Hi2 Proficient = 2.747504e-31
p value ranksum for S+ high Low5 Naive vs S+ low Low5 Proficient = 7.198786e-31
p value ranksum for S+ high Low5 Naive vs S+ low Hi3 Proficient = 2.060746e-30
p value ranksum for S+ high Low5 Naive vs S+ low Low6 Proficient = 3.314912e-30
p value ranksum for S+ low Hi3 Naive vs S+ low Low6 Naive = 8.957897e-30
p value ranksum for S+ high Hi1 Naive vs S+ high Low5 Proficient = 1.609640e-29
p value ranksum for S+ high Hi3 Proficient vs S+ high Low4 Naive = 2.340961e-29
p value ranksum for S+ high Hi1 Proficient vs S+ low Hi2 Naive = 3.314404e-29
p value ranksum for S+ high Hi1 Proficient vs S+ high Low6 Naive = 3.481065e-29
p value ranksum for S+ high Hi1 Proficient vs S+ high Low5 Naive = 3.647119e-29
p value ranksum for S+ high Low4 Proficient vs S+ low Hi1 Naive = 5.264583e-29
p value ranksum for S+ high Low5 Proficient vs S+ high Low6 Naive = 6.520861e-29
p value ranksum for S+ high Low5 Naive vs S+ low Low4 Proficient = 7.849042e-29
p value ranksum for S+ high Low4 Naive vs S+ low Low5 Proficient = 8.413532e-29
p value ranksum for S+ high Hi2 Naive vs S+ high Low4 Proficient = 1.335472e-28
p value ranksum for S+ high Hi1 Naive vs S+ high Low6 Proficient = 1.464462e-28
p value ranksum for S+ high Hi2 Proficient vs S+ high Low5 Naive = 1.605802e-28
p value ranksum for S+ high Low5 Proficient vs S+ low Hi2 Naive = 3.162115e-28
p value ranksum for S+ low Hi3 Naive vs S+ low Low5 Naive = 5.601773e-28
p value ranksum for S+ high Hi2 Proficient vs S+ low Hi2 Naive = 6.926124e-28
p value ranksum for S+ high Hi2 Naive vs S+ low Hi2 Proficient = 7.272625e-28
p value ranksum for S+ high Low4 Naive vs S+ low Low6 Proficient = 8.335503e-28
p value ranksum for S+ high Low5 Naive vs S+ low Hi2 Proficient = 9.552267e-28
p value ranksum for S+ high Low4 Naive vs S+ low Low4 Proficient = 1.225807e-27
p value ranksum for S+ low Hi3 Naive vs S+ low Low4 Naive = 1.500298e-27
p value ranksum for S+ low Hi1 Proficient vs S+ low Hi2 Naive = 1.610805e-26

p value ranksum for S+ high Hi2 Naive vs S+ high Low5 Proficient = 3.061492e-26
p value ranksum for S+ high Hi3 Naive vs S+ high Low4 Proficient = 1.480644e-25
p value ranksum for S+ high Hi1 Proficient vs S+ high Low4 Naive = 1.963508e-25
p value ranksum for S+ high Low6 Naive vs S+ high Low6 Proficient = 8.315663e-25
p value ranksum for S+ high Hi2 Naive vs S+ high Low6 Proficient = 1.029803e-24
p value ranksum for S+ high Hi3 Naive vs S+ high Low5 Proficient = 1.330274e-24
p value ranksum for S+ low Hi1 Naive vs S+ low Hi3 Naive = 1.653851e-24
p value ranksum for S+ high Low4 Naive vs S+ high Low5 Proficient = 2.169269e-24
p value ranksum for S+ high Hi3 Proficient vs S+ low Hi3 Naive = 5.210635e-24
p value ranksum for S+ high Hi3 Naive vs S+ high Low6 Proficient = 1.192688e-23
p value ranksum for S+ high Hi1 Naive vs S+ low Low4 Proficient = 1.323810e-23
p value ranksum for S+ high Hi1 Naive vs S+ low Hi1 Proficient = 2.049187e-23
p value ranksum for S+ high Low5 Naive vs S+ low Hi1 Proficient = 5.301750e-23
p value ranksum for S+ low Hi3 Naive vs S+ low Hi3 Proficient = 7.917503e-23
p value ranksum for S+ low Hi3 Naive vs S+ low Low6 Proficient = 1.006930e-22
p value ranksum for S+ high Hi3 Naive vs S+ low Hi2 Proficient = 1.202993e-22
p value ranksum for S+ high Hi2 Proficient vs S+ high Low4 Naive = 1.387507e-22
p value ranksum for S+ low Hi3 Naive vs S+ low Low5 Proficient = 2.102233e-22
p value ranksum for S+ high Low4 Proficient vs S+ high Low6 Naive = 5.070325e-21
p value ranksum for S+ high Low4 Naive vs S+ low Hi1 Proficient = 7.072513e-21
p value ranksum for S+ high Hi2 Naive vs S+ low Low4 Proficient = 2.856814e-20
p value ranksum for S+ high Low5 Naive vs S+ high Low5 Proficient = 3.206123e-20
p value ranksum for S+ high Hi1 Naive vs S+ low Hi3 Proficient = 5.078273e-20
p value ranksum for S+ high Hi1 Proficient vs S+ low Hi3 Naive = 5.810203e-20
p value ranksum for S+ high Low4 Proficient vs S+ low Hi2 Naive = 2.097908e-19
p value ranksum for S+ high Hi3 Naive vs S+ low Hi1 Proficient = 3.977556e-19
p value ranksum for S+ high Low4 Naive vs S+ low Hi1 Naive = 4.490735e-19
p value ranksum for S+ low Hi3 Naive vs S+ low Low4 Proficient = 4.967755e-19
p value ranksum for S+ high Hi2 Naive vs S+ low Hi1 Proficient = 6.947657e-19
p value ranksum for S+ high Low6 Proficient vs S+ low Hi2 Naive = 9.232889e-19
p value ranksum for S+ high Low5 Naive vs S+ high Low6 Proficient = 1.123145e-18
p value ranksum for S+ low Hi2 Proficient vs S+ low Hi3 Naive = 2.456664e-18
p value ranksum for S+ high Hi1 Naive vs S+ high Hi2 Proficient = 4.749906e-18
p value ranksum for S+ high Low6 Proficient vs S+ low Low6 Naive = 3.447990e-17
p value ranksum for S+ high Low4 Proficient vs S+ low Low5 Naive = 7.612804e-17
p value ranksum for S+ high Low4 Proficient vs S+ low Low6 Naive = 1.858410e-16
p value ranksum for S+ high Low6 Proficient vs S+ low Low5 Naive = 2.382713e-16
p value ranksum for S+ high Hi2 Proficient vs S+ low Hi3 Naive = 4.418051e-16
p value ranksum for S+ high Hi3 Naive vs S+ low Low4 Proficient = 1.149849e-15
p value ranksum for S+ high Low5 Proficient vs S+ low Low6 Naive = 1.227625e-15
p value ranksum for S+ high Low4 Proficient vs S+ high Low5 Naive = 1.292900e-15
p value ranksum for S+ high Low6 Naive vs S+ low Hi3 Naive = 1.494204e-15
p value ranksum for S+ high Low5 Proficient vs S+ low Low5 Naive = 2.766471e-15
p value ranksum for S+ high Hi1 Naive vs S+ low Low4 Naive = 4.799526e-15
p value ranksum for S+ high Hi3 Proficient vs S+ high Low5 Proficient = 2.619240e-14
p value ranksum for S+ high Low6 Proficient vs S+ low Low4 Naive = 3.443270e-14
p value ranksum for S+ high Low4 Naive vs S+ high Low4 Proficient = 4.287966e-14
p value ranksum for S+ high Hi1 Naive vs S+ low Low5 Proficient = 5.609273e-14
p value ranksum for S+ high Hi1 Proficient vs S+ high Low5 Proficient = 5.698391e-14
p value ranksum for S+ low Hi1 Proficient vs S+ low Low6 Naive = 9.601045e-14
p value ranksum for S+ high Low5 Proficient vs S+ low Low4 Naive = 1.186126e-13

p value ranksum for S+ high Hi2 Naive vs S+ low Hi3 Proficient = 1.726455e-13
p value ranksum for S+ low Hi2 Proficient vs S+ low Low6 Naive = 3.282364e-13
p value ranksum for S+ high Hi1 Naive vs S+ high Hi3 Proficient = 3.500771e-13
p value ranksum for S+ low Hi2 Proficient vs S+ low Low5 Naive = 4.324329e-13
p value ranksum for S+ high Hi3 Proficient vs S+ high Low6 Proficient = 5.279289e-13
p value ranksum for S+ high Hi3 Naive vs S+ low Hi3 Proficient = 6.142118e-13
p value ranksum for S+ low Hi1 Proficient vs S+ low Low5 Naive = 1.107753e-12
p value ranksum for S+ high Low5 Proficient vs S+ low Hi3 Naive = 1.510253e-12
p value ranksum for S+ high Low4 Proficient vs S+ low Low4 Naive = 1.797981e-12
p value ranksum for S+ low Hi1 Proficient vs S+ low Hi3 Naive = 1.846777e-12
p value ranksum for S+ high Hi1 Proficient vs S+ high Low4 Proficient = 3.205394e-12
p value ranksum for S+ high Hi1 Proficient vs S+ high Low6 Proficient = 3.398776e-12
p value ranksum for S+ high Low4 Naive vs S+ high Low6 Proficient = 4.617561e-12
p value ranksum for S+ high Hi3 Proficient vs S+ high Low4 Proficient = 1.066213e-11
p value ranksum for S+ high Hi2 Naive vs S+ high Hi2 Proficient = 2.793690e-11
p value ranksum for S+ high Hi2 Proficient vs S+ high Hi3 Naive = 3.258844e-11
p value ranksum for S+ high Hi1 Naive vs S+ low Low6 Proficient = 7.690587e-11
p value ranksum for S+ high Low4 Naive vs S+ high Low6 Naive = 1.381971e-10
p value ranksum for S+ high Low4 Proficient vs S+ low Low6 Proficient = 1.988923e-10
p value ranksum for S+ high Low5 Naive vs S+ low Hi3 Naive = 4.158998e-10
p value ranksum for S+ high Low6 Proficient vs S+ low Low6 Proficient = 8.278083e-10
p value ranksum for S+ high Low6 Proficient vs S+ low Low5 Proficient = 1.235399e-09
p value ranksum for S+ low Hi1 Proficient vs S+ low Low4 Naive = 1.257334e-09
p value ranksum for S+ low Hi1 Naive vs S+ low Hi2 Naive = 3.726324e-09
p value ranksum for S+ low Hi2 Proficient vs S+ low Low4 Naive = 4.368046e-09
p value ranksum for S+ low Low4 Proficient vs S+ low Low5 Naive = 4.725224e-09
p value ranksum for S+ high Low5 Proficient vs S+ low Low6 Proficient = 5.014132e-09
p value ranksum for S+ high Hi3 Naive vs S+ low Low4 Naive = 6.460150e-09
p value ranksum for S+ high Low5 Proficient vs S+ low Low5 Proficient = 7.716650e-09
p value ranksum for S+ high Hi3 Proficient vs S+ low Hi1 Proficient = 7.907901e-09
p value ranksum for S+ high Low4 Proficient vs S+ low Low5 Proficient = 1.009230e-08
p value ranksum for S+ high Hi3 Naive vs S+ low Low5 Proficient = 1.433207e-08
p value ranksum for S+ high Hi1 Naive vs S+ high Hi1 Proficient = 1.695512e-08
p value ranksum for S+ high Hi1 Proficient vs S+ low Hi1 Proficient = 2.423966e-08
p value ranksum for S+ high Low4 Proficient vs S+ low Hi3 Naive = 2.902771e-08
p value ranksum for S+ high Low6 Proficient vs S+ low Hi3 Proficient = 2.963262e-08
p value ranksum for S+ low Low4 Proficient vs S+ low Low6 Naive = 4.206065e-08
p value ranksum for S+ high Hi2 Naive vs S+ low Low4 Naive = 4.924398e-08
p value ranksum for S+ high Hi2 Proficient vs S+ high Low6 Proficient = 5.895646e-08
p value ranksum for S+ high Low5 Naive vs S+ low Hi1 Naive = 6.947719e-08
p value ranksum for S+ high Low6 Naive vs S+ low Hi1 Naive = 6.947719e-08
p value ranksum for S+ low Hi1 Proficient vs S+ low Low6 Proficient = 8.130761e-08
p value ranksum for S+ high Hi2 Naive vs S+ low Low5 Proficient = 8.907557e-08
p value ranksum for S+ high Hi1 Proficient vs S+ low Hi2 Proficient = 1.182647e-07
p value ranksum for S+ low Hi2 Naive vs S+ low Hi3 Naive = 1.485047e-07
p value ranksum for S+ high Hi3 Proficient vs S+ low Hi2 Proficient = 1.851316e-07
p value ranksum for S+ low Hi3 Proficient vs S+ low Low6 Naive = 2.653718e-07
p value ranksum for S+ low Hi2 Proficient vs S+ low Low6 Proficient = 5.115846e-07
p value ranksum for S+ high Hi1 Naive vs S+ low Low6 Naive = 5.172944e-07
p value ranksum for S+ high Hi3 Naive vs S+ high Hi3 Proficient = 5.514653e-07
p value ranksum for S+ high Low5 Proficient vs S+ low Low4 Proficient = 8.347358e-07

p value ranksum for S+ high Low6 Proficient vs S+ low Low4 Proficient = 8.706833e-07
p value ranksum for S+ high Hi2 Naive vs S+ high Hi3 Proficient = 1.143371e-06
p value ranksum for S+ high Hi2 Proficient vs S+ high Low5 Proficient = 1.350219e-06
p value ranksum for S+ low Hi3 Proficient vs S+ low Low5 Naive = 1.354712e-06
p value ranksum for S+ high Hi2 Proficient vs S+ high Low4 Proficient = 1.995684e-06
p value ranksum for S+ low Hi1 Proficient vs S+ low Low5 Proficient = 2.100040e-06
p value ranksum for S+ high Hi3 Naive vs S+ low Low6 Proficient = 2.624800e-06
p value ranksum for S+ high Low5 Proficient vs S+ low Hi3 Proficient = 3.407744e-06
p value ranksum for S+ high Low6 Proficient vs S+ low Hi3 Naive = 4.140801e-06
p value ranksum for S+ high Low4 Naive vs S+ high Low5 Naive = 4.412000e-06
p value ranksum for S+ high Low4 Proficient vs S+ low Hi3 Proficient = 4.455800e-06
p value ranksum for S+ low Hi2 Proficient vs S+ low Low5 Proficient = 6.529156e-06
p value ranksum for S+ high Low4 Proficient vs S+ low Low4 Proficient = 6.722165e-06
p value ranksum for S+ high Hi1 Naive vs S+ low Low5 Naive = 1.086588e-05
p value ranksum for S+ high Hi2 Naive vs S+ low Low6 Proficient = 1.997455e-05
p value ranksum for S+ low Hi1 Proficient vs S+ low Hi3 Proficient = 2.678689e-05
p value ranksum for S+ high Hi1 Proficient vs S+ high Hi2 Naive = 7.366466e-05
p value ranksum for S+ high Hi2 Proficient vs S+ low Low6 Naive = 7.874869e-05
p value ranksum for S+ high Hi2 Proficient vs S+ low Low5 Naive = 1.148543e-04
p value ranksum for S+ high Hi1 Proficient vs S+ high Hi3 Naive = 2.472002e-04
p value ranksum for S+ high Low6 Proficient vs S+ low Hi2 Proficient = 2.532435e-04
p value ranksum for S+ low Hi1 Proficient vs S+ low Low4 Proficient = 5.340482e-04
p value ranksum for S+ low Low5 Naive vs S+ low Low5 Proficient = 7.254955e-04
p value ranksum for S+ low Hi2 Proficient vs S+ low Hi3 Proficient = 7.453125e-04
p value ranksum for S+ low Low4 Proficient vs S+ low Low6 Proficient = 1.009723e-03
p value ranksum for S+ high Hi1 Naive vs S+ high Hi2 Naive = 1.151260e-03
p value ranksum for S+ high Hi3 Proficient vs S+ low Low4 Proficient = 1.185108e-03
p value ranksum for S+ high Hi1 Proficient vs S+ low Low4 Proficient = 1.193712e-03
p value ranksum for S+ low Low5 Proficient vs S+ low Low6 Naive = 1.352626e-03
p value ranksum for S+ low Low4 Naive vs S+ low Low4 Proficient = 1.558452e-03
p value ranksum for S+ high Low4 Naive vs S+ low Hi2 Naive = 1.897653e-03
p value ranksum for S+ low Low4 Naive vs S+ low Low5 Naive = 2.011750e-03
p value ranksum for S+ low Low5 Naive vs S+ low Low6 Proficient = 2.480895e-03
p value ranksum for S+ low Low4 Naive vs S+ low Low6 Naive = 3.318772e-03
p value ranksum for S+ high Hi2 Proficient vs S+ low Hi2 Proficient = 4.141701e-03
p value ranksum for S+ high Hi2 Proficient vs S+ low Hi1 Proficient = 5.565961e-03
p value ranksum for S+ low Low6 Naive vs S+ low Low6 Proficient = 6.274769e-03
p value ranksum for S+ high Hi1 Proficient vs S+ low Hi3 Proficient = 9.069664e-03
p value ranksum for S+ low Hi2 Proficient vs S+ low Low4 Proficient = 1.059422e-02
p value ranksum for S+ high Hi3 Naive vs S+ low Low6 Naive = 1.364171e-02
p value ranksum for S+ low Low4 Proficient vs S+ low Low5 Proficient = 1.393659e-02
p value ranksum for S+ high Hi3 Proficient vs S+ low Low5 Naive = 1.451745e-02
p value ranksum for S+ high Hi3 Proficient vs S+ low Hi3 Proficient = 1.527039e-02
p value ranksum for S+ low Hi3 Proficient vs S+ low Low6 Proficient = 1.921355e-02
p value ranksum for S+ high Hi3 Naive vs S+ low Low5 Naive = 1.987757e-02
p value ranksum for S+ high Hi3 Proficient vs S+ low Low6 Naive = 2.360966e-02
p value ranksum for S+ low Hi1 Proficient vs S+ low Hi2 Proficient = 2.937134e-02
p value ranksum for S+ high Low6 Naive vs S+ low Hi2 Naive = 3.128206e-02
p value ranksum for S+ high Low5 Proficient vs S+ low Hi2 Proficient = 3.590076e-02
p value ranksum for S+ low Hi3 Proficient vs S+ low Low4 Naive = 3.762475e-02

p values below are > pFDR

p value ranksum for S+ high Low4 Naive vs S+ low Hi3 Naive = 4.727005e-02
p value ranksum for S+ high Low5 Proficient vs S+ low Hi1 Proficient = 9.924166e-02
p value ranksum for S+ high Low4 Proficient vs S+ low Hi2 Proficient = 1.026699e-01
p value ranksum for S+ high Hi1 Naive vs S+ high Hi3 Naive = 1.304190e-01
p value ranksum for S+ high Hi2 Proficient vs S+ low Low6 Proficient = 1.309441e-01
p value ranksum for S+ high Hi2 Naive vs S+ low Low6 Naive = 1.342747e-01
p value ranksum for S+ high Hi2 Proficient vs S+ low Low4 Naive = 1.422028e-01
p value ranksum for S+ high Hi1 Proficient vs S+ high Hi2 Proficient = 1.434771e-01
p value ranksum for S+ high Hi1 Proficient vs S+ low Low5 Naive = 1.452669e-01
p value ranksum for S+ low Hi3 Proficient vs S+ low Low5 Proficient = 1.642874e-01
p value ranksum for S+ high Hi1 Proficient vs S+ low Low6 Naive = 1.860779e-01
p value ranksum for S+ high Low6 Proficient vs S+ low Hi1 Proficient = 1.992913e-01
p value ranksum for S+ high Low5 Naive vs S+ low Hi2 Naive = 2.087381e-01
p value ranksum for S+ high Hi1 Proficient vs S+ low Low5 Proficient = 2.218156e-01
p value ranksum for S+ high Hi1 Proficient vs S+ low Low6 Proficient = 2.749164e-01
p value ranksum for S+ high Hi2 Naive vs S+ high Hi3 Naive = 2.840836e-01
p value ranksum for S+ high Low4 Proficient vs S+ high Low6 Proficient = 3.059459e-01
p value ranksum for S+ high Hi2 Proficient vs S+ high Hi3 Proficient = 3.217958e-01
p value ranksum for S+ high Low5 Proficient vs S+ high Low6 Proficient = 3.278753e-01
p value ranksum for S+ low Low4 Naive vs S+ low Low5 Proficient = 3.451968e-01
p value ranksum for S+ high Hi2 Proficient vs S+ low Low5 Proficient = 3.476151e-01
p value ranksum for S+ high Hi2 Proficient vs S+ low Low4 Proficient = 3.659043e-01
p value ranksum for S+ high Hi2 Naive vs S+ low Low5 Naive = 4.266987e-01
p value ranksum for S+ high Hi1 Proficient vs S+ low Low4 Naive = 4.576809e-01
p value ranksum for S+ high Hi1 Proficient vs S+ high Hi3 Proficient = 4.610296e-01
p value ranksum for S+ high Low4 Proficient vs S+ high Low5 Proficient = 5.021124e-01
p value ranksum for S+ high Hi3 Proficient vs S+ low Low6 Proficient = 5.087022e-01
p value ranksum for S+ low Hi3 Proficient vs S+ low Low4 Proficient = 5.260454e-01
p value ranksum for S+ low Low5 Proficient vs S+ low Low6 Proficient = 5.854854e-01
p value ranksum for S+ high Low4 Proficient vs S+ low Hi1 Proficient = 6.026132e-01
p value ranksum for S+ high Hi3 Proficient vs S+ low Low5 Proficient = 6.672540e-01
p value ranksum for S+ high Low5 Naive vs S+ high Low6 Naive = 7.020946e-01
p value ranksum for S+ low Low4 Naive vs S+ low Low6 Proficient = 9.131831e-01
p value ranksum for S+ low Low5 Naive vs S+ low Low6 Naive = 9.266942e-01
p value ranksum for S+ high Hi2 Proficient vs S+ low Hi3 Proficient = 9.383660e-01
p value ranksum for S+ high Hi3 Proficient vs S+ low Low4 Naive = 9.784413e-01

Ranksum or t-test for prp trough for theta High gamma

pFDR = 2.880435e-02

p value ranksum for S+ low Hi1 Naive vs S+ low Hi3 Proficient = 1.606489e-30
p value ranksum for S+ low Hi1 Naive vs S+ low Low5 Proficient = 2.470865e-30
p value ranksum for S+ high Hi3 Proficient vs S+ low Hi1 Naive = 2.635379e-30
p value ranksum for S+ high Hi1 Naive vs S+ low Hi1 Naive = 4.408699e-30

p value t-test for S+ high Low6 Naive vs S+ low Hi3 Proficient = 3.572796e-29
p value ranksum for S+ high Hi3 Naive vs S+ low Hi1 Naive = 7.366833e-28
p value ranksum for S+ low Hi1 Naive vs S+ low Low4 Naive = 1.035450e-27
p value ranksum for S+ low Hi1 Naive vs S+ low Low6 Proficient = 2.224373e-27
p value ranksum for S+ high Hi2 Naive vs S+ low Hi1 Naive = 3.623331e-27
p value ranksum for S+ low Hi1 Naive vs S+ low Hi1 Proficient = 4.910301e-27
p value ranksum for S+ low Hi1 Naive vs S+ low Hi2 Proficient = 5.772588e-27
p value ranksum for S+ low Hi1 Naive vs S+ low Low6 Naive = 1.355677e-26
p value ranksum for S+ high Hi1 Naive vs S+ high Low6 Naive = 5.791392e-26
p value ranksum for S+ high Low5 Proficient vs S+ low Hi1 Naive = 9.450751e-26
p value t-test for S+ high Low6 Naive vs S+ low Low5 Proficient = 2.201503e-25
p value t-test for S+ high Low6 Naive vs S+ low Hi2 Proficient = 7.781817e-25
p value ranksum for S+ low Hi1 Naive vs S+ low Low4 Proficient = 1.147750e-24
p value ranksum for S+ high Hi3 Proficient vs S+ high Low6 Naive = 9.281600e-24
p value ranksum for S+ high Hi3 Naive vs S+ high Low6 Naive = 1.531677e-23
p value ranksum for S+ high Low6 Proficient vs S+ low Hi1 Naive = 1.682723e-23
p value ranksum for S+ high Low4 Proficient vs S+ low Hi1 Naive = 3.494008e-23
p value t-test for S+ high Low6 Naive vs S+ low Low6 Proficient = 1.087696e-22
p value t-test for S+ high Low6 Naive vs S+ low Hi1 Proficient = 1.373257e-22
p value t-test for S+ high Low5 Naive vs S+ low Hi3 Proficient = 1.429818e-22
p value ranksum for S+ high Hi2 Proficient vs S+ low Hi1 Naive = 9.864397e-22
p value ranksum for S+ low Hi1 Naive vs S+ low Low5 Naive = 3.690786e-21
p value t-test for S+ high Low6 Naive vs S+ low Low4 Proficient = 9.686330e-21
p value ranksum for S+ high Hi1 Proficient vs S+ low Hi1 Naive = 1.054280e-20
p value t-test for S+ high Low6 Naive vs S+ low Low4 Naive = 7.460965e-20
p value ranksum for S+ high Hi1 Naive vs S+ high Low5 Naive = 1.915456e-19
p value ranksum for S+ high Hi3 Proficient vs S+ high Low5 Naive = 6.571887e-19
p value ranksum for S+ high Hi2 Naive vs S+ high Low6 Naive = 1.102645e-18
p value t-test for S+ high Low6 Naive vs S+ low Low6 Naive = 2.246319e-18
p value t-test for S+ high Low5 Naive vs S+ low Low5 Proficient = 2.976712e-18
p value ranksum for S+ high Low5 Proficient vs S+ high Low6 Naive = 1.106276e-17
p value t-test for S+ high Low5 Naive vs S+ low Hi2 Proficient = 1.203362e-17
p value ranksum for S+ high Hi3 Naive vs S+ high Low5 Naive = 1.019405e-16
p value t-test for S+ high Low5 Naive vs S+ low Hi1 Proficient = 4.136522e-16
p value ranksum for S+ high Hi1 Proficient vs S+ high Low6 Naive = 1.553847e-15
p value ranksum for S+ high Low4 Naive vs S+ low Hi3 Proficient = 2.132069e-15
p value ranksum for S+ high Low4 Naive vs S+ low Low5 Proficient = 2.316584e-15
p value t-test for S+ high Low5 Naive vs S+ low Low6 Proficient = 3.011509e-15
p value ranksum for S+ high Hi3 Proficient vs S+ high Low4 Naive = 4.557392e-15
p value ranksum for S+ low Hi2 Naive vs S+ low Low5 Proficient = 5.732754e-15
p value t-test for S+ high Low5 Naive vs S+ low Hi1 Naive = 1.087756e-14
p value ranksum for S+ high Hi1 Naive vs S+ high Low4 Naive = 1.271486e-14
p value ranksum for S+ low Hi1 Naive vs S+ low Hi3 Naive = 1.985035e-14
p value ranksum for S+ high Hi3 Proficient vs S+ low Hi2 Naive = 2.266841e-14
p value ranksum for S+ high Hi1 Naive vs S+ low Hi2 Naive = 2.367336e-14
p value ranksum for S+ high Low4 Proficient vs S+ high Low6 Naive = 3.837640e-14
p value t-test for S+ high Low6 Naive vs S+ high Low6 Proficient = 2.360650e-13
p value ranksum for S+ high Hi2 Proficient vs S+ high Low6 Naive = 3.050697e-13
p value ranksum for S+ low Hi2 Naive vs S+ low Hi3 Proficient = 4.149510e-13
p value ranksum for S+ high Hi2 Naive vs S+ high Low5 Naive = 6.049953e-13
p value t-test for S+ high Low5 Naive vs S+ low Low4 Naive = 6.096624e-13

p value t-test for S+ high Low5 Naive vs S+ low Low4 Proficient = 2.479484e-12
p value t-test for S+ high Low5 Naive vs S+ low Low6 Naive = 4.562052e-12
p value ranksum for S+ low Hi2 Naive vs S+ low Low4 Naive = 4.946112e-12
p value ranksum for S+ high Hi3 Naive vs S+ low Hi2 Naive = 7.886965e-12
p value ranksum for S+ low Hi2 Naive vs S+ low Low6 Naive = 9.474661e-12
p value ranksum for S+ high Hi3 Naive vs S+ high Low4 Naive = 9.784312e-12
p value ranksum for S+ low Hi2 Naive vs S+ low Low6 Proficient = 1.323552e-11
p value t-test for S+ high Low5 Naive vs S+ high Low5 Proficient = 1.424078e-11
p value ranksum for S+ high Hi1 Proficient vs S+ high Low5 Naive = 1.634689e-11
p value ranksum for S+ high Low4 Naive vs S+ low Hi1 Naive = 2.508717e-11
p value ranksum for S+ high Hi2 Naive vs S+ low Hi2 Naive = 6.771772e-11
p value ranksum for S+ low Hi2 Naive vs S+ low Hi2 Proficient = 7.295898e-11
p value t-test for S+ high Low6 Naive vs S+ low Low5 Naive = 8.795752e-11
p value ranksum for S+ high Low4 Naive vs S+ low Low6 Proficient = 2.244046e-10
p value ranksum for S+ low Hi1 Proficient vs S+ low Hi2 Naive = 4.012733e-10
p value ranksum for S+ high Low4 Naive vs S+ low Hi1 Proficient = 4.247182e-10
p value ranksum for S+ high Low5 Proficient vs S+ low Hi2 Naive = 5.467038e-10
p value ranksum for S+ high Low4 Naive vs S+ low Hi2 Proficient = 7.364182e-10
p value ranksum for S+ high Low4 Naive vs S+ low Low4 Naive = 9.961284e-10
p value ranksum for S+ high Hi2 Naive vs S+ high Low4 Naive = 1.114555e-09
p value ranksum for S+ high Low4 Naive vs S+ high Low5 Proficient = 5.203921e-09
p value t-test for S+ high Low5 Naive vs S+ high Low6 Proficient = 5.671375e-09
p value ranksum for S+ high Hi1 Proficient vs S+ high Low4 Naive = 5.816186e-09
p value ranksum for S+ low Hi2 Naive vs S+ low Low4 Proficient = 6.388689e-09
p value ranksum for S+ high Hi1 Proficient vs S+ low Hi2 Naive = 8.521904e-09
p value ranksum for S+ high Low4 Proficient vs S+ high Low5 Naive = 1.009230e-08
p value ranksum for S+ high Low4 Proficient vs S+ low Hi2 Naive = 2.082841e-08
p value ranksum for S+ high Low4 Naive vs S+ low Low6 Naive = 2.526572e-08
p value t-test for S+ high Low6 Naive vs S+ low Hi1 Naive = 5.548567e-08
p value ranksum for S+ high Low6 Proficient vs S+ low Hi2 Naive = 2.378631e-07
p value ranksum for S+ high Hi2 Proficient vs S+ low Hi2 Naive = 2.872825e-07
p value ranksum for S+ low Hi2 Naive vs S+ low Low5 Naive = 3.038566e-07
p value ranksum for S+ high Low4 Naive vs S+ low Low4 Proficient = 4.305987e-07
p value ranksum for S+ high Hi2 Proficient vs S+ high Low5 Naive = 6.472652e-07
p value ranksum for S+ high Low4 Naive vs S+ high Low4 Proficient = 7.918081e-07
p value ranksum for S+ low Hi3 Naive vs S+ low Low5 Proficient = 8.367223e-07
p value t-test for S+ high Low5 Naive vs S+ high Low6 Naive = 1.534385e-06
p value ranksum for S+ high Hi1 Naive vs S+ low Hi3 Naive = 1.694393e-06
p value ranksum for S+ high Hi3 Proficient vs S+ low Hi3 Naive = 1.911156e-06
p value ranksum for S+ high Hi1 Naive vs S+ high Hi2 Proficient = 2.572324e-06
p value t-test for S+ high Low5 Naive vs S+ low Low5 Naive = 2.586043e-06
p value ranksum for S+ high Hi2 Proficient vs S+ high Hi3 Proficient = 3.241592e-06
p value ranksum for S+ high Hi2 Proficient vs S+ low Low5 Proficient = 4.157738e-06
p value ranksum for S+ high Low4 Naive vs S+ high Low6 Proficient = 8.077336e-06
p value ranksum for S+ high Hi2 Proficient vs S+ low Hi3 Proficient = 8.234298e-06
p value t-test for S+ high Low6 Naive vs S+ low Hi3 Naive = 1.646100e-05
p value ranksum for S+ low Low4 Proficient vs S+ low Low5 Proficient = 2.445063e-05
p value ranksum for S+ low Hi3 Naive vs S+ low Hi3 Proficient = 3.359950e-05
p value ranksum for S+ low Hi1 Naive vs S+ low Hi2 Naive = 3.547189e-05
p value ranksum for S+ high Hi1 Naive vs S+ low Low5 Naive = 4.282613e-05
p value ranksum for S+ high Low6 Proficient vs S+ low Low5 Proficient = 4.315442e-05

p value ranksum for S+ high Hi3 Proficient vs S+ low Low4 Proficient = 1.228289e-04
p value ranksum for S+ high Hi3 Naive vs S+ low Hi3 Naive = 1.496626e-04
p value ranksum for S+ high Hi2 Proficient vs S+ high Hi3 Naive = 1.564425e-04
p value ranksum for S+ high Hi3 Proficient vs S+ high Low6 Proficient = 1.969645e-04
p value ranksum for S+ high Hi2 Proficient vs S+ high Low4 Naive = 2.154325e-04
p value ranksum for S+ low Hi3 Naive vs S+ low Low4 Naive = 2.848476e-04
p value ranksum for S+ low Hi3 Proficient vs S+ low Low4 Proficient = 3.043934e-04
p value ranksum for S+ high Hi1 Naive vs S+ low Low4 Proficient = 3.455181e-04
p value ranksum for S+ high Low4 Proficient vs S+ low Low5 Proficient = 3.593998e-04
p value ranksum for S+ low Hi3 Proficient vs S+ low Low5 Naive = 4.203157e-04
p value ranksum for S+ high Hi3 Naive vs S+ low Low5 Naive = 4.972005e-04
p value ranksum for S+ low Hi1 Proficient vs S+ low Low5 Proficient = 4.983749e-04
p value ranksum for S+ high Low4 Naive vs S+ high Low6 Naive = 6.267425e-04
p value ranksum for S+ low Hi2 Naive vs S+ low Hi3 Naive = 6.276588e-04
p value ranksum for S+ low Hi3 Naive vs S+ low Low6 Naive = 9.063781e-04
p value ranksum for S+ high Hi1 Proficient vs S+ low Hi3 Naive = 1.062133e-03
p value ranksum for S+ low Hi3 Naive vs S+ low Low6 Proficient = 1.090494e-03
p value ranksum for S+ low Low5 Naive vs S+ low Low5 Proficient = 1.126931e-03
p value ranksum for S+ high Hi1 Naive vs S+ high Low6 Proficient = 1.159637e-03
p value ranksum for S+ high Hi2 Naive vs S+ low Hi3 Naive = 1.275896e-03
p value ranksum for S+ high Hi3 Proficient vs S+ low Low5 Naive = 1.433592e-03
p value ranksum for S+ high Hi1 Proficient vs S+ high Hi2 Proficient = 2.146075e-03
p value ranksum for S+ high Low5 Proficient vs S+ low Low5 Proficient = 2.896547e-03
p value ranksum for S+ high Hi3 Proficient vs S+ high Low4 Proficient = 3.056348e-03
p value ranksum for S+ high Hi1 Naive vs S+ high Low4 Proficient = 3.118318e-03
p value ranksum for S+ high Low6 Proficient vs S+ low Hi3 Proficient = 3.245706e-03
p value ranksum for S+ low Hi2 Proficient vs S+ low Low5 Proficient = 3.377819e-03
p value ranksum for S+ high Hi2 Proficient vs S+ low Low4 Naive = 4.521329e-03
p value ranksum for S+ low Hi2 Proficient vs S+ low Hi3 Naive = 4.547808e-03
p value ranksum for S+ high Hi3 Proficient vs S+ low Hi1 Proficient = 5.026919e-03
p value ranksum for S+ high Hi2 Proficient vs S+ low Low6 Proficient = 5.891593e-03
p value ranksum for S+ high Low4 Naive vs S+ low Low5 Naive = 7.342803e-03
p value ranksum for S+ high Hi2 Naive vs S+ high Hi2 Proficient = 7.936009e-03
p value ranksum for S+ high Low5 Proficient vs S+ low Hi3 Naive = 8.431294e-03
p value ranksum for S+ high Hi3 Proficient vs S+ high Low5 Proficient = 9.069664e-03
p value ranksum for S+ low Hi1 Proficient vs S+ low Hi3 Naive = 9.714294e-03
p value ranksum for S+ high Hi1 Naive vs S+ low Hi1 Proficient = 1.046933e-02
p value ranksum for S+ high Hi3 Proficient vs S+ low Hi2 Proficient = 1.097688e-02
p value ranksum for S+ high Low4 Naive vs S+ low Hi3 Naive = 1.165332e-02
p value ranksum for S+ high Hi3 Naive vs S+ low Low4 Proficient = 1.377732e-02
p value ranksum for S+ low Hi2 Proficient vs S+ low Low5 Naive = 1.489674e-02
p value ranksum for S+ low Low5 Naive vs S+ low Low6 Proficient = 1.497364e-02
p value ranksum for S+ high Hi2 Proficient vs S+ low Hi1 Proficient = 1.562091e-02
p value t-test for S+ low Low4 Naive vs S+ low Low5 Naive = 1.689252e-02
p value ranksum for S+ high Hi1 Naive vs S+ low Hi2 Proficient = 1.719082e-02
p value ranksum for S+ high Hi2 Naive vs S+ low Low5 Naive = 1.771532e-02
p value ranksum for S+ high Low6 Proficient vs S+ low Low4 Naive = 1.835047e-02
p value ranksum for S+ high Hi1 Proficient vs S+ low Low4 Proficient = 1.848310e-02
p value ranksum for S+ high Hi2 Proficient vs S+ low Hi2 Proficient = 2.018969e-02
p value ranksum for S+ high Hi1 Naive vs S+ high Low5 Proficient = 2.191289e-02
p value t-test for S+ high Low5 Naive vs S+ low Hi2 Naive = 2.284325e-02

p value ranksum for S+ high Hi2 Proficient vs S+ low Low6 Naive = 2.372468e-02
p value ranksum for S+ high Hi2 Proficient vs S+ high Low5 Proficient = 2.629902e-02

p values below are > pFDR

p value ranksum for S+ high Low6 Proficient vs S+ low Low6 Proficient = 3.257953e-02
p value ranksum for S+ high Hi2 Naive vs S+ high Hi3 Proficient = 3.291627e-02
p value ranksum for S+ low Low5 Naive vs S+ low Low6 Naive = 3.550026e-02
p value ranksum for S+ high Low4 Proficient vs S+ low Hi3 Proficient = 3.872283e-02
p value ranksum for S+ low Low5 Proficient vs S+ low Low6 Proficient = 3.970447e-02
p value ranksum for S+ high Hi1 Proficient vs S+ high Low6 Proficient = 4.298879e-02
p value ranksum for S+ low Hi1 Proficient vs S+ low Low5 Naive = 4.326467e-02
p value ranksum for S+ high Hi3 Naive vs S+ high Low6 Proficient = 4.582373e-02
p value ranksum for S+ high Hi1 Proficient vs S+ low Low5 Naive = 4.917173e-02
p value t-test for S+ high Low5 Naive vs S+ low Hi3 Naive = 4.929876e-02
p value ranksum for S+ low Hi2 Proficient vs S+ low Hi3 Proficient = 4.976715e-02
p value ranksum for S+ high Hi2 Naive vs S+ low Low5 Proficient = 5.297135e-02
p value ranksum for S+ low Low4 Proficient vs S+ low Low6 Proficient = 5.297135e-02
p value ranksum for S+ high Low6 Proficient vs S+ low Low6 Naive = 5.432510e-02
p value ranksum for S+ high Low4 Proficient vs S+ low Hi3 Naive = 5.743212e-02
p value ranksum for S+ high Hi1 Naive vs S+ low Low6 Proficient = 5.823628e-02
p value t-test for S+ low Low4 Naive vs S+ low Low4 Proficient = 5.910098e-02
p value ranksum for S+ high Low4 Proficient vs S+ low Low4 Naive = 6.329077e-02
p value ranksum for S+ high Hi1 Naive vs S+ low Low6 Naive = 7.021029e-02
p value ranksum for S+ high Low4 Naive vs S+ low Hi2 Naive = 7.166612e-02
p value ranksum for S+ high Hi2 Naive vs S+ high Low6 Proficient = 7.398714e-02
p value ranksum for S+ low Hi1 Proficient vs S+ low Hi3 Proficient = 7.533079e-02
p value ranksum for S+ high Hi3 Naive vs S+ high Low4 Proficient = 8.235087e-02
p value ranksum for S+ low Hi3 Naive vs S+ low Low4 Proficient = 8.422396e-02
p value ranksum for S+ high Hi3 Proficient vs S+ low Low6 Proficient = 8.989224e-02
p value ranksum for S+ low Hi3 Proficient vs S+ low Low5 Proficient = 9.427124e-02
p value ranksum for S+ low Low4 Proficient vs S+ low Low5 Naive = 1.011936e-01
p value ranksum for S+ high Hi1 Proficient vs S+ high Low4 Proficient = 1.057463e-01
p value ranksum for S+ low Hi1 Proficient vs S+ low Low4 Proficient = 1.192772e-01
p value ranksum for S+ low Low5 Proficient vs S+ low Low6 Naive = 1.262302e-01
p value ranksum for S+ high Low4 Proficient vs S+ low Low6 Proficient = 1.267895e-01
p value ranksum for S+ high Low5 Proficient vs S+ low Hi3 Proficient = 1.267895e-01
p value ranksum for S+ high Hi1 Proficient vs S+ low Hi1 Proficient = 1.304190e-01
p value ranksum for S+ high Low5 Proficient vs S+ low Low4 Proficient = 1.325289e-01
p value ranksum for S+ high Low5 Proficient vs S+ low Low5 Naive = 1.328270e-01
p value ranksum for S+ high Hi1 Naive vs S+ high Hi2 Naive = 1.335938e-01
p value ranksum for S+ high Hi3 Naive vs S+ high Hi3 Proficient = 1.368279e-01
p value ranksum for S+ high Hi3 Proficient vs S+ low Low6 Naive = 1.416970e-01
p value ranksum for S+ high Low6 Proficient vs S+ low Hi2 Proficient = 1.474687e-01
p value ranksum for S+ high Hi2 Naive vs S+ low Low4 Proficient = 1.480458e-01
p value ranksum for S+ low Hi2 Proficient vs S+ low Low4 Proficient = 1.503714e-01
p value ranksum for S+ high Low6 Proficient vs S+ low Hi3 Naive = 1.531480e-01
p value ranksum for S+ high Low5 Proficient vs S+ high Low6 Proficient = 1.569105e-01
p value ranksum for S+ high Hi1 Naive vs S+ low Low4 Naive = 1.652895e-01
p value ranksum for S+ high Low4 Proficient vs S+ low Low6 Naive = 1.745650e-01
p value ranksum for S+ high Hi2 Naive vs S+ low Hi3 Proficient = 1.771584e-01

p value ranksum for S+ high Hi1 Proficient vs S+ high Low5 Proficient = 1.978514e-01
p value ranksum for S+ high Hi3 Naive vs S+ low Hi1 Proficient = 1.978514e-01
p value ranksum for S+ high Low6 Proficient vs S+ low Hi1 Proficient = 2.021941e-01
p value ranksum for S+ low Hi3 Naive vs S+ low Low5 Naive = 2.100383e-01
p value ranksum for S+ low Hi1 Proficient vs S+ low Low4 Naive = 2.114500e-01
p value ranksum for S+ high Hi3 Proficient vs S+ low Hi3 Proficient = 2.125963e-01
p value ranksum for S+ low Low4 Proficient vs S+ low Low6 Naive = 2.135006e-01
p value ranksum for S+ high Hi2 Proficient vs S+ high Low4 Proficient = 2.202595e-01
p value ranksum for S+ high Hi3 Naive vs S+ low Hi2 Proficient = 2.202595e-01
p value ranksum for S+ high Hi3 Naive vs S+ low Low5 Proficient = 2.249515e-01
p value ranksum for S+ high Hi1 Proficient vs S+ low Hi2 Proficient = 2.329293e-01
p value ranksum for S+ high Hi3 Proficient vs S+ low Low4 Naive = 2.362707e-01
p value ranksum for S+ high Low5 Proficient vs S+ low Low4 Naive = 2.399781e-01
p value ranksum for S+ high Hi1 Naive vs S+ high Hi3 Naive = 2.419345e-01
p value ranksum for S+ high Hi2 Proficient vs S+ low Low4 Proficient = 2.419345e-01
p value ranksum for S+ high Hi3 Naive vs S+ high Low5 Proficient = 2.520345e-01
p value ranksum for S+ low Hi3 Proficient vs S+ low Low6 Naive = 2.607066e-01
p value ranksum for S+ high Hi1 Proficient vs S+ low Low5 Proficient = 2.624249e-01
p value ranksum for S+ high Hi2 Naive vs S+ high Low4 Proficient = 2.813119e-01
p value ranksum for S+ high Hi1 Naive vs S+ high Hi1 Proficient = 2.934555e-01
p value ranksum for S+ low Hi3 Proficient vs S+ low Low6 Proficient = 3.011008e-01
p value ranksum for S+ high Hi3 Naive vs S+ low Hi3 Proficient = 3.207898e-01
p value ranksum for S+ high Hi2 Proficient vs S+ low Hi3 Naive = 3.528696e-01
p value ranksum for S+ high Low4 Naive vs S+ high Low5 Naive = 3.615479e-01
p value ranksum for S+ low Hi1 Proficient vs S+ low Low6 Proficient = 3.691931e-01
p value ranksum for S+ low Hi2 Proficient vs S+ low Low4 Naive = 3.715210e-01
p value ranksum for S+ high Hi1 Proficient vs S+ high Hi3 Proficient = 3.847824e-01
p value ranksum for S+ high Low4 Proficient vs S+ low Hi2 Proficient = 4.327012e-01
p value ranksum for S+ high Hi2 Proficient vs S+ high Low6 Proficient = 4.412145e-01
p value ranksum for S+ high Low5 Proficient vs S+ low Low6 Proficient = 4.597767e-01
p value ranksum for S+ high Hi3 Naive vs S+ low Low6 Naive = 4.667595e-01
p value ranksum for S+ high Low4 Proficient vs S+ high Low5 Proficient = 4.877755e-01
p value ranksum for S+ high Hi3 Naive vs S+ low Low6 Proficient = 5.007999e-01
p value ranksum for S+ high Low4 Proficient vs S+ low Low5 Naive = 5.016782e-01
p value ranksum for S+ high Low4 Proficient vs S+ high Low6 Proficient = 5.260454e-01
p value t-test for S+ low Low4 Naive vs S+ low Low6 Naive = 5.289714e-01
p value ranksum for S+ low Hi2 Proficient vs S+ low Low6 Proficient = 5.300901e-01
p value t-test for S+ low Low4 Naive vs S+ low Low5 Proficient = 5.354251e-01
p value t-test for S+ low Low4 Naive vs S+ low Low6 Proficient = 5.489715e-01
p value ranksum for S+ high Hi1 Proficient vs S+ low Low6 Proficient = 5.505481e-01
p value ranksum for S+ high Low5 Proficient vs S+ low Low6 Naive = 5.856694e-01
p value ranksum for S+ high Low6 Proficient vs S+ low Low5 Naive = 5.959643e-01
p value ranksum for S+ high Low4 Proficient vs S+ low Low4 Proficient = 6.040507e-01
p value ranksum for S+ high Hi2 Naive vs S+ low Hi1 Proficient = 6.127075e-01
p value ranksum for S+ high Low4 Proficient vs S+ low Hi1 Proficient = 6.170564e-01
p value ranksum for S+ low Hi1 Proficient vs S+ low Low6 Naive = 6.220552e-01
p value ranksum for S+ high Hi1 Proficient vs S+ high Hi2 Naive = 6.257944e-01
p value ranksum for S+ low Hi3 Proficient vs S+ low Low4 Naive = 6.499708e-01
p value ranksum for S+ high Hi1 Naive vs S+ low Hi3 Proficient = 6.838331e-01
p value ranksum for S+ high Hi2 Naive vs S+ high Low5 Proficient = 6.944640e-01
p value ranksum for S+ high Hi3 Proficient vs S+ low Low5 Proficient = 6.959876e-01

p value ranksum for S+ high Hi2 Proficient vs S+ low Low5 Naive = 7.003507e-01
p value ranksum for S+ high Hi1 Naive vs S+ high Hi3 Proficient = 7.344641e-01
p value ranksum for S+ high Hi2 Naive vs S+ low Hi2 Proficient = 7.767781e-01
p value ranksum for S+ high Hi2 Naive vs S+ low Low4 Naive = 7.862653e-01
p value ranksum for S+ high Hi1 Proficient vs S+ low Low6 Naive = 7.977546e-01
p value ranksum for S+ high Low6 Proficient vs S+ low Low4 Proficient = 8.133589e-01
p value ranksum for S+ high Hi2 Naive vs S+ high Hi3 Naive = 8.277896e-01
p value ranksum for S+ low Hi2 Proficient vs S+ low Low6 Naive = 8.455862e-01
p value ranksum for S+ high Hi3 Naive vs S+ low Low4 Naive = 8.660627e-01
p value ranksum for S+ high Hi2 Naive vs S+ low Low6 Proficient = 8.925654e-01
p value ranksum for S+ high Hi1 Proficient vs S+ low Hi3 Proficient = 8.941959e-01
p value t-test for S+ high Low6 Naive vs S+ low Hi2 Naive = 9.101942e-01
p value ranksum for S+ high Hi1 Proficient vs S+ high Hi3 Naive = 9.203401e-01
p value ranksum for S+ low Hi1 Proficient vs S+ low Hi2 Proficient = 9.236148e-01
p value ranksum for S+ high Hi2 Naive vs S+ low Low6 Naive = 9.309414e-01
p value ranksum for S+ high Low5 Proficient vs S+ low Hi2 Proficient = 9.318070e-01
p value ranksum for S+ high Hi1 Naive vs S+ low Low5 Proficient = 9.482123e-01
p value ranksum for S+ low Low6 Naive vs S+ low Low6 Proficient = 9.650669e-01
p value ranksum for S+ high Hi1 Proficient vs S+ low Low4 Naive = 9.710101e-01
p value ranksum for S+ high Low5 Proficient vs S+ low Hi1 Proficient = 9.777925e-01
