# Supplementary material for: The Olfactory Bulb Facilitates Use of Category Bounds for Classification of Odorants in Different Intensity Groups
Source: Front Cell Neurosci. 2020 Dec 11;14:613635. doi: 10.3389/fncel.2020.613635 (PMC7759615; doi:10.3389/fncel.2020.613635)
Supplement: Supplementary file 8 [file Table_8.pdf]

Table S8. Generalized linear regression model for Figure 5G, beta auROC for tPRP.

auROC: auROC
group: S+: high vs. S+ low
perCorr: naïve vs. proficient
peak_trough: peak vs. trough
comp_group: between (1), within low (2) and within high (3)

Generalized linear regression model:

auROC~group+perCorr+peak_trough+comp_group+group*perCorr*peak_trough*comp_group

Distribution = Normal

Estimated Coefficients:

	Estimate	SE	tStat	pValue
(Intercept)	0.38416	0.0030	124.15	0
group_2	-0.0443	0.0042	-10.391	3.43e-25
perCorr_2	-0.2828	0.0044	-64.056	0
peak_trough_2	-0.0049	0.0043	-1.1256	0.26034
comp_group_2	-0.2964	0.0062	-47.586	0
comp_group_3	-0.2917	0.0062	-46.829	0
group_2:perCorr_2	0.0237	0.0060	3.9002	9.6586e-05
group_2:peak_trough_2	0.0066	0.0060	1.1048	0.26925
perCorr_2:peak_trough_2	-0.005	0.0062	-0.803	0.42199
group_2:comp_group_2	0.0351	0.0085	4.1236	3.7539e-05
group_2:comp_group_3	0.1333	0.0086	15.377	6.9188e-53
perCorr_2:comp_group_2	0.2538	0.0089	28.46	8.5225e-173
perCorr_2:comp_group_3	0.2432	0.0088	27.547	2.5526e-162
peak_trough_2:comp_group_2	-0.002	0.0088	-0.305	0.75994
peak_trough_2:comp_group_3	0.0075	0.0088	0.8522	0.39407
group_2:perCorr_2:peak_trough_2	-4.9e-05	0.0086	-0.005	0.99545
group_2:perCorr_2:comp_group_2	-0.019	0.0121	-1.572	0.11589
group_2:perCorr_2:comp_group_3	-0.096	0.0122	-7.907	2.8345e-15
group_2:peak_trough_2:comp_group_2	-0.015	0.0120	-1.320	0.1868
group_2:peak_trough_2:comp_group_3	-0.008	0.0122	-0.718	0.47236
perCorr_2:peak_trough_2:comp_group_2	0.0043	0.0126	0.3475	0.7282
perCorr_2:peak_trough_2:comp_group_3	0.0014	0.0124	0.1127	0.91023
group_2:perCorr_2:peak_trough_2:comp_group_2	0.0245	0.0172	1.425	0.15417
group_2:perCorr_2:peak_trough_2:comp_group_3	0.0056	0.0173	0.32831	0.74268

12800 observations, 12776 error degrees of freedom
Estimated Dispersion: 0.00889
F-statistic vs. constant model: 1.06e+03, p-value = 0

Ranksum or t-test for auROC peak for theta Beta

pFDR = 4.015152e-02

p value ranksum for S+ high between Proficient vs S+ low between Naive = 7.829228e-312
p value ranksum for S+ high between Proficient vs S+ high between Naive = 4.946774e-288
p value ranksum for S+ low between Proficient vs S+ low between Naive = 3.110070e-226
p value ranksum for S+ high between Naive vs S+ low between Proficient = 6.143911e-194
p value ranksum for S+ high between Proficient vs S+ low within low Proficient = 1.015631e-165
p value ranksum for S+ high between Proficient vs S+ low within low Naive = 2.740246e-162
p value ranksum for S+ high between Proficient vs S+ low within high Naive = 2.253341e-161
p value ranksum for S+ high within high Naive vs S+ high between Proficient = 2.797960e-151
p value ranksum for S+ high within low Naive vs S+ high between Proficient = 5.362066e-145
p value ranksum for S+ high within high Proficient vs S+ high between Proficient = 3.459014e-139
p value ranksum for S+ high within low Proficient vs S+ high between Proficient = 1.590949e-138
p value ranksum for S+ low within low Naive vs S+ low between Proficient = 1.636691e-125
p value ranksum for S+ high within high Naive vs S+ low between Proficient = 1.774511e-118
p value ranksum for S+ low within high Naive vs S+ low between Proficient = 7.877407e-118
p value ranksum for S+ low within low Proficient vs S+ low between Proficient = 3.496950e-117
p value ranksum for S+ high within low Naive vs S+ low between Proficient = 6.132797e-110
p value ranksum for S+ high between Proficient vs S+ low within high Proficient = 1.368893e-103
p value ranksum for S+ high within low Proficient vs S+ low between Proficient = 1.540932e-100
p value ranksum for S+ high within high Proficient vs S+ low between Proficient = 8.885324e-99
p value ranksum for S+ low within high Proficient vs S+ low between Proficient = 1.223270e-58
p value ranksum for S+ low within high Proficient vs S+ low between Naive = 3.031270e-33
p value ranksum for S+ low within high Proficient vs S+ low within low Naive = 2.381677e-32
p value ranksum for S+ high within high Naive vs S+ low within high Proficient = 5.627676e-32
p value ranksum for S+ high within low Naive vs S+ low within high Proficient = 7.749338e-29
p value ranksum for S+ low within high Proficient vs S+ low within high Naive = 8.767851e-28
p value ranksum for S+ low within low Proficient vs S+ low within high Proficient = 2.318867e-24
p value ranksum for S+ high between Naive vs S+ low within high Proficient = 2.987222e-22
p value ranksum for S+ high between Naive vs S+ low within low Naive = 3.478581e-20
p value ranksum for S+ high within high Naive vs S+ high between Naive = 8.695140e-19
p value ranksum for S+ high within high Proficient vs S+ low within high Proficient = 7.796372e-16
p value ranksum for S+ high within low Proficient vs S+ low within high Proficient = 2.013477e-15
p value ranksum for S+ high within low Naive vs S+ high between Naive = 9.607773e-13
p value ranksum for S+ low within low Naive vs S+ low between Naive = 9.992291e-10
p value ranksum for S+ high between Naive vs S+ low within high Naive = 8.167386e-09
p value ranksum for S+ high within high Naive vs S+ low between Naive = 1.281923e-07
p value ranksum for S+ high between Naive vs S+ low between Naive = 2.135067e-07
p value ranksum for S+ high within high Proficient vs S+ low within low Naive = 2.466570e-07
p value ranksum for S+ high within high Proficient vs S+ high within high Naive = 1.641282e-06
p value ranksum for S+ high within low Proficient vs S+ high between Naive = 2.693889e-06
p value ranksum for S+ low within low Proficient vs S+ low within low Naive = 6.484667e-06
p value ranksum for S+ high within high Naive vs S+ low within low Proficient = 1.574753e-05
p value ranksum for S+ high between Naive vs S+ low within low Proficient = 1.360366e-04
p value ranksum for S+ high within low Naive vs S+ low between Naive = 2.367020e-04
p value ranksum for S+ high within high Proficient vs S+ high between Naive = 1.930521e-03
p value ranksum for S+ high within high Proficient vs S+ high within low Naive = 2.414544e-03
p value ranksum for S+ high within low Proficient vs S+ high within high Proficient = 2.922648e-03

p value ranksum for S+ high within low Naive vs S+ low within low Proficient = 3.002405e-03
p value ranksum for S+ high between Proficient vs S+ low between Proficient = 3.025566e-03
p value ranksum for S+ high within high Proficient vs S+ low within high Naive = 3.378139e-03
p value ranksum for S+ high within low Proficient vs S+ low between Naive = 1.083664e-02
p value ranksum for S+ low within high Naive vs S+ low between Naive = 2.310525e-02
p value ranksum for S+ low within low Naive vs S+ low within high Naive = 3.119446e-02
p value ranksum for S+ high within high Naive vs S+ low within high Naive = 3.800178e-02

p values below are > pFDR

p value ranksum for S+ high within low Naive vs S+ low within low Naive = 4.504471e-02
p value ranksum for S+ high within low Proficient vs S+ low within low Naive = 7.816093e-02
p value ranksum for S+ low within low Proficient vs S+ low within high Naive = 9.426616e-02
p value ranksum for S+ high within low Naive vs S+ high within high Naive = 1.674298e-01
p value ranksum for S+ high within low Proficient vs S+ low within low Proficient = 1.704349e-01
p value ranksum for S+ high within low Proficient vs S+ high within high Naive = 2.728906e-01
p value ranksum for S+ high within low Naive vs S+ low within high Naive = 3.716854e-01
p value ranksum for S+ high within low Proficient vs S+ low within high Naive = 4.489826e-01
p value ranksum for S+ high within high Naive vs S+ low within low Naive = 5.211830e-01
p value ranksum for S+ high within high Proficient vs S+ low within low Proficient = 6.440095e-01
p value ranksum for S+ high within high Proficient vs S+ low between Naive = 8.429099e-01
p value ranksum for S+ low within low Proficient vs S+ low between Naive = 9.362364e-01
p value ranksum for S+ high within low Proficient vs S+ high within low Naive = 9.852325e-01

Ranksum or t-test for auROC trough for theta Beta

pFDR = 3.863636e-02

p value t-test for S+ high within low Naive vs S+ high between Proficient = 0
p value ranksum for S+ high between Proficient vs S+ low between Naive = 3.529242e-312
p value ranksum for S+ high between Proficient vs S+ high between Naive = 2.881124e-291
p value ranksum for S+ low between Proficient vs S+ low between Naive = 6.617463e-236
p value ranksum for S+ high between Naive vs S+ low between Proficient = 6.592165e-210
p value t-test for S+ high within low Naive vs S+ low between Proficient = 1.109737e-177
p value ranksum for S+ high between Proficient vs S+ low within low Proficient = 1.306962e-166
p value ranksum for S+ high between Proficient vs S+ low within low Naive = 1.123489e-161
p value ranksum for S+ high between Proficient vs S+ low within high Naive = 4.405457e-161
p value ranksum for S+ high within high Naive vs S+ high between Proficient = 1.181253e-150
p value ranksum for S+ high within low Proficient vs S+ high between Proficient = 3.554739e-140
p value ranksum for S+ high within high Proficient vs S+ high between Proficient = 5.461032e-138
p value ranksum for S+ low within low Proficient vs S+ low between Proficient = 7.041019e-128
p value ranksum for S+ low within low Naive vs S+ low between Proficient = 3.369034e-126
p value ranksum for S+ high within high Naive vs S+ low between Proficient = 2.195928e-121
p value ranksum for S+ low within high Naive vs S+ low between Proficient = 5.711633e-120
p value ranksum for S+ high within low Proficient vs S+ low between Proficient = 8.020556e-106
p value ranksum for S+ high between Proficient vs S+ low within high Proficient = 2.116372e-103
p value ranksum for S+ high within high Proficient vs S+ low between Proficient = 1.908895e-100

p value ranksum for S+ low within high Proficient vs S+ low between Proficient = 1.858904e-59
p value t-test for S+ high within low Naive vs S+ low within high Proficient = 5.152997e-47
p value ranksum for S+ high within high Naive vs S+ low within high Proficient = 1.151636e-37
p value ranksum for S+ low within high Proficient vs S+ low between Naive = 1.622008e-37
p value ranksum for S+ low within high Proficient vs S+ low within low Naive = 1.685021e-33
p value ranksum for S+ low within low Proficient vs S+ low within high Proficient = 1.851877e-32
p value ranksum for S+ low within high Proficient vs S+ low within high Naive = 9.145811e-30
p value ranksum for S+ high between Naive vs S+ low within high Proficient = 6.228806e-28
p value ranksum for S+ high within low Proficient vs S+ low within high Proficient = 2.685922e-22
p value ranksum for S+ high within high Proficient vs S+ low within high Proficient = 1.827227e-17
p value ranksum for S+ high within high Naive vs S+ high between Naive = 4.312895e-16
p value t-test for S+ high within low Naive vs S+ high between Naive = 6.002669e-16
p value ranksum for S+ high between Naive vs S+ low within low Naive = 8.537466e-11
p value ranksum for S+ high within high Proficient vs S+ high within high Naive = 3.930130e-09
p value ranksum for S+ high within high Proficient vs S+ high within low Naive = 4.187827e-09
p value t-test for S+ high within low Naive vs S+ low between Naive = 8.547504e-09
p value ranksum for S+ high within high Naive vs S+ low between Naive = 1.048008e-07
p value ranksum for S+ high within low Proficient vs S+ high between Naive = 3.277198e-07
p value ranksum for S+ high between Naive vs S+ low within low Proficient = 8.043828e-07
p value ranksum for S+ high within high Proficient vs S+ low within low Naive = 7.001088e-06
p value ranksum for S+ high between Naive vs S+ low within high Naive = 2.412480e-05
p value ranksum for S+ high between Naive vs S+ low between Naive = 2.690402e-05
p value ranksum for S+ high within low Proficient vs S+ high within high Proficient = 5.579152e-05
p value t-test for S+ high within low Naive vs S+ low within high Naive = 1.051615e-04
p value ranksum for S+ low within low Naive vs S+ low between Naive = 4.851787e-04
p value ranksum for S+ high within high Proficient vs S+ low within low Proficient = 2.020461e-03
p value ranksum for S+ high within high Proficient vs S+ low within high Naive = 2.651050e-03
p value ranksum for S+ high within low Proficient vs S+ low between Naive = 6.384380e-03
p value ranksum for S+ high within high Naive vs S+ low within low Proficient = 1.046603e-02
p value ranksum for S+ high within high Naive vs S+ low within high Naive = 1.357684e-02
p value t-test for S+ high within low Naive vs S+ low within low Naive = 2.597713e-02
p value ranksum for S+ high within high Proficient vs S+ low between Naive = 3.468395e-02

p values below are > pFDR

p value t-test for S+ high within low Naive vs S+ low within low Proficient = 3.954677e-02
p value ranksum for S+ low within low Proficient vs S+ low between Naive = 4.924654e-02
p value ranksum for S+ high within high Naive vs S+ low within low Naive = 8.989381e-02
p value ranksum for S+ low within high Naive vs S+ low between Naive = 9.836666e-02
p value ranksum for S+ high within low Proficient vs S+ high within high Naive = 1.126602e-01
p value ranksum for S+ high between Proficient vs S+ low between Proficient = 1.232473e-01
p value ranksum for S+ low within low Proficient vs S+ low within low Naive = 2.202491e-01
p value ranksum for S+ low within low Naive vs S+ low within high Naive = 2.700167e-01
p value ranksum for S+ high within high Proficient vs S+ high between Naive = 3.637122e-01
p value ranksum for S+ low within low Proficient vs S+ low within high Naive = 4.404019e-01
p value ranksum for S+ high within low Proficient vs S+ low within low Proficient = 4.491234e-01
p value ranksum for S+ high within low Proficient vs S+ high within low Naive = 4.595107e-01
p value t-test for S+ high within low Naive vs S+ high within high Naive = 7.737662e-01
p value ranksum for S+ high within low Proficient vs S+ low within high Naive = 9.441969e-01
p value ranksum for S+ high within low Proficient vs S+ low within low Naive = 9.523776e-01
