# Supplementary material for: The Olfactory Bulb Facilitates Use of Category Bounds for Classification of Odorants in Different Intensity Groups
Source: Front Cell Neurosci. 2020 Dec 11;14:613635. doi: 10.3389/fncel.2020.613635 (PMC7759615; doi:10.3389/fncel.2020.613635)
Supplement: Supplementary file 9 [file Table_9.pdf]

Table S9. Generalized linear regression model for Figure 5G, beta auROC for tPRP.

auROC: auROC
group: S+: high vs. S+ low
perCorr: naïve vs. proficient
peak_trough: peak vs. trough
comp_group: between (1), within low (2) and within high (3)

Generalized linear regression model:

auROC~group+perCorr+peak_trough+comp_group+group*perCorr*peak_trough*comp_group

Distribution = Normal

Estimated Coefficients:

	Estimate	SE	tStat	pValue
(Intercept)	0.40848	0.0031	128.79	0
group_2	-0.0625	0.0043	-14.286	6.0313e-46
perCorr_2	-0.2820	0.0045	-62.334	0
peak_trough_2	-0.2561	0.0044	-57.114	0
comp_group_2	-0.3109	0.0063	-48.709	0
comp_group_3	-0.3243	0.0063	-50.798	0
group_2:perCorr_2	0.04213	0.0062	6.7599	1.4404e-11
group_2:peak_trough_2	0.10422	0.0061	16.83	7.0278e-63
perCorr_2:peak_trough_2	0.16792	0.0063	26.24	7.4076e-148
group_2:comp_group_2	0.06139	0.0087	7.0234	2.2751e-12
group_2:comp_group_3	0.14085	0.0088	15.841	5.4962e-56
perCorr_2:comp_group_2	0.25808	0.0091	28.226	4.3379e-170
perCorr_2:comp_group_3	0.24536	0.0090	27.114	1.8502e-157
peak_trough_2:comp_group_2	0.21214	0.0090	23.495	1.556e-119
peak_trough_2:comp_group_3	0.22993	0.0090	25.465	1.4191e-139
group_2:perCorr_2:peak_trough_2	-0.0716	0.0088	-8.1334	4.5561e-16
group_2:perCorr_2:comp_group_2	-0.0679	0.0125	-5.437	5.5173e-08
group_2:perCorr_2:comp_group_3	-0.0812	0.0125	-6.4832	9.3102e-11
group_2:peak_trough_2:comp_group_2	-0.0724	0.0123	-5.8628	4.6647e-09
group_2:peak_trough_2:comp_group_3	-0.1120	0.0125	-8.9105	5.762e-19
perCorr_2:peak_trough_2:comp_group_2	-0.1549	0.0129	-11.984	6.4422e-33
perCorr_2:peak_trough_2:comp_group_3	-0.1441	0.0127	-11.26	2.6596e-29
group_2:perCorr_2:peak_trough_2:comp_group_2	0.06891	0.0176	3.8984	9.7341e-05
group_2:perCorr_2:peak_trough_2:comp_group_3	0.03212	0.0177	1.8114	0.070103

12800 observations, 12776 error degrees of freedom
Estimated Dispersion: 0.00933
F-statistic vs. constant model: 744, p-value = 0

Ranksum or t-test for auROC peak for theta High gamma

pFDR = 4.545455e-02

p value ranksum for S+ high between Proficient vs S+ low between Naive = 4.562668e-306
p value ranksum for S+ high between Proficient vs S+ high between Naive = 2.002156e-289
p value ranksum for S+ low between Proficient vs S+ low between Naive = 3.666292e-207
p value ranksum for S+ high between Naive vs S+ low between Proficient = 1.137175e-176
p value ranksum for S+ high between Proficient vs S+ low within low Proficient = 2.211818e-164
p value ranksum for S+ high between Proficient vs S+ low within low Naive = 3.072166e-161
p value ranksum for S+ high between Proficient vs S+ low within high Naive = 1.890894e-160
p value ranksum for S+ high within high Naive vs S+ high between Proficient = 7.181943e-151
p value ranksum for S+ high within high Proficient vs S+ high between Proficient = 1.502562e-144
p value ranksum for S+ high within low Naive vs S+ high between Proficient = 4.339316e-144
p value ranksum for S+ high within low Proficient vs S+ high between Proficient = 4.046900e-139
p value ranksum for S+ low within low Naive vs S+ low between Proficient = 5.925188e-129
p value ranksum for S+ high within high Naive vs S+ low between Proficient = 1.310680e-123
p value ranksum for S+ high between Proficient vs S+ low within high Proficient = 4.785913e-123
p value ranksum for S+ low within low Proficient vs S+ low between Proficient = 4.682596e-111
p value ranksum for S+ low within high Naive vs S+ low between Proficient = 1.779146e-110
p value ranksum for S+ high within low Naive vs S+ low between Proficient = 1.441805e-105
p value ranksum for S+ high within high Proficient vs S+ low between Proficient = 2.347875e-104
p value ranksum for S+ high within low Proficient vs S+ low between Proficient = 2.785470e-98
p value ranksum for S+ low within high Proficient vs S+ low between Proficient = 2.282857e-67
p value ranksum for S+ high within high Naive vs S+ high between Naive = 2.105681e-53
p value ranksum for S+ high between Naive vs S+ low within low Naive = 1.653478e-50
p value ranksum for S+ low within low Naive vs S+ low between Naive = 2.209753e-34
p value ranksum for S+ high within high Naive vs S+ low between Naive = 5.094242e-31
p value ranksum for S+ low within high Proficient vs S+ low within low Naive = 9.003016e-27
p value ranksum for S+ high within high Naive vs S+ low within high Proficient = 4.100306e-26
p value ranksum for S+ high within low Naive vs S+ high between Naive = 4.601411e-21
p value ranksum for S+ high within high Proficient vs S+ high between Naive = 1.576541e-18
p value ranksum for S+ low within low Proficient vs S+ low within low Naive = 6.125409e-18
p value ranksum for S+ high within high Naive vs S+ low within low Proficient = 6.352039e-17
p value ranksum for S+ high within low Naive vs S+ low within high Proficient = 1.955019e-16
p value ranksum for S+ high within low Proficient vs S+ high between Naive = 2.556081e-14
p value ranksum for S+ high within high Naive vs S+ low within high Naive = 1.203775e-13
p value ranksum for S+ low within high Proficient vs S+ low within high Naive = 2.380379e-13
p value ranksum for S+ low within low Naive vs S+ low within high Naive = 3.230791e-13
p value ranksum for S+ high between Proficient vs S+ low between Proficient = 1.057753e-12
p value ranksum for S+ high within high Proficient vs S+ low within high Proficient = 1.170076e-12
p value ranksum for S+ high between Naive vs S+ low within high Naive = 3.280534e-11
p value ranksum for S+ high within low Proficient vs S+ low within low Naive = 5.808600e-11
p value ranksum for S+ low within high Proficient vs S+ low between Naive = 7.149255e-11
p value ranksum for S+ low within low Proficient vs S+ low within high Proficient = 2.069262e-10
p value ranksum for S+ high between Naive vs S+ low between Naive = 1.424247e-09
p value ranksum for S+ high between Naive vs S+ low within low Proficient = 1.270719e-08
p value ranksum for S+ high within high Proficient vs S+ low within low Naive = 2.066804e-08
p value ranksum for S+ high within low Proficient vs S+ low within high Proficient = 2.503299e-08
p value ranksum for S+ high within low Naive vs S+ low within low Naive = 8.756921e-08

p value ranksum for S+ high within low Naive vs S+ low between Naive = 1.321779e-07
p value ranksum for S+ high within high Proficient vs S+ low between Naive = 2.984430e-07
p value ranksum for S+ high within low Naive vs S+ high within high Naive = 1.514524e-06
p value ranksum for S+ high within low Proficient vs S+ low between Naive = 1.077586e-05
p value ranksum for S+ high within low Proficient vs S+ high within high Naive = 1.541372e-05
p value ranksum for S+ high between Naive vs S+ low within high Proficient = 2.090862e-05
p value ranksum for S+ high within high Proficient vs S+ high within high Naive = 3.161059e-05
p value ranksum for S+ high within low Naive vs S+ low within low Proficient = 7.501106e-04
p value ranksum for S+ high within high Proficient vs S+ low within low Proficient = 9.726340e-04
p value ranksum for S+ high within low Proficient vs S+ low within low Proficient = 7.756311e-03
p value ranksum for S+ high within high Naive vs S+ low within low Naive = 9.953599e-03
p value ranksum for S+ high within low Naive vs S+ low within high Naive = 1.764151e-02
p value ranksum for S+ high within high Proficient vs S+ low within high Naive = 3.377201e-02
p value ranksum for S+ low within high Naive vs S+ low between Naive = 4.369190e-02

p values below are > pFDR

p value ranksum for S+ high within low Proficient vs S+ low within high Naive = 1.430984e-01
p value ranksum for S+ low within low Proficient vs S+ low between Naive = 1.765837e-01
p value ranksum for S+ low within low Proficient vs S+ low within high Naive = 3.646225e-01
p value ranksum for S+ high within low Proficient vs S+ high within high Proficient = 4.686241e-01
p value ranksum for S+ high within high Proficient vs S+ high within low Naive = 8.435513e-01
p value ranksum for S+ high within low Proficient vs S+ high within low Naive = 8.836119e-01

Ranksum or t-test for auROC trough for theta High gamma

pFDR = 3.560606e-02

p value ranksum for S+ high between Naive vs S+ low between Proficient = 7.847335e-143
p value ranksum for S+ low between Proficient vs S+ low between Naive = 7.146886e-134
p value ranksum for S+ low within high Naive vs S+ low between Proficient = 1.454382e-80
p value ranksum for S+ high between Proficient vs S+ high between Naive = 1.856296e-80
p value ranksum for S+ low within low Naive vs S+ low between Proficient = 2.681877e-73
p value ranksum for S+ high within high Naive vs S+ low between Proficient = 6.222026e-67
p value ranksum for S+ high within low Naive vs S+ low between Proficient = 1.043572e-63
p value ranksum for S+ high within low Proficient vs S+ low between Proficient = 3.208438e-59
p value ranksum for S+ high within high Proficient vs S+ low between Proficient = 5.226671e-56
p value ranksum for S+ high between Proficient vs S+ low between Naive = 1.105470e-53
p value ranksum for S+ high between Naive vs S+ low within high Proficient = 3.638664e-46
p value ranksum for S+ high between Proficient vs S+ low within high Naive = 3.944018e-46
p value ranksum for S+ low within low Proficient vs S+ low between Proficient = 6.821428e-39
p value ranksum for S+ low within high Proficient vs S+ low between Naive = 1.458620e-35
p value ranksum for S+ high within high Naive vs S+ high between Proficient = 2.798241e-35
p value ranksum for S+ low within high Proficient vs S+ low within high Naive = 1.378683e-34
p value ranksum for S+ high between Proficient vs S+ low within low Naive = 4.643725e-34
p value ranksum for S+ high within low Naive vs S+ high between Proficient = 1.662334e-32
p value ranksum for S+ low within high Proficient vs S+ low within low Naive = 1.653354e-28

p value ranksum for S+ high within high Proficient vs S+ high between Proficient = 4.413469e-28
p value ranksum for S+ high within high Naive vs S+ low within high Proficient = 2.104080e-27
p value ranksum for S+ high between Naive vs S+ low within low Proficient = 1.185496e-26
p value ranksum for S+ high within low Naive vs S+ low within high Proficient = 1.699843e-26
p value ranksum for S+ high within low Proficient vs S+ high between Proficient = 7.540880e-26
p value ranksum for S+ high within low Proficient vs S+ low within high Proficient = 1.376202e-21
p value ranksum for S+ low within low Proficient vs S+ low within high Naive = 3.745802e-21
p value ranksum for S+ high within high Proficient vs S+ low within high Proficient = 1.410959e-20
p value ranksum for S+ low within low Proficient vs S+ low between Naive = 1.079477e-14
p value ranksum for S+ high within high Naive vs S+ low within low Proficient = 3.000153e-14
p value ranksum for S+ low within low Proficient vs S+ low within low Naive = 7.297341e-14
p value ranksum for S+ high between Proficient vs S+ low between Proficient = 9.646084e-14
p value ranksum for S+ low within high Proficient vs S+ low between Proficient = 2.495077e-13
p value ranksum for S+ high within low Naive vs S+ low within low Proficient = 4.621683e-13
p value ranksum for S+ high between Naive vs S+ low between Naive = 2.409790e-12
p value ranksum for S+ high within high Proficient vs S+ low within low Proficient = 4.802110e-10
p value ranksum for S+ high within low Proficient vs S+ low within low Proficient = 1.137724e-08
p value ranksum for S+ high between Proficient vs S+ low within low Proficient = 4.702505e-08
p value ranksum for S+ low within low Proficient vs S+ low within high Proficient = 9.399273e-08
p value ranksum for S+ low within high Naive vs S+ low between Naive = 3.968507e-07
p value ranksum for S+ high within low Proficient vs S+ high between Naive = 2.168380e-05
p value ranksum for S+ high between Naive vs S+ low within low Naive = 1.728469e-04
p value ranksum for S+ high within high Proficient vs S+ high between Naive = 2.501778e-04
p value ranksum for S+ high within low Proficient vs S+ low within high Naive = 5.113225e-04
p value ranksum for S+ high within high Naive vs S+ low between Naive = 2.843562e-03
p value ranksum for S+ low within low Naive vs S+ low within high Naive = 3.067042e-03
p value ranksum for S+ high within low Naive vs S+ low between Naive = 6.342524e-03
p value ranksum for S+ high within high Proficient vs S+ low within high Naive = 1.325553e-02

p values below are > pFDR

p value ranksum for S+ high within high Naive vs S+ high between Naive = 3.736122e-02
p value ranksum for S+ high within low Proficient vs S+ high within high Naive = 5.054908e-02
p value ranksum for S+ high within low Proficient vs S+ high within low Naive = 5.265836e-02
p value ranksum for S+ high within low Naive vs S+ high between Naive = 9.101314e-02
p value ranksum for S+ low within low Naive vs S+ low between Naive = 1.000968e-01
p value ranksum for S+ high within high Proficient vs S+ low between Naive = 1.319718e-01
p value ranksum for S+ high within high Proficient vs S+ high within low Naive = 1.732740e-01
p value ranksum for S+ high within high Proficient vs S+ high within high Naive = 1.969239e-01
p value ranksum for S+ high within high Naive vs S+ low within low Naive = 1.982043e-01
p value ranksum for S+ high within high Naive vs S+ low within high Naive = 2.398444e-01
p value ranksum for S+ high within low Naive vs S+ low within low Naive = 2.548051e-01
p value ranksum for S+ high within low Proficient vs S+ low within low Naive = 2.986947e-01
p value ranksum for S+ high within low Naive vs S+ low within high Naive = 2.994369e-01
p value ranksum for S+ high between Naive vs S+ low within high Naive = 3.204784e-01
p value ranksum for S+ high between Proficient vs S+ low within high Proficient = 4.507840e-01
p value ranksum for S+ high within low Proficient vs S+ high within high Proficient = 4.843503e-01
p value ranksum for S+ high within low Proficient vs S+ low between Naive = 8.126334e-01
p value ranksum for S+ high within high Proficient vs S+ low within low Naive = 9.169751e-01
p value ranksum for S+ high within low Naive vs S+ high within high Naive = 9.346706e-01
