# Supplementary material for: The Olfactory Bulb Facilitates Use of Category Bounds for Classification of Odorants in Different Intensity Groups
Source: Front Cell Neurosci. 2020 Dec 11;14:613635. doi: 10.3389/fncel.2020.613635 (PMC7759615; doi:10.3389/fncel.2020.613635)
Supplement: Supplementary file 10 [file Table_10.pdf]

Table S10. Generalized linear regression model for Figure 6C, AUC for decoding with beta tPRP.

auc: AUC
naive_prof_sh: naïve (1), proficient (2) and shuffled (3)
rewarded_stimulus: S+ high vs. S+ low
peak_trough: peak vs. trough

Generalized linear regression model:

auc~naive_prof_sh+rewarded_stimulus+peak_trough+peak_trough*naive_prof_sh*rewarded_stimulus

Estimated Coefficients:

	Estimate	SE	tStat	pValue
(Intercept)	0.46571	0.0342	13.599	4.7402e-22
naive_prof_sh_2	-0.4081	0.0484	-8.428	1.6654e-12
naive_prof_sh_3	-0.4716	0.0484	-9.738	5.2102e-15
rewarded_stimulus_2	-0.1074	0.0468	-2.282	0.025249
peak_trough_1	0.0005	0.0484	0.0110	0.9912
naive_prof_sh_2:rewarded_stimulus_2	0.1072	0.0674	1.5904	0.11591
naive_prof_sh_3:rewarded_stimulus_2	0.1026	0.0663	1.5474	0.12591
naive_prof_sh_2:peak_trough_1	0.0331	0.0684	0.4838	0.62992
naive_prof_sh_3:peak_trough_1	-0.0005	0.0684	-0.0078	0.99378
rewarded_stimulus_2:peak_trough_1	0.01888	0.0663	0.2848	0.77657
naive_prof_sh_2:rewarded_stimulus_2:peak_trough_1	-0.0464	0.0953	-0.4870	0.62765
naive_prof_sh_3:rewarded_stimulus_2:peak_trough_1	-0.0188	0.0937	-0.2013	0.84093

88 observations, 76 error degrees of freedom
Estimated Dispersion: 0.00821
F-statistic vs. constant model: 34.3, p-value = 4.89e-25

Ranksum or t-test p values for auc for peak for Theta/Beta

pFDR = 3.666667e-02

p value t-test for S+ high proficient vs S+ low Shuffled = 9.095658e-08
p value t-test for S+ high Shuffled vs S+ low proficient = 3.073861e-05
p value t-test for S+ high proficient vs S+ high Shuffled = 6.727628e-05
p value t-test for S+ high naive vs S+ high proficient = 1.345256e-04
p value t-test for S+ low proficient vs S+ low Shuffled = 2.570850e-04
p value t-test for S+ high proficient vs S+ low naive = 3.898615e-04
p value t-test for S+ low naive vs S+ low proficient = 3.958057e-04
p value t-test for S+ high naive vs S+ low proficient = 7.749269e-04
p value t-test for S+ high naive vs S+ low Shuffled = 4.235160e-03
p value t-test for S+ high naive vs S+ high Shuffled = 1.244029e-02
p value t-test for S+ low naive vs S+ low Shuffled = 3.168130e-02

p values below are > pFDR

p value t-test for S+ high Shuffled vs S+ low naive = 4.201709e-02
p value t-test for S+ high proficient vs S+ low proficient = 2.555075e-01
p value t-test for S+ high naive vs S+ low naive = 4.935638e-01
p value t-test for S+ high Shuffled vs S+ low Shuffled = 8.115147e-01

Ranksum or t-test p values for auc for trough for Theta/Beta

pFDR = 4.000000e-02

p value t-test for S+ high proficient vs S+ low Shuffled = 1.015528e-08
p value t-test for S+ high proficient vs S+ high Shuffled = 2.097361e-05
p value t-test for S+ high Shuffled vs S+ low proficient = 4.481341e-05
p value t-test for S+ high naive vs S+ high proficient = 6.529532e-05
p value t-test for S+ high proficient vs S+ low naive = 7.335402e-05
p value t-test for S+ low proficient vs S+ low Shuffled = 3.059119e-04
p value t-test for S+ low naive vs S+ low proficient = 3.841502e-04
p value t-test for S+ high naive vs S+ low proficient = 4.092708e-04
p value t-test for S+ low naive vs S+ low Shuffled = 1.989186e-02
p value t-test for S+ high Shuffled vs S+ low naive = 2.207316e-02
p value t-test for S+ high naive vs S+ low Shuffled = 2.537644e-02
p value t-test for S+ high naive vs S+ high Shuffled = 3.317037e-02

p values below are > pFDR

p value t-test for S+ high proficient vs S+ low proficient = 1.449736e-01
p value t-test for S+ high Shuffled vs S+ low Shuffled = 8.115147e-01
p value t-test for S+ high naive vs S+ low naive = 9.952131e-01
