# Supplementary material for: The Olfactory Bulb Facilitates Use of Category Bounds for Classification of Odorants in Different Intensity Groups
Source: Front Cell Neurosci. 2020 Dec 11;14:613635. doi: 10.3389/fncel.2020.613635 (PMC7759615; doi:10.3389/fncel.2020.613635)
Supplement: Supplementary file 11 [file Table_11.pdf]

Table S11. Generalized linear regression model for Figure 6D, AUC for decoding with gamma tPRP.

auc: AUC
naive_prof_sh: naïve (1), proficient (2) and shuffled (3)
rewarded_stimulus: S+ high vs. S+ low
peak_trough: peak vs. trough

Generalized linear regression model:

auc~naive_prof_sh+rewarded_stimulus+peak_trough+peak_trough*naive_prof_sh*rewarded_stimulus

Distribution = Normal

Estimated Coefficients:

	Estimate	SE	tStat	pValue
(Intercept)	0.22606	0.03237	6.9821	9.4884e-10
naive_prof_sh_2	-0.1676	0.04578	-3.6614	0.00046118
naive_prof_sh_3	-0.2150	0.04578	-4.6962	1.1529e-05
rewarded_stimulus_2	-0.0110	0.04433	-0.2483	0.80456
peak_trough_1	0.28179	0.04578	6.1542	3.2759e-08
naive_prof_sh_2:rewarded_stimulus_2	-0.0345	0.06373	-0.5426	0.58893
naive_prof_sh_3:rewarded_stimulus_2	-0.0150	0.06269	-0.2404	0.81063
naive_prof_sh_2:peak_trough_1	-0.2463	0.06475	-3.8049	0.00028508
naive_prof_sh_3:peak_trough_1	-0.2817	0.06475	-4.3517	4.1537e-05
rewarded_stimulus_2:peak_trough_1	-0.1095	0.06269	-1.746	0.084751
naive_prof_sh_2:rewarded_stimulus_2:peak_trough_1	0.12785	0.09013	1.4184	0.16016
naive_prof_sh_3:rewarded_stimulus_2:peak_trough_1	0.10951	0.08866	1.235	0.22063

88 observations, 76 error degrees of freedom
Estimated Dispersion: 0.00734
F-statistic vs. constant model: 29.2, p-value = 6.88e-23

Ranksum or t-test p values for auc for peak for Theta/High Gamma

pFDR = 4.000000e-02

p value t-test for S+ high proficient vs S+ low Shuffled = 2.973977e-10
p value t-test for S+ high proficient vs S+ low naive = 8.815079e-06
p value t-test for S+ high proficient vs S+ high Shuffled = 1.227773e-05
p value t-test for S+ high Shuffled vs S+ low proficient = 5.925143e-05
p value t-test for S+ low proficient vs S+ low Shuffled = 2.547504e-04
p value ranksum for S+ high naive vs S+ low Shuffled = 3.108003e-04

p value t-test for S+ low naive vs S+ low proficient = 3.265346e-04
p value ranksum for S+ high naive vs S+ high proficient = 5.827506e-04
p value ranksum for S+ high naive vs S+ high Shuffled = 5.827506e-04
p value ranksum for S+ high naive vs S+ low proficient = 6.216006e-04
p value t-test for S+ low naive vs S+ low Shuffled = 1.282605e-03
p value t-test for S+ high Shuffled vs S+ low naive = 3.267493e-02

p values below are > pFDR

p value t-test for S+ high Shuffled vs S+ low Shuffled = 6.554883e-02
p value t-test for S+ high proficient vs S+ low proficient = 1.105860e-01
p value ranksum for S+ high naive vs S+ low naive = 1.649184e-01

Ranksum or t-test p values for auc for trough for Theta/High Gamma

pFDR = 3.000000e-02

p value t-test for S+ high proficient vs S+ low Shuffled = 9.566958e-07
p value t-test for S+ high proficient vs S+ low naive = 3.978992e-04
p value t-test for S+ high proficient vs S+ high Shuffled = 5.643952e-04
p value t-test for S+ high naive vs S+ low Shuffled = 2.170073e-03
p value t-test for S+ high naive vs S+ high proficient = 4.054752e-03
p value t-test for S+ low proficient vs S+ low Shuffled = 8.527486e-03
p value t-test for S+ high Shuffled vs S+ low proficient = 9.886924e-03
p value t-test for S+ low naive vs S+ low proficient = 1.112702e-02
p value t-test for S+ high naive vs S+ low naive = 2.764568e-02

p values below are > pFDR

p value t-test for S+ high naive vs S+ low proficient = 4.107863e-02
p value t-test for S+ high Shuffled vs S+ low Shuffled = 6.554883e-02
p value t-test for S+ high naive vs S+ high Shuffled = 8.384816e-02
p value t-test for S+ low naive vs S+ low Shuffled = 1.180108e-01
p value t-test for S+ high proficient vs S+ low proficient = 8.807808e-01
p value t-test for S+ high Shuffled vs S+ low naive = 9.165773e-01
