# Supplementary material for: The Olfactory Bulb Facilitates Use of Category Bounds for Classification of Odorants in Different Intensity Groups
Source: Front Cell Neurosci. 2020 Dec 11;14:613635. doi: 10.3389/fncel.2020.613635 (PMC7759615; doi:10.3389/fncel.2020.613635)
Supplement: Supplementary file 12 [file Table_12.pdf]

Table S12. Generalized linear regression model for Figure 6G, decision times for decoding with beta tPRP.

decision_time: decision time
naive_prof_sh: naïve (1), proficient (2) and shuffled (3)
rewarded_stimulus: S+ high vs. S+ low
peak_trough: peak vs. trough

Generalized linear regression model:

decision_time~naive_prof_sh+rewarded_stimulus+peak_trough_lick+peak_trough_lick*naive_prof_sh*rewarded_stimulus

Distribution = Normal

Estimated Coefficients:

	Estimate	SE	tStat	pValue
(Intercept)	0.6	0.2866	2.0934	0.039692
naive_prof_sh_2	4.285	0.4053	10.573	1.6188e-16
rewarded_stimulus_2	0.525	0.3924	1.3377	0.18503
peak_trough_lick_1	-0.171	0.4053	-0.4229	0.67355
peak_trough_lick_2	0.0285	0.4053	0.0704	0.94399
naive_prof_sh_2:rewarded_stimulus_2	-1.910	0.5642	-3.386	0.0011294
naive_prof_sh_2:peak_trough_lick_1	-2.557	0.5732	-4.461	2.8199e-05
naive_prof_sh_2:peak_trough_lick_2	-1.857	0.5732	-3.2398	0.001784
rewarded_stimulus_2:peak_trough_lick_1	-0.466	0.5550	-0.8397	0.40373
rewarded_stimulus_2:peak_trough_lick_2	0.8589	0.5550	1.5476	0.12594
naive_prof_sh_2:rewarded_stimulus_2:peak_trough_lick_1	2.8804	0.7978	3.61	0.000550
naive_prof_sh_2:rewarded_stimulus_2:peak_trough_lick_2	0.5410	0.7978	0.67813	0.49978

87 observations, 75 error degrees of freedom
Estimated Dispersion: 0.575
F-statistic vs. constant model: 26.6, p-value = 1.55e-21

pFDR = 3.571429e-02

p value t-test for S+ high proficient licks vs S+ high naive peak = 5.950289e-12
p value t-test for S+ low proficient licks vs S+ low naive licks = 3.519123e-08
p value t-test for S+ low proficient licks vs S+ low naive peak = 3.439264e-06
p value t-test for S+ high proficient licks vs S+ low naive licks = 2.544611e-05
p value t-test for S+ low proficient peak vs S+ low naive licks = 7.184731e-05
p value t-test for S+ low proficient peak vs S+ low naive peak = 2.574242e-04
p value t-test for S+ high proficient licks vs S+ low naive peak = 3.055757e-04
p value ranksum for S+ high naive peak vs S+ low proficient licks = 3.108003e-04
p value ranksum for S+ high naive peak vs S+ low proficient peak = 3.108003e-04

p value ranksum for S+ high naive peak vs S+ low naive licks = 5.827506e-04
p value ranksum for S+ high proficient peak vs S+ high naive peak = 5.827506e-04
p value ranksum for S+ high naive peak vs S+ low naive peak = 5.827506e-04
p value ranksum for S+ high proficient peak vs S+ low naive licks = 5.827506e-04
p value ranksum for S+ high proficient peak vs S+ low naive peak = 1.165501e-03
p value t-test for S+ high naive licks vs S+ high naive peak = 4.266356e-03
p value ranksum for S+ high proficient peak vs S+ high naive licks = 7.575758e-03
p value t-test for S+ high naive licks vs S+ low proficient licks = 1.253333e-02
p value t-test for S+ high proficient licks vs S+ low proficient peak = 2.764386e-02
p value t-test for S+ low proficient licks vs S+ low proficient peak = 2.943481e-02
p value t-test for S+ high proficient licks vs S+ high naive licks = 3.227294e-02

p values below are > pFDR

p value ranksum for S+ high proficient peak vs S+ low proficient peak = 5.097125e-02
p value t-test for S+ high naive licks vs S+ low naive peak = 6.137032e-02
p value ranksum for S+ low naive licks vs S+ low naive peak = 6.293706e-02
p value t-test for S+ high naive licks vs S+ low naive licks = 1.279047e-01
p value t-test for S+ high naive licks vs S+ low proficient peak = 1.300334e-01
p value t-test for S+ high proficient licks vs S+ low proficient licks = 3.547780e-01
p value t-test for S+ high proficient licks vs S+ high proficient peak = 3.559177e-01
p value ranksum for S+ high proficient peak vs S+ low proficient licks = 8.907537e-01

pFDR = 3.035714e-02

p value t-test for S+ high proficient licks vs S+ low proficient trough = 1.346615e-16
p value t-test for S+ low proficient licks vs S+ low proficient trough = 1.496850e-08
p value t-test for S+ low proficient licks vs S+ low naive licks = 3.519123e-08
p value t-test for S+ low proficient licks vs S+ low naive trough = 1.058953e-06
p value t-test for S+ high proficient licks vs S+ low naive licks = 2.544611e-05
p value t-test for S+ high proficient licks vs S+ low naive trough = 1.571071e-04
p value ranksum for S+ high proficient trough vs S+ low proficient trough = 3.108003e-04
p value ranksum for S+ high naive trough vs S+ low proficient licks = 3.108003e-04
p value t-test for S+ high proficient licks vs S+ high naive trough = 4.142242e-04
p value ranksum for S+ high proficient trough vs S+ low naive licks = 1.165501e-03
p value ranksum for S+ high proficient trough vs S+ low naive trough = 1.165501e-03
p value ranksum for S+ high proficient trough vs S+ high naive trough = 1.165501e-03
p value ranksum for S+ low proficient trough vs S+ low naive trough = 9.324009e-03
p value ranksum for S+ high proficient trough vs S+ high naive licks = 9.324009e-03
p value ranksum for S+ low proficient trough vs S+ low naive licks = 1.149961e-02
p value t-test for S+ high naive licks vs S+ low proficient licks = 1.253333e-02
p value ranksum for S+ high naive trough vs S+ low proficient trough = 1.367521e-02

p values below are > pFDR

p value t-test for S+ high proficient licks vs S+ high naive licks = 3.227294e-02
p value t-test for S+ high naive licks vs S+ low naive licks = 1.279047e-01
p value t-test for S+ high naive licks vs S+ low naive trough = 1.332301e-01
p value t-test for S+ high naive licks vs S+ high naive trough = 1.708237e-01
p value ranksum for S+ high naive trough vs S+ low naive licks = 2.179487e-01

p value t-test for S+ high proficient licks vs S+ high proficient trough = 3.215801e-01
p value t-test for S+ high proficient licks vs S+ low proficient licks = 3.547780e-01
p value t-test for S+ high naive licks vs S+ low proficient trough = 8.050793e-01
p value ranksum for S+ low naive licks vs S+ low naive trough = 9.114219e-01
p value ranksum for S+ high naive trough vs S+ low naive trough = 9.417249e-01
p value ranksum for S+ high proficient trough vs S+ low proficient licks = 1
