# Supplementary material for: The Olfactory Bulb Facilitates Use of Category Bounds for Classification of Odorants in Different Intensity Groups
Source: Front Cell Neurosci. 2020 Dec 11;14:613635. doi: 10.3389/fncel.2020.613635 (PMC7759615; doi:10.3389/fncel.2020.613635)
Supplement: Supplementary file 13 [file Table_13.pdf]

Table S13. Generalized linear regression model for Figure 6H, decision times for decoding with gamma tPRP.

decision_time: decision time
naive_prof_sh: naïve (1), proficient (2) and shuffled (3)
rewarded_stimulus: S+ high vs. S+ low
peak_trough: peak vs. trough

Generalized linear regression model:

decision_time~naive_prof_sh+rewarded_stimulus+peak_trough_lick+peak_trough_lick*naive_prof_sh*rewarded_stimulus

Distribution = Normal

Estimated Coefficients:

	Estimate	SE	tStat	pValue
(Intercept)	0.4428	0.36618	1.2094	0.23031
naive_prof_sh_2	3.1143	0.51785	6.0138	6.118e-08
rewarded_stimulus_2	0.76964	0.50141	1.535	0.129
peak_trough_lick_1	-0.014	0.5178	-0.0275	0.97807
peak_trough_lick_2	0.4285	0.5178	0.82759	0.41053
naive_prof_sh_2:rewarded_stimulus_2	-1.226	0.7208	-1.7019	0.092911
naive_prof_sh_2:peak_trough_lick_1	-1.385	0.7323	-1.892	0.062333
naive_prof_sh_2:peak_trough_lick_2	0.9142	0.7323	1.2484	0.21576
rewarded_stimulus_2:peak_trough_lick_1	-0.710	0.7091	-1.0023	0.31943
rewarded_stimulus_2:peak_trough_lick_2	1.3339	0.7091	1.8812	0.063832
naive_prof_sh_2:rewarded_stimulus_2:peak_trough_lick_1	2.1964	1.0194	2.1546	0.034401
naive_prof_sh_2:rewarded_stimulus_2:peak_trough_lick_2	-1.3482	1.0194	-1.3226	0.19

87 observations, 75 error degrees of freedom
Estimated Dispersion: 0.939
F-statistic vs. constant model: 19.6, p-value = 7.38e-18

Ranksum or t-test p values for decision times for peak for Theta/High Gamma

pFDR = 3.392857e-02

p value t-test for S+ high proficient licks vs S+ high naive peak = 1.443960e-09
p value t-test for S+ low proficient licks vs S+ low naive licks = 3.519123e-08
p value t-test for S+ low proficient licks vs S+ low naive peak = 4.337943e-06
p value t-test for S+ high proficient licks vs S+ low naive licks = 2.544611e-05
p value t-test for S+ low proficient peak vs S+ low naive licks = 1.014191e-04
p value ranksum for S+ high naive peak vs S+ low proficient licks = 3.108003e-04
p value ranksum for S+ high naive peak vs S+ low proficient peak = 3.108003e-04

p value t-test for S+ high proficient licks vs S+ low naive peak = 3.287776e-04
p value ranksum for S+ high proficient peak vs S+ low naive licks = 5.827506e-04
p value ranksum for S+ high proficient peak vs S+ low naive peak = 5.827506e-04
p value ranksum for S+ high proficient peak vs S+ high naive peak = 5.827506e-04
p value t-test for S+ low proficient peak vs S+ low naive peak = 8.845277e-04
p value ranksum for S+ high proficient peak vs S+ high naive licks = 2.331002e-03
p value ranksum for S+ high proficient peak vs S+ low proficient peak = 5.283605e-03
p value ranksum for S+ high naive peak vs S+ low naive licks = 1.048951e-02
p value t-test for S+ high naive licks vs S+ low proficient licks = 1.253333e-02
p value t-test for S+ high proficient licks vs S+ low proficient peak = 1.438688e-02
p value t-test for S+ low proficient licks vs S+ low proficient peak = 1.821618e-02
p value t-test for S+ high proficient licks vs S+ high naive licks = 3.227294e-02

p values below are > pFDR

p value ranksum for S+ high naive peak vs S+ low naive peak = 4.836830e-02
p value t-test for S+ high naive licks vs S+ high naive peak = 7.080020e-02
p value t-test for S+ high naive licks vs S+ low naive licks = 1.279047e-01
p value t-test for S+ high naive licks vs S+ low naive peak = 1.574622e-01
p value t-test for S+ high naive licks vs S+ low proficient peak = 1.620438e-01
p value ranksum for S+ low naive licks vs S+ low naive peak = 2.505828e-01
p value t-test for S+ high proficient licks vs S+ low proficient licks = 3.547780e-01
p value t-test for S+ high proficient licks vs S+ high proficient peak = 6.036451e-01
p value ranksum for S+ high proficient peak vs S+ low proficient licks = 8.574981e-01

Ranksum or t-test p values for decision times for trough for Theta/High Gamma

pFDR = 3.035714e-02

p value t-test for S+ high proficient licks vs S+ high naive trough = 4.591330e-12
p value t-test for S+ low proficient licks vs S+ low naive licks = 3.519123e-08
p value t-test for S+ low proficient licks vs S+ low naive trough = 6.696581e-07
p value t-test for S+ high proficient licks vs S+ low naive licks = 2.544611e-05
p value t-test for S+ high proficient licks vs S+ low naive trough = 1.399027e-04
p value ranksum for S+ high naive trough vs S+ low proficient licks = 3.108003e-04
p value ranksum for S+ high proficient trough vs S+ high naive trough = 5.827506e-04
p value ranksum for S+ high naive trough vs S+ low naive licks = 5.827506e-04
p value ranksum for S+ high proficient trough vs S+ low naive trough = 1.165501e-03
p value ranksum for S+ high proficient trough vs S+ low naive licks = 1.165501e-03
p value t-test for S+ high naive licks vs S+ high naive trough = 4.355537e-03
p value t-test for S+ high proficient licks vs S+ low proficient trough = 6.444888e-03
p value t-test for S+ high naive licks vs S+ low naive trough = 9.031325e-03
p value t-test for S+ low proficient licks vs S+ low proficient trough = 9.145467e-03
p value ranksum for S+ high proficient trough vs S+ low proficient trough = 1.087801e-02
p value t-test for S+ high naive licks vs S+ low proficient licks = 1.253333e-02
p value ranksum for S+ low naive licks vs S+ low naive trough = 2.214452e-02

p values below are > pFDR

p value t-test for S+ high proficient licks vs S+ high naive licks = 3.227294e-02
p value ranksum for S+ high proficient trough vs S+ low proficient licks = 1.013209e-01
p value ranksum for S+ high naive trough vs S+ low proficient trough = 1.025641e-01
p value ranksum for S+ low proficient trough vs S+ low naive trough = 1.025641e-01
p value ranksum for S+ high proficient trough vs S+ high naive licks = 1.037296e-01
p value t-test for S+ high naive licks vs S+ low naive licks = 1.279047e-01
p value t-test for S+ high proficient licks vs S+ high proficient trough = 1.346417e-01
p value t-test for S+ high proficient licks vs S+ low proficient licks = 3.547780e-01
p value t-test for S+ high naive licks vs S+ low proficient trough = 4.157137e-01
p value ranksum for S+ high naive trough vs S+ low naive trough = 1
p value ranksum for S+ low proficient trough vs S+ low naive licks = 1
