# Supplementary material for: The Olfactory Bulb Facilitates Use of Category Bounds for Classification of Odorants in Different Intensity Groups
Source: Front Cell Neurosci. 2020 Dec 11;14:613635. doi: 10.3389/fncel.2020.613635 (PMC7759615; doi:10.3389/fncel.2020.613635)
Supplement: Supplementary file 14 [file Table_14.pdf]

Table S14. Generalized linear regression model for Figure 7A, percent correct behavior for different dilutions and different reward concentration range for proficient mice.

percent_correct: percent_correct behavior
rewarded_stimulus: S+ high vs. S+ low
concentration: log10(c_{liq})

Generalized linear regression model:

percent_correct ~ rewarded_stimulus + concentration + rewarded_stimulus*concentration

Distribution = Normal

Estimated Coefficients:

	Estimate	SE	tStat	pValue
(Intercept)	86.423	2.5129	34.392	1.0798e-49
rewarded_stimulus_2	-9.3646	3.3243	-2.8171	0.0061029
concentration	6.2397	2.8288	2.2058	0.030271
rewarded_stimulus_2:concentration	-12.041	3.7422	-3.2176	0.0018673

84 observations, 80 error degrees of freedom
Estimated Dispersion: 210
F-statistic vs. constant model: 4.82, p-value = 0.00388

Ranksum or t-test p values for performance PRP peak

pFDR = 1.590909e-02

p value t-test for S+ high 10 vs S+ low 1 = 6.197236e-05
p value t-test for S+ high 1 vs S+ low 1 = 2.028441e-04
p value t-test for S+ low 0.1 vs S+ low 3.2 = 3.511489e-04
p value t-test for S+ low 0.32 vs S+ low 1 = 5.866567e-04
p value ranksum for S+ high 3.2 vs S+ low 1 = 6.660007e-04
p value t-test for S+ low 0.1 vs S+ low 1 = 9.753371e-04
p value t-test for S+ high 0.1 vs S+ low 1 = 1.368494e-03
p value t-test for S+ high 0.32 vs S+ high 10 = 2.226203e-03
p value t-test for S+ high 0.1 vs S+ high 10 = 3.031361e-03
p value t-test for S+ high 0.032 vs S+ low 1 = 4.378465e-03
p value t-test for S+ high 10 vs S+ low 3.2 = 4.486540e-03
p value t-test for S+ low 0.032 vs S+ low 1 = 4.572085e-03
p value t-test for S+ low 1 vs S+ low 10 = 5.843911e-03
p value t-test for S+ low 0.32 vs S+ low 3.2 = 7.348578e-03
p value t-test for S+ high 0.32 vs S+ low 0.1 = 9.108927e-03

p value t-test for S+ high 0.32 vs S+ low 0.32 = 1.090341e-02
p value t-test for S+ high 1 vs S+ low 3.2 = 1.274534e-02
p value t-test for S+ high 0.032 vs S+ high 10 = 1.380549e-02
p value t-test for S+ high 0.032 vs S+ high 1 = 1.391562e-02
p value t-test for S+ high 0.32 vs S+ high 1 = 1.397453e-02
p value t-test for S+ low 0.032 vs S+ low 3.2 = 1.479060e-02

p values below are > pFDR

p value t-test for S+ high 0.1 vs S+ high 1 = 2.327960e-02
p value ranksum for S+ high 3.2 vs S+ low 3.2 = 2.930403e-02
p value t-test for S+ high 0.32 vs S+ high 3.2 = 3.464817e-02
p value t-test for S+ high 0.32 vs S+ low 1 = 3.626457e-02
p value t-test for S+ low 3.2 vs S+ low 10 = 5.017965e-02
p value t-test for S+ high 10 vs S+ low 10 = 5.939058e-02
p value t-test for S+ high 0.1 vs S+ high 0.32 = 6.551260e-02
p value t-test for S+ high 0.32 vs S+ low 10 = 6.599536e-02
p value t-test for S+ high 0.32 vs S+ low 0.032 = 6.661982e-02
p value t-test for S+ low 1 vs S+ low 3.2 = 7.440746e-02
p value t-test for S+ high 0.1 vs S+ low 3.2 = 1.055446e-01
p value t-test for S+ high 0.1 vs S+ high 3.2 = 1.389751e-01
p value t-test for S+ high 0.032 vs S+ high 0.32 = 1.517497e-01
p value t-test for S+ high 0.032 vs S+ low 0.1 = 1.738664e-01
p value t-test for S+ high 0.1 vs S+ low 0.1 = 1.763565e-01
p value t-test for S+ high 0.032 vs S+ low 0.32 = 1.900597e-01
p value t-test for S+ high 1 vs S+ low 10 = 1.900658e-01
p value t-test for S+ high 0.032 vs S+ low 3.2 = 1.954901e-01
p value t-test for S+ high 0.1 vs S+ low 0.32 = 1.987343e-01
p value t-test for S+ high 10 vs S+ low 0.032 = 2.158387e-01
p value t-test for S+ high 0.032 vs S+ high 3.2 = 2.159586e-01
p value ranksum for S+ high 3.2 vs S+ low 10 = 2.284382e-01
p value t-test for S+ high 1 vs S+ high 10 = 2.845086e-01
p value t-test for S+ low 0.32 vs S+ low 10 = 2.850467e-01
p value t-test for S+ low 0.032 vs S+ low 0.1 = 3.228048e-01
p value t-test for S+ high 10 vs S+ low 0.1 = 3.256758e-01
p value t-test for S+ high 10 vs S+ low 0.32 = 3.263131e-01
p value t-test for S+ low 0.1 vs S+ low 10 = 3.468508e-01
p value t-test for S+ high 1 vs S+ low 0.032 = 4.236869e-01
p value t-test for S+ high 0.032 vs S+ low 0.032 = 4.934520e-01
p value t-test for S+ low 0.032 vs S+ low 0.32 = 5.383338e-01
p value t-test for S+ high 0.1 vs S+ low 0.032 = 5.933036e-01
p value t-test for S+ high 0.032 vs S+ low 10 = 6.393657e-01
p value ranksum for S+ high 3.2 vs S+ low 0.032 = 6.620047e-01
p value t-test for S+ high 1 vs S+ low 0.32 = 6.788112e-01
p value t-test for S+ high 1 vs S+ low 0.1 = 6.925101e-01
p value t-test for S+ high 0.032 vs S+ high 0.1 = 7.141343e-01
p value ranksum for S+ high 3.2 vs S+ low 0.32 = 7.545788e-01
p value t-test for S+ low 0.032 vs S+ low 10 = 7.568185e-01
p value t-test for S+ high 0.1 vs S+ low 10 = 7.958690e-01
p value ranksum for S+ high 3.2 vs S+ high 10 = 8.181818e-01
p value t-test for S+ high 0.32 vs S+ low 3.2 = 8.389730e-01

p value t-test for S+ high 1 vs S+ high 3.2 = 9.515091e-01
p value t-test for S+ low 0.1 vs S+ low 0.32 = 9.684046e-01
p value ranksum for S+ high 3.2 vs S+ low 0.1 = 1
