# Supplementary material for: The Olfactory Bulb Facilitates Use of Category Bounds for Classification of Odorants in Different Intensity Groups
Source: Front Cell Neurosci. 2020 Dec 11;14:613635. doi: 10.3389/fncel.2020.613635 (PMC7759615; doi:10.3389/fncel.2020.613635)
Supplement: Supplementary file 15 [file Table_15.pdf]

Table S15. Generalized linear regression model for Figure 7B, decoding performance from trough beta tPRP for different dilutions and different reward concentration range for proficient mice.

percent_correct: performance
rewarded_stimulus: S+ high vs. S+ low
concentration: log10(c_{liq})

Generalized linear regression model:

percent_correct ~ 1 + rewarded_stimulus*concentration

Distribution = Normal

Estimated Coefficients:

	Estimate	SE	tStat	pValue
(Intercept)	85.693	2.608	32.857	3.2767e-48
rewarded_stimulus_2	-9.0887	3.4501	-2.6343	0.010119
concentration	6.5646	2.9359	2.2359	0.028142
rewarded_stimulus_2:concentration	-12.732	3.8839	-3.2781	0.0015478

84 observations, 80 error degrees of freedom
Estimated Dispersion: 226
F-statistic vs. constant model: 4.7, p-value = 0.00451

Ranksum or t-test p values for performance PRP trough

pFDR = 1.742424e-02

p value t-test for S+ high 0.1 vs S+ high 10 = 4.275173e-04
p value t-test for S+ high 10 vs S+ low 1 = 7.806227e-04
p value t-test for S+ low 0.1 vs S+ low 3.2 = 8.602360e-04
p value ranksum for S+ high 1 vs S+ low 1 = 1.332001e-03
p value t-test for S+ high 3.2 vs S+ low 1 = 1.355498e-03
p value t-test for S+ high 0.32 vs S+ low 0.1 = 1.928740e-03
p value t-test for S+ high 0.1 vs S+ high 1 = 2.053236e-03
p value t-test for S+ low 0.1 vs S+ low 1 = 2.205180e-03
p value t-test for S+ high 0.32 vs S+ high 10 = 2.281161e-03
p value t-test for S+ high 10 vs S+ low 3.2 = 2.614974e-03
p value t-test for S+ low 0.32 vs S+ low 1 = 3.844791e-03
p value ranksum for S+ high 1 vs S+ low 3.2 = 4.662005e-03
p value t-test for S+ low 0.032 vs S+ low 1 = 5.164684e-03
p value t-test for S+ low 0.032 vs S+ low 3.2 = 5.842041e-03
p value t-test for S+ high 3.2 vs S+ low 3.2 = 5.941231e-03

p value t-test for S+ low 1 vs S+ low 10 = 8.696786e-03
p value t-test for S+ high 0.1 vs S+ low 1 = 9.326518e-03
p value t-test for S+ high 0.032 vs S+ high 10 = 9.921597e-03
p value t-test for S+ high 0.32 vs S+ high 1 = 1.030723e-02
p value t-test for S+ high 0.32 vs S+ low 10 = 1.123389e-02
p value t-test for S+ high 0.032 vs S+ high 1 = 1.405012e-02
p value t-test for S+ high 0.032 vs S+ low 1 = 1.637316e-02
p value t-test for S+ low 3.2 vs S+ low 10 = 1.658962e-02

p values below are > pFDR

p value t-test for S+ high 0.32 vs S+ low 0.032 = 1.821870e-02
p value t-test for S+ low 0.32 vs S+ low 3.2 = 2.140985e-02
p value t-test for S+ high 0.1 vs S+ low 0.1 = 2.296833e-02
p value t-test for S+ high 0.32 vs S+ high 3.2 = 2.795461e-02
p value t-test for S+ high 10 vs S+ low 10 = 4.690139e-02
p value t-test for S+ high 0.1 vs S+ high 3.2 = 4.691845e-02
p value t-test for S+ high 0.032 vs S+ low 0.1 = 4.877195e-02
p value t-test for S+ high 0.1 vs S+ high 0.32 = 7.755302e-02
p value t-test for S+ high 0.32 vs S+ low 0.32 = 8.107104e-02
p value t-test for S+ high 0.1 vs S+ low 3.2 = 8.542417e-02
p value t-test for S+ high 0.32 vs S+ low 1 = 8.655218e-02
p value t-test for S+ high 0.032 vs S+ high 3.2 = 1.315519e-01
p value t-test for S+ low 0.032 vs S+ low 0.1 = 1.318152e-01
p value t-test for S+ high 0.032 vs S+ low 3.2 = 1.545757e-01
p value t-test for S+ low 1 vs S+ low 3.2 = 1.590365e-01
p value t-test for S+ high 3.2 vs S+ low 10 = 1.789244e-01
p value t-test for S+ high 0.032 vs S+ high 0.32 = 1.831199e-01
p value t-test for S+ high 0.1 vs S+ low 0.032 = 2.192740e-01
p value t-test for S+ high 0.1 vs S+ low 10 = 2.290472e-01
p value t-test for S+ high 0.032 vs S+ low 0.032 = 2.303477e-01
p value ranksum for S+ high 1 vs S+ low 10 = 2.390942e-01
p value t-test for S+ low 0.1 vs S+ low 10 = 2.658109e-01
p value t-test for S+ high 0.032 vs S+ low 10 = 2.736841e-01
p value t-test for S+ high 10 vs S+ low 0.32 = 2.835149e-01
p value t-test for S+ high 10 vs S+ low 0.032 = 2.931513e-01
p value t-test for S+ low 0.1 vs S+ low 0.32 = 3.170312e-01
p value ranksum for S+ high 1 vs S+ low 0.32 = 3.922744e-01
p value t-test for S+ high 3.2 vs S+ low 0.32 = 4.552627e-01
p value ranksum for S+ high 1 vs S+ low 0.032 = 4.675325e-01
p value t-test for S+ high 0.032 vs S+ low 0.32 = 4.766303e-01
p value t-test for S+ high 0.1 vs S+ low 0.32 = 5.222477e-01
p value t-test for S+ high 3.2 vs S+ low 0.032 = 5.445926e-01
p value ranksum for S+ high 1 vs S+ high 3.2 = 5.562771e-01
p value t-test for S+ high 3.2 vs S+ high 10 = 5.848340e-01
p value t-test for S+ high 10 vs S+ low 0.1 = 6.026653e-01
p value t-test for S+ low 0.032 vs S+ low 0.32 = 7.182533e-01
p value t-test for S+ low 0.032 vs S+ low 10 = 7.201408e-01
p value t-test for S+ high 0.32 vs S+ low 3.2 = 7.702902e-01
p value t-test for S+ high 0.032 vs S+ high 0.1 = 7.834078e-01
p value t-test for S+ high 3.2 vs S+ low 0.1 = 9.582097e-01

p value t-test for S+ low 0.32 vs S+ low 10 = 9.969905e-01
p value ranksum for S+ high 1 vs S+ high 10 = 1
p value ranksum for S+ high 1 vs S+ low 0.1 = 1

Table S16. Generalized linear regression model for Figure 7C, decoding performance from peak beta tPRP for different dilutions and different reward concentration range for proficient mice.

percent_correct: performance
rewarded_stimulus: S+ high vs. S+ low
concentration: log10(c_{liq})

Generalized linear regression model:

percent_correct ~ 1 + rewarded_stimulus*concentration

Distribution = Normal

Estimated Coefficients:

	Estimate	SE	tStat	pValue
(Intercept)	89.426	3.0576	29.248	1.7832e-44
rewarded_stimulus_2	-6.9863	4.0448	-1.7272	0.087985
concentration	6.3614	3.442	1.8482	0.068269
rewarded_stimulus_2:concentration	-16.227	4.5533	-3.5637	0.00061999

84 observations, 80 error degrees of freedom
Estimated Dispersion: 310
F-statistic vs. constant model: 4.98, p-value = 0.0032

Ranksum or t-test p values for for behavioral percent correct

pFDR = 2.348485e-02

p value t-test for S+ high 0.32 vs S+ low 0.1 = 6.973347e-05
p value t-test for S+ high 0.32 vs S+ low 0.32 = 1.088867e-04
p value t-test for S+ high 0.32 vs S+ low 0.032 = 1.257214e-04
p value ranksum for S+ low 0.032 vs S+ low 1 = 1.554002e-04
p value ranksum for S+ low 0.1 vs S+ low 1 = 1.554002e-04
p value ranksum for S+ low 0.32 vs S+ low 1 = 1.554002e-04
p value t-test for S+ high 10 vs S+ low 1 = 5.454719e-04
p value t-test for S+ high 0.1 vs S+ low 0.1 = 8.000025e-04
p value t-test for S+ high 1 vs S+ low 1 = 1.228219e-03
p value t-test for S+ high 3.2 vs S+ low 1 = 1.265312e-03
p value ranksum for S+ low 0.032 vs S+ low 3.2 = 1.398601e-03
p value ranksum for S+ low 0.1 vs S+ low 3.2 = 1.398601e-03
p value t-test for S+ low 1 vs S+ low 10 = 1.597567e-03
p value t-test for S+ high 1 vs S+ low 0.1 = 1.799570e-03

p value t-test for S+ high 0.1 vs S+ low 0.32 = 1.812981e-03
p value ranksum for S+ low 0.32 vs S+ low 3.2 = 2.020202e-03
p value t-test for S+ high 0.1 vs S+ low 0.032 = 2.358235e-03
p value t-test for S+ high 10 vs S+ low 3.2 = 3.335819e-03
p value t-test for S+ high 0.32 vs S+ low 10 = 3.346744e-03
p value t-test for S+ high 0.1 vs S+ low 1 = 4.674206e-03
p value t-test for S+ high 0.032 vs S+ low 0.1 = 9.378124e-03
p value t-test for S+ high 1 vs S+ low 0.32 = 9.769418e-03
p value t-test for S+ high 1 vs S+ low 3.2 = 9.977639e-03
p value t-test for S+ high 0.32 vs S+ high 10 = 1.019184e-02
p value t-test for S+ high 3.2 vs S+ low 3.2 = 1.024581e-02
p value t-test for S+ high 0.032 vs S+ low 1 = 1.256575e-02
p value t-test for S+ high 0.032 vs S+ low 0.32 = 1.369850e-02
p value t-test for S+ high 0.032 vs S+ low 0.032 = 1.452099e-02
p value t-test for S+ high 1 vs S+ low 0.032 = 1.651341e-02
p value t-test for S+ high 0.1 vs S+ high 0.32 = 1.973287e-02
p value t-test for S+ low 3.2 vs S+ low 10 = 2.060732e-02

p values below are > pFDR

p value t-test for S+ high 0.32 vs S+ high 1 = 2.469813e-02
p value ranksum for S+ low 0.1 vs S+ low 10 = 2.564103e-02
p value t-test for S+ high 3.2 vs S+ low 0.1 = 3.485725e-02
p value t-test for S+ high 10 vs S+ low 0.1 = 3.975030e-02
p value t-test for S+ high 0.32 vs S+ high 3.2 = 4.045224e-02
p value t-test for S+ high 0.1 vs S+ high 10 = 4.814461e-02
p value t-test for S+ high 0.1 vs S+ low 3.2 = 5.340588e-02
p value ranksum for S+ low 0.032 vs S+ low 10 = 6.293706e-02
p value ranksum for S+ low 0.32 vs S+ low 10 = 6.666667e-02
p value t-test for S+ high 0.32 vs S+ low 1 = 6.920469e-02
p value t-test for S+ high 1 vs S+ high 10 = 7.752921e-02
p value t-test for S+ high 3.2 vs S+ low 0.32 = 7.815169e-02
p value t-test for S+ high 0.032 vs S+ high 10 = 7.951179e-02
p value t-test for S+ high 3.2 vs S+ low 0.032 = 9.029969e-02
p value t-test for S+ high 0.1 vs S+ high 1 = 1.282671e-01
p value t-test for S+ high 0.032 vs S+ high 0.32 = 1.328991e-01
p value t-test for S+ high 0.032 vs S+ low 3.2 = 1.414066e-01
p value t-test for S+ high 10 vs S+ low 10 = 1.533754e-01
p value t-test for S+ high 0.032 vs S+ high 1 = 1.610899e-01
p value t-test for S+ high 0.032 vs S+ low 10 = 1.622027e-01
p value t-test for S+ low 1 vs S+ low 3.2 = 1.665064e-01
p value t-test for S+ high 0.1 vs S+ low 10 = 1.869959e-01
p value t-test for S+ high 3.2 vs S+ high 10 = 2.083573e-01
p value t-test for S+ high 0.032 vs S+ high 3.2 = 2.550839e-01
p value t-test for S+ high 0.1 vs S+ high 3.2 = 2.933496e-01
p value t-test for S+ high 10 vs S+ low 0.32 = 3.130419e-01
p value t-test for S+ high 10 vs S+ low 0.032 = 3.877701e-01
p value ranksum for S+ low 0.1 vs S+ low 0.32 = 4.666667e-01
p value t-test for S+ high 0.032 vs S+ high 0.1 = 5.435941e-01
p value t-test for S+ high 0.32 vs S+ low 3.2 = 6.952260e-01
p value t-test for S+ high 3.2 vs S+ low 10 = 7.786168e-01

p value t-test for S+ high 1 vs S+ high 3.2 = 8.530366e-01
p value t-test for S+ high 1 vs S+ low 10 = 8.719961e-01
p value ranksum for S+ low 0.032 vs S+ low 0.1 = 1
p value ranksum for S+ low 0.032 vs S+ low 0.32 = 1

Table S17. Generalized linear regression model for Figure 7D, decoding performance from trough gamma tPRP for different dilutions and different reward concentration range for proficient mice.

percent_correct: performance
rewarded_stimulus: S+ high vs. S+ low
concentration: log10(c_{liq})

Generalized linear regression model:

percent_correct ~ 1 + rewarded_stimulus*concentration

Distribution = Normal

Estimated Coefficients:

	Estimate	SE	tStat	pValue
(Intercept)	87.168	2.4539	35.523	9.4931e-51
rewarded_stimulus_2	-8.6307	3.2462	-2.6587	0.00947
concentration	2.8671	2.7624	1.0379	0.30244
rewarded_stimulus_2:concentration	-6.5797	3.6543	-1.8005	0.075544

84 observations, 80 error degrees of freedom
Estimated Dispersion: 200
F-statistic vs. constant model: 2.84, p-value = 0.0429

Ranksum or t-test p values for performance PRP peak

pFDR = 2.272727e-03

p value t-test for S+ high 10 vs S+ low 1 = 9.214445e-04
p value t-test for S+ low 0.32 vs S+ low 1 = 1.045137e-03
p value t-test for S+ high 1 vs S+ low 1 = 2.116638e-03

p values below are > pFDR

p value t-test for S+ high 0.1 vs S+ low 1 = 3.507690e-03
p value t-test for S+ high 3.2 vs S+ low 1 = 4.173494e-03
p value ranksum for S+ low 0.1 vs S+ low 1 = 4.662005e-03
p value t-test for S+ low 1 vs S+ low 10 = 6.273991e-03
p value t-test for S+ low 0.032 vs S+ low 1 = 6.554917e-03
p value t-test for S+ high 0.32 vs S+ high 10 = 9.137966e-03
p value t-test for S+ high 0.032 vs S+ low 1 = 9.235680e-03
p value t-test for S+ high 10 vs S+ low 3.2 = 2.068718e-02
p value t-test for S+ high 0.32 vs S+ high 1 = 3.388814e-02

p value t-test for S+ low 0.32 vs S+ low 3.2 = 3.513617e-02
p value t-test for S+ high 0.1 vs S+ high 0.32 = 4.124002e-02
p value t-test for S+ high 1 vs S+ low 3.2 = 4.895494e-02
p value t-test for S+ high 0.1 vs S+ high 10 = 4.982036e-02
p value t-test for S+ high 0.32 vs S+ low 1 = 5.094336e-02
p value t-test for S+ low 0.032 vs S+ low 3.2 = 5.338320e-02
p value t-test for S+ low 3.2 vs S+ low 10 = 6.520494e-02
p value t-test for S+ low 1 vs S+ low 3.2 = 7.623335e-02
p value t-test for S+ high 10 vs S+ low 10 = 7.813081e-02
p value t-test for S+ high 0.1 vs S+ low 3.2 = 8.643161e-02
p value t-test for S+ high 0.32 vs S+ low 0.1 = 8.992225e-02
p value t-test for S+ high 3.2 vs S+ low 3.2 = 9.100605e-02
p value t-test for S+ high 0.032 vs S+ high 10 = 9.639036e-02
p value ranksum for S+ low 0.1 vs S+ low 3.2 = 1.048951e-01
p value t-test for S+ high 0.32 vs S+ low 10 = 1.168635e-01
p value t-test for S+ high 0.1 vs S+ high 1 = 1.211051e-01
p value t-test for S+ high 0.032 vs S+ high 1 = 1.219508e-01
p value t-test for S+ high 0.032 vs S+ high 0.32 = 1.775010e-01
p value t-test for S+ high 0.032 vs S+ low 3.2 = 1.815640e-01
p value t-test for S+ high 10 vs S+ low 0.032 = 1.955971e-01
p value t-test for S+ high 0.32 vs S+ high 3.2 = 2.286055e-01
p value t-test for S+ high 0.32 vs S+ low 0.32 = 2.372893e-01
p value t-test for S+ high 1 vs S+ high 10 = 2.441947e-01
p value t-test for S+ high 10 vs S+ low 0.32 = 2.905737e-01
p value t-test for S+ low 0.032 vs S+ low 0.1 = 2.960255e-01
p value t-test for S+ high 0.32 vs S+ low 0.032 = 3.227786e-01
p value t-test for S+ high 1 vs S+ low 10 = 3.266503e-01
p value ranksum for S+ low 0.1 vs S+ low 10 = 3.404817e-01
p value t-test for S+ high 3.2 vs S+ high 10 = 3.916442e-01
p value t-test for S+ high 1 vs S+ low 0.032 = 3.970193e-01
p value t-test for S+ high 10 vs S+ low 0.1 = 5.396662e-01
p value t-test for S+ high 1 vs S+ low 0.32 = 5.400889e-01
p value t-test for S+ high 0.032 vs S+ low 0.1 = 5.601733e-01
p value t-test for S+ high 0.032 vs S+ high 0.1 = 5.675631e-01
p value t-test for S+ high 3.2 vs S+ low 0.032 = 5.996208e-01
p value t-test for S+ high 0.1 vs S+ low 0.032 = 6.305971e-01
p value t-test for S+ high 3.2 vs S+ low 10 = 6.394502e-01
p value t-test for S+ high 0.1 vs S+ low 10 = 6.747518e-01
p value t-test for S+ high 0.32 vs S+ low 3.2 = 7.022227e-01
p value t-test for S+ low 0.032 vs S+ low 10 = 7.111444e-01
p value t-test for S+ high 0.032 vs S+ high 3.2 = 7.311231e-01
p value t-test for S+ high 1 vs S+ high 3.2 = 7.543895e-01
p value t-test for S+ low 0.032 vs S+ low 0.32 = 7.568933e-01
p value t-test for S+ high 3.2 vs S+ low 0.32 = 7.634614e-01
p value t-test for S+ high 0.1 vs S+ low 0.1 = 7.670429e-01
p value t-test for S+ high 0.1 vs S+ low 0.32 = 8.105180e-01
p value t-test for S+ high 3.2 vs S+ low 0.1 = 8.515123e-01
p value ranksum for S+ low 0.1 vs S+ low 0.32 = 8.784771e-01
p value t-test for S+ high 1 vs S+ low 0.1 = 8.984098e-01
p value t-test for S+ high 0.032 vs S+ low 0.032 = 9.038892e-01
p value t-test for S+ high 0.032 vs S+ low 10 = 9.228006e-01

p value t-test for S+ high 0.032 vs S+ low 0.32 = 9.262571e-01
p value t-test for S+ high 0.1 vs S+ high 3.2 = 9.275861e-01
p value t-test for S+ low 0.32 vs S+ low 10 = 9.757174e-01

Table S18. Generalized linear regression model for Figure 7E, decoding performance from peak gamma tPRP for different dilutions and different reward concentration range for proficient mice.

percent_correct: performance
rewarded_stimulus: S+ high vs. S+ low
concentration: log10(c_{liq})

Generalized linear regression model:

percent_correct ~ 1 + rewarded_stimulus*concentration

Distribution = Normal

Estimated Coefficients:

	Estimate	SE	tStat	pValue
(Intercept)	66.689	2.2355	29.831	4.1863e-45
rewarded_stimulus_2	-2.9874	2.9573	-1.0102	0.31546
concentration	1.1073	2.5166	0.44001	0.66111
rewarded_stimulus_2:concentration	-1.9384	3.3291	-0.58226	0.56203

84 observations, 80 error degrees of freedom
Estimated Dispersion: 166
F-statistic vs. constant model: 0.373, p-value = 0.773

Ranksum or t-test p values for performance PRP trough

pFDR = 7.575758e-04

p values below are > pFDR

p value t-test for S+ low 1 vs S+ low 10 = 1.001714e-03
p value t-test for S+ low 0.1 vs S+ low 1 = 7.664337e-03
p value t-test for S+ low 1 vs S+ low 3.2 = 1.152265e-02
p value t-test for S+ high 10 vs S+ low 1 = 1.401693e-02
p value t-test for S+ low 0.32 vs S+ low 1 = 1.714500e-02
p value t-test for S+ high 3.2 vs S+ low 1 = 2.049895e-02
p value t-test for S+ high 1 vs S+ low 1 = 2.448640e-02
p value t-test for S+ low 0.032 vs S+ low 1 = 2.624250e-02
p value t-test for S+ high 0.1 vs S+ low 1 = 3.027459e-02
p value t-test for S+ high 0.032 vs S+ low 1 = 3.114465e-02
p value t-test for S+ high 0.32 vs S+ high 10 = 3.785846e-02
p value t-test for S+ high 0.032 vs S+ high 0.32 = 4.672511e-02

p value t-test for S+ low 3.2 vs S+ low 10 = 7.269387e-02
p value t-test for S+ high 0.32 vs S+ high 3.2 = 8.726529e-02
p value t-test for S+ high 0.1 vs S+ high 0.32 = 9.601021e-02
p value t-test for S+ high 0.32 vs S+ high 1 = 9.719933e-02
p value t-test for S+ high 0.32 vs S+ low 10 = 1.666844e-01
p value t-test for S+ high 0.32 vs S+ low 1 = 1.848783e-01
p value t-test for S+ low 0.32 vs S+ low 10 = 1.991510e-01
p value t-test for S+ high 10 vs S+ low 3.2 = 2.136656e-01
p value t-test for S+ low 0.1 vs S+ low 3.2 = 2.152828e-01
p value t-test for S+ low 0.1 vs S+ low 0.32 = 2.318918e-01
p value t-test for S+ low 0.032 vs S+ low 3.2 = 2.343478e-01
p value t-test for S+ high 0.32 vs S+ low 0.032 = 2.587420e-01
p value t-test for S+ high 0.32 vs S+ low 0.1 = 2.614628e-01
p value t-test for S+ high 1 vs S+ low 3.2 = 3.082649e-01
p value t-test for S+ high 3.2 vs S+ low 3.2 = 3.294222e-01
p value t-test for S+ high 0.1 vs S+ low 3.2 = 3.947784e-01
p value t-test for S+ high 0.032 vs S+ low 3.2 = 3.972193e-01
p value t-test for S+ high 3.2 vs S+ high 10 = 4.596233e-01
p value t-test for S+ low 0.032 vs S+ low 0.32 = 4.779533e-01
p value t-test for S+ low 0.1 vs S+ low 10 = 5.403700e-01
p value t-test for S+ high 10 vs S+ low 0.32 = 5.681404e-01
p value t-test for S+ high 0.32 vs S+ low 0.32 = 5.944785e-01
p value t-test for S+ low 0.32 vs S+ low 3.2 = 6.069022e-01
p value t-test for S+ high 0.1 vs S+ high 10 = 6.158871e-01
p value t-test for S+ high 0.1 vs S+ low 10 = 6.268453e-01
p value t-test for S+ high 0.032 vs S+ high 10 = 6.289336e-01
p value t-test for S+ high 0.032 vs S+ low 10 = 6.311818e-01
p value t-test for S+ high 3.2 vs S+ low 10 = 6.361375e-01
p value t-test for S+ low 0.032 vs S+ low 10 = 6.395563e-01
p value t-test for S+ high 1 vs S+ low 0.32 = 6.528487e-01
p value t-test for S+ high 0.032 vs S+ high 1 = 7.111802e-01
p value t-test for S+ high 0.32 vs S+ low 3.2 = 7.172874e-01
p value t-test for S+ high 3.2 vs S+ low 0.32 = 7.230510e-01
p value t-test for S+ high 0.1 vs S+ high 1 = 7.403389e-01
p value t-test for S+ high 1 vs S+ low 10 = 7.546642e-01
p value t-test for S+ high 0.032 vs S+ low 0.32 = 7.567525e-01
p value t-test for S+ high 0.1 vs S+ low 0.32 = 7.578457e-01
p value t-test for S+ high 1 vs S+ high 10 = 7.866493e-01
p value t-test for S+ high 10 vs S+ low 0.1 = 8.093702e-01
p value t-test for S+ high 1 vs S+ high 3.2 = 8.098793e-01
p value t-test for S+ high 10 vs S+ low 0.032 = 8.224387e-01
p value t-test for S+ high 10 vs S+ low 10 = 8.331710e-01
p value t-test for S+ high 1 vs S+ low 0.1 = 9.184762e-01
p value t-test for S+ high 0.1 vs S+ low 0.032 = 9.220790e-01
p value t-test for S+ high 0.032 vs S+ low 0.032 = 9.255609e-01
p value t-test for S+ high 1 vs S+ low 0.032 = 9.303501e-01
p value t-test for S+ high 0.1 vs S+ low 0.1 = 9.333401e-01
p value t-test for S+ high 0.1 vs S+ high 3.2 = 9.354779e-01
p value t-test for S+ high 0.032 vs S+ low 0.1 = 9.367686e-01
p value t-test for S+ high 3.2 vs S+ low 0.032 = 9.512003e-01
p value t-test for S+ high 0.032 vs S+ high 3.2 = 9.532486e-01

p value t-test for S+ high 3.2 vs S+ low 0.1 = 9.634191e-01
p value t-test for S+ low 0.032 vs S+ low 0.1 = 9.812784e-01
p value t-test for S+ high 0.032 vs S+ high 0.1 = 9.904394e-01

Table S19. Generalized linear regression model for Figure S2C, modulation index for gamma tPRP for different dilutions.

MI: modulation index
spm: S+ vs S-
conc: odorant dilution

Generalized linear regression model:
MI ~ 1 + spm + conc
Distribution = Normal

Estimated Coefficients:

	Estimate	SE	tStat	pValue
(Intercept)	0.010355	0.00086423	11.981	8.5447e-23
spm_1	0.00059027	0.0019296	0.30591	0.76016
conc	0.0021542	0.0011239	1.9166	0.057462

134 observations, 131 error degrees of freedom
Estimated Dispersion: 2.68e-05
F-statistic vs. constant model: 6.33, p-value = 0.00237

Ranksum or t-test p values for MI

pFDR = 1.000000e-02

p value ranksum for 0.1 vs 10 = 6.421430e-05
p value ranksum for 0.1 vs 3.2 = 4.253384e-03
p value ranksum for 0.032 vs 10 = 7.718472e-03

p values below are > pFDR

p value t-test for 1 vs 10 = 1.618364e-02
p value ranksum for 0.1 vs 0.32 = 2.457265e-02
p value ranksum for 0.1 vs 1 = 6.165312e-02
p value t-test for 0.32 vs 10 = 7.414093e-02
p value ranksum for 0.032 vs 3.2 = 8.235222e-02
p value ranksum for 3.2 vs 10 = 1.415789e-01
p value ranksum for 0.032 vs 0.1 = 1.508630e-01
p value t-test for 1 vs 3.2 = 2.313003e-01
p value ranksum for 0.032 vs 0.32 = 3.712989e-01
p value t-test for 0.32 vs 3.2 = 4.045467e-01
p value ranksum for 0.032 vs 1 = 6.014098e-01
p value t-test for 0.32 vs 1 = 6.564709e-01

Table S20. Generalized linear regression model for Figure S3C, modulation index for gamma tPRP for different dilutions.

MI: modulation index
spm: S+ vs S-
conc: odorant dilution

Generalized linear regression model:
MI ~ 1 + spm + conc
Distribution = Normal

Estimated Coefficients:

	Estimate	SE	tStat	pValue
(Intercept)	0.015772	0.0039755	3.9672	0.0001796
spm_1	-0.0052307	0.0063649	-0.82181	0.4141
conc	0.0052688	0.0035073	1.5022	0.13773

70 observations, 67 error degrees of freedom
Estimated Dispersion: 0.000122
F-statistic vs. constant model: 1.99, p-value = 0.144

Ranksum or t-test p values for MI

pFDR = 5.000000e-02

p values below are > pFDR

p value t-test for 1 vs 0.32 = 1.406246e-01
p value t-test for 1 vs 0.1 = 2.143311e-01
p value t-test for 0.32 vs 0.1 = 2.629057e-01
p value t-test for 1 vs 0.032 = 2.780378e-01
p value ranksum for 10 vs 1 = 3.100090e-01
p value ranksum for 10 vs 3.2 = 3.151656e-01
p value ranksum for 3.2 vs 0.32 = 3.364168e-01
p value ranksum for 3.2 vs 0.1 = 4.233160e-01
p value t-test for 0.32 vs 0.032 = 5.072941e-01
p value ranksum for 3.2 vs 1 = 5.213770e-01
p value ranksum for 3.2 vs 0.032 = 5.557506e-01
p value ranksum for 10 vs 0.032 = 7.824305e-01
p value t-test for 0.1 vs 0.032 = 8.187300e-01

p value ranksum for 10 vs 0.32 = 8.605031e-01
p value ranksum for 10 vs 0.1 = 9.209513e-01
