# Supplementary figures and images for: The Olfactory Bulb Facilitates Use of Category Bounds for Classification of Odorants in Different Intensity Groups
Source: Front Cell Neurosci. 2020 Dec 11;14:613635. doi: 10.3389/fncel.2020.613635 (PMC7759615; doi:10.3389/fncel.2020.613635)

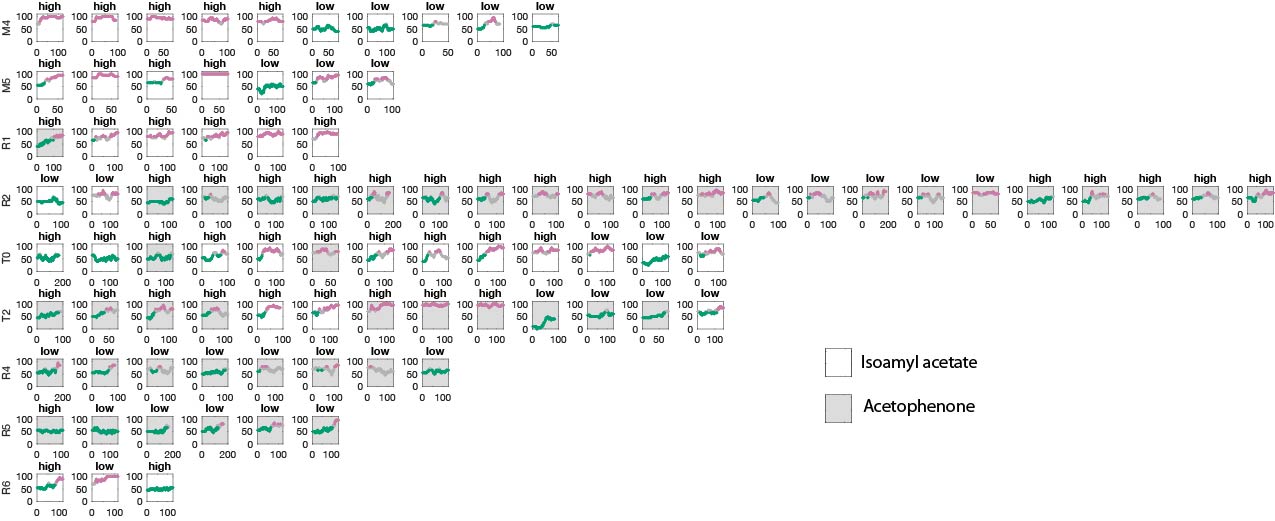

Supplement: Supplementary Figure 1 — Percent correct for the go-no go concentration task shown as a function of trial number for all sessions performed by the nine animals included in this study. Sessions are shown from first to last. The number of days between sessions is 1–5 days. The odorant group that was rewarded is shown above the plot for each session (high: 10, 3.3, and 1% cliq, low: 0.33, 0.1, and 0.033% cliq). [file Image_1.JPEG]

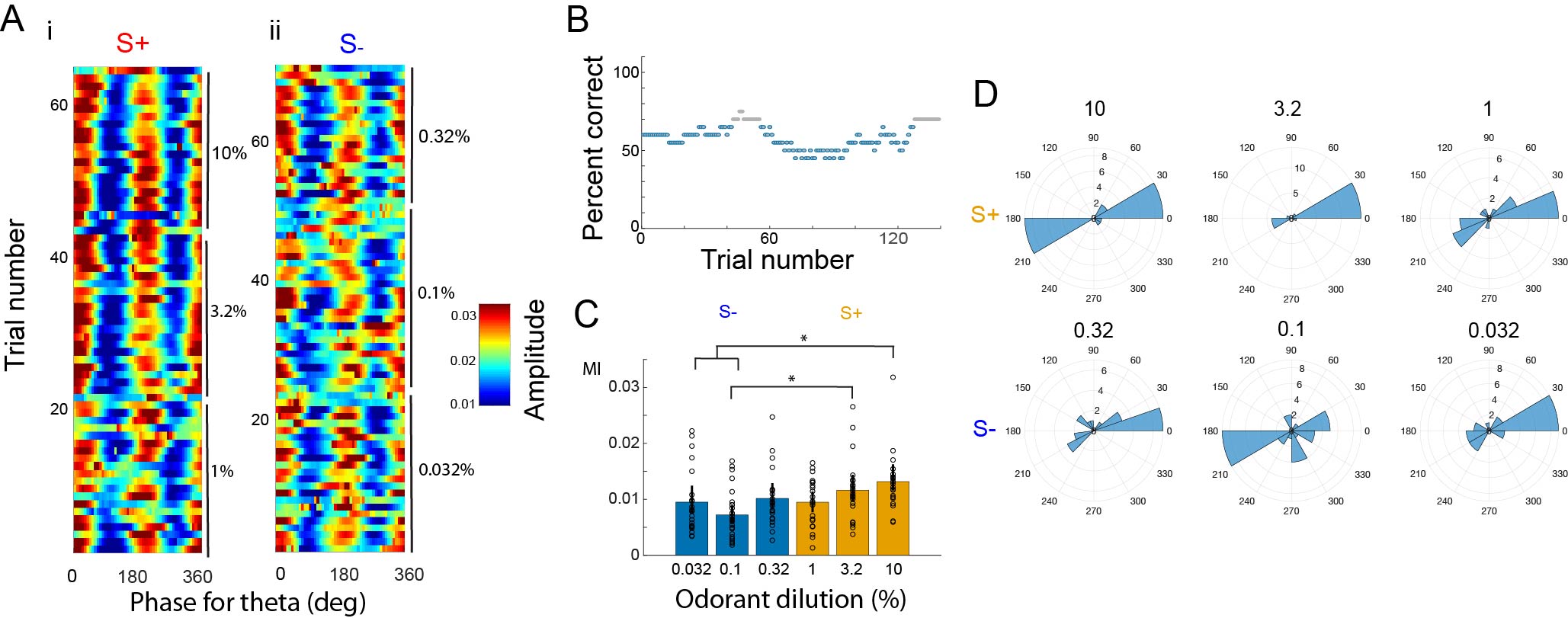

Supplement: Supplementary Figure 2 — Phase amplitude coupling for the go-no go odorant concentration task for a session where the mouse was naïve and the rewarded stimuli were high concentration odorants. (A–D) tPAC analysis shown for a go-no go session where the mouse was naïve in differentiating between the high concentration odorants (cliq 1–10%, rewarded stimulus, S+) and the low concentration odorants (cliq 0.033–0.33%, unrewarded stimulus, S-). (A) Pseudocolor image showing the per-trial average amplitude for the theta envelope for the gamma LFP. The odorant used was isoamyl acetate. Odorant dilutions (cliq) for S+ were 10, 3.33, and 1% and for S- were 0.33, 0.1, and 0.033%. (B) Percent correct as a function of trial number. Light gray: percent correct >65% and <80%, blue: percent correct ≤ 65%. (C) Strength of tPAC quantified as the modulation index (MI) displayed for the six odorant dilutions. A GLM analysis indicated that there is no difference between S+ and S- (p > 0.05, 134 trials, 131 d.f., F-Statistic 6.33, p-value for the model < 0.05, Supplementary Table 19). The asterisk denotes post-hoc differences evaluated with either t-test or ranksum, p < pFDR = 0.01. (D) Rose plot histograms for the peak phase angle for gamma tPAC shown in (A). [file Image_2.JPEG]

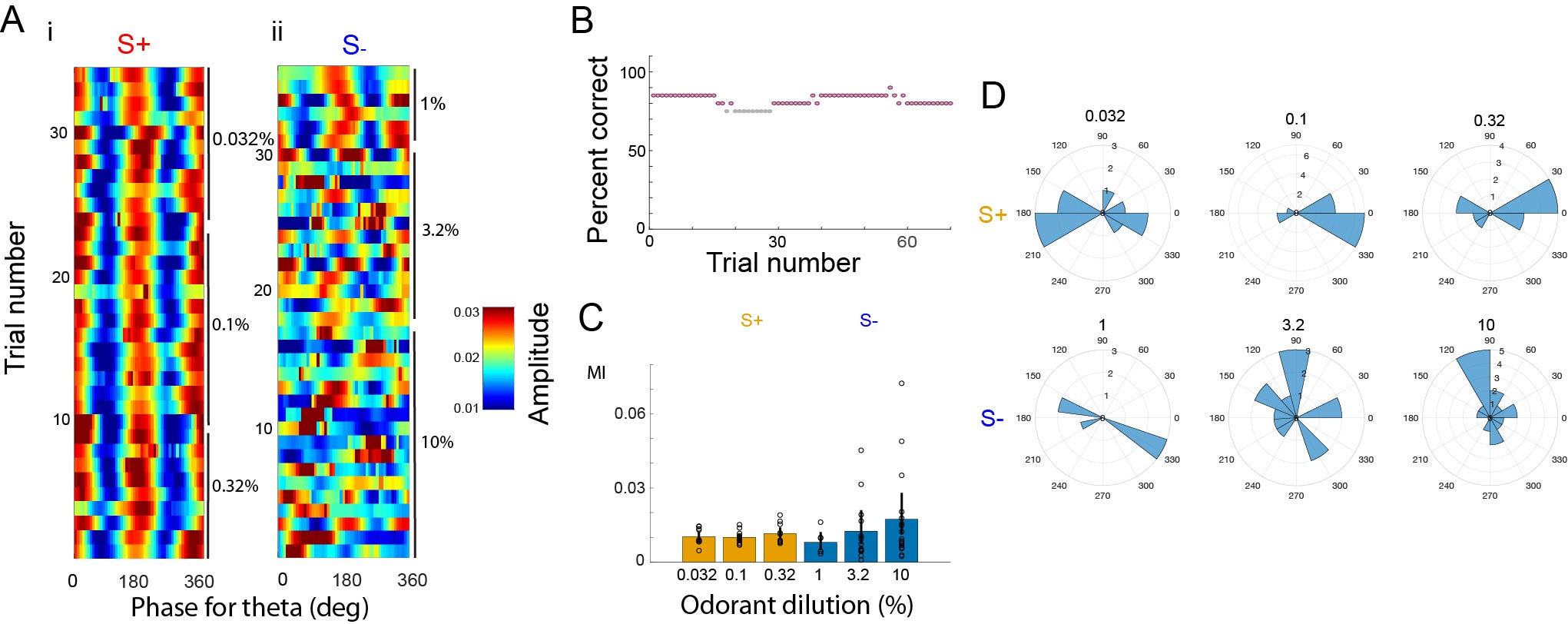

Supplement: Supplementary Figure 3 — Phase amplitude coupling for the go-no go odorant concentration task for a session where the mouse was proficient and the rewarded stimuli were low concentration odorants. (A–D) tPAC analysis shown for a go-no go session where the mouse was proficient in differentiating between the high concentration odorants (cliq 1–10%, rewarded stimulus, S-) and the low concentration odorants (cliq 0.033–0.33%, unrewarded stimulus, S+). (A) Pseudocolor image showing the per-trial average amplitude for the theta envelope for the gamma LFP. The odorant used was isoamyl acetate. Odorant dilutions (cliq) for S- were 10, 3.33, and 1% and for S+ were 0.33, 0.1, and 0.033%. (B) Percent correct as a function of trial number. Light gray: percent correct >65% and <80%, magenta: percent correct ≥ 80%. (C) Strength of tPAC quantified as the modulation index (MI) displayed for the six odorant dilutions. A GLM analysis indicated that there is no difference between S+ and S- (p > 0.05, 70 trials, 67 d.f., F-Statistic 1.99, p-value for the model > 0.05, Supplementary Table 20). (D) Rose plot histograms for the peak phase angle for gamma tPAC shown in (A). [file Image_3.JPEG]
